# Electro-mechanically switchable hydrocarbons based on [8]annulenes

**DOI:** 10.1038/s41467-022-28384-8

**Published:** 2022-02-14

**Authors:** Magdalena Tasić, Jakov Ivković, Göran Carlström, Michaela Melcher, Paolo Bollella, Jesper Bendix, Lo Gorton, Petter Persson, Jens Uhlig, Daniel Strand

**Affiliations:** 1 Centre for Analysis and Synthesis, Department of Chemistry, Lund University, Box 124, SE-221 00 Lund, Sweden; 2 Department of Analytical Chemistry/Biochemistry, Lund University, Box 124, SE-221 00 Lund, Sweden; 3 Department of Chemistry, University of Copenhagen, Universitetsparken 5, DK-2100 Copenhagen, Denmark; 4 Division of Theoretical Chemistry, Department of Chemistry, Lund University, Box 124, SE-221 00 Lund, Sweden; 5 Division of Chemical Physics, Department of Chemistry, Lund University, Box 124, SE-221 00 Lund, Sweden; 6 Present Address: Department of Chemistry, University of Bari “A. Moro”, Via E. Orabona 4, 70125 Bari, Italy

**Keywords:** Organic molecules in materials science, Organic molecules in materials science, Electronic materials

## Abstract

Pure hydrocarbons with shape and conjugation properties that can be switched by external stimuli is an intriguing prospect in the design of new responsive materials and single-molecule electronics. Here, we develop an oligomeric [8]annulene-based material that combines a remarkably efficient topological switching upon redox changes with structural simplicity, stability, and straightforward synthesis: 5,12-alkyne linked dibenzo[*a,e*]cyclooctatetraenes (dbCOTs). Upon reduction, the structures accommodate a reversible reorganization from a *pseudo*-conjugated tub-shape to a conjugated aromatic system. This switching in oligomeric structures gives rise to multiple defined states that are deconvoluted by electrochemical, NMR, and optical methods. The combination of stable electromechanical responsivity and ability to relay electrons stepwise through an extended (*pseudo*-conjugated) π-system in partially reduced structures validate alkyne linked dbCOTs as a practical platform for developing new responsive materials and switches based on [8]annulene cores.

## Abstract (web summary)

Pure hydrocarbons with properties that can be switched by external stimuli are interesting for the design and development of new responsive materials. Here, the authors develop an oligomeric [8]annulene-based material that combines topological switching upon redox changes with structural simplicity, stability, and straightforward synthesis.

## Introduction

**Fig. 1:**
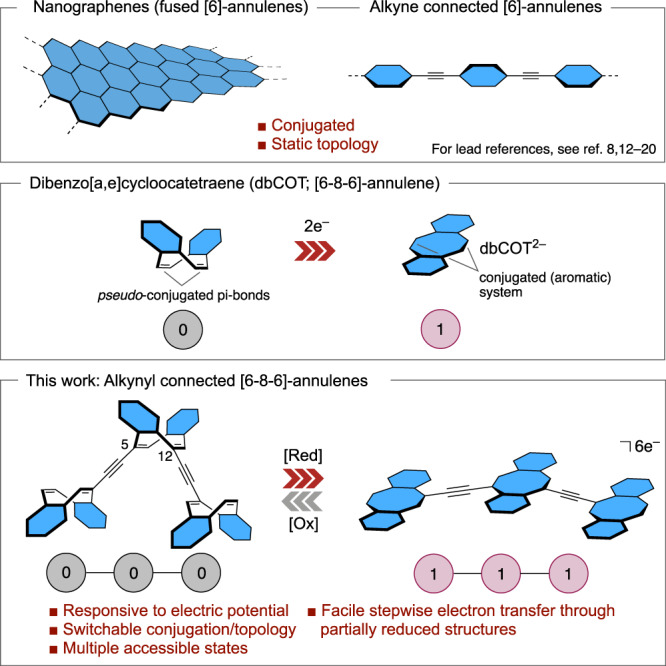
Conjugatively linked annulene systems. Aromatic rings are highlighted in blue. Colored circles show a Boolean logic of switching in [8]annulene units.

Miniaturization of components and their interconnections are critical factors in technological progress. Obvious examples include the evolution of silicon-based field-effect transistors and integrated circuits. Ultimately, switches^1, 2^, actuators^3–5^, and other responsive units^6,7^ based on single molecules are envisioned^8–11^. Considerable advances have been made recently in this direction, in no small part driven by the development of molecular junctions based on hydrocarbon structures like nanographene ribbons^8,12–16^ and variations of conjugatively linked aromatic systems^17–20^. Though favorable in many respects, the properties of such materials are, however, essentially static. Hydrocarbons with an intrinsic topological responsivity to electrical stimulus acting as conjugation switches or actuators is therefore an intriguing prospect. Not least as electric potential^21–23^ is singularly suited to integrated systems in that it can be reversibly delivered from small surfaces and also be relayed through a molecular framework to leverage the action of several responsive cores operating in consort. Cyclooctatetraene (COT)^24–28^, the [8]annulene homolog to benzene, suggests ample potential in this direction. Injection of electrons into the π-system of an [8]annulene gives rise to a topological reorganization (switch) from a folded tub-shape in the ground state, where there is little or no electronic contact across the eight-member ring, to a flat aromatic structure in the reduced state (Fig. 1)^29–34^. This shape-shifting property was first exploited in size-switchable cavities^35^. Beyond structural considerations, partially reduced COT-dimers were shown to exhibit charge transfer between [8]annulene cores^36–48^, and cross-(*pseudo*)conjugated vinyl-linked COTs with up to four units have been shown to possess intriguing redox properties^49^. However, the difficulty in developing practical *pseudo*-conjugated COT-based materials that can sterically accommodate planarization in consecutive redox cycles appears to have curbed much of the early enthusiasm for such materials.

In this work, we overturn these limitations in a design that combines a stable and reversible electromechanical responsivity of the [8]annulene cores with straightforward modular synthesis, structural rigidity, and chemical stability: oligomeric dibenzo[*a*,*e*]cyclooctatetranes (dbCOTs)^50^ connected by alkynyl spacers at *pseudo*-conjugated 5,12-positions of the [8]annulene core. With this system, the projected electromechanical switching (reduction-oxidation) of linked [8]annulenes is accomplished preparatively, and significantly, stable switching over many two-electron redox-cycles is demonstrated by spectroelectrochemical methods. In addition, electron transport through a disubstituted [8]annulene unit in a partially reduced trimer is accomplished as a critical step towards integrating such systems in junctions. The demonstrated combination of favorable electromechanical properties, ability to relay electrons across an expanded π-system, and various spectroscopic readout modes of the electronic states introduce new opportunities for implementing [8]annulenes in both responsive materials and ultimately in devices.

## Results

### Design and synthesis of *pseudo*-conjugated dbCOT-oligomers

**Fig. 2:**
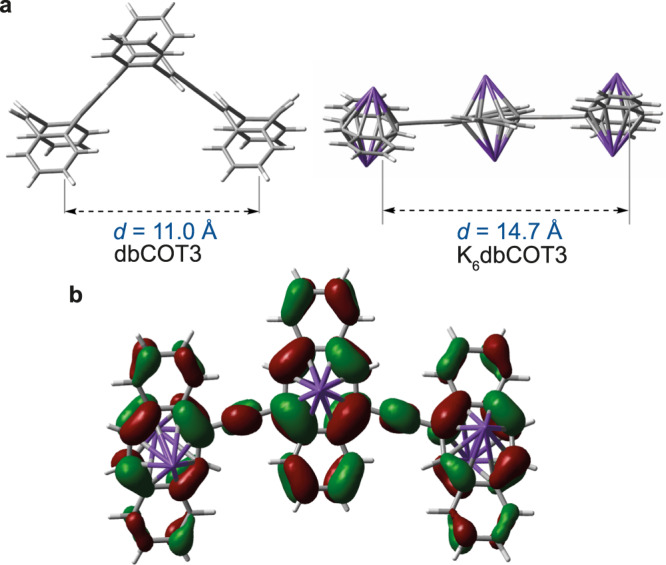
Structures of dbCOT3 and K_6_dbCOT3. **a** Geometries of dbCOT3 and K_6_dbCOT3 optimized using the B3LYP/6-311 + G(d,p) basis set and a polarizable continuum model of the THF solvent. **b** HOMO of K_6_dbCOT3. Molecular orbital plotted using a standard isovalue of 0.02. Potassium ions are shown in purple.

At the outset, we envisioned a molecular design based on dbCOT units joined by alkynyl spacers at 5,12-related positions for several reasons: (i) The carbon-carbon triple bond combines optimal orbital overlap to the annulene π-system with minimal steric demand; critical to accommodate planarization in a redox cycle; (ii) Fused benzene rings grant stability to the [8]annulene core; (iii) Linking the [8]annulenes with a 1,4 relationship is electronically attractive as these positions are *pseudo*-conjugated in the ground state and, as such, contrasts the cross-conjugation of prior related systems^49^. The viability of this design was supported by an exploratory density functional theory (DFT) investigation of a trimeric substrate dbCOT3 (Fig. 2a). Upon reduction with two electrons per dbCOT unit, the projected planarization was accommodated with only a slight distortion of the disubstituted central unit. Calculation of the molecular orbitals of this hexa-anion also show three π-levels that are fully delocalized across the molecular framework as exemplified by the highest occupied molecular orbital (HOMO) level in K_6_dbCOT3 in Fig. 2b. Concerning actuation potential, a noteworthy elongation of 34% per repeating unit (defined as the repeating unit of a hypothetical infinite chain) was found.

**Fig. 3:**
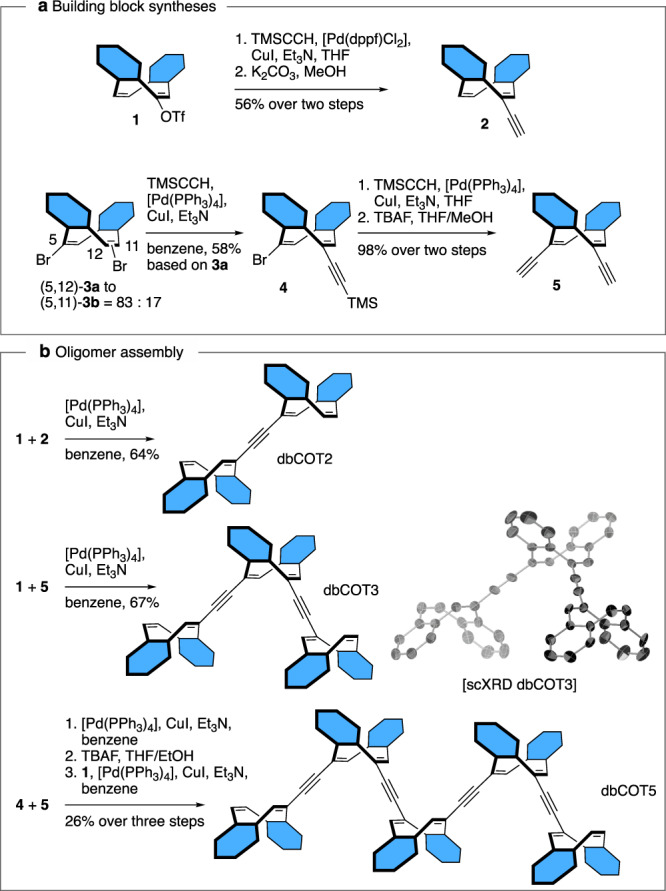
Synthesis of alkyne-linked dbCOT oligomers. **a** Synthesis of building blocks **1**, **2**, **4**, and **5**. **b** Assembly of dbCOT2, dbCOT3, and dbCOT5. The inset shows thermal ellipsoids for the single crystal XRD structure of dbCOT3 at 30% probability. Hydrogen atoms and solvate molecules are omitted for clarity.

In addition to structural simplicity, a vital design consideration was synthetic accessibility (Fig. 3). Thus, the simplest oligomer, dbCOT2, was synthesized in just three steps from the known triflate **1**:^51^ Alkynylation of **1** with trimethylsilyl-acetylene followed by a fluoride mediated deprotection of the silyl group, and finally, a Sonogashira cross-coupling of alkyne **2** and triflate **1**, gave dbCOT2 in 36% overall yield.

Oligomers with an odd number of dbCOT units were deconvoluted into bi-directional syntheses starting from di-yne **5**. Key to this approach was the finding that 5,12-dibromide **3a** was formed with an 83:17 preference for the 5,11-isomer **3b** when eliminating the corresponding 5,6,11,12-tetrabromide using 1,5-diazabicyclo[4.3.0]non-5-ene (DBN) as the base^52^. The mixture of regioisomers **3a** and **3b** was intractable at this stage, but because **3a** was found significantly less prone to undergo double alkynylation under Sonogashira conditions than **3b**, separation of the isomers was readily accomplished by a kinetic resolution during the formation of alkyne **4**. The trimeric dbCOT3 was then completed in 38% overall yield via alkynylation of **4**, de-silylation to di-yne **5**, and finally a cross-coupling of **5** with triflate **1**.

The structure of dbCOT3 was corroborated by single crystal X-ray diffraction (scXRD) analysis. The successful crystallization is noteworthy, not least, as each dbCOT unit is stereogenic by virtue of planar chirality^53,54^. As such, it has four stereoisomers, two *C*_1_ and two *C*s isomers, that rapidly interconvert at room temperature through tub-to-tub inversions. The formation of single crystals consisting of a single conformer is thus a result of a spontaneous resolution of the fluxional chirality of **4** into one of its two *meso*-forms.

To reach longer oligomers, we developed an iterative strategy wherein the desymmetrized building block **4** is joined bidirectionally to di-yne **5**. The silyl groups were then cleaved, and a cross-coupling of the resulting di-yne with triflate **1** gave dbCOT5 in just six overall steps from **3a** (15% overall yield). To our knowledge, this structure represents the longest well-defined COT-based oligomer reported. The stepwise synthetic approach is attractive as it can, in principle, be used either bi- or uni-directionally to assemble well-defined oligomers of arbitrary length.

### Chemically induced expansion/contraction cycles

**Fig. 4:**
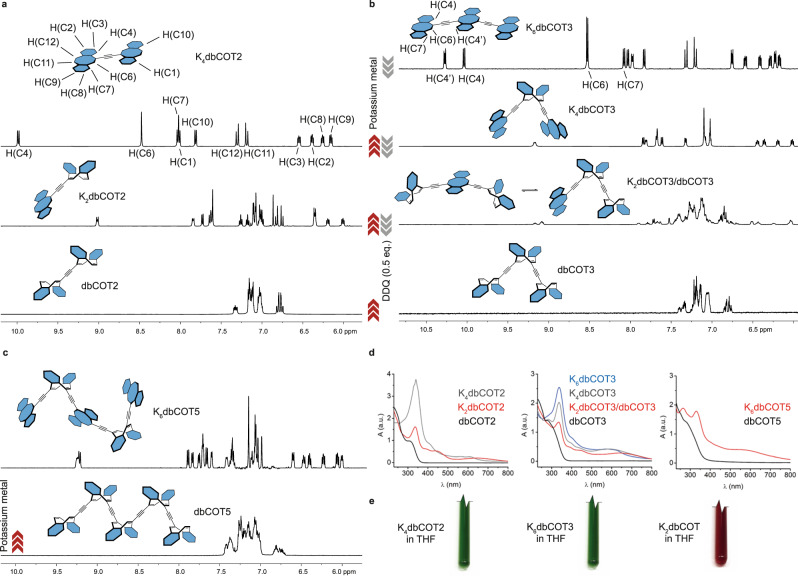
Redox behavior of dbCOT2, dbCOT3, and dbCOT5. **a**–**c** ^1^H NMR spectra in THF-*d8* of dbCOT2, dbCOT3, and dbCOT5 at various stages of reduction. The spectrum of K_2_dbCOT3 contains ~25 mol% dbCOT3. Data for the fluxional K_2_dbCOT2 and K_2_dbCOT3 were acquired at *T* = 268 K and 210 K respectively; **d** UV–VIS spectra were recorded in quartz Young’s NMR tubes. The structure at each point of reduction was confirmed by ^1^H NMR spectroscopy. **e** Images of NMR tubes (cropped) containing exhaustively reduced dbCOT2, dbCOT3, and dbCOT in THF-*d8*. For additional experimental details on K_2_dbCOT, see Supplementary Information. Potassium counterions are omitted for clarity.

To derive a detailed description of structural rearrangements in the oligomers over a redox cycle, we first employed chemical reduction and analyzed the changes by NMR spectroscopy. Gratifyingly, exhaustive reductions of dbCOT2 and dbCOT3 were readily accomplished with potassium metal using a stop-and-go method (Fig. 4a, b, see Supplementary Information for experimental details)^36^. dbCOT2 and dbCOT3 both gave a clean and complete conversion into their respective four and six electron reduced species. Each compound’s ^1^H NMR spectrum was fully assigned using a combination of 1D and 2D NMR spectroscopy methods (Supplementary Tables 4, 7). Several distinct features in the spectral data converged in support of aromatic and planar dbCOT units: The number and symmetry of peaks are consistent with a symmetry element at the center of each structure and that the dbCOT units are no longer stereogenic. Strong rotating frame NOEs (ROEs) between protons at C6 and C7 in both K_2_dbCOT2 and K_6_dbCOT3 also confirm the proximity of these atoms consistent with planarization (Supplementary Figs. 17, 18). This enhancement is absent in the tub-shaped neutral state wherein the corresponding interatomic distance is much longer. Planarity is further supported by the strong deshielding of protons at C4 and C4’ in reduced structures due to proximity to the alkyne spacers^55^. Moreover, protons on the COT rings (e.g., C6 and C11/C12 in dbCOT2) exhibit a characteristic downfield shift of 0.5–0.7 ppm from deshielding by the diatropic ring-current. Finally, the strong shielding of the C2/C3 and C8/C9 positions on the flanking benzene rings in reduced units is characteristic of a negative charge buildup^56,57^. Quantitatively, an upfield shift of 17 to 19 ppm in the ^13^C spectrum and 0.7–0.9 ppm in the ^1^H spectrum is seen for these positions in K_2_dbCOT2 compared to dbCOT2 (Supplementary Tables 2, 3).

With respect to global conformation, the alkyne linker units allow for rotational flexibility. In K_6_dbCOT3 this dynamic behavior is reflected in observed ROEs from H(C4′) to both H(C4) and H(C6) that are close to equal in intensity (Supplementary Fig. 18). DFT optimizations, however, also show that the close to co-planar conformation depicted in Fig. 2a. represents a minimum energy conformation that is favored over the corresponding orthogonal geometry (obtained by optimization with two simultaneous constraints between the dbCOT-units) by Δ*E*_tot_ = ~1.1 kcal/mol due to the expanded conjugation (Supplementary Table 11).

To verify the number of electrons accepted by each unit, a titration experiment was conducted: dbCOT was added to a solution of K_4_dbCOT2, resulting in an instantaneous two-electron transfer to give a 48:52 mixture of K_2_dbCOT2 and the known K_2_dbCOT (Supplementary Fig. 19). This result confirms that each reduced unit in the oligomer has accepted precisely two electrons^58^.

By carefully following the progression of reduction in dbCOT2 and dbCOT3 by ^1^H NMR spectroscopy, each 2n-electron reduced state could be formed selectively (Fig. 4a, b). K_2_dbCOT3 was characterized as a ~75:25 mixture with dbCOT3 since further reduction gave a mixture that also included K_4_dbCOT3. The structure of each state was confirmed by assignment of the respective ^1^H NMR spectrum (Supplementary Tables 3, 5, and 6). As shown by broadening in the room temperature NMR spectra, K_2_dbCOT3 and K_2_dbCOT2 are both fluxional structures, where the electrons are relayed between dbCOT units.

Reduction of dbCOT5 under the same conditions proved more challenging, and K_2_dbCOT5 and K_4_dbCOT5 could not be identified due to the severe broadening of the signals from rapid electron transfer between the dbCOT units. On the other hand, a well-defined hexa-anion, K_6_dbCOT5, was obtained (Fig. 4c). The fluxional chirality of each of the two non-reduced dbCOT units means that some conformers do not have a true central mirror plane/axis. However, as evident from the *pseudo*-symmetrical ^1^H NMR spectrum, charge repulsion causes an arrangement wherein every second dbCOT unit is reduced analogously to what is seen in K_4_dbCOT3. The assigned ^1^H NMR spectrum corroborate this arrangement (Supplementary Table 8). Further reduction of this compound was possible, but complete conversion into well-defined species was not achieved beyond this stage.

The UV–VIS spectra for each oligomer at the various stages of reduction are given in Fig. 4d. A substantial change of the red onset from ~350 nm into the visible region is seen upon reduction for all species consistent with an expansion of the conjugated system. There is also a linear increase in the extinction coefficient of the peak at ~340 nm with increasing reduction. Together with the fact that this peak is non-shifting, the observation aligns with an increasing number of localized chromophores contributing to the spectrum. The absence of distinct shifts to longer cut-off wavelengths for the fully reduced structures is a consequence of the conformational flexibility around the linker units. All reduced oligomers exhibit a green color in solution that contrasts the burgundy tone of the parent K_2_dbCOT. The strong red-shift of the main optical absorption is consistent with a conjugated system that expands onto the alkyne linkers (Fig. 4e).

The reduced states of dbCOT2 and dbCOT3 were then investigated by electron paramagnetic resonance (EPR) spectroscopy^59,60^. All states, as well as intermediate stages between them, were found completely EPR silent at room temperature putting an upper limit of persistent odd-electron species at ~0.1 μM (analyte concentration 1.5–5.0 mM). Singly reduced intermediates must thus undergo disproportionation to two-electron species during the experiment or rapidly accept a second electron from the metal surface during reduction^29,43^. In addition, inter- or intramolecular electron transfers within partially reduced samples occur effectively as two-electron processes on the EPR time scale at ambient temperatures.

With this clarified picture of each oligomers’ redox behavior, we finally turned to demonstrate full redox cycles by alternating reducing and oxidizing conditions. Recently such a re-oxidation was achieved for K_2_dbCOT using dry O_2_ as oxidant^61^. We found that 2,3-dichloro-5,6-dicyano-1,4-benzoquinone (DDQ) was a more suitable ^1^H NMR spectroscopy-silent four-electron oxidant that could be administered with a precise stoichiometry and without interfering with subsequent redox cycles. By performing alkali metal reduction followed by re-oxidation with DDQ in the NMR tube, complete stepwise redox cycles were achieved for both dbCOT2 and dbCOT3. With dbCOT2, three consecutive redox cycles were performed by alternating oxidation and reduction conditions in the same vessel (Supplementary Figs. 20–23).

Collectively, these experiments produce several noteworthy results. The formation of K_6_dbCOT3 exemplifies preparative formation of an exhaustively reduced [8]annulene oligomer beyond two COT units. In terms of stability, the reduced structures were found to be stable for days at room temperature without noticeable degradation and complete redox cycles could be achieved even in the challenging environment of solution chemistry. Furthermore, both the donation and acceptance of electrons in the structures were shown to be fast processes. The rate of expansion-contraction cycles of the described oligomers, or similar systems, will thus likely be limited by the electron donors and drains rather than by the intrinsic properties of the molecular framework itself.

### Consecutive electrochemically driven redox-cycles

**Fig. 5:**
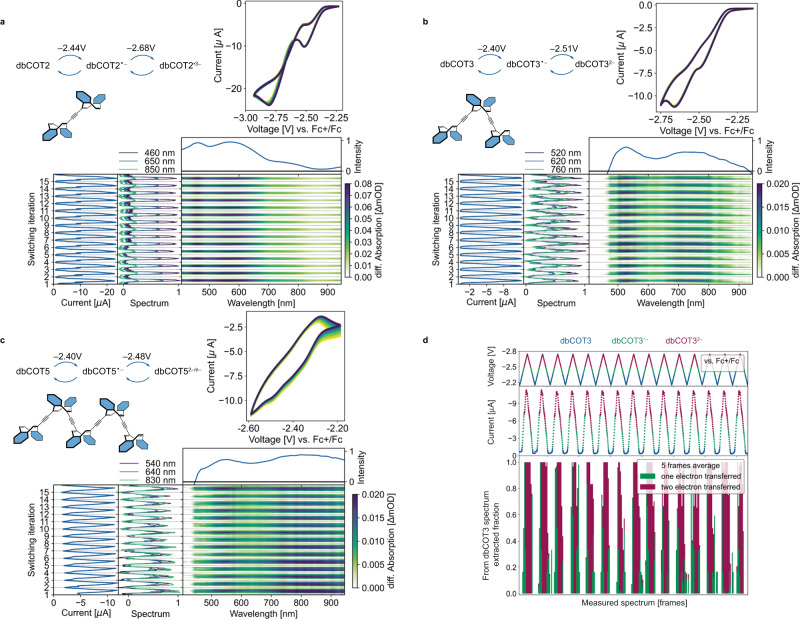
Spectroelectrochemical characterization of dbCOT2, dbCOT3, and dbCOT5. **a**–**c** Peak potentials (Ep) were recorded in separate CV experiments. Voltammograms were recorded during in situ spectroelectrochemistry measurements (15 cycles shown). Differences in peak potential reflect increased resistance due to the working electrode’s proximity to the vessel wall in spectroelectrochemistry experiments. UV–VIS spectra were recorded in reflective mode using an incandescent lamp as the light source (400–1000 nm). The heatmap shows the differential spectra relative to the ground state spectrum. The spectral trace around the maximum difference is shown above each heatmap. Spectral slices at selected wavelengths and the corresponding measured electric current during the CV are shown in the middle and left panel, respectively. Electrochemistry conditions: solvent = THF; supporting electrolyte = Bu_4_NPF_6_; working electrode = polished gold disc; reference electrode = silver wire *pseudo*-electrode. Potentials are referenced to that of ferrocene (Fc). **d** Optical deconvolution of dbCOT3, dbCOT3^•−^, and dbCOT3^2−^ during electrochemical cycling of dbCOT3. Lower panel: factorial deconvolution of the spectra in (**b**) using two species-associated spectra. Middle and top panel: measured current and applied voltage, arbitrarily colored to guide the eye with respect to state.

Essential for more advanced applications and integration of redox responsive systems is the ability to accept electrons reversibly from a surface. To evaluate this, and to derive additional structural information across a redox cycle, we conducted an in-situ spectroelectrochemical investigation of redox cycling for dbCOT2, dbCOT3, and dbCOT5 (Fig. 5).

First, the potential of the first and second reducing events for each oligomer were measured by cyclic voltammetry (CV), differential pulse voltammetry, and anodic stripping differential pulse voltammetry (Supplementary Fig. 27 and Supplementary Table 10). Integration of the CV waves of dbCOT2 showed that the second and third reductions are close in potential. In contrast, dbCOT3 accepts one electron in each of the first two waves. For dbCOT5, the data is not sufficiently resolved to determine the number of electrons accepted in each wave unambiguously. Given the multiple ways electrons can be accepted by the five dbCOT units and the fluxional nature of partly reduced species, this is not surprising. Owing to the distribution of charge over larger conjugated systems, the reducing potentials for all oligomers were found significantly lower than for dbCOT itself^29^. The difference is 0.22–0.26 V for the first wave and 0.40–0.58 V for the second wave (Fig. 5a–c and Supplementary Table 10). The small difference in potential between the first and second reducing event in the oligomers likely contributes to the absence of radical anions in EPR experiments.

Each compound’s UV–VIS spectrum was then continuously recorded in reflective mode during CV using the polished surface of the working electrode as a mirror^62–64^. Cycling the oligomers over the first two waves gave spectral traces that were consistent with chemical reversibility over many cycles, 15 shown (Fig. 5a–c). dbCOT5, and to a lesser degree dbCOT3, have a limited solubility in THF and the slight fluctuations seen in the spectral response with these compounds are related to the precipitation of scattering particles. The absorption onset in the VIS-region follows the trend observed during the chemical reduction with potassium metal but the spectra are all red-shifted by approximately ~100 nm due to differences in environment and counterions^65^.

The differential-spectra features are repetitive and stable during cycling, which suggested the possibility of also probing the states of the system by optical methods. Additional CV measurements were therefore conducted: First, the potential regions encompassing the first and second waves of dbCOT3 were cycled separately. The measurements were then modeled, and the differential spectra for the radical-anion dbCOT3^•−^ and dianion dbCOT3^2−^ extracted. When applying a 100 nm shift to the spectrum associated with dbCOT3^2−^, the spectral features match those obtained for K_2_dbCOT3, strengthening the assignment. Using the two spectra from cycling each wave individually, the measured differential spectra from cycling over both waves (shown in Fig. 5b) could be deconvoluted and expressed as fractions of the created state (Fig. 5d). See also Supplementary Fig. 29 for detailed information on the procedure. With this method, three distinct species, one ground state, and two reduced states are clearly distinguishable over a cycle showing that the state of a logical device based on such structures can also be read out optically.

### Electron relay within partially reduced dbCOT oligomers

**Fig. 6:**
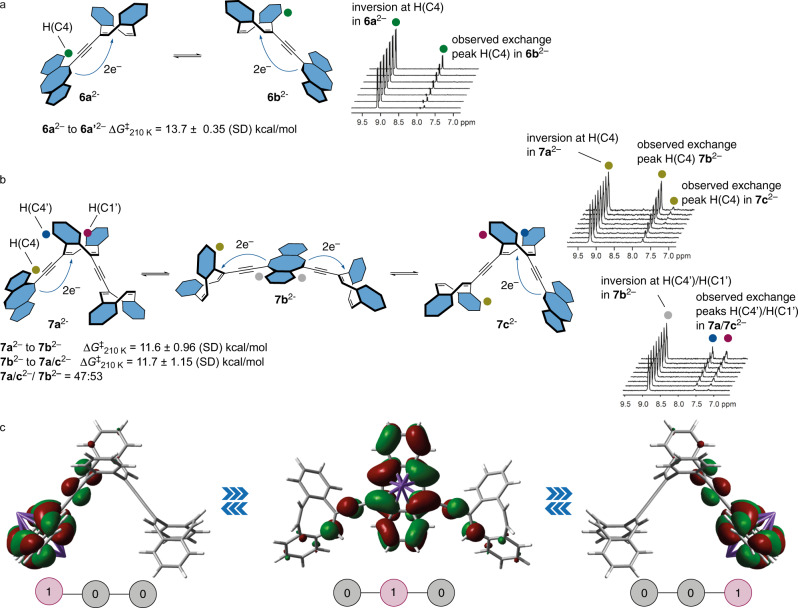
Electron relay within dimeric and trimeric structures. **a** Electron relay in K_2_dbCOT2. 1D ROE NMR spectra show inversion of H(C4) in K_2_dbCOT2 with mixing times from 25 to 200 ms. **b** Electron relay in K_2_dbCOT3. 1D ROE NMR spectra show inversion of H(C4) in **7a**^2−^ and H(C4’)/H(C1’) in **7b**^2−^ with mixing times from 25 to 200 ms. **c** DFT calculated molecular orbitals illustrating the HOMO level throughout electron relay in K_2_dbCOT3 along with a schematic representation of logical states. Optimized geometries and HOMO orbitals obtained using the B3LYP/6-311 + G(d,p) level of theory with a polarizable continuum solvent model of THF. Molecular orbitals plotted using a standard isovalue of 0.02. Potassium counterions are omitted for clarity (panels **a**, **b**).

The success of the spectroelectrochemical cycling around two-electron reduced species prompted a more detailed analysis also of the fluxional behavior of the dianions of dbCOT2 and dbCOT3 (Fig. 6a, b). In particular, we sought to derive detailed information on the possibility of electron transport through the responsive cores. This would be critical for integration and has not been previously demonstrated for related systems.

At reduced temperatures, the rearrangements were slow enough to derive the kinetic parameters by ^1^H NMR spectroscopy. The structures **6a**^2−^ and **6b**^2−^ (as well as a **7a**^2−^ and **7c**^2−^) are identical structures but individual protons at equivalent positions in the structures are not. Rearrangements can therefore be followed by chemical exchange NMR spectroscopy. Selective inversion of H(C4) in **6a**^2−^ thus gave magnetization transfer to the H(C4′) in **6b**^2−^. Extrapolation of temperature-dependent kinetic measurements (239–268 K) gave a free energy barrier of 13.7 ± 0.35 (SD) kcal/mol at 210 K for rearrangement of **6a**^2−^ to **6b**^2–^. This is in the same order as that observed for unsubstituted COTs in a vinyl-linked dimer^49^. Repetition of the experiment at different concentrations showed zero-order kinetics verifying an intramolecular mechanism for the electron redistribution (Supplementary Fig. 26). Thus, while intermolecular electron transfer is facile between dbCOTs of different reduction states (cf., the reduction of dbCOT with K_4_dbCOT2), relay of electrons between adjacent dbCOT cores occurs through an intramolecular mechanism.

The K_2_dbCOT3 system introduces additional levels of complexity due to the presence of two states that are distinct by NMR spectroscopy (**7a**^2−^ and **7b**^2−^) and a more intricate stereoisomerism. Still, activation parameters for a relay across the three cores of dbCOT3 could be derived using the same method as for K_2_dbCOT2. For electron relay to a central unit from a terminal unit, the barrier was measured to 11.6 ± 0.95 (SD) kcal/mol, and for relay from a reduced center to a terminal unit to 11.7 ± 1.15 (SD) kcal/mol. From a design perspective, it is noteworthy that planarization of the disubstituted central unit in K_2_dbCOT3 is actually more facile than the planarization of a monosubstituted terminal unit in K_2_dbCOT2. Another key observation is that selective inversion of the H(C4) proton in **7a**^2−^ gives a magnetization transfer, first to H(C4) in **7b**^2−^, and then to H(C4) in **7c**^2−^ (Fig. 6b). Combined with the demonstration that electron relay is intramolecular, this result shows that electron relay through a disubstituted [8]annulene core is indeed viable. The lower free energy barrier of relay in K_2_dbCOT3 compared to K_2_dbCOT2 is interpreted as a result of a better-stabilized transition state (TS) for the rearrangement. A plausible mechanism thus involves electronic contact between adjacent dbCOT units leading to distribution of negative charge over two alkyne linkers and their adjoining vinylene units in the TS for K_2_dbCOT3 but only one such motif for K_2_dbCOT2. The same effect also manifests in **7b**^2−^ being slightly more populated than **7a**^2−^. DFT modeling supports the assigned geometries of **7a**^2−^ and **7b**^2−^ as well as their HOMO orbitals extending onto the linker units (Fig. 6c). As expected, the non-reduced units efficiently cut conjugation along the alkynyl dimension.

Combined, the kinetic analysis of electron transfer within K_2_dbCOT2 and K_2_dbCOT3 provides a detailed description of a stepwise electron relay that interconverts the partially reduced states of dbCOT oligomers. Because intramolecular relay to and from both terminal and internal units are described, the data provide a picture of electron relay within alkynyl-linked dbCOTs that can be extrapolated to systems of arbitrary length. The successful injection of electrons from a terminal unit into a central unit and then onto a second terminal unit is intriguing as it captures, in a single molecule, the elements of an integrated switch or gate in action.

## Discussion

The potential of topologically responsive hydrocarbon materials based on [8]annulenes has long been recognized. However, practical issues, not least related to synthesis and stability, appear to have held back such advances. In this work, we have developed a molecular design that overcomes critical limitations of previous systems: 5,12-alkynyl linked dibenzo[*a*,*e*]cyclooctatetraenes. We also show that such structures can be readily assembled in a modular fashion from a small set of abundantly available components. Concerning function, stable redox-driven switching cycles are demonstrated by both spectroelectrochemical and chemical methods. The reduced states are structurally characterized in detail and provide distinct spectral responses that can be read by spectroscopic methods. In an electrochemically reduced trimeric structure, three stored states can be retrieved during cycling via deconvolution of the optical spectra representing a simple optical switch with multiple channels.

More generally, the described system was developed outgoing from a small set of straightforward design principles that should lend also to further adaptations and refinements of [8]annulene-based materials. The alkynyl linkers used to join the [8]annulene cores suggest convenient synthetic handles for attachment to surfaces and other molecular entities. We anticipate that the presented structures will inspire new entries to shape-shifting materials and provide a leap towards the intriguing possibility of implementation of [8]annulenes as single-molecule logical gates. Such work is ongoing within our laboratories and will be reported in due course.

## Supplementary information

Supplementary Information

## Data Availability

Synthetic procedures and characterization data for all new compounds including copies of NMR spectra, procedures for chemical reduction/oxidation of oligomers and dbCOT, assigned NMR spectra and structural elucidation for all reduced and partially reduced species including 1D/2D NMR spectra used in assignment, experimental details for chemical exchange NMR experiments, procedures and data for electrochemical characterization (CV, DPV, AS/DPV, and in situ spectroelectrochemistry), and details of DFT calculations and EPR experiments are available from the supplementary information file or from the authors upon request. Crystallographic data is available free of charge from the Cambridge Crystallographic Data Centre under reference number CCDC 1957125 [https://www.ccdc.cam.ac.uk/mystructures/structuredetails/f4565d47-ebe4-e911-967f-00505695f620].
