# Supplementary material for: Electro-mechanically switchable hydrocarbons based on [8]annulenes
Source: Nat Commun. 2022 Feb 14;13:860. doi: 10.1038/s41467-022-28384-8 (PMC8844043; doi:10.1038/s41467-022-28384-8)
Supplement: Supplementary file 1 — Supplementary Information [file 41467_2022_28384_MOESM1_ESM.pdf]

Supplementary Information

Electro-Mechanically Switchable [8]Annulene Oligomers

Magdalena Tasić,[1] Jakov Ivković,[1] Göran Carlström,[1] Michaela Melcher,[1] Paolo Bollella,[2] Jesper Bendix, [3] Lo Gorton,[2] Petter Persson,[4] Jens Uhlig,[5] and Daniel Strand[1,*]

[1] Centre for Analysis and Synthesis (CAS), Department of Chemistry, Lund University, Box 124, 22100 Lund, Sweden; [2] Department of Analytical Chemistry/Biochemistry, Lund University, Box 124, 221 00 Lund, Sweden, [3] Department of Chemistry, University of Copenhagen, Universitetsparken 5, 2100 Copenhagen, Denmark. [4] Division of Theoretical Chemistry, Department of Chemistry, Lund University, Box 124, 221 00 Lund, Sweden. [5] Division of Chemical Physics, Department of Chemistry, Lund University, Box 124, SE-22100 Lund, Sweden.

I.	*General methods*	*S3*
II.	*Synthetic procedures and spectroscopic data for compounds* ***2**, **3a/b**, **4**, **S1**, **5**, dbCOT2, dbCOT3, **S2**, and dbCOT5*	*S4*
III.	*Single crystal X-ray diffraction analysis of dbCOT3*	*S11*
IV.	*Reduction of dbCOT, dbCOT2, dbCOT3, and dbCOT5 with potassium metal*	*S13*
V.	*Structural and conformational assignment of dbCOT2, K_2dbCOT2, K_4dbCOT2, K_2dbCOT3, K_4dbCOT3, K_6dbCOT3, K_6dbCOT5*	*S21*
VI.	*Electron count in reduced oligomers*	*S40*
VII.	*Electron paramagnetic resonance (EPR) characterization*	*S40*
VIII.	*Redox cycles of dbCOT, dbCOT2, and dbCOT3*	*S41*
IX.	*NMR characterization of intramolecular electron transfer*	*S45*
X.	*Cyclic voltammetry, differential pulse voltammetry, and anodic stripping voltammetry characterization*	*S48*
XI.	*Spectroelectrochemical analysis*	*S52*
XII.	*Density functional theory calculations*	*S56*
XIII.	*Copies of COSY, ^{13}C-HSQC, and ^{13}C-HMBC for dbCOT2, K_2dbCOT2, K_4dbCOT2, K_2dbCOT3, K_4dbCOT3, K_6dbCOT3, and K_6dbCOT5. Excitation transfer spectra for K_2dbCOT3.*	*S91*
XIV.	*Copies of ^{1}H and ^{13}C NMR spectra*	*S114*
XV.	*Supplementary references*	*S149*

I. General methods

Unless otherwise noted, air- and moisture-sensitive reactions were carried out in oven-dried (>110 °C) glassware capped with a rubber septum under a positive pressure of argon. Air- and moisture-sensitive reagents, solvents, and solutions were transferred via syringe or stainless-steel cannula under a dry argon atmosphere. Reactions were stirred using oven-dried Teflon®-coated magnetic stir bars. Room temperature indicates a temperature in the span 20 – 25 °C. Elevated temperatures were maintained using silicone oil baths actively controlled by thermostat devices. Commercial reagents and solvents were purchased from Fisher Scientific (Acros Organics and Alfa Aesar), Sigma-Aldrich, or VWR (TCI and Apollo Scientific) with a purity of 95% or higher and used as received unless otherwise noted. Commercial catalysts were stored and handled in an argon-filled glove box. CH_2Cl_2, Et_2O, THF, toluene, *n*-hexane, and acetonitrile were obtained from a dry solvent dispensing system. Analytical thin layer chromatography (TLC) was performed on silica-backed TLC plates (60 F_{254} silica gel) and visualized by UV-light (λ = 254 nm) and/or treatment with either *p*-anisaldehyde stain, $KMnO_4$ stain, or phosphomolybdic acid (PMA) stain followed by gentle heating. Preparative column chromatography was performed according to the method of Still and co-workers[1] using silica gel 60 Å (40–60 µm particle size) columns or a Biotage automatic flash purification system. NMR spectra were recorded on a 400 MHz (^1H at 400 MHz and ^{13}C at 101 MHz) Bruker Avance II or a 500 MHz (^1H at 500 MHz and ^{13}C at 126 MHz) Bruker Avance III HD spectrometer. Unless noted otherwise, the NMR spectra were recorded at T = 298 K. Spectra were processed using Mnova v10.0. ^1H NMR chemical shifts are referenced to the residual solvent peak as internal standard ($CDCl_3$ = 7.26 ppm; THF-d_8 = 3.58 and 1.73 ppm; DMSO-d_6 = 2.50 ppm) and are listed as follows: chemical shifts (δ, ppm), multiplicity, scalar coupling constant(s) in Hz, and integral values. NMR data for planar chiral structures are given for mixtures of interconverting stereoisomers. The multiplicity of signals from these compounds are reported based on the apparent shape of the respective peaks. ^{13}C NMR chemical shifts are referenced to the residual solvent peak as internal standard ($CDCl_3$ = 77.16 ppm; THF-d_8 = 67.57 and 25.37 ppm; DMSO-d_6 = 39.62 ppm). Infrared spectra were recorded on a Bruker Alpha-P FT/IR instrument with a Diamond ATR sensor and are reported as follows: wavenumbers (cm^{-1}), description (w = weak, m = medium, s = strong, br = broad). Samples were prepared as a film for liquid or neat for solid substances. UV-VIS spectra for reduced oligomers were obtained by placing quartz Young's tube with samples directly in an Agilent Technologies Cary 60 UV-

VIS spectrophotometer. The homogeneity of the samples was confirmed by ^{1}H NMR spectroscopy prior to collection of UV-VIS data. Melting points were measured on a BÜCHI B-540 melting point apparatus and are uncorrected. High-resolution mass spectra (HRMS) were recorded on a Q-TOF, a LTQ Velos PRO Orbitrap ESI spectrometer, or a JEOL GCmate II spectrometer by direct probe using EI-ionization (70 eV).

*II. Synthetic procedures and spectroscopic data for compounds **2**, **3a/b**, **4**, **S1**, **5**, dbCOT2, dbCOT3, **S2**, and dbCOT5*

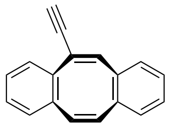

2

(5*Z*,11*Z*)-5-Ethynyldibenzo[*a,e*]cyclooctatetraene (2). To a stirred solution of triflate **1**[2] (0.63 g, 1.5 mmol), CuI (43 mg, 0.23 mmol), Et_3N (0.52 mL, 3.7 mmol), and ethynyltrimethylsilane (0.30 mL, 2.1 mmol) in THF (15 mL) was added Pd(dppf)$Cl_2 \cdot CH_2Cl_2$ (61 mg, 0.075 mmol) in one portion. After stirring at 50 °C overnight, the mixture was concentrated under reduced pressure, the residues re-dissolved in MeOH (15 mL), and then stirred. K_2CO_3 (0.30 g, 2.2 mmol) was added in one portion. After 2.5 h, the mixture was dry loaded onto silica and purified by flash chromatography (eluting with 17% CH_2Cl_2 in heptane) to afford alkyne **2**.

Yield: 0.19 g (56%), isolated as an orange oil, >95% pure by ^{1}H NMR spectroscopy and a single spot by TLC.

R*f*: 0.42 (16:1 EtOAc/heptane).

^{1}H NMR (400 MHz, $CDCl_3$) δ: 7.39 – 7.34 (m, 1H), 7.28 (s, 1H), 7.23 – 7.14 (m, 4H), 7.10 – 7.02 (m, 3H), 6.82 (d, J = 11.8 Hz, 1H), 6.77 (d, J = 11.8 Hz, 1H), 2.98 (s, 1H) ppm.

^{13}C NMR (101 MHz, $CDCl_3$) δ: 140.5, 137.1, 136.91, 136.89, 136.0, 133.2, 133.0, 129.3, 129.0, 128.8, 128.7, 127.8, 127.6, 127.3, 127.0, 125.9, 86.0, 76.2 ppm.

FTIR (CH_2Cl_2 film): 3287 (m), 3013 (w), 1491 (m), 1413 (m), 1211 (s), 600 (s) cm^{-1}.

HRMS-ESI (m/z): $[M + H]^+$ Calcd for $C_{18}H_{13}$ 229.1012; Found: 229.1018.

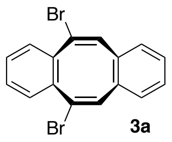
3a

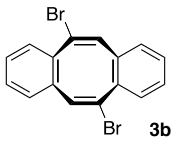
3b

(5*E*,11*E*)-5,12-Dibromodibenzo[*a,e*]cyclooctatetraene (3a) and (5*E*,11*E*)-5,11-dibromodibenzo[*a,e*]cyclooctatetraene (3b). To a stirred solution of 5,6,11,12-tetrabromo-5,6,11,12-tetrahydrodibenzo[*a,e*][8]annulene (5.6 g, 11 mmol) in toluene (196 mL) heated to 120 °C was added 1,5-diazabicyclo[4.3.0]non-5-ene (7.0 mL, 56 mmol) dropwise via syringe. After 2 h, TLC indicated full conversion of the starting material. The reaction mixture was then cooled to room temperature, washed with 10% H_2SO_4 (150 mL) and then with water (150 mL). The organic layer was separated, passed through a phase separator, and concentrated under reduced pressure to furnish mixture of regioisomers (**3a** to **3b** = 83:17 (^{13}C NMR spectroscopy)) that was not readily separable by column chromatography.

Yield: 3.8 g (98%). Isolated as an off-white solid, >95% pure by ^{1}H NMR. Single spot by TLC ($KMnO_4$ stain).

R*f*: 0.71 (10:1 hexane/EtOAc).

^{1}H NMR (400 MHz, $CDCl_3$) δ: 7.41 – 7.35 (m, 2H), 7.34 – 7.30 (m, 2H), 7.26 – 7.15 (m, 4H), 7.10 – 7.03 (m, 2H) ppm.

^{13}C NMR (101 MHz, $CDCl_3$) δ: major regioisomer **3a**: 137.5, 136.1, 134.9, 129.5, 128.8, 128.4, 127.7, 122.1 ppm; minor regioisomer **3b**: 138.1, 135.8, 133.9, 130.3, 128.7, 127.8, 127.8, 123.6 ppm.

FTIR (CH_2Cl_2 film): 3061 (w), 3021 (w), 1636 (m br), 1485 (m), 1430 (w), 1335 (w), 1164 (m br), 928 (m), 876 (s), 837 (m), 758 (s), 746 (s), 685 (s), 600 (m), 576 (m), 460 (w) cm^{-1}.

HRMS-APCI (m/z): $[M + NH_4]^+$ Calcd for $C_{16}H_{14}Br_2N$ 377.9493; Found: 377.9489.

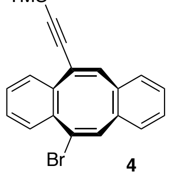
4

(((5*E*,11*E*)-12-Bromodibenzo[*a,e*][8]annulen-5-yl)ethynyl)trimethylsilane (4). To a stirred solution of a 83 : 17 mixture of dibromides **3a** and **3b** (98 mg, 0.27 mmol), [Pd(PPh_3)$_4$] (6.0

mg, 5.2 μmol), CuI (4.0 mg, 21 μmol) and Et_3N (0.11 mL, 0.79 mmol) in benzene (2.7 mL) was added ethynyltrimethylsilane (81 μL, 0.59 mmol) in one portion, and the resulting mixture heated to 50 °C. After 90 min, TLC indicated complete conversion of the starting material. The reaction was then quenched by addition of ethylenediamine (20 μL), dry-loaded onto silica, and purified by flash chromatography (100% heptane) to afford alkyne **4**.

Yield: 46 mg (58% based on **3a**). Isolated as a pale-yellow oil, >95% pure in ^{1}H NMR. A single spot by TLC.

R*f*: 0.26 (heptane).

^{1}H NMR (500 MHz, $CDCl_3$) δ: 7.40 – 7.36 (m, 1H), 7.34 – 7.31 (m, 1H), 7.27 – 7.25 (m, 2H; 2 singlets), 7.25 – 7.22 (m, 2H), 7.18 – 7.14 (m, 2H), 7.09 – 7.03 (m, 2H), 0.23 (s, 9H) ppm.

^{13}C NMR (126 MHz, $CDCl_3$) δ: 139.8, 138.1, 136.4, 136.3, 135.9, 134.6, 129.8, 128.9, 128.8, 128.7, 128.3, 128.0, 127.6, 127.5, 126.5, 122.7, 106.3, 94.3, 0.1 ppm.

FTIR (CH_2Cl_2 film): 2959 (w), 2144 (w), 1487 (w), 1250 (w), 1061 (w), 917 (w), 840 (s), 759 (m), 691 (w) cm^{-1}.

HRMS-ESI (m/z): $[M + H]^+$ Calcd for $C_{21}H_{20}BrSi$ 379.0518; Found: 379.0515.

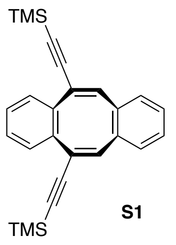

S1

(5*E*,11*E*)-5,12-Bis((trimethylsilyl)ethynyl)dibenzo[*a,e*][8]annulene (S1). To a stirred solution of bromide **4** (0.43 g, 1.1 mmol), $[Pd(PPh_3)_4]$ (66 mg, 57 μmol), CuI (11 mg, 58 μmol), and Et_3N (1.3 mL, 9.3 mmol) in dry THF (25 mL) was added ethynyltrimethylsilane (0.35 mL, 2.5 mmol) in one portion. The resulting mixture was heated to 50 °C. After 20 h, TLC indicated complete conversion of the starting material. The reaction was then quenched by addition of ethylenediamine (0.20 mL). The resulting mixture was dry-loaded onto celite, and purified by an automated flash purification system (1% ethyl acetate in heptane) to afford di-yne **S1**.

Yield: 0.46 g, quant. Isolated as a colorless oil, >95% pure in ^{1}H NMR and a single spot by TLC.

R$_f$: 0.37 (10:1 heptane/CH_2Cl_2).

^{1}H NMR (400 MHz, $CDCl_3$) δ: 7.36 – 7.31 (m, 2H), 7.25 – 7.20 (m, 4H), 7.17 – 7.12 (m, 2H), 7.07 – 7.02 (m, 2H), 0.22 (s, 18H) ppm.

^{13}C NMR (101 MHz, $CDCl_3$) δ: 139.7, 136.7, 136.2, 129.2, 128.6, 127.9, 127.5, 126.9, 106.8, 93.8, 0.1 ppm.

FTIR (CH_2Cl_2 film): 2959 (w), 2141 (w), 1492 (w), 1249 (w), 1083 (w), 1050 (w), 905 (w), 842 (s), 757 (m) cm^{-1}.

HRMS-ESI (m/z): $[M + H]^+$ Calcd for $C_{26}H_{29}Si_2$ 397.1808; Found: 397.1797.

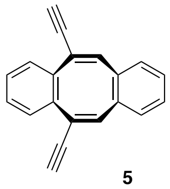

5

(5*Z*,11*Z*)-5,12-Diethynyldibenzo[*a,e*]cyclooctatetraene (5). To a stirred solution of silyl protected di-yne **S1** (0.15 g, 0.37 mmol) in THF/MeOH 3:1 (16 mL), was added TBAF (1.8 mL, 1.8 mmol, 1.0 M in THF) dropwise over 30 seconds and the resulting mixture was heated to 35 °C. After stirring for 2.5 h, TLC indicated complete conversion of the starting material. The reaction mixture was evaporated, and the remaining residue dry-loaded onto celite. Purification by an automated flash purification system (10% to 15% toluene in heptane) afforded di-yne **5**.

Yield: 87 mg (98%). Isolated as a pale-yellow oil, >95% pure by ^{1}H NMR spectroscopy and a single spot by TLC.

R$_f$: 0.17 (10:1 heptane/CH_2Cl_2).

^{1}H NMR (400 MHz, $CDCl_3$): 7.38 – 7.31 (m, 2H), 7.28 – 7.26 (m, 2H), 7.25 – 7.22 (m, 2H), 7.20 – 7.16 (m, 2H), 7.10 – 7.04 (m, 2H), 3.03 (s, 2H) ppm.

^{13}C NMR (101 MHz, $CDCl_3$) δ: 140.1, 136.5, 135.9, 129.0, 128.6, 128.1, 127.6, 125.7, 85.4, 77.0 ppm.

FTIR (CH_2Cl_2 film): 3284 (s), 3059 (w), 3018 (w), 1487 (w), 1430 (w), 1244 (w br), 1027 (w), 869 (m), 759 (m), 652 (m), 622 (m), 605 (m) cm^{-1}.

HRMS-ESI (m/z): $[M + H]^+$ Calcd for $C_{20}H_{13}$ 253.1017; Found: 253.1019.

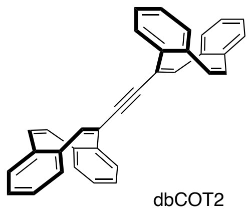

dbCOT2

dbCOT2: 1,2-Bis((5*Z*,11*Z*)-dibenzo[*a,e*]cyclooctatetraen-5-yl)ethyne. Ethynyl-dbCOT **2** (0.11 g, 0.50 mmol), triflate **1** (0.18 g, 0.50 mmol), [Pd(PPh_3)$_4$] (29 mg, 25 µmol), CuI (10 mg, 53 µmol) and Et_3N (0.21 mL, 1.5 mmol) were charged in a round bottom flask and benzene (5.0 mL) was added. The resulting mixture was stirred at 50 °C overnight, then cooled down to room temperature, and concentrated under reduced pressure. The remaining crude mixture was dry-loaded onto silica and purified by flash chromatography (25% CH_2Cl_2 in heptane) to afford dbCOT2.

Yield: 0.14 g (64%). Isolated as an amorphous pale-yellow solid, >95% pure in ^{1}H NMR and a single spot by TLC.

R*f*: 0.46 (7:1 EtOAc/heptane).

^{1}H NMR (500 MHz, $CDCl_3$) δ: 7.41 – 7.35 (m, 2H), 7.22 – 7.15 (m, 10H), 7.07 – 7.05 (m, 6H), 6.85 – 6.76 (m, 4H) ppm.

^{13}C NMR (126 MHz, $CDCl_3$) δ: 139.2, 139.0, 138.5, 137.4, 137.3, 137.12, 137.10, 136.8, 136.4, 133.2, 133.06, 133.04, 129.57, 129.54, 129.0, 128.84, 128.82, 128.7, 127.57, 127.4, 127.20, 127.18, 127.03, 126.99, 90.8, 90.7 ppm.

FTIR ($CDCl_3$ film): 3057 (m), 3012 (m), 1490 (s), 1427 (w), 1363 (w), 1213 (w), 908 (s), 860 (m), 771 (s), 752 (s), 733 (s), 600 (w), 516 (w), 454 (w) cm^{-1}.

HRMS-APCI (m/z): [M + H]$^+$ Calcd for $C_{34}H_{23}$ 431.1800; Found: 431.1797.

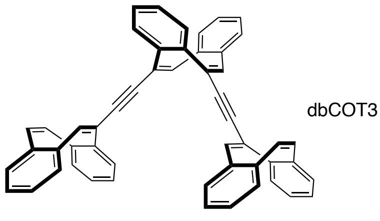

dbCOT3

dbCOT3: (5*Z*,11*Z*)-5,12-Bis(((5*Z*,11*Z*)-dibenzo[*a,e*]cyclooctatetraen-5-yl)ethynyl) dibenzo[*a,e*]cyclooctatetraene. Di-yne **5** (18 mg, 0.071 mmol), triflate **1** (54 mg, 0.15 mmol),

[$Pd(PPh_3)_4$] (4.0 mg, 3.5 μmol), CuI (1.0 mg, 5.3 μmol) and Et_3N (39 μL, 0.28 mmol) were dissolved in benzene (0.70 mL) and stirred at 50 °C for 6 hours after which TLC indicated complete conversion of **5**. The reaction mixture was then cooled to room temperature, dry-loaded onto silica, and purified by flash chromatography (14% to 25% benzene in heptane) to afford dbCOT3.

Yield: 31 mg (67%). Isolated as a pale-yellow solid, >95% pure in ^{1}H NMR and a single spot by TLC.

R_f: 0.48 (1:1 heptane/benzene).

^{1}H NMR (400 MHz, $CDCl_3$) δ: 7.48–7.31 (m, 4H), 7.29–7.12 (m, 16H), 7.10-7.00 (m, 8H), 6.89–6.73 (m, 4H) ppm.

^{13}C NMR (101 MHz, $CDCl_3$) δ: 139.4, 139.2, 138.96, 138.8, 137.4, 137.3, 137.14, 137,12, 136.9, 136.42, 136.37, 133.2, 133.1, 129.6, 129.2, 128.94, 128.87, 128.84, 128.74, 127.72, 127.89, 127.86, 127.6, 127.42, 127.38, 127.25, 127.22, 127.0, 126.86, 126.85, 91.5, 90.5 ppm.

FTIR (CH_2Cl_2 film): 3056 (w), 3013 (w), 1489 (m), 1428 (w), 1362 (w), 1264 (w), 859 (m), 754 (s) cm^{-1}.

HRMS-APCI (m/z): $[M + H]^+$ Calcd for $C_{52}H_{33}$ 657.2582; Found: 657.2566.

mp: 140 °C (decomposition).

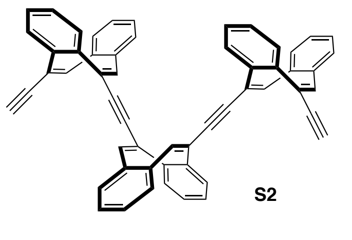

S2

(5*Z*,11*Z*)-5,12-Bis(((5*Z*,11*Z*)-12-ethynyldibenzo[*a,e*]cyclooctatetraen-5-yl)ethynyl) dibenzo[*a,e*]cyclooctatetraene (**S2**). A solution of bromide **4** (61 mg, 0.16 mmol), di-yne **5** (19 mg, 0.075 mmol), [$Pd(PPh_3)_4$] (4.0 mg, 3.5 μmol), CuI (1.0 mg, 5.3 μmol) and Et_3N (43 μL, 0.31 mmol) were dissolved in benzene (0.50 mL) and stirred at 50 °C for 18 h. The mixture was then cooled to room temperature, dry-loaded onto silica, and passed through a short column of silica (eluting with heptane/benzene 3:1). Fractions containing the major product (R_f = 0.35, heptane/benzene 2:1) were pooled and concentrated to yield 28 mg of an amorphous off-white oil. The crude product was re-dissolved in EtOH/THF 1:1 (1.0 mL). To this mixture was

added TBAF (0.16 mL, 0.16 mmol, 1.0 M in THF) dropwise. The resulting solution was heated to 40 °C and stirred overnight. The mixture was dry-loaded onto silica and purified by flash chromatography (eluting with 50% benzene in pentane) to afford di-yne **S2**.

Yield: 24 mg (44%). Isolated as a white amorphous solid, >95% pure in ^{1}H NMR spectroscopy and a single spot by TLC.

R*f*: 0.45 (1:1 heptane/benzene).

^{1}H NMR (500 MHz, $CDCl_3$) δ: 7.45 – 7.32 (m, 6H), 7.30 – 7.12 (m, 18H), 7.10 – 7.02 (m, 6H), 3.07 – 2.97 (m, 2H) ppm.

^{13}C NMR (126 MHz, $CDCl_3$) δ: 140.29, 140.27, 140.24, 139.24, 139.22, 139.06, 139.01, 138.9, 138.82, 138.80, 137.03, 137.00, 136.89, 136.86, 136.83, 136.41, 136.39, 136.37, 136.34, 136.32, 136.30, 135.99, 135.95, 129.39, 129.37, 129.27, 129.11, 129.0, 128.77, 128.74, 128.72, 128.69, 128.5, 128.12, 128.07, 128.0, 127.94, 127.90, 127.6, 127.5, 126.79, 126.77, 125.8, 91.1, 90.9, 85.59, 85.57, 77.1, 76.9 ppm.

FTIR (CH_2Cl_2 film): 3287 (m), 3058 (w), 3017 (w), 1487 (m), 1429 (w), 1360 (m), 1287 (w), 908 (m), 866 (m), 756 (s), 732 (s), 697 (w), 650 (m), 603 (m) cm^{-1}.

HRMS-ESI (m/z): $[M + H]^{+}$ Calcd for $C_{56}H_{33}$ 705.2582; Found: 705.2579.

mp: 125 °C (decomposition).

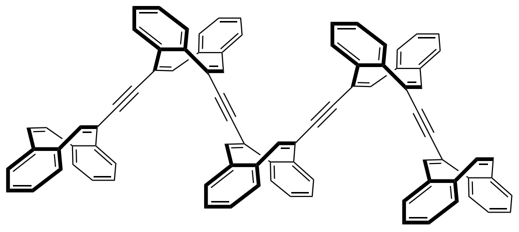

dbCOT5

dbCOT5: (5*Z*,11*Z*)-5,12-Bis(((5*Z*,11*Z*)-12-(((5*Z*,11*Z*)-dibenzo[*a,e*]cyclooctatetraen-5-yl)ethynyl)dibenzo[*a,e*]cyclooctatetraen-5-yl)ethynyl)dibenzo[*a,e*]cyclooctatetraene. Triflate **1** (16 mg, 46 μmol), di-yne **S2** (16 mg, 23 μmol), $[Pd(PPh_3)_4]$ (1.0 mg, 0.86 μmol), CuI (1.0 mg, 5.3 μmol) and Et_3N (12 μL, 86 μmol) were dissolved in benzene (0.70 mL) and stirred at 50 °C. After 60 min, the reaction showed complete consumption of starting materials (^{1}H NMR spectroscopy of an aliquot of the reaction mixture). The mixture was immediately cooled

to room temperature, dry-loaded onto silica, and purified via flash chromatography (eluting with 33% benzene in heptane) to afford dbCOT5.

Yield: 14 mg (58%). Isolated as a white solid, >95% pure by ^{1}H NMR spectroscopy and a single spot by TLC.

R*f*: 0.27 (2:1 heptane/benzene).

^{1}H NMR (500 MHz, $CDCl_3$) δ: 7.48 – 7.32 (m, 8H), 7.26 – 6.97 (m, 40H), 6.87 – 6.70 (m, 4H) ppm.

^{13}C NMR (126 MHz, $CDCl_3$) δ: 139.40, 139.36, 139.27, 139.22, 139.19, 139.12, 139.10, 139.06, 139.04, 139.01, 138.98, 138.92, 138.89, 138.87, 138.82, 137.4, 137.3, 137.0, 136.9, 136.88, 136.86, 136.38, 136.36, 136.33, 136.2, 133.08, 133.07, 129.6, 129.38, 129.34, 129.31, 129.25, 128.88, 128.84, 128.7, 128.4, 128.0, 127.9, 127.69, 127.64, 127.58, 127.57, 127.4, 127.3, 127.24, 127.22, 127.02, 126.97, 126.87, 126.86, 126.84, 126.81, 91.56, 91.52, 91.47, 91.43, 91.2, 91.11, 91.09, 91.04, 91.01, 90.5, 90.4 ppm.

FTIR (CH_2Cl_2 film): 3058 (w), 3014 (w), 2955 (w), 2924 (w), 1721 (w), 1488 (m), 1428 (w), 1362 (w), 1286 (w), 906 (m), 864 (m br), 756 (s), 731 (s), 600 (w) cm^{-1}.

HRMS-APCI (m/z): $[M + NH_4]^+$ Calcd for $C_{88}H_{56}N$ 1126.4413; Found: 1126.4418.

mp: 130 °C (decomposition).

III. Single crystal X-ray diffraction analysis of dbCOT3

A sample of dbCOT3 in THF was gently warmed until all solids were completely dissolved. The resulting solution was stored in a sealed vial at room temperature for two weeks during which time crystals formed. Crystals were cut to size and mounted under a protective layer of parathon oil. Datasets were collected for several crystals from different batches but all gave very weak diffraction data (Supplementary Table 1, Supplementary Figure 1). Elevated R-values reflects poor crystal quality.

Supplementary Table 1. Crystal data for dbCOT3 [a]

Chemical formula	C_{52} H_{32}, $3(C_4H_8O)$	
Formula Mass	873.09	
Crystal size /mm^3	0.1 x 0.05 x 0.05	
Crystal habit	colorless, rod	
Crystal system	monoclinic	
Unit cell dimensions:	a = 8.6794 (19) Å	α = 90 °
	b = 34.059 (7) Å	β = 109.96 (3) °
	c = 9.0862 (18) Å	γ = 90 °
Unit cell volume /Å^3	2524.6 (10)	
Collection temperature/K	110 (2)	
Space group	*P* 21/m	
Number of formula units per cell, *Z*	*2*	
Radiation type	MoKα	
Absorption coefficient, μ/mm^{-1}	0.069	
No. reflections collected / unique	12705 / 5899 [$R_{(int)}$ = 0.1790]	
Final R_1 values $(I > 2\sigma(I))$	R_1 = 0.1332, wR_2 = 0.1474	
Final R_1 values *(all data)*	R_1 = 0.4775, wR_2 = 0.2014	
Final $wR(F^2)$ values $(I > 2\sigma(I)$	0.201	
Goodness of fit on F^2	1.095	
Largest diff. peak and hole /e-/Å^3	0.309 and –0.422	
CCDC	1957125	

[a] Both independent solvent molecules were modeled isotropically to obtain a stable refinement. Removal of the electron density originating from the THFs with SQUEEZE gave an unstable refinement.

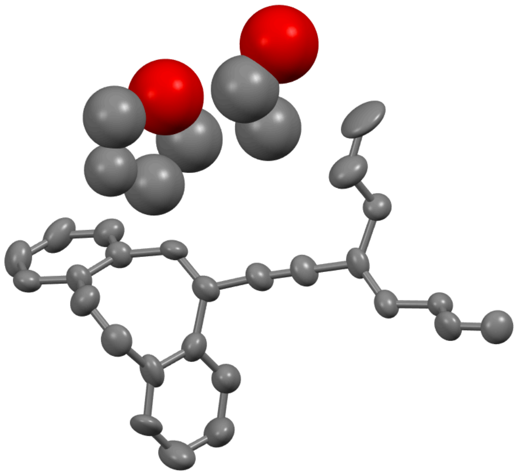

Supplementary Figure 1. The asymmetric unit of dbCOT3. Thermal ellipsoids are shown at 30% probability. Gray = carbon atom; red = oxygen atom. Hydrogen atoms are omitted for clarity.

IV. Reduction of dbCOT, dbCOT2, dbCOT3, and dbCOT5 with potassium metal

General procedure. A sample of dbCOT or the respective oligomer was charged in an oven-dried J. Young NMR tube in an argon filled glovebox. A small piece of freshly cut potassium metal (excess) was adhesively deposited by pressing into a thin film on the glass surface of the broad top chamber of the tube (Supplementary Figure 2). Dry and oxygen-free THF-d_8 was introduced via a gas-tight syringe. The tube was then sealed, exported out of the glovebox, and sonicated in an upright position until the oligomer was fully dissolved (clear colorless solution).

Reduction was initiated by turning the tube upside down, thus exposing the liquid to the metal surface, followed by mild shaking. A color-change was indicative of the reduction proceeding. Returning the tube to an upright position ceased reduction immediately, and the samples could be stored for days without noticeable decomposition or further reduction. The degree of reduction was monitored by ^{1}H NMR spectroscopy. Unless otherwise noted, NMR spectra were obtained at ambient temperature.

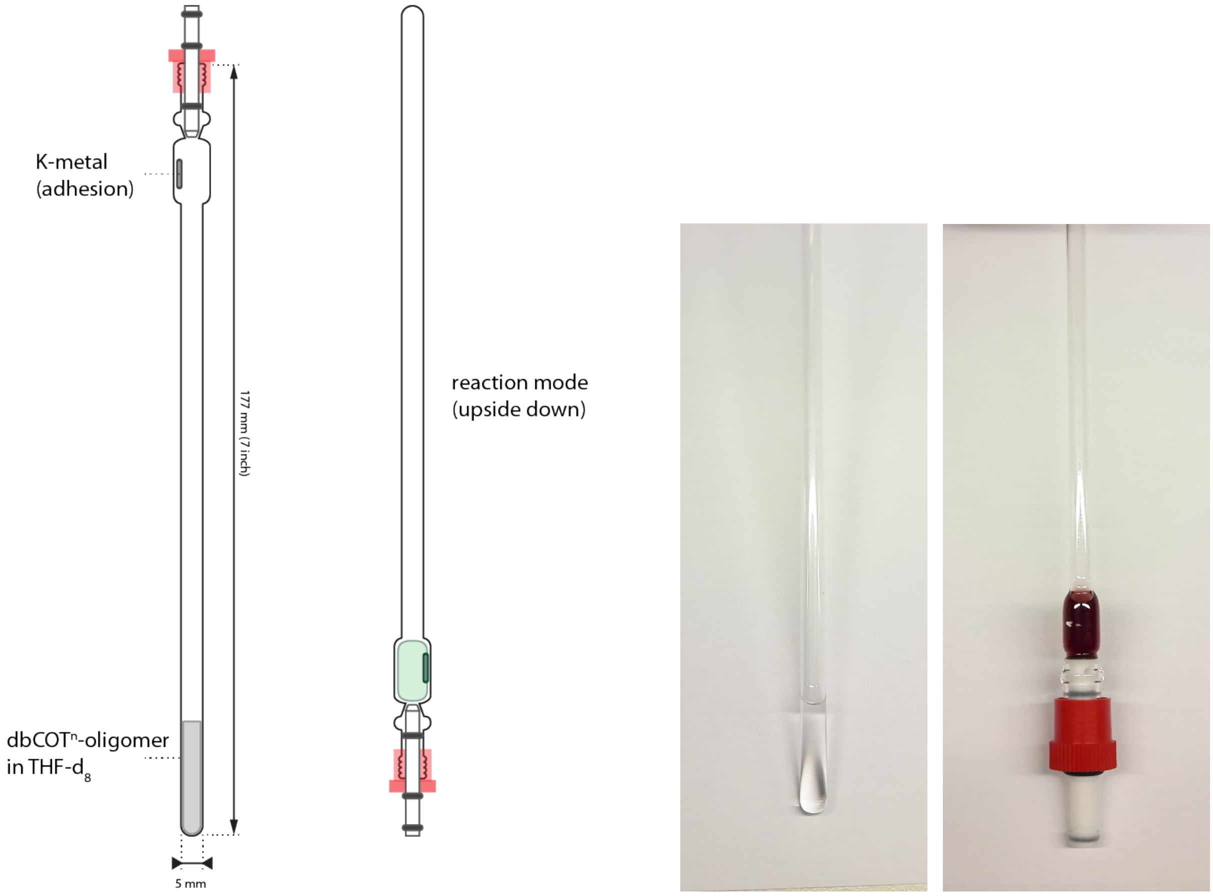

Supplementary Figure 2. Left: Schematic representation of the experiment setup; Right: Images of a typical experiment (reduction of dbCOT) showing the tube in an upright and upside-down position.

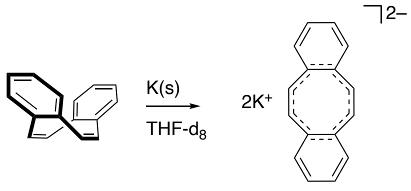

K_2dbCOT

Reduction of dbCOT to K_2dbCOT.[3] Following the general procedure, dbCOT (3.1 mg, 15 μmol) was dissolved in THF-d_8 (0.50 mL). Upon exposure of the resulting colorless solution to potassium metal (excess) the mixture gradually turned deep burgundy. The reaction was analyzed at regular intervals by ^{1}H NMR until a clean conversion into K_2dbCOT was achieved.

^{1}H NMR (500 MHz, THF-d_8) δ: 7.87 (dd, J = 6.6, 3.4 Hz, 4H), 7.17 (s, 4H), 6.19 (dd, J = 6.7, 3.3 Hz, 4H) ppm.

^{13}C NMR (126 MHz, THF-d_8) δ: 135.7, 109.4, 108.9, 95.8 ppm.

UV-VIS (λ-max in THF-d_8): 595, 550, 510, 470, 395, 335, 265, 245 nm.

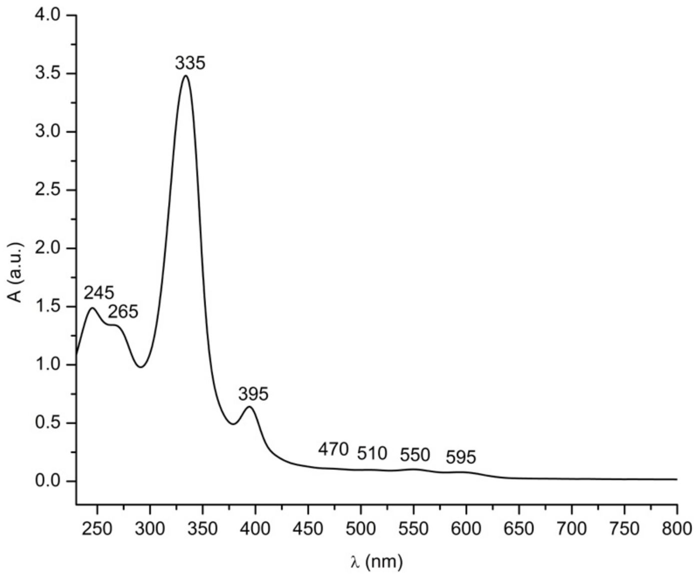

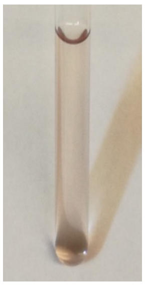

Supplementary Figure 3. Left: UV-VIS spectrum of K_2dbCOT; Right: Image of a dilute solution of K_2dbCOT in THF-d_8.

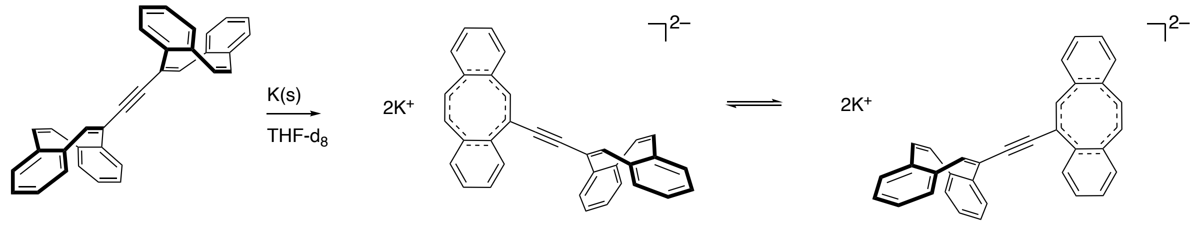

K$_2$dbCOT2 (**6a/b**)

Reduction of dbCOT2 to K$_2$dbCOT2. Following the general procedure, dbCOT2 (0.58 mg, 1.3 µmol) was dissolved in THF-d$_8$ (0.50 mL). Upon exposure of the resulting colorless solution to potassium metal (excess) the mixture gradually turned deep green. The reaction was analyzed at regular intervals by ^{1}H NMR spectroscopy at 237.2 K until a clean formation of K$_2$dbCOT2 was achieved.

^{1}H NMR (500 MHz, THF-d$_8$, at T = 237.2 K) δ: 9.09 – 9.00 (m, 1H), 7.91 – 7.83 (m, 1H), 7.76 (d, J = 7.8 Hz, 1H), 7.71 – 7.58 (m, 3H), 7.33 – 7.25 (m, 1H), 7.24 – 7.18 (m, 1H), 7.17 – 7.09 (m, 4H), 7.08 – 7.04 (m, 2H), 7.02 (d, J = 7.7 Hz, 1H), 6.89 (s, 1H), 6.85 (d, J = 11.7 Hz, 1H), 6.78 (d, J = 11.7 Hz, 1H), 6.43 – 6.34 (m, 2H), 6.22 (dd, J = 8.4, 5.8 Hz, 1H), 6.04 (dd, J = 8.3, 5.8 Hz, 1H) ppm.

^{13}C NMR (126 MHz, THF-d$_8$, at T = 237.2 K) δ: 140.6, 138.7, 137.0, 136.3, 135.4 (2 signals overlapping), 134.3, 133.2, 133.1, 131.3, 130.6, 130.0, 129.1, 128.5, 128.1, 126.3, 126.2, 126.1, 125.3, 125.1, 112.3 (2 signals overlapping), 112.2, 111.9, 111.6, 110.3, 110.1, 106.6, 106.5, 102.5, 98.1, 97.2, 90.8, 82.0 ppm.

UV-VIS (λ-max in THF-d$_8$): 645, 435, 380, 335 nm.

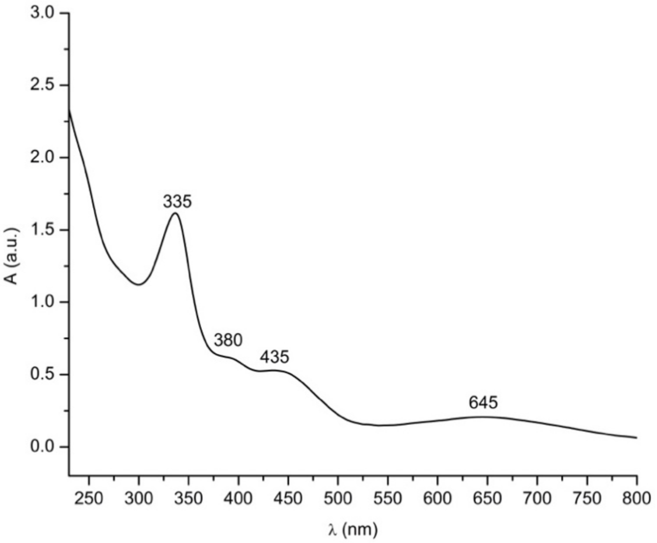

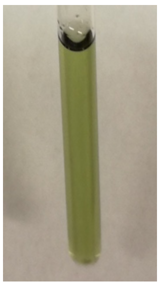

Supplementary Figure 4. Left: UV-VIS spectrum of K_2dbCOT2; Right: Image of a dilute solution of K_2dbCOT2 in THF-d_8.

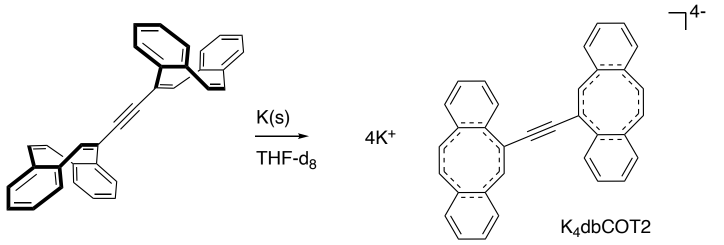

Reduction of dbCOT2 to K_4dbCOT2. Reduction of K_2dbCOT2 was continued following the general procedure. The reaction was analyzed at regular intervals by ^{1}H NMR spectroscopy until a clean formation of K_4dbCOT2 (deep green) was achieved.

^{1}H NMR (500 MHz, THF-d_8) δ: 9.99 (d, J = 9.0 Hz, 2H), 8.48 (s, 2H), 8.08 – 7.97 (m, 4H), 7.81 (d, J = 8.4 Hz, 2H), 7.30 (d, J = 13.0 Hz, 2H), 7.19 (d, J = 13.0 Hz, 2H), 6.55 (dd, J = 8.7, 5.7 Hz, 2H), 6.39 (dd, J = 8.3, 5.8 Hz, 2H), 6.26 (dd, J = 8.4, 5.8 Hz, 2H), 6.16 (dd, J = 8.3, 5.8 Hz, 2H) ppm.

^{13}C NMR (126 MHz, THF-d_8) δ: 136.7, 136.4, 135.5, 134.2, 111.3, 111.2, 110.5, 110.3, 110.0, 109.5, 108.8, 108.3, 101.5, 100.3, 98.8, 97.4, 89.4 ppm.

UV-VIS (λ-max in THF-d_8): 600, 555, 490, 400, 340, 275 nm.

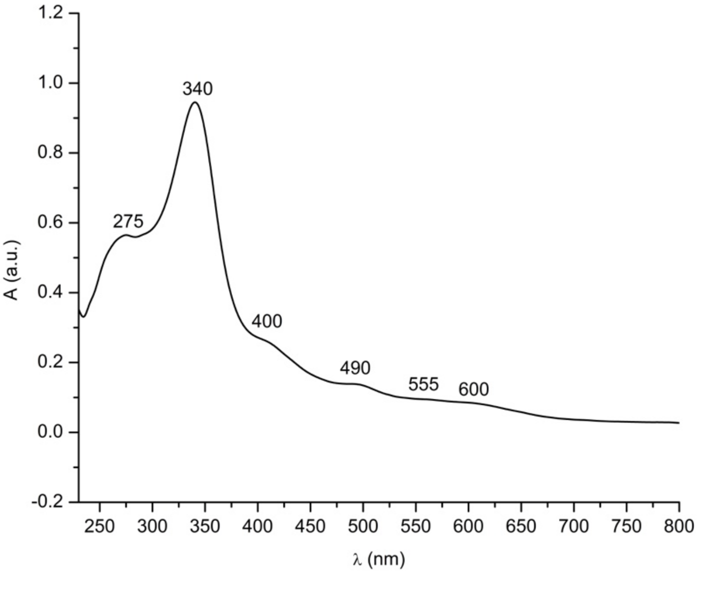

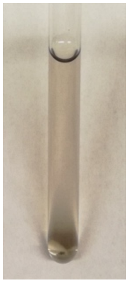

Supplementary Figure 5. Left: UV-VIS spectrum of K_4dbCOT2; Right: Image of a dilute solution of K_4dbCOT2 in THF-d_8.

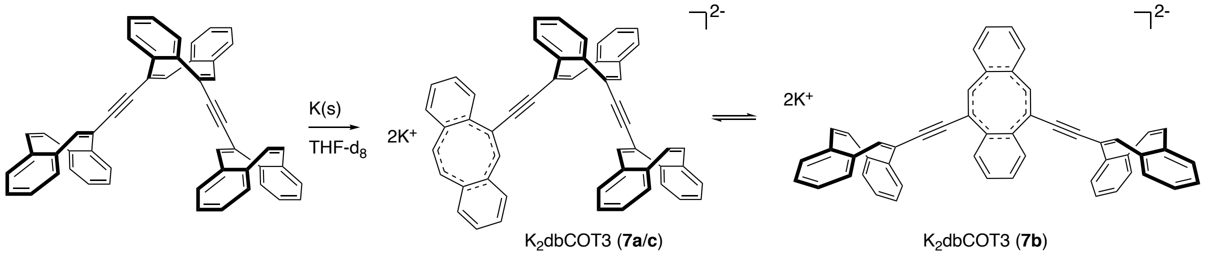

Reduction of dbCOT3 to K_2dbCOT3. Following the general procedure, dbCOT3 (0.34 mg, 0.52 μmol) was dissolved in THF-d_8 (0.45 mL). Upon exposure of the resulting colorless solution to potassium metal (excess), the mixture gradually turned deep green. The reaction was analyzed at regular intervals by ^{1}H NMR spectroscopy (223.2 K) until ~75% conversion into K_2dbCOT2 (mixture of regiosomers) was achieved. Further reduction gave formation also of K_4dbCOT3. Ratio of **7a/c** : **7b**: = 47:53.

^{1}H NMR (500 MHz, THF-d_8, at *T* = 223.2 K) δ: 7a: 9.16 (dd, *J* = 7.4 Hz, 1H), **7b**: 9.08 (dd, *J* = 8.7, 6.7, 3.6 Hz, 2H), 7.92 – 7.88 (m, 1H), 7.79 (t, *J* = 7.9 Hz, 1H), 7.72 (s, 1H), 7.71 (s, 1H), 7.70 – 7.64 (m, 2H), 7.63 (d, *J* = 3.8 Hz, 1H), 7.53 – 7.50 (m, 2H), 7.49 – 6.66 (m, 25H), 6.54 – 6.48 (m, 2H), 6.48 – 6.40 (m, 2H), 6.27 – 6.22 (m, 1H), 6.08 – 6.00 (m, 3H) ppm. Mixture with 25% dbCOT3.

Selected ^{13}C chemical shifts by HSQC (126 MHz, THF-d_8, at *T* = 223.2 K) δ: 135.9, 135.7, 135.4, 134.5, 134.2, 131.4 (two overlapping peaks), 130.2, 129.8, 112.2, 110.44, 110.36, 107.1, 106.5, 98.2, 97.6 ppm.

UV-VIS (λ-max in THF-d_8): 635, 425, 385, 335 nm.

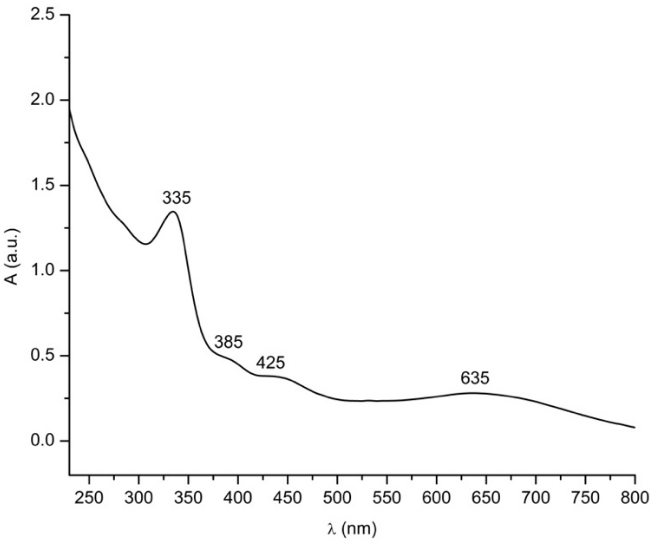

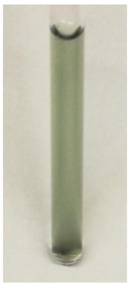

Supplementary Figure 6. Left: UV-VIS spectrum of K_2dbCOT3; Right: Image of a dilute solution of K_2dbCOT3 in THF-d_8.

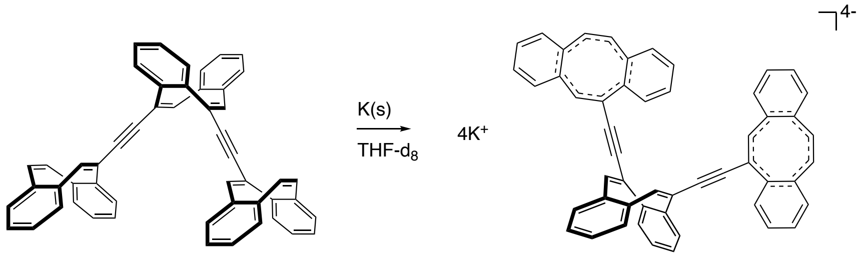

Reduction of dbCOT3 to K_4dbCOT3. Reduction of K_2dbCOT3 was continued following the general procedure. The reaction was analyzed at regular intervals by ^{1}H NMR spectroscopy until a clean formation of K_4dbCOT3 (deep green) was achieved.

^{1}H NMR (500 MHz, THF-d_8) δ: 9.16 (d, J = 7.3 Hz, 2H), 7.84 (d, J = 8.5 Hz, 2H), 7.80 (dd, J = 5.8, 3.4 Hz, 2H), 7.70–7.64 (m, 4H), 7.61 (d, J = 8.5 Hz, 2H), 7.33 (dd, J = 5.8, 3.4 Hz, 2H), 7.13–7.06 (m, 6H), 7.05–7.00 (m, 4H), 6.44 (dd, J = 8.8, 5.8 Hz, 2H), 6.36 (dd, J = 8.4, 5.8 Hz, 2H), 6.19 (dd, J = 8.7, 5.8 Hz, 2H), 6.02 (dd, J = 8.5, 5.8 Hz, 2H) ppm.

^{13}C NMR (126 MHz, THF-d_8) δ: 141.1, 139.9, 136.5, 136.4, 135.6, 132.5, 132.2, 130.7, 130.0, 128.4, 127.0, 126.2, 113.5, 113.3, 112.9, 112.7, 111.6, 111.2, 108.0, 107.9, 103.3, 99.3, 98.3, 91.9, 83.4 ppm. Two additional signals are overlapping.

UV-VIS (λ-max in THF-d_8): 585, 535, 420, 390, 335, 285 nm.

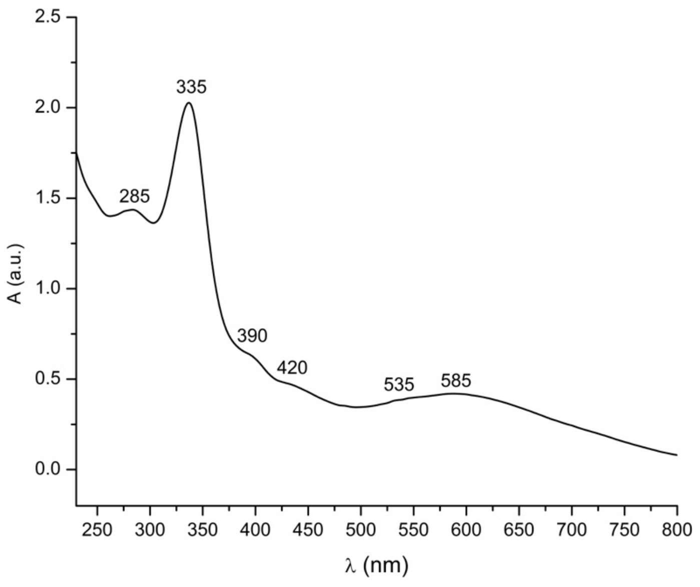

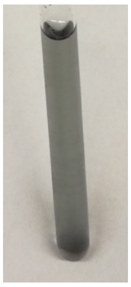

Supplementary Figure 7. Left: UV-VIS spectrum of K_4dbCOT3; Right: Image of a dilute solution of K_4dbCOT3 in THF-d_8.

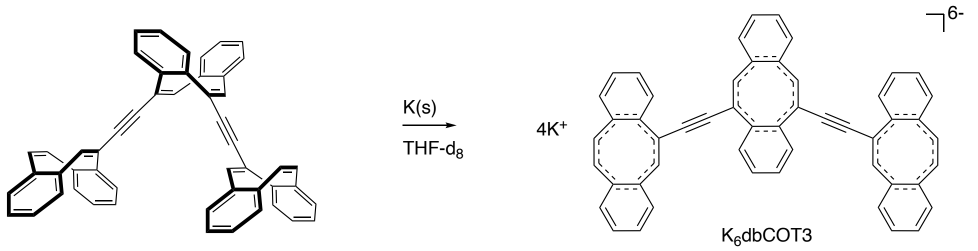

Exhaustive reductive extension of dbCOT3. Reduction of K_4dbCOT3 was continued following the general procedure. The reaction was analyzed at regular intervals by ^{1}H NMR spectroscopy (268.2 K) until a clean formation of K_6dbCOT3 (deep green) was achieved.

^{1}H NMR (500 MHz, THF-d_8, at T = 268.2 K) δ: 10.27 (dd, J = 7.0, 3.5 Hz, 2H), 10.04 (d, J = 9.0 Hz, 2H), 8.56 (s, 2H), 8.55 (s, 2H), 8.08 (d, J = 8.5 Hz, 2H) 8.03 (d, J = 8.5 Hz, 2H), 8.00 (d, J = 7.4 Hz, 2H), 7.83 (d, J = 8.5 Hz, 2H), 7.32 (d, J = 13.2 Hz, 2H), 7.21 (d, J = 13.2 Hz, 2H), 6.79 (dd, J = 7.1, 3.3 Hz, 2H), 6.59 (dd, J = 9.0, 5.6 Hz, 2H), 6.41 (dd, J = 8.5, 5.6 Hz, 2H), 6.29 (dd, J = 8.5, 5.7 Hz, 2H), 6.26 (dd, J = 6.7, 3.1 Hz, 2H), 6.17 (dd, J = 8.4, 5.7 Hz, 2H) ppm.

^{13}C NMR (126 MHz, THF-d$_8$, at *T* = 268.2 K) δ: 136.7, 136.45, 136.35, 135.5, 134.5, 134.2, 112.0, 111.2, 111.1, 110.5. 110.4, 110.3, 110.2, 110.0, 109.6, 109.5, 108.7, 108.2, 104.6, 101.5, 100.9, 100.3, 98.8, 97.4, 90.4, 89.4 ppm.

UV-VIS (λ-max THF-d$_8$): 615, 575, 395, 340, 280 nm.

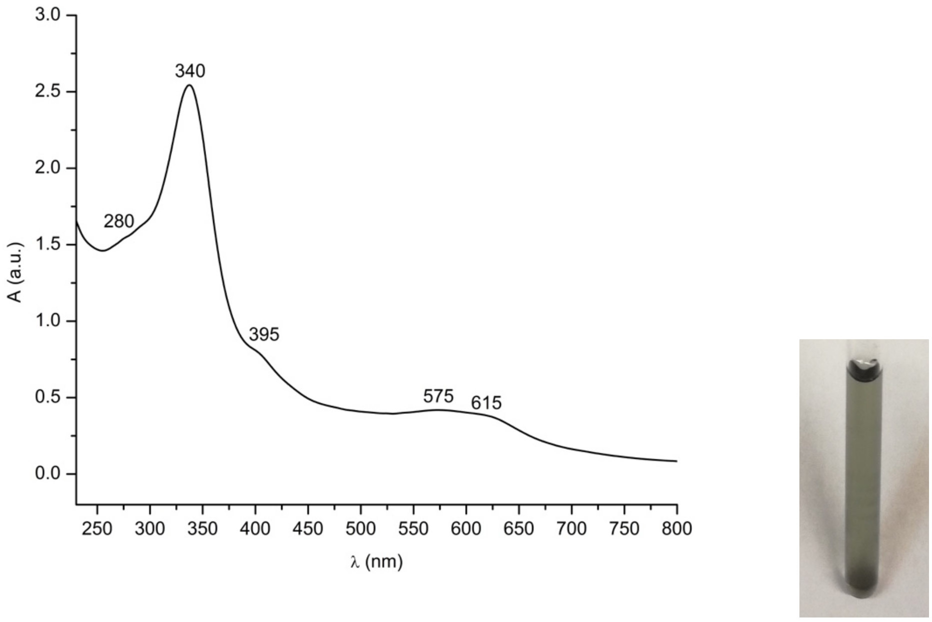

Supplementary Figure 8. Left: UV-VIS spectrum of K$_6$dbCOT3; Right: Image of a dilute solution of K$_6$dbCOT3 in THF-d$_8$.

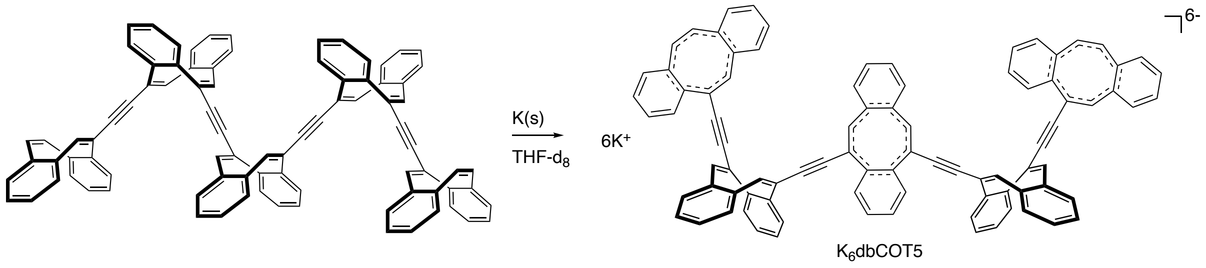

Reduction of dbCOT5 to K$_6$dbCOT5. Following the general procedure, dbCOT5 (0.60 mg, 0.52 µmol) was dissolved in THF-d$_8$ (0.50 mL). Upon exposure of the resulting colorless solution to potassium metal (excess) the mixture gradually turned deep green. The reaction was analyzed at regular intervals by ^{1}H NMR spectroscopy until a clean formation of K$_6$dbCOT5 (deep green) was achieved.

^{1}H NMR (500 MHz, THF-d$_8$, at *T* = 273.0 K) δ: 9.27 – 9.22 (m, 1H), 9.22 (d, *J* = 9.0 Hz, 1H), 7.89 (d, *J* = 7.6 Hz, 1H), 7.83 (d, *J* = 7.3 Hz, 1H), 7.75 (d, *J* = 7.3 Hz, 1H), 7.70 (t, *J* = 4.1 Hz, 1H), 7.70 (d, *J* = 4.0 Hz, 1H), 7.66 (d, *J* = 8.2 Hz, 1H), 7.60 (d, *J* = 4.9 Hz, 1H), 7.42

(dt, J = 5.9, 2.9 Hz, 1H), 7.38 – 7.31 (m, 2H), 7.40 – 7.31 (m, 4H), 7.14 (s, 2H), 7.03 (d, J = 1.7 Hz, 1H), 6.99 (br s, 1H), 6.59 (dd, J = 6.8, 3.5 Hz, 1H), 6.50 – 6.44 (m, 1H), 6.43 – 6.38 (m, 1H), 6.26 – 6.21 (m, 1H), 6.08 – 6.04 (m, 1H), 6.00 (dt, J = 6.4, 2.9 Hz, 1H) ppm.

Selected ^{13}C chemical shifts by HSQC (126 MHz, THF-d$_8$, at T = 273.0 K) δ: 135.4, 135.3, 135.1, 134.4, 131.6, 131.4, 129.6, 129.5, 128.9, 128.82, 127.9, 127.1, 126.0, 125.9, 125.2 (two signals overlapping), 111.9, 111.5, 110.37, 110.36, 110.1, 106.8, 106.3, 102.1, 98.2, 97.1 ppm.

UV-VIS (λ-max in THF-d$_8$): 530, 330, 265 nm.

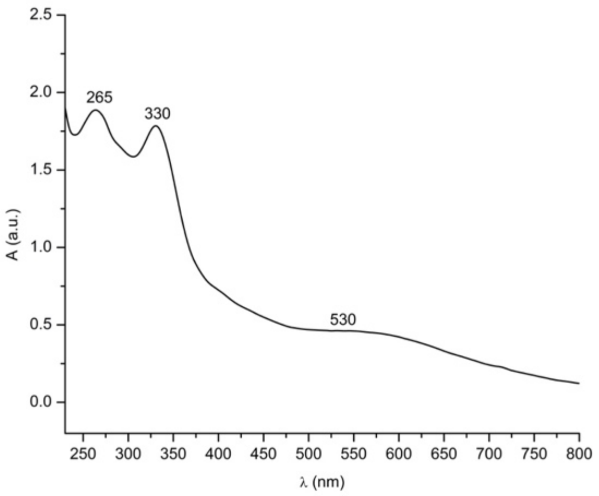

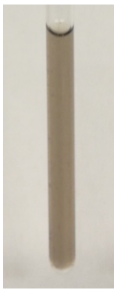

Supplementary Figure 9. Left: UV-VIS spectrum of K$_6$dbCOT5; Right: Image of a dilute solution of K$_6$dbCOT5 in THF-d$_8$.

V. Structural and conformational assignment of dbCOT2, K$_2$dbCOT2, K$_4$dbCOT2, K$_2$dbCOT3, K$_4$dbCOT3, K$_6$dbCOT3, K$_6$dbCOT5

The ^{1}H NMR spectra of reduced derivatives of dbCOT2, dbCOT3, and dbCOT5 were assigned using a combination of one-dimensional (1D) and two-dimensional (2D) NMR experiments: COSY, ^{1}H-^{13}C HMBC, ^{1}H-^{13}C HSQC, and ROESY. For simplicity, the ^{1}H NMR peaks are labeled with the carbon to which the respective proton is attached (H(Cn)). From the left, as drawn, positions on a second dbCOT unit are designated Cn' and on a third unit Cn''. Where needed for clarity, unlabeled carbons are given the name of the closest carbon with a C–H bond that has the lowest number together with "adjacent" (*e.g.* C6'adjacent for the carbon between C6' and C7'). ^{13}C NMR chemical shifts needed for assignment are listed. Fluxionality refers to electron relay between adjacent dbCOT units in partially reduced structures.

Each dbCOT unit is stereogenic (planar chirality) and the stereoisomers rapidly interconvert at room temperature. Oligomers that exhibit diastereoisomerism are characterized as a mixture of diastereomers since the chemical shift differences between diastereomers are small. Structures where the planar chirality of non-reduced units breaks a symmetrical arrangement of reduced units into diastereomers (*e.g.*, K_2dbCOT3 b-series and K_6dbCOT5) are described as *pseudo*-symmetrical.

Assignment of dbCOT2.

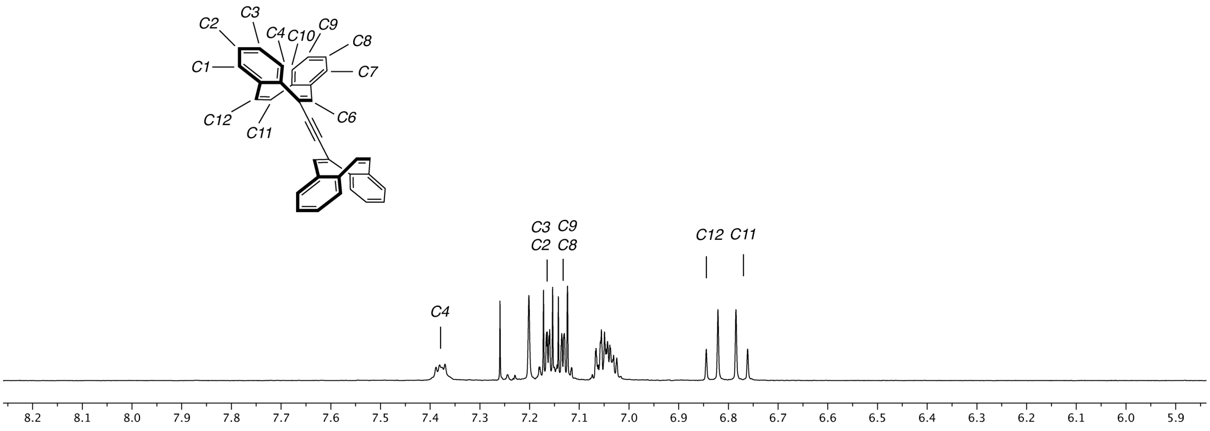

Supplementary Figure 10. Selected assignments in the ^{1}H NMR spectrum (500 MHz) of K_2dbCOT2 in $CDCl_3$ at 373.0 K.

Supplementary Table 2. Assigned ^{1}H chemical shifts and relevant ^{13}C chemical shifts for dbCOT2 in $CDCl_3$ at 373.1 K.

Proton	^{1}H δ (ppm)	Carbon	^{13}C δ (ppm)	Proton	^{1}H δ (ppm)	Carbon	^{13}C δ (ppm)
H(C1)	7.04	C1		H(C8)	7.13	C8	127.1/127.4
H(C2)	7.16	C2	127.7/127.2	H(C9)	7.13	C9	127.1/127.4
H(C3)	7.16	C3	127.7/127.2	H(C10)	7.06	C10	
H(C4)	7.38	C4		H(C11)	6.77	C11	
H(C6)	7.20	C6	91.3	H(C12)	6.83	C12	
H(C7)	7.06	C7	128.9				

^{1}H NMR and selected ^{13}C chemical shift assignment of dbCOT2. The NMR chemical shift assignment of dbCOT2 was first performed on a sample in $CDCl_3$ in a Young's NMR tube heated to 373K where the diastereomers are fast interconverting. The number of observed peaks in the ^{1}H and ^{13}C spectra shows that there is a symmetry element (mirror plane/two-fold axis) at the center of the structure. The chemical shifts were referenced to the solvent peaks at 7.26/77.16 ppm.

The peak at δ = 7.20 ppm is the only singlet and is assigned as H(C6). There is a $^{3}J_{CH}$ HMBC correlation from H(C6) to δ = 91.3 ppm which can be assigned to the alkyne carbon. H(C6) has a $^{3}J_{CH}$ correlation to δ = 128.9 ppm. δ = 128.9 ppm shows an HSQC correlation to δ = 7.06 ppm and can be assigned as H(C7)/C7. H(C7) has a COSY correlation to δ = 7.13 ppm, which can be assigned to H(C8). The multiplet peak at δ = 7.13 ppm integrates as 2H and shows two HSQC correlations at δ = 127.1 ppm and 127.4 ppm. δ = 7.06 ppm has another COSY correlation to δ = 6.77 ppm, one part of a characteristic pattern of two strongly coupled vinylic protons. As there are no other COSY correlations from δ = 7.06 ppm (besides a weak correlation to H(C6)), we can assign δ = 6.77 ppm as H(C11) and H(C10) as having a chemical shift very close to H(C7). δ = 7.13 ppm has no other COSY correlations, besides weak correlations to H(C6) and H(C11). From this it follows that H(C8) and H(C9) have very similar ^{1}H chemical shifts, δ = 7.13 ppm, but slightly different ^{13}C chemical shifts, δ = 127.1 ppm and 127.4 ppm. They could, however, not be disambiguated. H(C11) has a COSY correlation to δ = 6.83 ppm, the other part of the strongly coupled resonances which is assigned to H(C12). H(C12) has a COSY correlation to δ = 7.04 ppm, assigned as H(C1). H(C1) has a COSY correlation to δ = 7.16 ppm, assigned as H(C2). The multiplet peak at δ = 7.16 ppm integrates as 2H and shows two HSQC correlations at δ = 127.2 ppm and 127.7 ppm. From δ = 7.16 ppm there is a COSY correlation to 7.38 ppm, which is assigned to H(C4). The proximity to the alkyne group causing a down-field shift change. As there are no other COSY correlations from δ = 7.16 ppm, besides a weak correlation to H(C12), it follows that H(C2) and H(C3) have very similar ^{1}H chemical shifts, δ = 7.16 ppm, but slightly different ^{13}C chemical shifts, δ = 127.2 ppm and 127.7 ppm. They could, however, not be disambiguated.

The ^{1}H and ^{13}C resonance assignments of C2, C3, C8, and C9 of dbCOT2 in $CDCl_3$ at 373.1 K could then be transferred to spectra recorded in $CDCl_3$ at 295.0 K by direct comparison, and

further on to data acquired in THF-d_8 at 295.0 K: H(C2)/H(C3): δ = 7.16 ppm; C2/C3: δ = 127.8/128.2 ppm; H(C8)/H(C9): δ = 7.12 ppm; C8/C9: δ = 127.7/128.1 ppm.

Assignment of K_2dbCOT2.

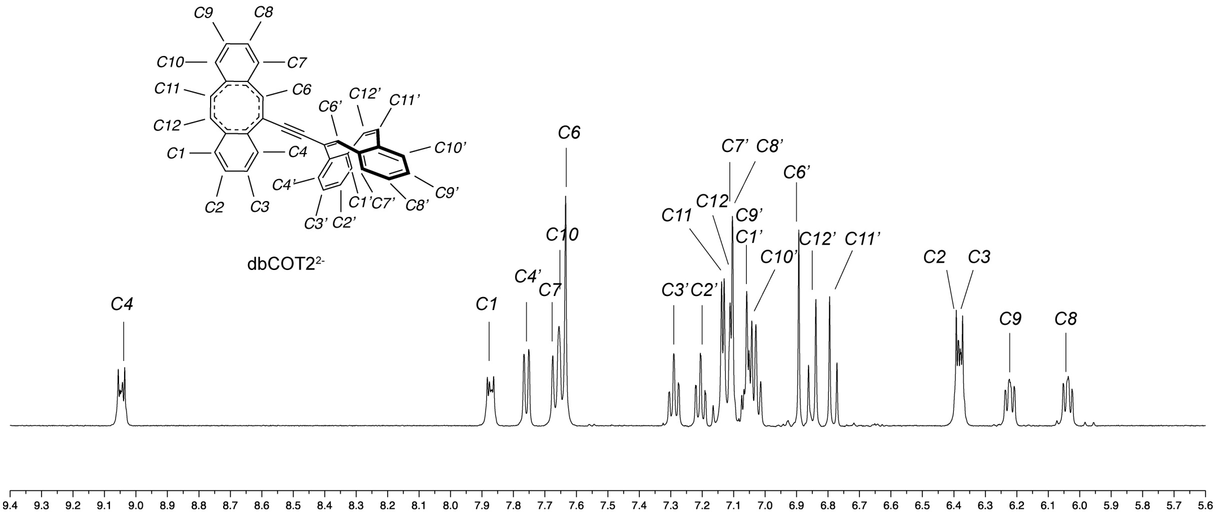

Supplementary Figure 11. Assigned ^{1}H NMR spectrum (500 MHz) of K_2dbCOT2 in THF-d_8. Spectrum recorded at T = 237.2 K.

Supplementary Table 3. Assigned ^{1}H chemical shifts and relevant ^{13}C chemical shifts for K_2dbCOT2

Proton	^{1}H δ (ppm)	Carbon	^{13}C δ (ppm)	Proton	^{1}H δ (ppm)	Carbon	^{13}C δ (ppm)
H(C1)	7.87	C1	135.4	H(C1')	7.05	C1'	128.1
H(C2)	6.38	C2		H(C2')	7.20	C2'	126.1
H(C3)	6.38	C3		H(C3')	7.29	C3'	126.2
H(C4)	9.05	C4		H(C4')	7.76	C4'	130.0
H(C6)	7.63	C6	98.1	H(C6')	6.89	C6'	
H(C7)	7.66	C7	134.3	H(C7')	7.11	C7'	129.1
H(C8)	6.22	C8		H(C8')	7.05	C8'	125.3
H(C9)	6.04	C9	106.6	H(C9')	7.11	C9'	126.3
H(C10)	7.64	C10	135.4	H(C10')	7.02	C10'	128.5
H(C11)	7.13	C11	102.5	H(C11')	6.78	C11'	

H(C12)	7.11	C12	97.2	H(C12’)	6.85	C12’

^{1}H NMR chemical shift assignment of K_2dbCOT2. The number of signals in the ^{1}H NMR spectrum shows that there are no higher symmetry elements in the structure. Broad peaks in the room temperature ^{1}H NMR spectrum that sharpen upon cooling, together with the presence of exchange peaks in the 1D ROE and 2D ROESY experiments show that the structure is fluxional.

Each of the two dbCOT units has one proton without neighboring protons: H(C6) and H(C6’). These serve as starting point for the assignment. There are two singlets in the ^{1}H NMR spectrum: δ = 7.63 ppm and δ = 6.89 ppm. As protons on aromatic rings are deshielded, δ = 7.63 ppm is assigned as H(C6), and δ = 6.89 ppm as H(C6’).

We then focus on the reduced dbCOT unit. H(C6) has a J_{CH} HSQC correlation to δ = 98.1 ppm which is assigned to C6. H(C6) has a $^{3}J_{CH}$ HMBC correlation to δ = 134.3 ppm. δ = 134.3 ppm has an HSQC correlation to δ = 7.66 ppm which is assigned as H(C7) on account of being the only proximal carbon to C6 that has a C–H bond (HSQC correlation). The assignment of C7 to δ = 134.3 ppm follows. H(C7) has a $^{3}J_{HH}$ COSY correlation to δ = 6.22 ppm which is assigned as H(C8). H(C8) has a COSY correlation to δ = 6.04 ppm which is assigned as H(C9). The HSQC correlation at δ = 6.04/106.6 ppm identifies C9. H(C9) has a COSY correlation to δ = 7.64 ppm which is assigned as H(C10). H(C10) has an HSQC correlation to δ = 135.4 ppm which is assigned as C10. H(C10) has $^{2/3}J_{CH}$ HMBC correlations to δ = 102.5 ppm and to δ = 111.9 ppm. Only the peak at δ = 102.5 ppm has an HSQC correlation and is assigned to C11. C11 has an HSQC correlation to δ = 7.13 and is assigned as H(C11). H(C11) has a $^{2}J_{CH}$ HMBC correlation to δ = 97.2 ppm. The signal at δ = 97.2 ppm has an HSQC correlation to δ = 7.11 ppm and is assigned as H(C12). The assignment of C12 to δ = 97.2 ppm follows. C12 has a $^{3}J_{CH}$ HMBC correlation to δ = 7.87 which is assigned to H(C1). H(C1) has an HSQC correlation to δ = 135.4 ppm which is assigned to C1. H(C1) has a COSY correlation to δ = 6.38 ppm which is assigned as H(C2). H(C2) and H(C3) have very similar chemical shifts, and the integral of the resonance at δ = 6.38 ppm shows that it corresponds to two protons. They also have very similar carbon chemical shifts, since there is only one HSQC correlation observed for δ = 6.38 ppm. The slightly skewed HSQC peak shape also indicates two overlapping peaks. H(C3) has a COSY correlation to δ = 9.05 ppm which is assigned as H(C4). H(C4) has a $^{4}J_{CH}$ HMBC

correlation to the alkyne carbon, corroborating the assignment. The extreme chemical shift of H(C4) is diagnostic of proximity to the alkyne moiety.

We then turn to the non-reduced dbCOT unit. First, we identify the roofed doublets at δ = 6.85 ppm and δ = 6.78 ppm in the ^{1}H NMR spectrum as characteristic of the two strongly coupled vinylic protons on the terminal non-reduced unit, H(C11') and H(C12'). The fluxional nature of K_2dbCOT2 is shown by selective inversion of H(C4) of the reduced dbCOT unit in a 1D NOESY experiment (see Figure 6A). This gives rise to an exchange peak with a proton chemical shift of 7.76 ppm within the non-reduced dbCOT unit and is assigned to H(C4'). There is a COSY correlation from H(C4') to δ = 7.29 ppm which is assigned as H(C3'). There is a COSY correlation from H(C3') to δ = 7.20 ppm which is assigned as H(C2'). There is a COSY correlation from H(C2') to δ = 7.05 ppm which is assigned to H(C1'). Observed HSQC correlations at these ^{1}H chemical shifts identify the ^{13}C chemical shifts of C1' (128.1 ppm), C2' (126.1 ppm), C3' (126.2 ppm), and C4' (130.0 ppm). A $^3J_{CH}$ HMBC correlation from C1' to δ = 6.85 ppm identifies this resonance (see above) as H(C12'). It then follows that the peak at δ = 6.78 ppm can be assigned as H(C11'). A $^3J_{CH}$ HMBC correlation from H(C11') to δ = 128.5 ppm identifies C10', and an HSQC correlation from C10' to 7.02 ppm identifies H(C10'). An HMBC correlation is observed from H(C6') at δ = 6.89 ppm to δ = 129.1 ppm, which is assigned as C7', and an HSQC correlation from C7' to δ = 7.11 ppm identifies H(C7'). The two remaining correlations observed in the HSQC spectrum, at 7.05/125.3 ppm and 7.11/126.3 ppm, must correspond to the remaining unassigned C8' and C9'. To assign these, we start with a search for HMBC correlations to the carbons without C–H bonds (absence of HSQC correlations). The carbon located between C6' and C7', here called C6'adjacent, can be assigned as δ = 138.7 ppm based on HMBC correlations from this signal to H(C6'), H(C7'), H(C7'), and H(C11'). An additional weak HMBC correlation to δ = 7.05 ppm, can then be assigned to H(C8'). It follows that δ = 7.11 ppm is assigned as H(C9') and concludes the assignment. Cross check of HMBC correlations corroborates the analysis.

Assignment of K_4dbCOT2.

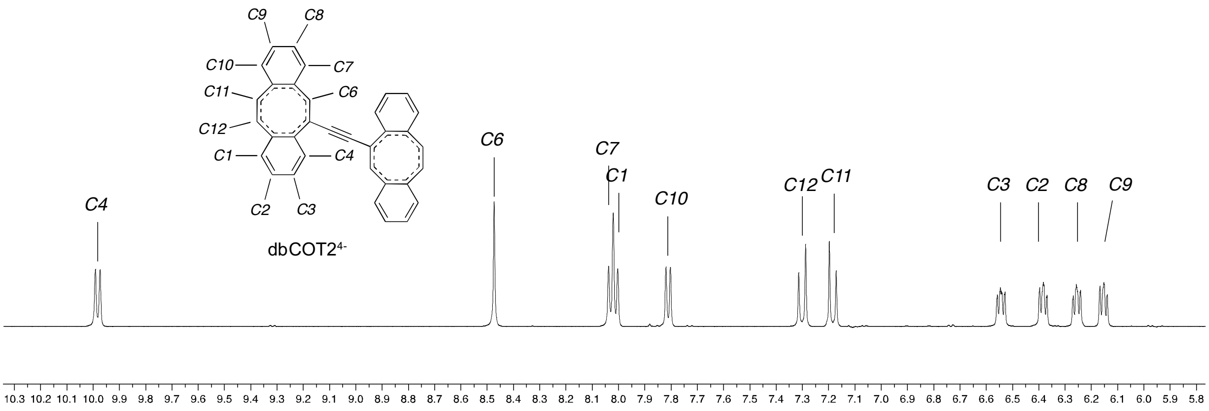

Supplementary Figure 12. Assigned ^{1}H NMR spectrum (500 MHz) of K_4dbCOT2 in THF-d_8.

Supplementary Table 4. Assigned ^{1}H chemical shifts and relevant ^{13}C chemical shifts for K_4dbCOT2

Proton	^{1}H δ (ppm)	Carbon	^{13}C δ (ppm)	Proton	^{1}H δ (ppm)	Carbon	^{13}C δ (ppm)
H(C1)	8.02	C1	136.7	H(C8)	6.26	C8	
H(C2)	6.39	C2		H(C9)	6.16	C9	
H(C3)	6.55	C3		H(C10)	7.81	C10	135.5
H(C4)	9.99	C4		H(C11)	7.19	C11	
H(C6)	8.48	C6		H(C12)	7.30	C12	
H(C7)	8.03	C7					

^{1}H NMR chemical shift assignment of K_4dbCOT2. A lack of broadening in the room temperature ^{1}H NMR spectrum show that there is no fluxionality in the structure. The number of peaks in the ^{1}H and ^{13}C spectra show that there is a symmetry element (mirror plane/two-fold axis) at the center of the structure.

The peak at δ = 8.48 ppm is the only singlet and is assigned as H(C6). There is a $^{3}J_{CH}$ HMBC correlation from H(C6) to δ = 89.4 ppm which can be assigned to the alkyne carbon. There is also a $^{4}J_{CH}$ HMBC correlation from this carbon to δ = 9.99 ppm, which is assigned to H(C4). The extreme downfield chemical shift of H(C4) results from proximity to the alkyne and is diagnostic of H(C4). H(C4) has a $^{3}J_{HH}$ COSY correlation to δ = 6.55 ppm which is assigned as H(C3). H(C3) has a COSY correlation to δ = 6.39 ppm which is assigned as H(C2). H(C2) has a COSY correlation to δ = 8.02 ppm which is assigned as H(C1). H(C1) has a J_{CH} HSQC

correlation to δ = 136.7 ppm assigned to C1. There is a $^{3}J_{CH}$ HMBC correlation from C1 to δ = 7.30 ppm which is assigned as H(C12). There is a COSY correlation from H(C12) to δ = 7.19 ppm which is assigned as H(C11). There is a $^{3}J_{CH}$ HMBC correlation from H(C11) to δ = 135.5 ppm which is assigned as C10. There is an HSQC correlation from C10 to δ = 7.81 ppm which is assigned as H(C10). There is a COSY correlation from H(C10) to δ = 6.16 ppm which is assigned as H(C9). There is a COSY correlation from H(C9) to δ = 6.26 ppm which is assigned as H(C8). There is a COSY correlation from H(C8) to δ = 8.03 ppm which is assigned to H(C7) and concludes the assignment.

Assignment of K_2dbCOT3.

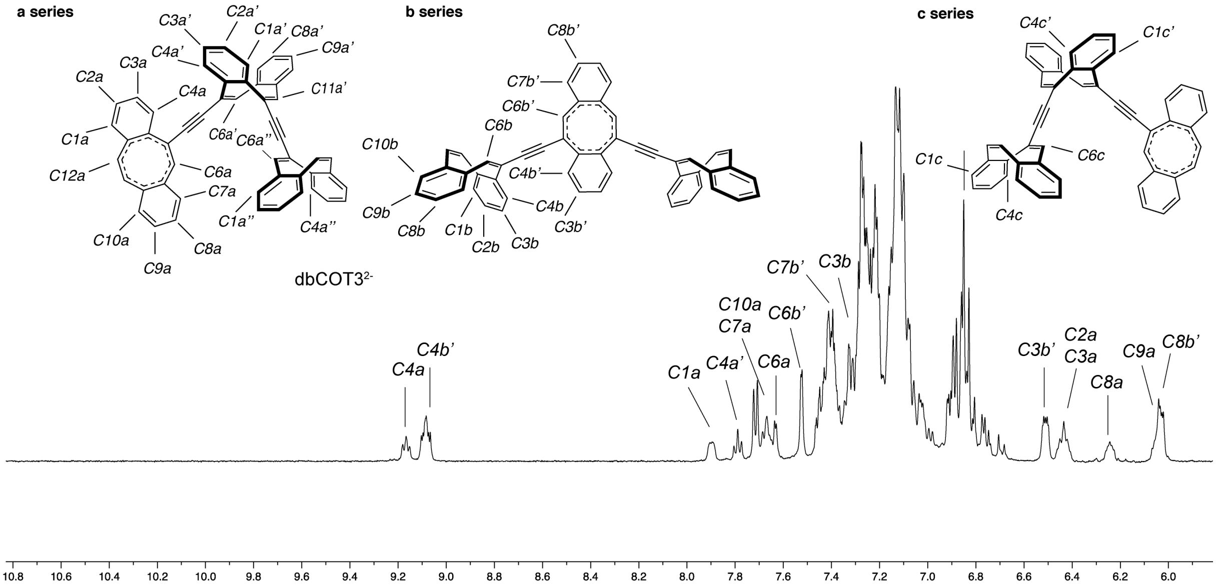

Supplementary Figure 13. Selected assignments in the ^{1}H NMR spectrum (500 MHz) of K_2dbCOT3 in THF-d_8. Spectrum recorded at T = 223.9 K.

Supplementary Table 5. Assigned ^{1}H chemical shifts and relevant ^{13}C chemical shifts for K_2dbCOT3.

Proton	^{1}H δ (ppm)	Carbon	^{13}C δ (ppm)	Proton	^{1}H δ (ppm)	Carbon	^{13}C δ (ppm)
**a-series**				**b-series**			
H(C1a)	7.89	C1a		H(C1b)	7.08	C1b	
H(C2a)	6.43	C2a		H(C2b)	7.32/7.25	C2b	
H(C3a)	6.45	C3a		H(C3b)	7.32/7.25	C3b	

H(C4a)	9.16	C4a		H(C4b)	7.71	C4b
H(C6a)	7.63	C6a	98.2	H(C6b)	6.85	C6b
H(C7a)	7.67	C7a	134.5	H(C8b)	7.14	C8b
H(C8a)	6.24	C8a		H(C9b)	7.13	C9b
H(C9a)	6.05	C9a		H(C10b)	7.38	C10b
H(C10a)	7.66	C10a		H(C3b’)	6.51	C3a’
H(C12a)	7.14	C12a		H(C4b’)	9.08	C4a’
H(C1a’)	7.39	C1a’		H(C6b’)	7.52	C6a’
H(C2a’)	7.42/7.30	C2a’		H(C7b’)	7.40	C7a’
H(C3a’)	7.42/7.30	C3a’		H(C8b’)	6.02	C8a’
H(C4a’)	7.79	C4a’				
H(C6a’)	7.25	C6a’		**c-series**		
H(C8a’)	7.16/7.11	C8a’		H(C1c)	7.02	C1c
H(C9a’)	7.16/7.11	C9a’		H(C4c)	7.38	C4c
H(C11a’)	6.90	C11a’		H(C6c)	7.16	C6c
H(C1a’’)	7.02	C1a’’		H(C1c’)	7.79	C1c’
H(C4a’’)	7.38	C4a’’		H(C4c’)	7.39	C4c’
H(C6a’’)	7.16	C6a’’				

^{1}H NMR chemical shift assignment of K_2dbCOT3. Broad peaks in the room temperature ^{1}H NMR spectrum that sharpen upon cooling show that the structure is fluxional. The b-series structure has a *pseudo*-symmetry plane through the center unit whereas the a/c series are of C_1-symmetry.

The downfield chemical shifts of δ = 9.17 ppm and δ = 9.08 ppm are diagnostic of a proximity to the alkyne spacer why these are assigned as H(C4a)/H(C4b’). Selective excitation of δ = 9.16 ppm in an 1D ROE experiment gives rise to only one exchange peak at δ = 7.71 ppm, whereas selective excitation of δ = 9.08 ppm gives rise to two exchange peaks at δ = 7.79 ppm and δ = 7.39 ppm. From symmetry, it follows that δ = 9.16 ppm is H(C4a) and δ = 9.08 ppm

is H(C4b'). Taking the inherent symmetry of the b-series into account, the integral of H(C4a) and H(C4b') gives a 47:53 ratio of concentrations for the a-series and b-series. Integration between δ = 7.50 ppm and δ = 6.60 ppm shows that the sample contains ~25% unreduced dbCOT3. The exchange experiments prove that the a-series, b-series, and c-series are fluxionally interconverting.

Nota bene: The a-series and c-series structures are identical by ^{1}H NMR spectroscopy. The protons on, for instance, the terminal units are however individual atoms. Magnetization from an individual proton on a terminal reduced unit in the a-series can therefore be transferred to two additional distinct states through rearrangement/electron relay: first to the equivalent position in the b-series, and then further on to the equivalent position on a non-reduced terminal unit in the a-series. This means that electron relay can be followed from **7a** through **7b** to **7c**.

Reduced dbCOT unit in the a-series: H(C4a) has a COSY correlation to δ = 6.45 ppm which is assigned as H(C3a). H(C3a) has a poorly resolved COSY correlation to δ = 6.43 ppm which is assigned as H(C2a). Integration of the signal at 6.43/6.45 ppm shows that it corresponds to two protons, and the HSQC spectrum has two cross peaks at this proton chemical shift. H(C2a) has a COSY correlation to δ = 7.89 ppm which is assigned as H(C1a). Based on the chemical shift, integration (1H), and coupling pattern (*pseudo*-singlet), δ = 7.63 ppm is assigned as H(C6a). H(C6a) has an HSQC correlation to δ = 98.2 ppm which is assigned as C6a. C6a has a $^3J_{CH}$ HMBC correlation to a proton at 7.67 ppm, that in turn has an HSQC correlation to an appropriate chemical shift for C7a, δ = 134.5 ppm. H(C7a) has a COSY correlation to δ = 6.24 ppm which is assigned to H(C8a). H(C8a) has a COSY correlation to δ = 6.05 ppm which is assigned to H(C9a). H(C9a) has a COSY correlation to δ = 7.66 ppm which is assigned to H(C10a).

Reduced dbCOT unit in the b-series: H(C4b') has a COSY correlation to δ = 6.51 ppm which is assigned as H(C3b'). Based on the chemical shift, integration (2H), and coupling pattern (*pseudo*-singlet), δ = 7.52 ppm is assigned as H(C6b'). The assignment is corroborated by the absence of COSY correlations, and the fact that this peak shows exchange to two positions, δ = 7.25 ppm and δ = 6.9 ppm. It must thus originate from two symmetry related protons at the center unit which end up in different chemical environments upon electron relay. From assignment of K_2dbCOT2 and K_4dbCOT2 we know that chemical shifts below ~6.5 ppm are diagnostic of the distal positions on the benzene rings of reduced units. The only remaining signal

in this region (*vide supra*) is δ = 6.02 ppm which is assigned to H(C8b’). H(C8b’) has a COSY correlation to δ = 7.40 ppm which is assigned to H(C7b’).

a-series, assignments from exchange spectroscopy: In an 1D ROE experiment H(C3b’) shows exchange to δ = 7.42 ppm and δ = 7.30 ppm due to a broken symmetry in the a-series structure. These peaks were assigned as H(C3a’)/H(C2a’) but were not disambiguated. H(C4b’) has exchange to δ = 7.79 ppm and to δ = 7.39 ppm. δ = 7.79 ppm was tentatively assigned to H(C4a’) due to a higher shift from deshielding by proximity to the reduced unit (*cf.*, H(C4’) vs H(C1’) in K_2dbCOT2). This gives δ = 7.39 ppm as H(C1a’). H(C6b’) has exchange to δ = 7.25 ppm and δ = 6.90 ppm. δ = 7.25 ppm was tentatively assigned to H(C6a’) due to a higher shift from deshielding by proximity to the reduced unit. This gives δ = 6.90 ppm as H(C11a’). H(C8b’) has exchange to δ = 7.16 ppm and δ = 7.11 ppm due to a broken symmetry in the a-series structure. These were assigned as H(C8a’)/H(C9a’) but were not disambiguated. H(C1a) has a through space ROE correlation to 7.14 which is assigned as H(C12a). This correlation also corroborates the flat geometry of a terminal unit.

b-series, assignments from exchange spectroscopy: H(C1a) has exchange to δ = 7.08 ppm which was assigned as H(C1b). H(C3a)/H(C2a) has exchange to δ = 7.32 ppm and δ = 7.25 ppm. These are assigned as H(C3b)/H(C2b) but were not further disambiguated. H(C4a) has exchange to δ = 7.71 ppm which was assigned as H(C4b). H(C6a) has exchange to δ = 6.85 ppm which was assigned to H(C6b). H(C8a) has exchange to δ = 7.14 ppm which was assigned as H(C8b). H(C9a) has exchange to δ = 7.13 ppm which was assigned as H(C9b). H(C10a) has exchange to δ = 7.38 ppm which was assigned as H(C10b). H(C6b’) has a through space ROE correlation to H(C7b’) which corroborates the flat geometry of the center unit.

c-series, assignments from exchange spectroscopy: H(C1a) has a weak exchange peak to δ = 7.02 ppm which is assigned as H(C1c). H(C1c) is chemically equivalent to H(C1a’’). H(C4a) has a weak exchange peak to δ = 7.38 ppm which was assigned as H(C4c). H(C4c) is chemically equivalent to H(C4a’’). H(C6a) has a weak exchange peak to δ = 7.16 ppm which is assigned as H(C6c). H(C6c) is chemically equivalent to H(C6a’’). H(C1a’) is chemically equivalent to H(C4c’). H(C4a’) is chemically equivalent to H(C1c’).

Assignment of K_4dbCOT3.

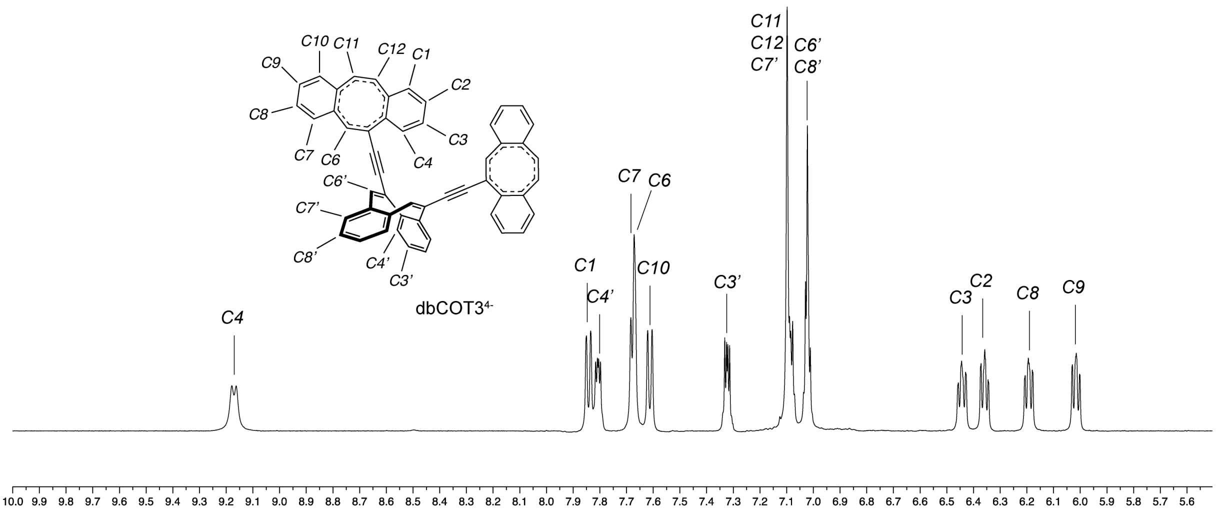

Supplementary Figure 14. Assigned ^{1}H NMR spectrum (500 MHz) of K_4dbCOT3 in THF-d_8.

Supplementary Table 6. Assigned ^{1}H chemical shifts and relevant ^{13}C chemical shifts for K_4dbCOT3

Proton	^{1}H δ (ppm)	Carbon	^{13}C δ (ppm)	Proton	^{1}H δ (ppm)	Carbon	^{13}C δ (ppm)
H(C1)	7.84	C1		H(C10)	7.61	C10	
H(C2)	6.36	C2		H(C11)	7.02	C11	
H(C3)	6.44	C3		H(C12)	7.09	C12	98.3
H(C4)	9.16	C4		H(C3')	7.33	C3'	
H(C6)	7.66	C6	99.3	H(C4')	7.80	C4'	
H(C7)	7.68	C7		H(C6')	7.02	C6'	
H(C8)	6.19	C8		H(C7')	7.08	C7'	130.0
H(C9)	6.02	C9		H(C8')	7.02	C8'	

^{1}H NMR chemical shift assignment of K_4dbCOT3. A lack of significant broadening in the room temperature ^{1}H NMR spectrum shows that there is no fluxionality in the structure. The number of peaks in the ^{1}H and ^{13}C spectra shows that there is a *pseudo*-plane of symmetry at the center of the structure.

The extreme downfield chemical shift of δ = 9.16 ppm is diagnostic of a proximity to the alkyne spacer in a planar structure and is assigned as H(C4). H(C4) has a COSY correlation to δ = 6.44 ppm which was assigned as H(C3). H(C3) has a COSY correlation to δ = 6.36 ppm which

was assigned to H(C2). H(C2) has a COSY correlation to δ = 7.84 ppm which is assigned to H(C1). H(C1) has an $^3J_{CH}$ HMBC correlation to δ = 98.3 ppm which is assigned as C12. C12 has an HSQC correlation to δ = 7.09 ppm which was assigned to H(C12). H(C12) has a COSY correlation to δ = 7.02 ppm which was assigned to H(C11). The singlet at δ = 7.66 ppm was assigned as H(C6) due to a more downfield chemical shift than the singlet at δ = 7.02 ppm. The singlet at δ = 7.02 ppm was assigned as H(C6'). H(C6) has an HSQC correlation to δ = 99.3 ppm which was assigned to C6. C6 has an $^3J_{CH}$ HMBC correlation to δ = 7.68 ppm which was assigned to H(C7). H(C7) has a COSY correlation to δ = 6.19 ppm which was assigned as H(C8). H(C8) has a COSY correlation to δ = 6.02 ppm which was assigned to H(C9). H(C9) has a COSY correlation to δ = 7.61 ppm which was assigned as H(C10).

H(C6') has an $^3J_{CH}$ HMBC correlation to δ = 130.0. δ = 130.0 ppm has an HSQC correlation from to δ = 7.08 ppm which was assigned as H(C7'). H(C7') has a poorly resolved COSY correlation to δ = 7.02 ppm which was assigned to H(C8'). There is a COSY correlation between δ = 7.80 ppm and δ = 7.33 ppm which are assigned to H(C3')/H(C4'). δ = 7.80 ppm has $^{2/3}J_{CH}$ HMBC correlations to two carbons without C–H bond as seen by the absence of HSQC correlations (δ = 108.0 ppm and δ = 111.2 ppm), and δ = 7.33 ppm has only one $^3J_{CH}$ HMBC correlation (δ = 108.0 ppm), which is consistent with δ = 7.80 ppm as H(C4') and δ = 7.33 ppm as H(C3') and concludes the assignment.

Assignment of K_6dbCOT3.

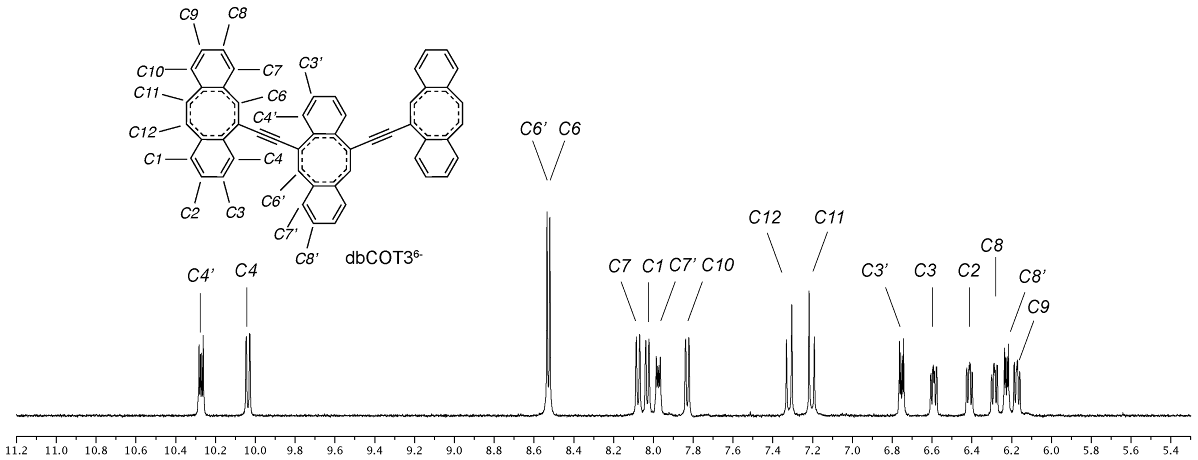

Supplementary Figure 15. Assigned ^{1}H NMR spectrum (500 MHz) of K_6dbCOT3 in THF-d_8. Spectrum recorded at T = 268.2 K.

Supplementary Table 7. Assigned ^{1}H chemical shifts and relevant ^{13}C chemical shifts for K_6dbCOT3

Proton	^{1}H δ (ppm)	Carbon	^{13}C δ (ppm)	Proton	^{1}H δ (ppm)	Carbon	^{13}C δ (ppm)
H(C1)	8.03	C1		H(C10)	7.83	C10	135.5
H(C2)	6.41	C2		H(C11)	7.21	C11	
H(C3)	6.59	C3		H(C12)	7.32	C12	97.4
H(C4)	10.04	C4		H(C3')	6.79	C3'	
H(C6)	8.55	C6	101.5	H(C4')	10.27	C4'	
H(C7)	8.08	C7	136.45	H(C6')	8.56	C6'	104.6
H(C8)	6.29	C8		H(C7')	8.00	C7'	136.35
H(C9)	6.17	C9		H(C8')	6.26	C8'	

^{1}H NMR chemical shift assignment of K_6dbCOT3. A lack of broadening in the room temperature ^{1}H NMR spectrum shows that there is no fluxionality in the structure. From the number of peaks in the ^{1}H and ^{13}C NMR spectra, there must be a symmetry element (plane/two-fold axis) through the center of the structure.

The extreme downfield shift of δ = 10.27 ppm and δ = 10.04 ppm are diagnostic of protons proximal to the alkyne in reduced structures. There is a COSY spin system from δ = 10.04 ppm that connects four distinct signals (as opposed to two), why δ = 10.04 ppm is assigned as H(C4). This assign δ = 10.27 ppm to H(C4'). There is a COSY correlation from H(C4) to δ = 6.59 which is assigned to H(C3). H(C3) has a COSY correlation to δ = 6.41 ppm which is assigned to H(C2). H(C2) has a COSY correlation to δ = 8.03 ppm which was assigned to H(C1). H(C1) has a $^3J_{CH}$ HMBC correlation to δ = 97.4 ppm which is assigned to C12. C12 has an HSQC correlation to δ = 7.32 ppm which is assigned to H(C12). H(C12) has a COSY correlation to δ = 7.21 ppm which is assigned to H(C11). H(C11) has an $^3J_{CH}$ HMBC correlation to δ = 135.45 ppm. δ = 135.45 ppm has an HSQC correlation to δ = 7.83 ppm which was assigned as H(C10). H(C10) has a COSY correlation to δ = 6.17 ppm which was assigned to H(C9). H(C9) has a COSY correlation to δ = 6.29 ppm which was assigned to H(C8). H(C8) has a COSY correlation to δ = 8.08 ppm which was assigned to H(C7). H(C7) has one HMBC correlation to a carbon with an HSQC correlation, δ = 101.5 ppm which was assigned as C6. C6 has an HSQC correlation to δ = 8.55 ppm which was assigned as H(C6).

The only remaining singlet in the spectrum, δ = 8.56 ppm, is assigned as H(C6'). H(C6') has an HSQC correlation to δ = 104.6 ppm is assigned as C6'. H(C6') has a HMBC correlation to δ = 136.35 ppm. δ = 136.35 ppm has an HSQC correlation to δ = 8.00 ppm which is assigned as H(C7') on account of being the only proximal carbon to H(C6') that has a C–H bond (HSQC correlation). H(C7') has a COSY correlation to δ = 6.26 ppm which is assigned as H(C8'). H(C4') has a COSY correlation to δ = 6.79 ppm which is assigned as H(C3') and concludes the assignment.

Assignment of K_6dbCOT5.

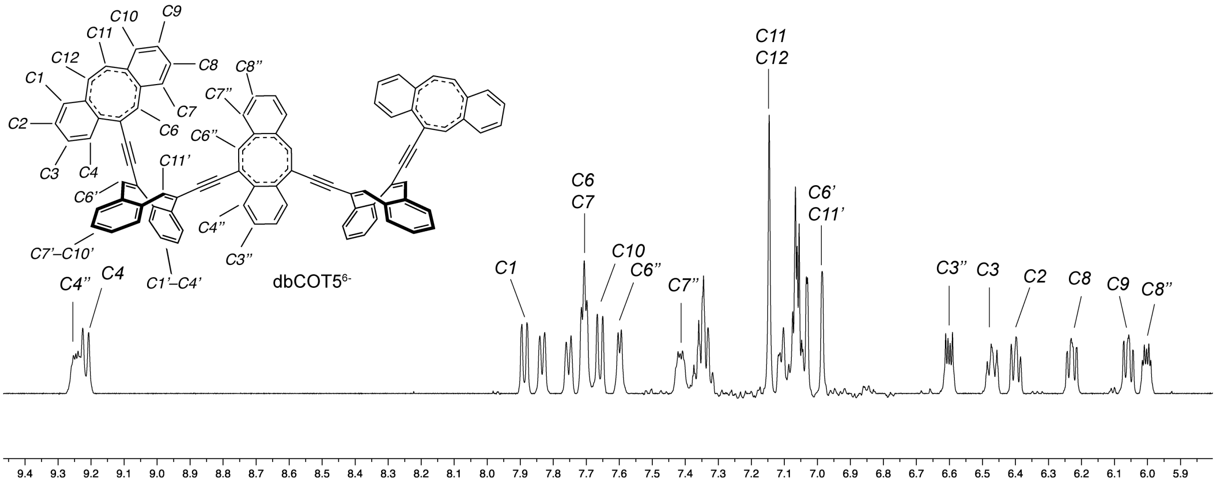

Supplementary Figure 16. Selected assignments in the ^{1}H NMR spectrum (500 MHz) of K_6dbCOT5 in THF-d_8. All Spectrum recorded at T = 273.0 K.

Supplementary Table 8. Assigned ^{1}H chemical shifts and relevant ^{13}C chemical shifts for K_6dbCOT5

Proton	^{1}H δ (ppm)	Carbon	^{13}C δ (ppm)	Proton	^{1}H δ (ppm)	Carbon	^{13}C δ (ppm)
H(C1)	7.89	C1		H(C3')	see text	C3'	
H(C2)	6.42	C2		H(C4')	see text	C4'	
H(C3)	6.46	C3		H(C6')	7.03/6.99	C6'	127.1/127.9
H(C4)	9.22	C4		H(C7')	see text	C7'	
H(C6)	7.70	C6	98.2	H(C8')	see text	C8'	
H(C7)	7.70	C7	135.5	H(C9')	see text	C9'	

H(C8)	6.23	C8		H(C10')	see text	C10'	
H(C9)	6.06	C9		H(C11')	7.03/6.99	C11'	127.1/127.9
H(C10)	7.66	C10	135.3	H(C3'')	6.60	C3''	
H(C11)	7.14	C11	102.1	H(C4'')	9.24	C4''	
H(C12)	7.14	C12	97.1	H(C6'')	7.59	C6''	
H(C1')	see text	C1'		H(C7'')	7.42	C7''	135.1
H(C2')	see text	C2'		H(C8'')	6.00	C8''	

^{1}H NMR chemical shift assignment of K_6dbCOT5. A lack of significant broadening in of signals in the ambient temperature ^{1}H NMR spectrum shows that there is no fluxionality in the structure. The number of peaks in the ^{1}H and ^{13}C spectra shows that there is a *pseudo*-plane of symmetry at the center of the structure.

The extreme downfield shift of δ = 9.24 ppm and δ = 9.22 ppm are diagnostic of protons proximal to the alkyne. Of these δ = 9.22 ppm, but not δ = 9.24 ppm, is part of a COSY spin system that connects four peaks. δ = 9.22 ppm is therefore assigned as H(C4) and δ = 9.24 ppm as (HC4'').

Terminal reduced dbCOT unit. H(C4) has a COSY correlation to δ = 6.46 ppm which is assigned as H(C3). H(C3) has a COSY correlation to δ = 6.42 ppm and is assigned to H(C2). H(C2) has a COSY correlation to δ = 7.89 which is assigned as H(C1). H(C1) has a $^3J_{CH}$ HMBC correlation to δ = 97.1 ppm which is assigned as C12. C12 has an HSQC correlation to δ = 7.14 ppm which is assigned to H(C12). H(C12) has a $^2J_{CH}$ HMBC correlation to δ = 102.1 ppm which is assigned to C11. C11 has an HSQC correlation to δ = 7.14 ppm which is assigned as H(C11). H(C11) and H(C12) thus overlaps, but C11 and C12 do not. H(C11) has an $^3J_{CH}$ HMBC correlation to δ = 135.3 ppm which, in turn, has an HSQC correlation to δ = 7.66 ppm and is assigned to H(C10). H(C10) has a COSY correlation to δ = 6.06 ppm which is assigned to H(C9). H(C9) has a COSY correlation to δ = 6.23 ppm which is assigned as H(C8). H(C8) has a COSY correlation to δ = 7.70 ppm which is assigned to H(C7). H(C7) has an $^3J_{CH}$ HMBC correlation to δ = 98.2 ppm and to δ = 135.5 ppm. Both peaks have HSQC correlations to δ = 7.70 ppm and H(C6) is assigned to δ = 7.70 ppm (integral 2H). By analogy with chemical shifts in the K_2dbCOT3 a-series, δ = 98.2 ppm is assigned to C6 and δ = 135.5 ppm to C7.

Central reduced dbCOT unit. H(C4'') has a COSY correlation to δ = 6.60 ppm which is assigned to H(C3''). δ = 6.00 is assigned as H(C8'') being the only unaccounted signal with a diagnostic shielding. H(C8'') has a COSY correlation to δ = 7.42 ppm which is assigned as H(C7''). H(C7'') has an HSQC correlation to δ = 135.1 ppm which is assigned as C7''. C7'' has an $^3J_{CH}$ HMBC correlation to δ = 7.59 ppm which is assigned as H(C6'').

Non-reduced dbCOT unit. δ = 7.03 ppm has an HSQC correlation to δ = 127.1 ppm and δ = 6.99 ppm has an HSQC correlation to δ = 127.9 ppm. Based on the integrals, the chemical shifts and coupling patterns of these peaks are assigned as H(C6')/H(C11') but could not be disambiguated. δ = 7.03 ppm has an $^3J_{CH}$ HMBC correlation to δ = 128.86 ppm. δ = 128.86 ppm has an HSQC correlation to δ = 7.10 ppm. δ = 7.10 ppm has an $^2J_{CH}$ HMBC correlation to δ = 125.2 ppm. δ = 125.2 ppm has an HSQC correlation to δ = 7.05 ppm. δ = 6.99 ppm has an $^3J_{CH}$ HMBC correlation to δ = 128.82 ppm. δ = 128.82 ppm has an HSQC correlation to δ = 7.06 ppm. The integral between δ = 7.08 ppm and δ = 7.04 ppm reveals three protons. δ = 7.05/125.2 ppm is therefore assigned as two CH groups on adjacent positions supported by the lack of clear COSY correlations. Starting from a vinylic proton following the carbon chain, this gives an order of δ = 7.03 ppm ⇒ δ = 7.10 ppm ⇒ δ = 7.05 ppm ⇒ δ = 7.05 ppm δ = 7.06 ppm ⇒ δ = 6.99 ppm corresponding to H(C6') ⇒ H(C7') ⇒ H(C8') ⇒ H(C9') ⇒ H(C10') ⇒ H(C11') or the reverse. This assignment is further corroborated by the chemical shifts which parallels those of equivalent positions in K_4dbCOT3.

There is a COSY correlation from δ = 7.75 ppm to δ = 7.33 ppm. There is a COSY correlation from δ = 7.33 ppm to δ = 7.35 ppm. There is a COSY correlation from δ = 7.35 ppm to δ = 7.83 ppm. There is an $^3J_{CH}$ HMBC correlation from δ = 7.03 ppm to δ = 139.9 ppm. There is an $^3J_{CH}$ HMBC correlation from δ = 7.35 ppm to δ = 139.9 ppm. δ = 139.9 ppm does not have an HSQC correlation and is assigned as C1'adjacent or C4'adjacent. There is an $^3J_{CH}$ HMBC correlation from δ = 6.99 ppm to δ = 139.5 ppm. There is an $^3J_{CH}$ HMBC correlation from δ = 7.33 ppm to δ = 139.5 ppm. δ = 139.5 ppm does not have an HSQC correlation and is assigned as C1'adjacent or C4'adjacent. From this, we assign δ = 7.35 ppm to as proximal to δ = 7.03 ppm and δ = 7.33 ppm to as proximal to δ = 6.99 ppm. Starting from a vinylic proton following the carbon chain, this gives an order of δ = 7.03 ppm ⇒ δ = 7.83 ppm ⇒ δ = 7.35 ppm ⇒ δ = 7.33 ppm δ = 7.75 ppm ⇒ δ = 6.99 ppm corresponding to H(C6') ⇒ H(C1') ⇒ H(C2') ⇒

H(C3’) ⇒ H(C4’) ⇒ H(C11’) or the reverse. This assignment is supported by the chemical shifts which parallels those of equivalent positions in K_4dbCOT3.

Confirmation of the planar conformation of reduced dbCOT units.

Partially reduced oligomers: The planar nature of one of the dbCOT units in K_2dbCOT2 was confirmed by a pronounced ROE between *H*–(C1) and *H*–(C12) in K_2dbCOT2 (Supplementary Figure 17). The extreme chemical shift of *H*–(C4), which originates from being in close proximity to the alkyne, further strengthens this assignment. K_2dbCOT3, K_4dbCOT3, and K_6dbCOT5 were assigned by analogy.

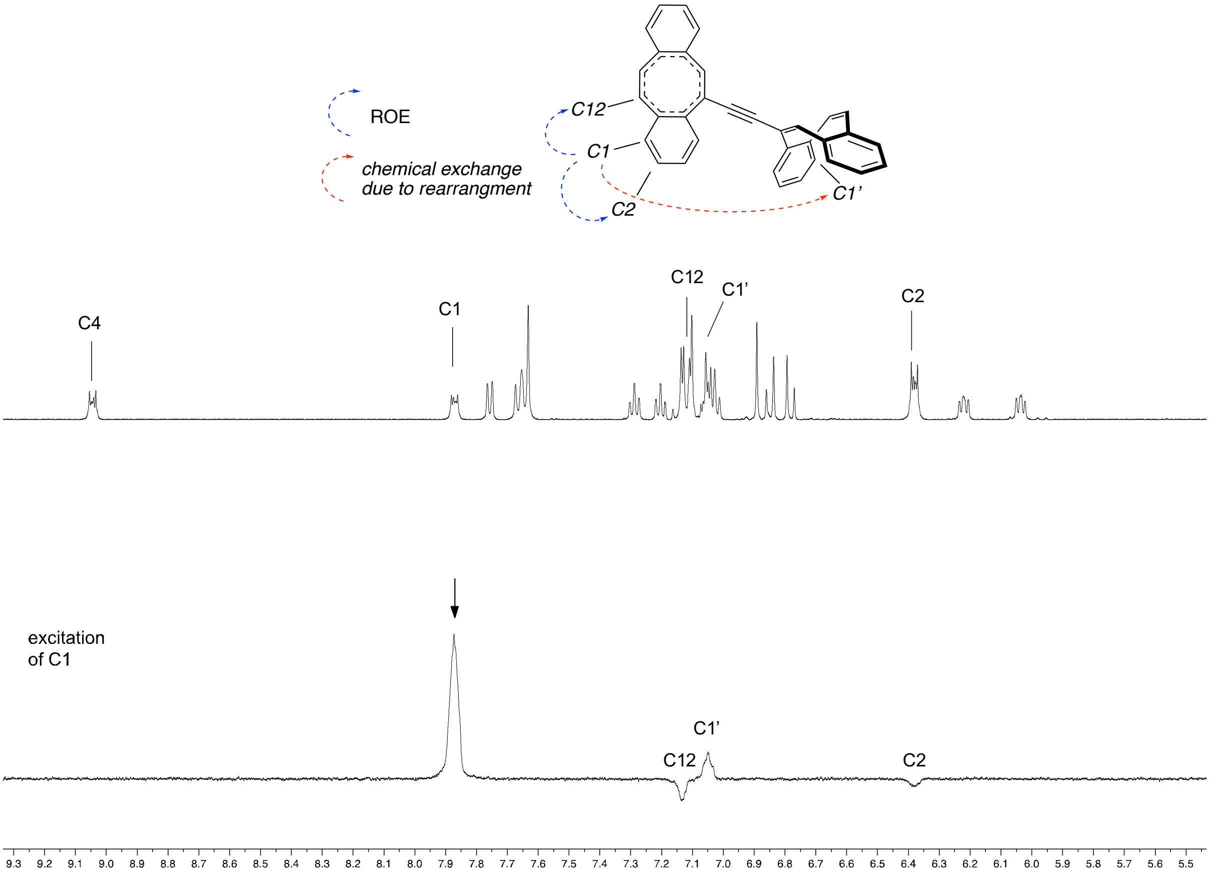

Supplementary Figure 17. Diagnostic ROE observed in the reduced dbCOT unit of K_2dbCOT2. Spectra recorded at 500 MHz in THF-d_8 at T = 248.0 K, mixing time 200 ms. The arrow indicates selective excitation of *H*–(C1) at 7.87 ppm.

Fully reduced structures: The planar structure of the terminal dbCOT units in K_6dbCOT3 was confirmed by a pronounced through-space ROE between *H*–(C1) and *H*–(C12) (Supplementary Figure 18). The same type of ROE was seen from *H*–(C6) to *H*–(C7). The ROEs between *H*–C4' and H–C4, and H–C4' and C6 indicate a propensity for adopting a globally planar structure wherein all dbCOT units are aligned in one plane and the π-system extends through the entirety of the structure. K_4dbCOT$_2$ was assigned similarly.

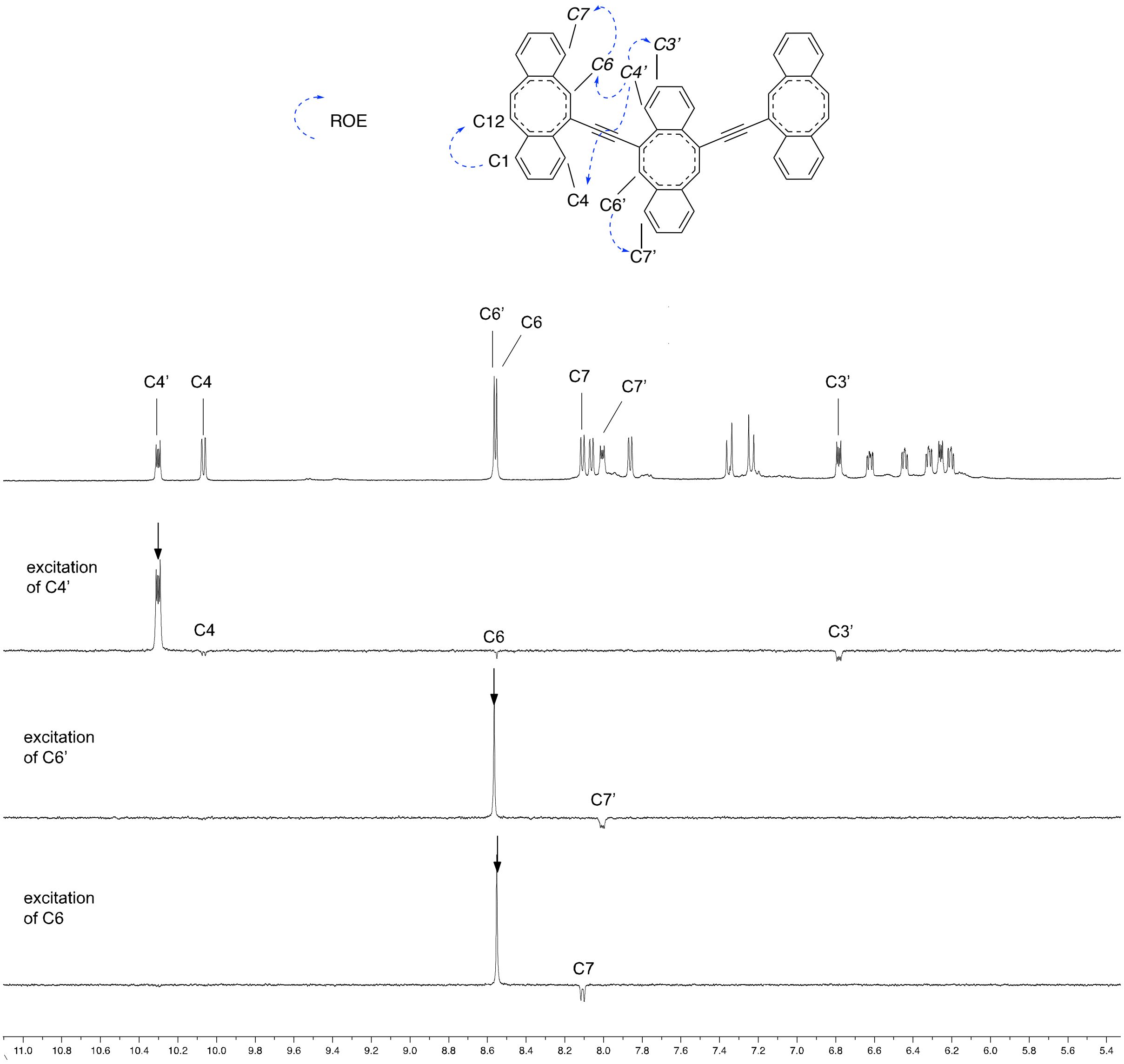

Supplementary Figure 18. Diagnostic ROE observed in K_6dbCOT3. Spectra recorded at 500 MHz in THF-d_8 at *T* = 268.2 K, mixing time = 400 ms. Top: Selective excitation of *H*–(C4') (δ = 10.31 ppm); Middle: *H*–(C6') (δ = 8.57 ppm); Bottom: *H*–(C6) (δ = 8.55 ppm).

VI. Electron count in reduced oligomers

Titration of K_4dbCOT2 with dbCOT. A solution of K_4dbCOT2 was prepared following the standard procedure: dbCOT2 (1.5 mg, 3.5 μmol) was dissolved in THF-d_8 (0.40 mL) in a Young's NMR tube and reduced. A solution of dbCOT (0.69 mg, 3.4 μmol) in THF-d_8 was then added in three 50 μL portions (~1.0 equiv.) under rigorous exclusion of air and moisture. The resulting mixture was analyzed by quantitative ^{1}H NMR spectroscopy (d1 = 30 s; T = 273.2 K) and showed a clean conversion into K_2dbCOT2 and the known K_2dbCOT (Supplementary Figure 19). Integration of the resonances at 9.08 ppm (*H*–(C4) in K_2dbCOT2) and 7.87 ppm (corresponding to 4H in K_2dbCOT) showed a ratio between the two species: K_2dbCOT2 to K_2dbCOT = 48:52. Formation of precisely one equivalent of K_2dbCOT confirms that K_4dbCOT2 is reduced with two electrons more than K_2dbCOT2.

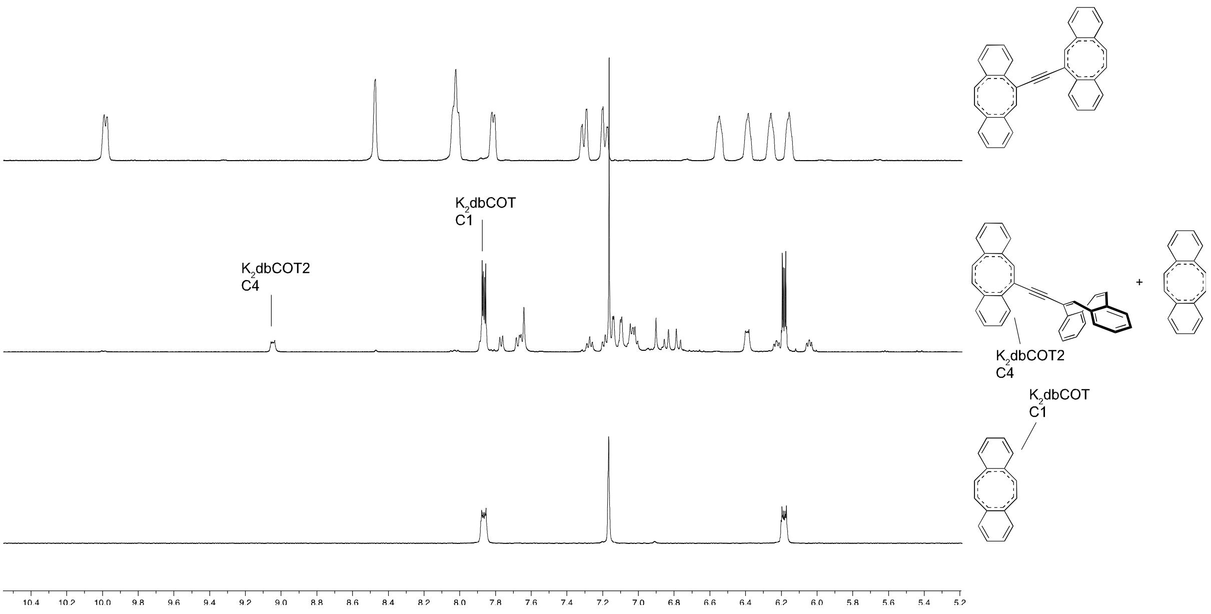

Supplementary Figure 19. Titration of K_4dbCOT2 with dbCOT. Top: ^{1}H NMR spectrum of K_4dbCOT2; Centre: quantitative ^{1}H NMR spectrum after addition of 0.96 equiv. of dbCOT to a solution of K_4dbCOT2; Bottom: ^{1}H NMR spectrum of K_2dbCOT.

VII. Electron paramagnetic resonance (EPR) characterization

Samples were prepared following the standard stop-and-go method. Solution X-band EPR spectra were recorded on liquid samples at RT as prepared in Young-tubes. Data were acquired on a Bruker Elexsys E500 spectrometer equipped with a Bruker SUPER-X CW-EPR bridge, a

Bruker ER 4116 DM dual mode cavity, and an EIP 538B frequency counter at ambient temperature. Modulation amplitudes were tested in the range 0.1-8 G and microwave power was tested between 0.2 and 200 mW. All well-defined stages of reduction for dbCOT2 and dbCOT3 (confirmed by ^{1}H NMR spectroscopy), as well as stages between these were investigated, but all samples were EPR silent, putting an upper limit on the concentration of odd-electron species of approx. 0.1 μM.

The concentration of each oligomer respectively was: dbCOT2: 5.0 mM; dbCOT3: 1.5 mM.

VIII. Redox cycles of dbCOT, dbCOT2, and dbCOT3.

General method for oxidation/reduction cycles of dbCOT-oligomers with K/DDQ. Under argon atmosphere in a glovebox, solutions of reduced dbCOT-oligomers prepared according to the general reductive method was treated with a stock solution of DDQ (2,3-dichloro-5,6-dicyano-1,4-benzoquinone) in dry and oxygen-free THF-d_8 via a gas-tight syringe. The solution was carefully vortexed (to prevent contact with the reactive metal) for 20 seconds, and exported out of the glovebox for NMR analysis. The procedure was iteratively repeated.

Titration of K_2dbCOT with DDQ. K_2dbCOT was prepared according to the general method by reacting dbCOT (0.55 mg, 2.7 μmol) with potassium metal (excess). The solution was treated stepwise with 20 μL aliquots of a stock solution of DDQ (1.2 mg, 5.2 μmol) in dry THF-d_8 (0.60 mL), until dbCOT was completely reformed as seen by ^{1}H NMR spectroscopy (Supplementary Figure 20).

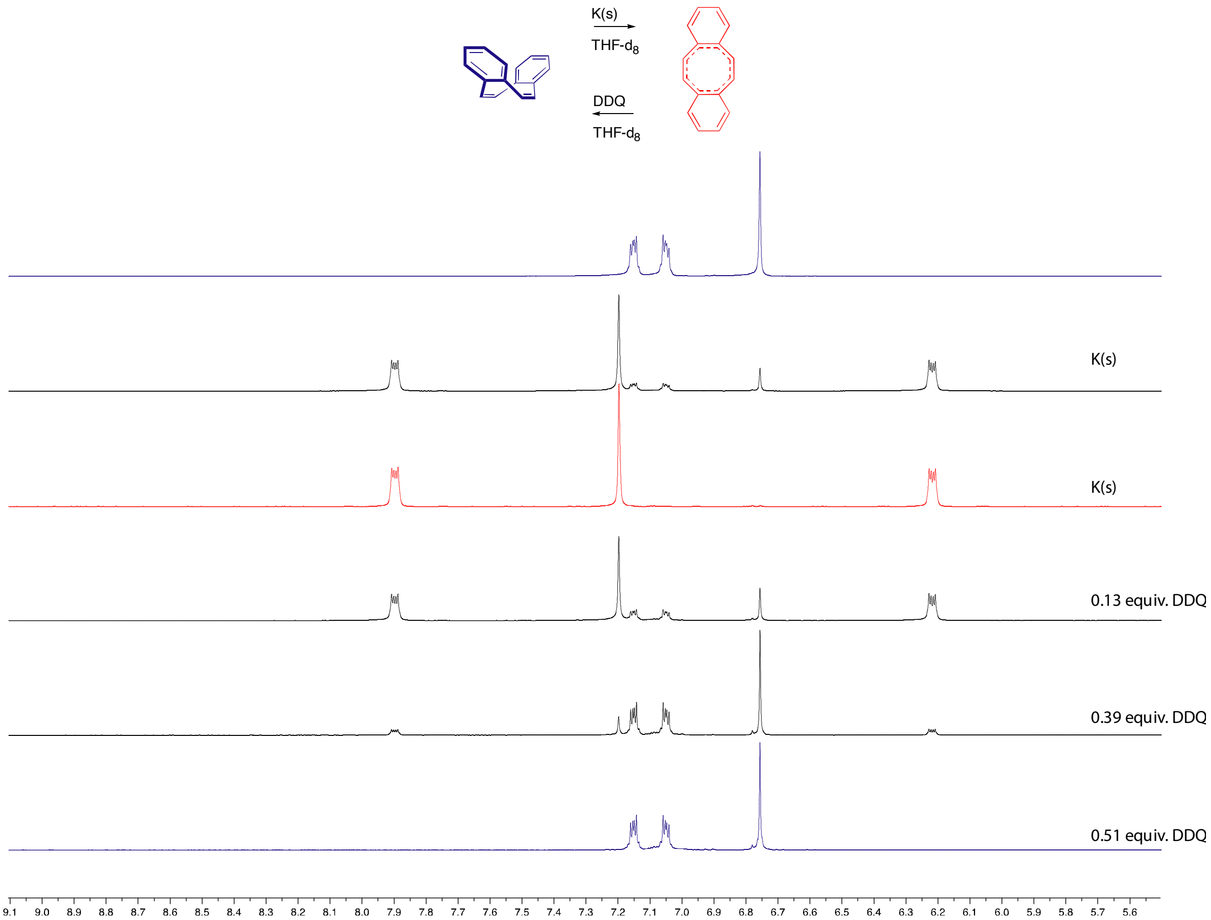

Supplementary Figure 20. Reduction-oxidation cycle of dbCOT. ^{1}H NMR spectra were recorded at T = 273.0 K.

Titration of K_4dbCOT2 with DDQ. K_4dbCOT2 was prepared according to the general method by reacting dbCOT2 (0.58 mg, 1.3 µmol) with potassium (excess) metal in THF-d_8 (0.45 mL). The solution was treated stepwise with 20 µL aliquots of a stock solution of DDQ (1.5 mg, 6.5 µmol) in dry THF-d_8 (0.50 mL), until dbCOT2 was completely reformed as seen by ^{1}H NMR spectroscopy (Supplementary Figure 21). The procedure could be repeated up to three cycles: Following the described cycle, K_2dbCOT2 was reformed by re-exposing the mixture to the potassium metal followed by addition of DDQ (Supplementary Figure 22).

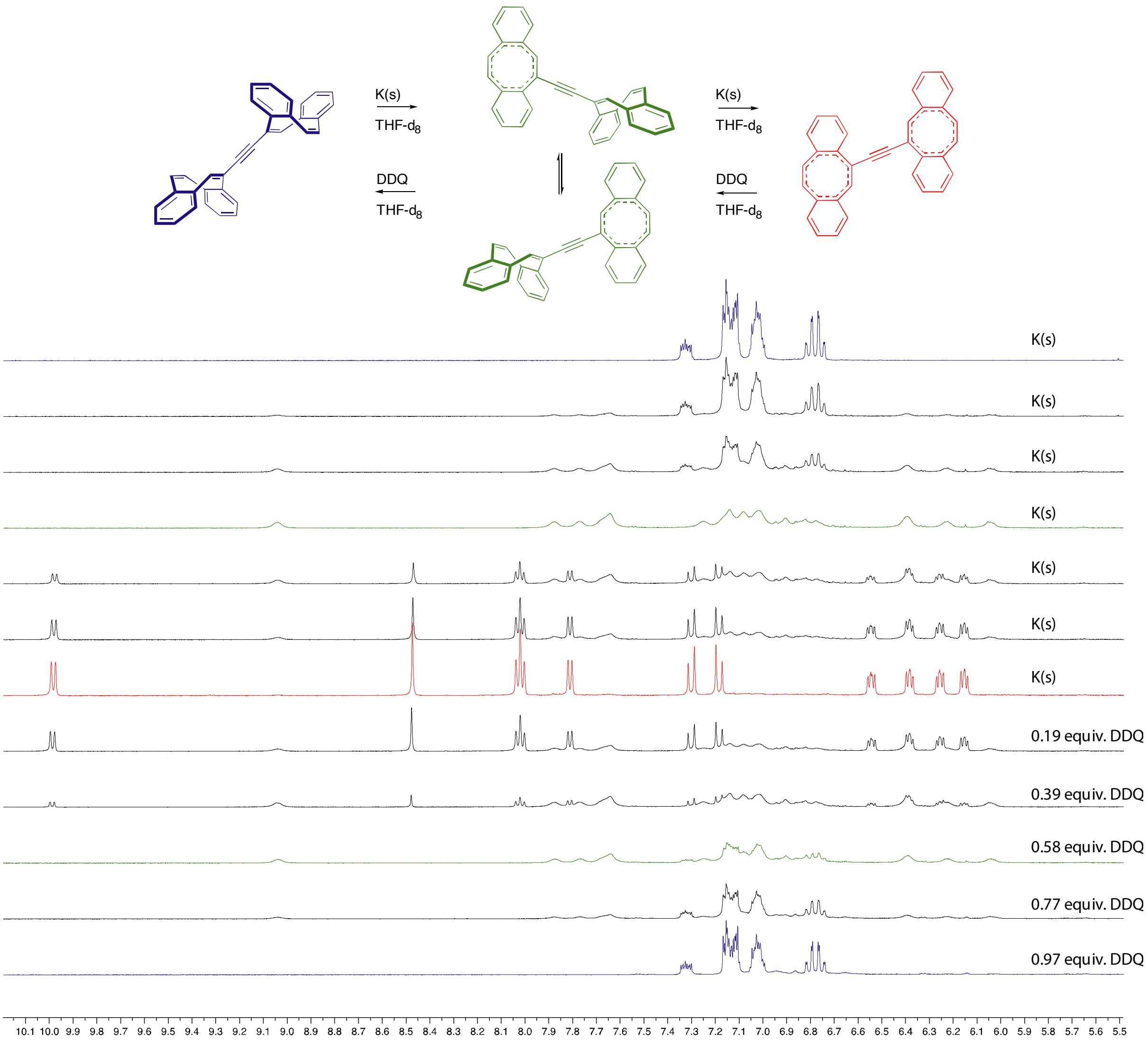

Supplementary Figure 21. Reduction-oxidation cycle of dbCOT2. Dilution of the sample reflects in reduced signal intensity.

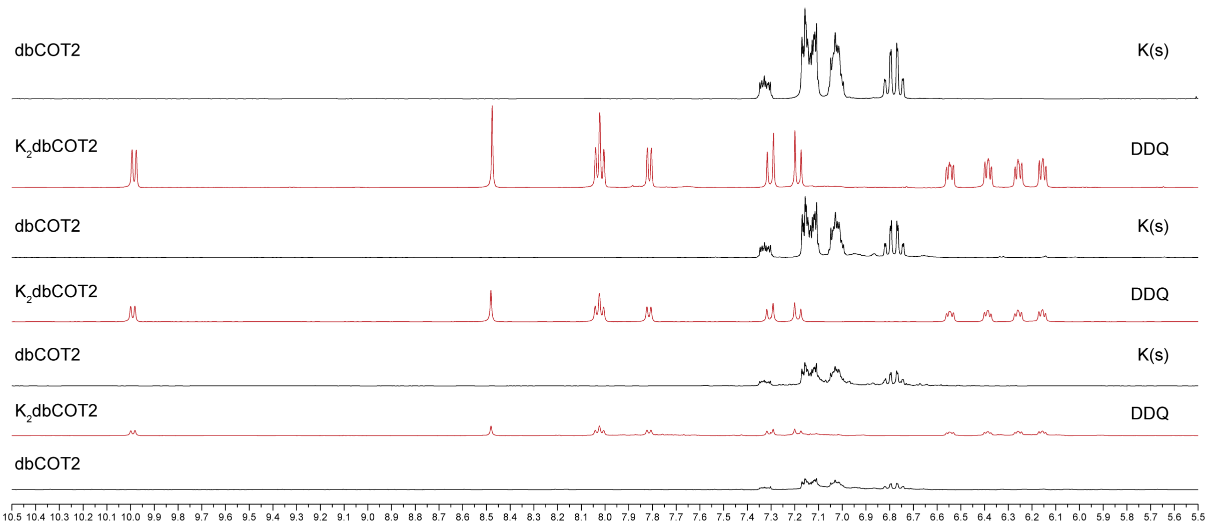

Supplementary Figure 22. Three consecutive oxidation-reduction cycles of dbCOT2. Dilution of the sample reflects in reduced signal intensity.

Titration of K_6dbCOT3 with DDQ. K_6dbCOT3 was prepared according to the general method by reacting dbCOT3 (0.69 mg, 1.1 μmol) with potassium (excess) metal in THF-d_8 (0.45 mL). The solution was treated stepwise with 20 μL aliquots of a stock solution of DDQ (1.2 mg, 5.2 μmol) in dry THF-d_8 (0.60 mL), until dbCOT3 was completely reformed as seen by ^{1}H NMR spectroscopy (Supplementary Figure 23).

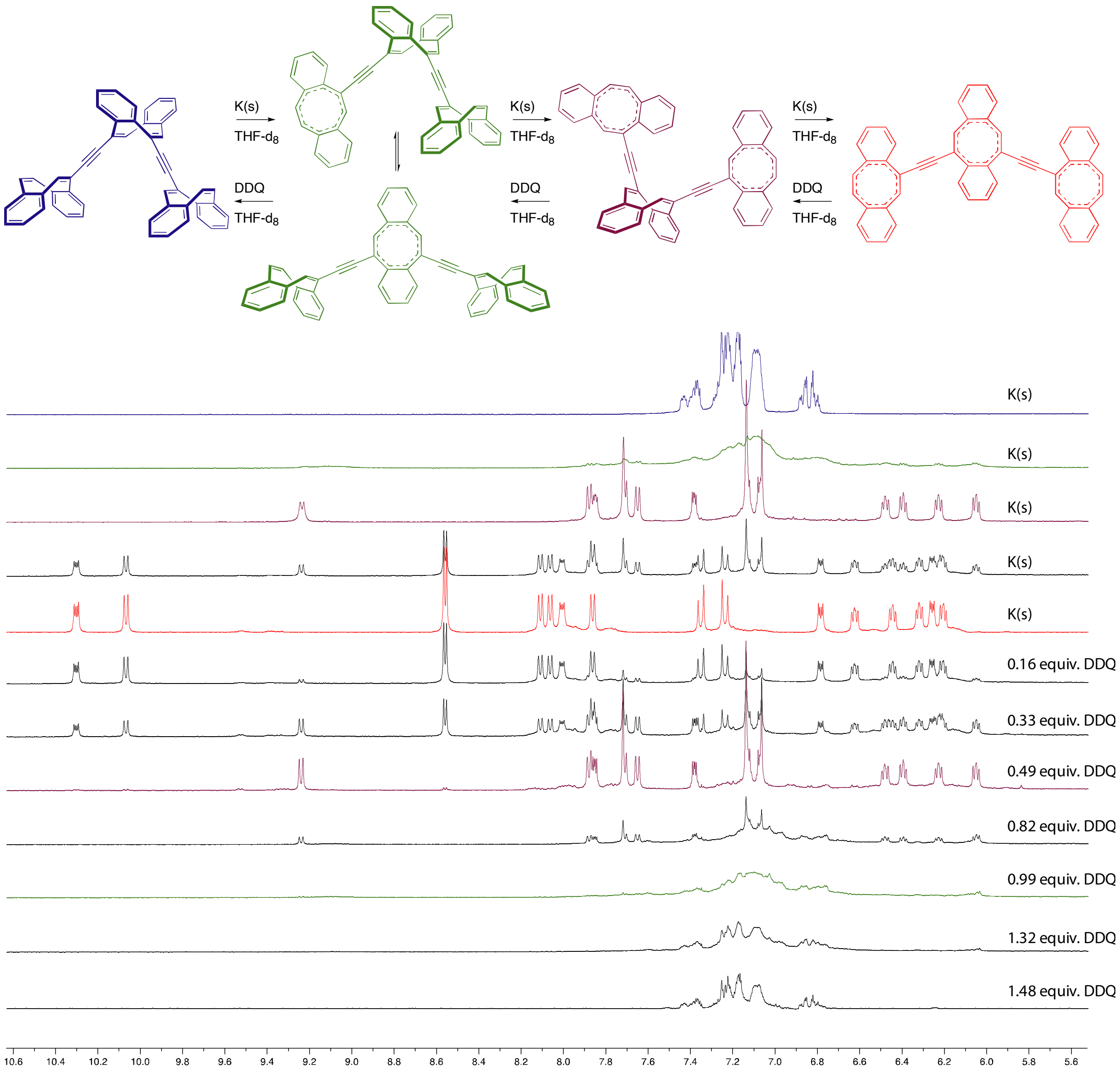

Supplementary Figure 23. Reduction-oxidation cycle of dbCOT3. ^{1}H NMR spectra were recorded at $T = 273.0$ K. Dilution of the sample reflects in reduced signal intensity.

IX. NMR characterization of intramolecular electron transfer.

NMR characterization of electron transfer in K_2dbCOT2 and K_2dbCOT3.

General procedure for determination of rate constants. Samples of K_2dbCOT2 and K_2dbCOT3 in Young's NMR tubes were prepared following the general procedure. 1D chemical exchange NMR data were obtained with the Double Pulse Field Gradient Spin Echo Nuclear Overhauser Enhancement (DPFGSE NOE) experiment[4] at variable mixing times from 25 – 200 ms, using a pulse sequence written in-house. The NMR spectrometer probe temperature was calibrated in the temperature range of 237.0 – 329.9 K using an NMR thermometer method based on

chemical shift difference in neat MeOH.[5] The build-up curve for the exchange peak was determined by plotting the integral ratio of the exchange peak to the inverted peak, the so called PANIC approach.[6] The rate constant of the exchange process is then given by the slope of the linear build-up curve, using only the data points for mixing times within the linear initial rate approximation.

Free energy barrier of charge transfer in K_2dbCOT2. Selective inversion of *H*–(C4) (δ = 9.08 ppm) gave rise to an exchange peak at 7.80 ppm. Spectra with 50, 75, 100, 200 and 300 ms mixing time were collected at the following temperatures: 239.0, 244.9, 250.7, 256.5, 262.3, and 268.2 K. The rate constants of the exchange process at different temperatures were determined as described above. The thermodynamic parameters were determined from Eyring's equation, and by linear fitting of rate constants at different temperatures in an Eyring plot.

Supplementary Table 9. Free energy barrier of charge transfer within K_2dbCOT2[a]

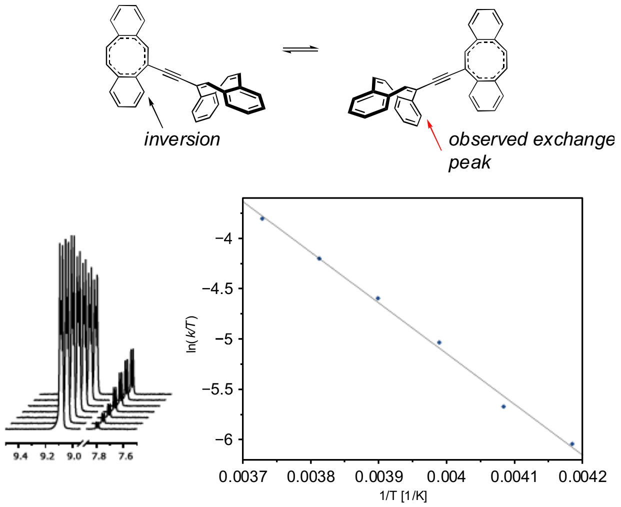

$\Delta G^{\ddagger}_{210K}$ (kcal/mol)$^{-1}$	$\Delta H^{\ddagger}$ (kcal/mol)$^{-1}$	$\Delta S^{\ddagger}$ (cal/mol K)$^{-1}$
13.7	10.0 ± 0.35 (SD)	-17.4 ± 1.37 (SD)

[a] Black arrow highlights the selectively inverted position. Red arrow indicates the position of the proton showing chemical exchange. Left: Representative stack of spectra with varying mixing times. Inversion at δ = 9.08 ppm and *T* = 268.2 K. Right: Eyring plot.

Free energy difference between isomers 7a/c and 7b of K_2dbCOT3 at 209.8 K. The relative concentrations of **7a/c** and **7b** at 209.8 K in THF-d_8 was measured by integration of the resonance from *H*–(C4)–**7a/c** (δ = 9.16 ppm) and 2*H*–(C4')–**7b** (δ = 9.08 ppm) in the ^{1}H NMR spectrum: **7a/c** to **7b** = 47:53.

Free energy barriers of charge transfer in K_2dbCOT3. Selective inversion of *H*–(C4a) (δ = 9.16 ppm) at 209.8 K gave rise to a strong exchange peak at δ = 7.72 ppm and a weak exchange peak at δ = 7.39 ppm (Supplementary Figure 24). This can be understood as electron relay from a terminal dbCOT unit to the centre unit and further on to the second terminal unit. The free energy barrier of the exchange process was derived from the rate constant using the Eyring equation: $\Delta G^{\ddagger}_{209.8\mathrm{K}} = 11.6 \pm 0.96$ kJ/mol^{-1}.

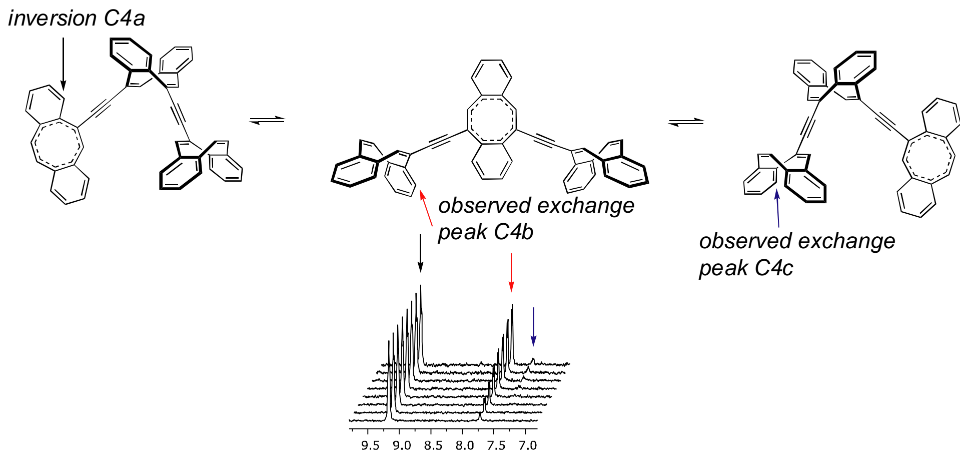

Supplementary Figure 24. Chemical exchange from the proton at C4a to C4b and to C4c in K_2dbCOT3.

Inversion of 2*H*–(C4b'/C1a') (δ = 9.08 ppm) at 209.8 K gave rise to a pair of resonances of equal size at δ = 7.79 ppm and 7.40 ppm, respectively. This can be understood as electron relay from the centre dbCOT unit to either terminal group. Spectra were collected using 25, 50, 75, 100, 125, and 150 ms mixing time (Supplementary Figure 25). The free energy barrier of the exchange process was derived using the Eyring equation: $\Delta G^{\ddagger}_{209.8\mathrm{K}} = 11.7 \pm 1.15$ (SD) kJ/mol^{-1}.

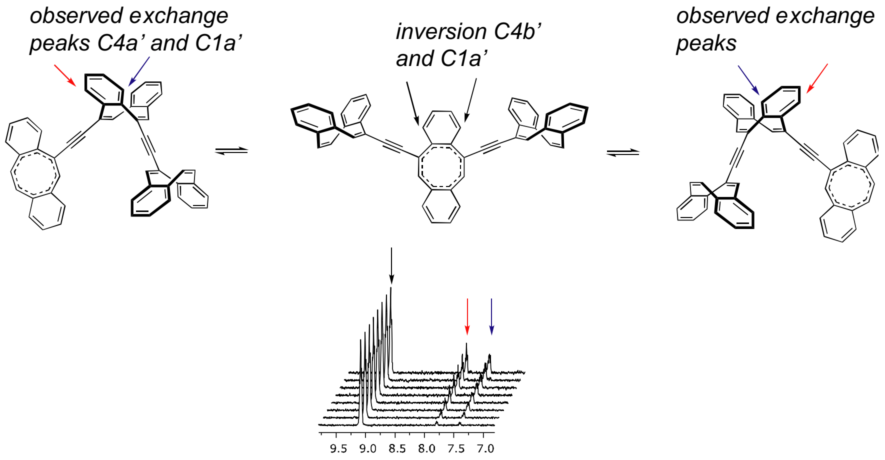

Supplementary Figure 25. Chemical exchange from the proton at C4b' to C4a' and C1b' to C1a' in K_2dbCOT3. C4b'and C1b' are pseudo-homotopic.

Concentration dependence of electron relay kinetics. K_2dbCOT2 (3.02 mM in 500 μL THF-d_8) was prepared in a Young's NMR tube following the general procedure. A DPFGSE NOE experiment with selective inversion at 9.08 ppm using 100 ms mixing time was performed at 268.0 K as described above. The sample was then diluted by addition of THF-d_8 in 100 μL aliquots to a final concentration of 1.51 mM. The ratio of integrals at 9.08 ppm and 7.80 ppm was not influenced over this concentration range (mean integral ratio = 2.55 ± 0.075 (SD)) showing that the observed electron relay follows zero order kinetics in K_2dbCOT2 and thus that any intermolecular contribution is small (Supplementary Figure 26).

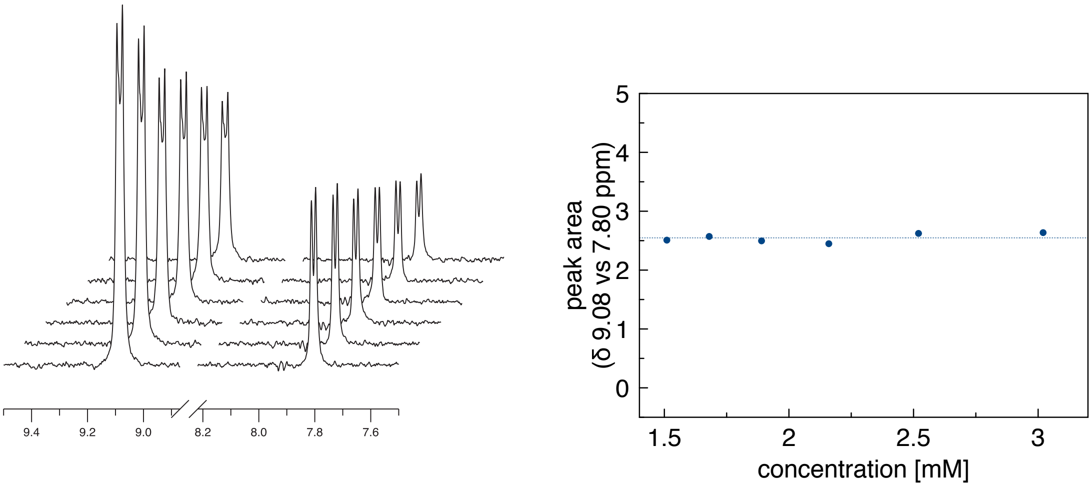

Supplementary Figure 26. Concentration dependence of charge relay in K_2dbCOT2. Left: Stack of chemical exchange spectra with concentrations from 3.02 mM to 1.51 mM; Right: Ratio between peak integrals as a function of concentration. Blue dotted line shows the average ratio of peak areas.

X. Cyclic voltammetry, differential pulse voltammetry, and anodic voltammetry stripping characterization

General: Cyclic voltammetry (CV), differential pulse voltammetry (DPV) and anodic stripping voltammetry (ASV) measurements were recorded using a three electrode potentiostat (Palmsens4 (2018), Palmsens BV.). The experiments were performed in glass cells that were predried at 135 °C overnight and cooled under a stream of argon. Experiments were performed in THF (dried over sodium-potassium alloy, vacuum distilled, and then purged with argon) under an argon atmosphere and at room temperature. The working electrode was mechanically polished using 0.05 μm alumina powder on a soft lapping pad until a glossy finish was obtained.

The polished electrode was further washed and sonicated (10 s), first in ultrapure water, and then in dry CH_2Cl_2, before drying in air.

Working electrode: Polycrystalline gold disc (d = 2 mm) from Palmsens BV (ItalSens).

Reference electrode: Silver wire pseudo-reference immersed in THF containing Bu_4NPF_6 (0.10 M) and $AgNO_3$ (sat.).

Counter electrode: Platinum wire.

Representative procedure for CV, DPV, and ASV measurements. To an electrochemical cell was added a solution of Bu_4NPF_6 (0.10 M) in THF (2.0 mL). Prior to each data collection, the cell was conditioned by four cycles of cyclic voltammetry (CV) at 0.40 V/s from 0.40 V to -3.00 V. The mixture was then gently vortexed and another four CV cycles were performed.

CV blank. CV data was recorded using the following parameters: E(begin) = -2.00 V; E(vertex1) = -3.40 V; E(vertex2) = -2.00 V; scan rate = 0.025 V/s; number of scans = 3.

DPV and ASV blank. DPV data was recorded from -2.00 V to -3.40 V using the following parameters: E(step) = 0.005 V; scan rate = -0.025 V/s; E(pulse) = 0.025 V; t(pulse) = 0.07 s. Electrochemical deposition prior to ASV measurements was performed at -3.40 V for 3 s followed by ASV and data was recorded from -3.40 V to -2.00 V using the following parameters: E(step) = 0.005 V; scan rate = 0.025 V/s; E(pulse) = 0.025 V; t(pulse) = 0.07 s.

Full CV. A solution of dbCOT (1.0 mL, 4.0 mM in THF), dbCOT2 (1.0 mL, 4.0 mM in THF), dbCOT3 (1.0 mL, 3.0 mM in THF), or dbCOT5 (1.0 mL, 1.8 mM in THF) was added to the cell. CV data was then recorded using the following parameters: E(begin) = -2.00 V; E(vertex1) = -3.40 V; E(vertex2) = -2.00 V; scan rate = 0.025 V/s; number of scans = 3.

Partial CV. CV data was then recorded over the first two redox processes using the following parameters: scan rate = 0.025 V/s; number of scans = 3. Potential windows:

- for dbCOT: E(begin) = -2.30 V; E(vertex1) = -3.20 V; E(vertex2) = -2.30 V;
- for dbCOT2: E(begin) = -2.20 V; E(vertex1) = -2.90 V; E(vertex2) = -2.20 V;
- for dbCOT3: E(begin) = -2.20 V; E(vertex1) = -2.63; E(vertex2) = -2.20 V;
- for dbCOT5: E(begin) = -2.05 V; E(vertex1) = -2.65; E(vertex2) = -2.05 V.

Partial DPV. DPV data was then recorded over the first two redox processes using the following parameters: E(step) = 0.005 V; scan rate = 0.025 V/s; E(pulse) = 0.025 V; t(pulse) = 0.07 s. Potential windows:

- for dbCOT: E(begin) = -2.10 V; E(end) = -3.15 V;
- for dbCOT2: E(begin) = -2.10 V; E(end) = -2.70 V;
- for dbCOT3: E(begin) = -2.10 V; E(end) = -2.55 V;
- for dbCOT5: E(begin) = -2.10 V; E(end) = -2.50 V.

Partial ASV. After collecting DPV data, electrochemical deposition was performed at E(begin) for 3 s followed by ASV and data was recorded using the following parameters: E(step) = 0.005 V; scan rate = 0.025 V/s; E(pulse) = 0.025 V; t(pulse) = 0.07 s. Potential windows:

- for dbCOT: E(begin) = -3.15 V; E(end) = -2.10 V;
- for dbCOT2: E(begin) = -2.70 V; E(end) = -2.10 V;
- for dbCOT3: E(begin) = -2.55 V; E(end) = -2.10 V;
- for dbCOT5: E(begin) = -2.50 V; E(end) = -2.10 V.

Reference to ferrocene. A solution of ferrocene (1.0 mL, 4.0 mM in THF) was added to the cell. CV data was then recorded using the following parameters: E(begin) = -0.30 V; E(vertex1) = +0.30 V; E(vertex2) = -0.30 V; scan rate = 0.025 V/s; number of scans = 3. DPV data was then recorded from +0.30 V to -0.30 V using the following parameters: E(step) = 0.005 V; scan rate = 0.025 V/s; E(pulse) = 0.025 V; t(pulse) = 0.07 s. Electrochemical deposition was performed at -0.30 V for 3 s followed by ASV and data was recorded from -0.30 V to +0.30 V using the following parameters: E(step) = 0.005 V; scan rate = 0.025 V/s; E(pulse) = 0.025 V; t(pulse) = 0.07 s.

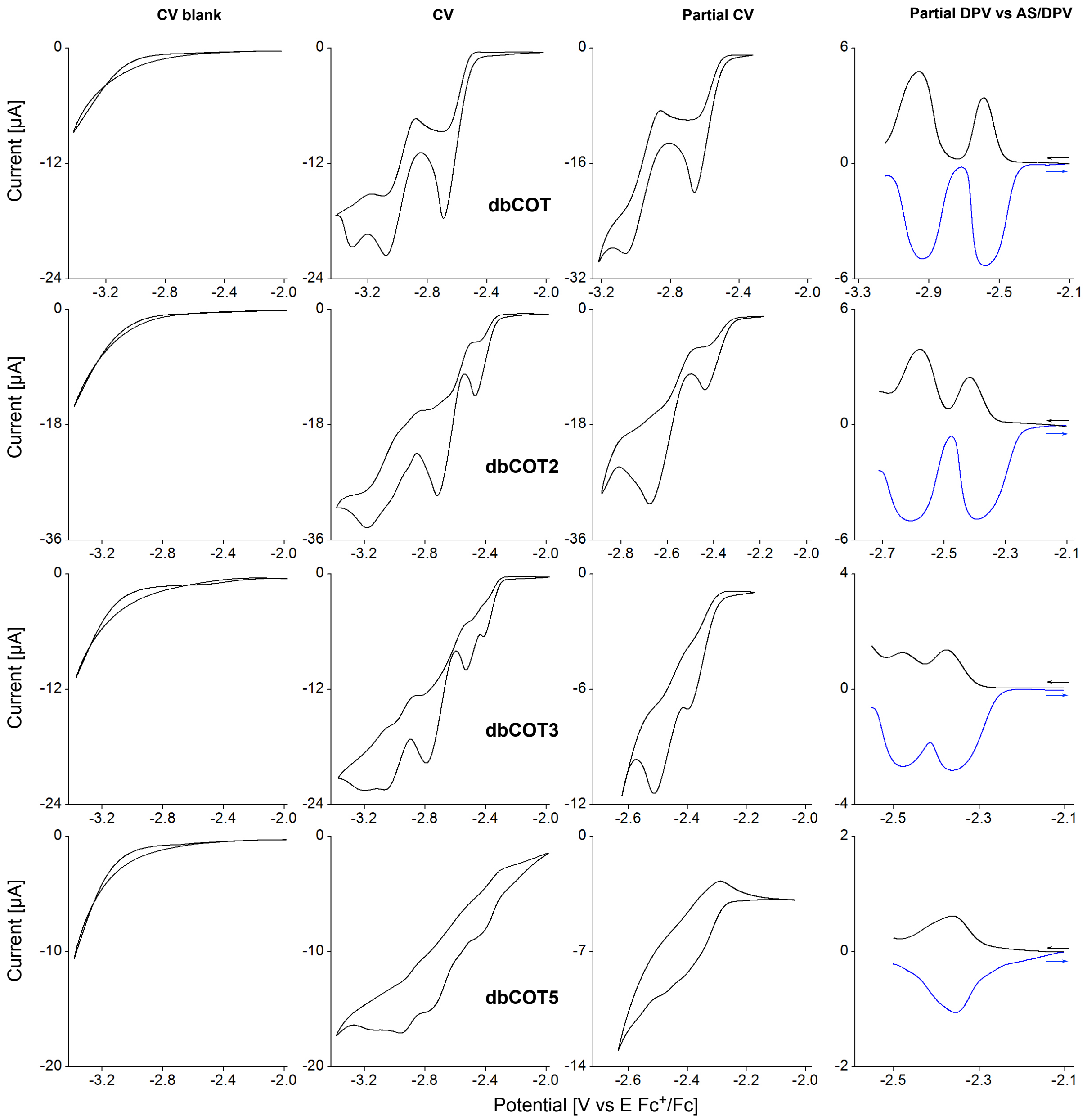

Supplementary Figure 27. Cyclic voltammetry and differential pulse voltammetry characterization of dbCOT, dbCOT2, dbCOT3 and dbCOT5. Several experiments were performed for each and representative datasets are shown.

Supplementary Table 10. CV, DPV, and ASV peak potentials for the first two waves. [a]

	Method	Peak potentials (observed) (V)	Integral (µAV)
dbCOT	CV	-2.66, -3.06	0.968, 0.961
	DPV	-2.58, -2.94	
	ASV	-2.59, -2.95	
dbCOT2	CV	-2.44, -2.68	0.710, 1.47
	DPV	-2.40, -2.61	
	ASV	-2.42, -2.58	
dbCOT3	CV	-2,40, -2.51	0.166, 0.170
	DPV	-2.36, -2.48	
	ASV	-2.37, -2.48	
dbCOT5	CV	-2.40, -2.48	Insufficiently resolved.
	DPV	-2.36, -2.39 (partially resolved)	
	ASV	-2.36, br	

[a] Observed peak potentials listed. Potentials are referenced to the ferrocene peak potential (0.00 V). Experimentally, -0.14 V was measured versus the Ag│$AgNO_3$ pseudo-reference electrode. Integration was done using the integration function in PSTrace V5.8. The area under the wave is assumed to be proportional to the number of electrons.

XI. Spectroelectrochemical analysis

General: Spectroelectrochemical measurements were performed using a Palmsens4 (2018) Palmsens BV and custom-made quartz cells (Herasil®) that were pre-dried at 135 °C overnight and cooled under a stream of argon. Experiments were performed in dry THF (dried to a persistent blue color over sodium-potassium alloy and then vacuum distilled) under an argon atmosphere and at room temperature. The light from an incandescent lamp was collimated by a quartz lens and directed with a UV/enhanced aluminum mirror onto the polished working electrode (see Section *General, Section X,* for polishing procedure). The specular reflection from the gold working electrode was collected with a broadband hollow-core fiber and guided into an Avantes BV AvaSpec-3648 spectrophotometer. In this configuration, absorbance changes are recorded in reflective mode with the beam passing twice through the thin interaction region

at the gold working electrode (Supplementary Figure 28). The bottom of the custom-made quartz cell was made from a flat quartz plate with optical quality.

Working electrode: Polycrystalline gold disc (d = 2 mm) from Palmsens BV (ItalSens).

Reference electrode: Silver wire pseudo-reference immersed in THF containing Bu_4NPF_6 (0.10 M) and $AgNO_3$ (sat.).

Counter electrode: Platinum wire.

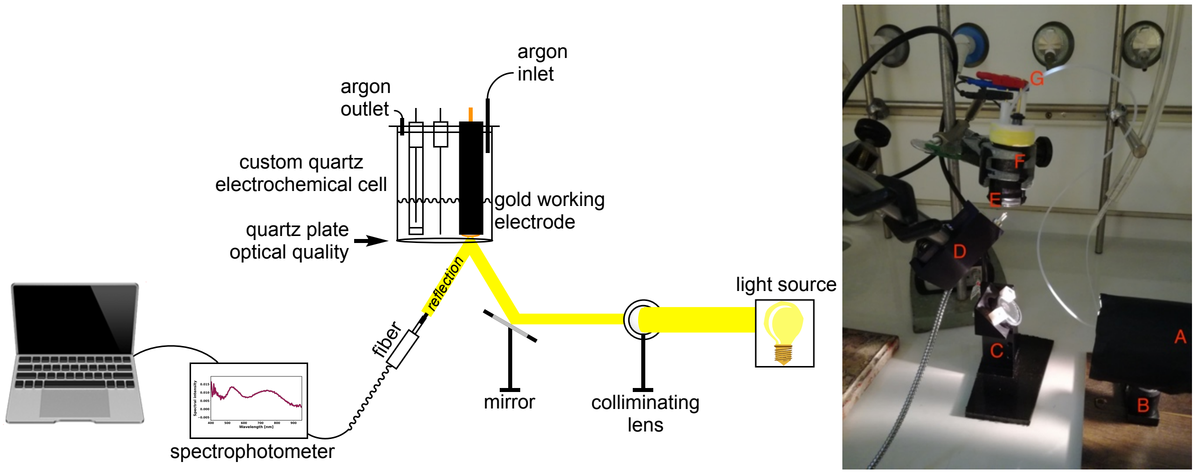

Supplementary Figure 28. Left: Schematic representation of the spectroelectrochemical setup; Right: Photograph of a typical experiment setup: A - light source; B - collimating lens; C – mirror; D - fiber connected to the spectrophotometer; E - gold working electrode; F - quartz electrochemical cell; G - argon inlet.

Representative procedure for spectroelectrochemical measurements. To an electrochemical cell was added a freshly prepared solution of Bu_4NPF_6 (0.10 M) in THF (2.0 mL). Prior to each data collection, the cell was conditioned by four cycles of cyclic voltammetry (CV) at 0.40 V/s from 0.40 V to -3.00 V. The mixture was then gently vortexed and another four CV cycles were performed. The light source and spectrophotometer were aligned and a blank UV-Vis spectrum recorded, after which the optical system was not altered. Appropriate scan widths were estimated from Supplementary Table 10.

CV blank. CV data was recorded using the following parameters: E(begin) = -2.00 V; E(vertex1) = -3.40 V; E(vertex2) = -2.00 V; scan rate = 0.025 V/s; number of scans = 4.

CV. A solution of dbCOT2 (1.0 mL, 4.0 mM in THF), dbCOT3 (1.0 mL, 3.0 mM in THF), or dbCOT5 (1.0 mL, 1.8 mM in THF) was added to the cell. The series of CV data was then recorded using the following parameters: scan rate = 0.025 V/s; number of scans = various.

During the CV measurements, the UV-Vis spectra was recorded in the reflective mode every 200 ms.

dbCOT2, scanning over the first two peaks: E(begin) = -2.30 V; E(vertex1) = -3.00 V; E(vertex2) = -2.30 V.

dbCOT3, scanning over the first two peaks: E(begin) = -2.20 V; E(vertex1) = -2.80; E(vertex2) = -2.20 V.

dbCOT5, scanning over the first two peaks: E(begin) = -2.25 V; E(vertex1) = -2.65 V; E(vertex2) = -2.25 V.

Reference to ferrocene. After the respective cycling experiment, a solution of ferrocene (1.0 mL, 4.0 mM in THF) was added to the cell. CV data was then recorded using the following parameters: E(begin) = -0.30 V; E(vertex1) = +0.30 V; E(vertex2) = -0.30 V; scan rate = 0.025 V/s; number of scans = 3.

Data processing procedure for spectroelectrochemical measurements. After the respective measurement, both the electrochemical data and the collected absorbance frames were exported as asci-files for further processing in a custom developed python analysis package. The two datasets were correlated by relating the repeated pattern of the CV data to the repeating pattern of the optical variations as outlined below. The correlation requires only small adjustments. Typically, accumulation of the optical spectra was started a few seconds before the start of the first CV cycle and ended a few seconds after the finish of the last. The start and end time/points, as well as the actual time-period between each collected spectral frame were then adjusted to overlap both experiments. These three parameters were initially set manually and then optimized during a Global analysis outlined below and in Supplementary Figure 29.

The optical spectra were assembled into a 2D matrix with wavelength and frames as the axes. For Figure 4A-C, the spectral axis was re-binned into 10 nm bins, background-subtracted, and presented in the heat map. Selected spectral slices of 20 nm width are shown in the middle panel to emphasize the correlation to the CV data. The y-axis of the measured CV current in the left panel is adjusted to compensate for the difference in start and stop time of the measurements. A typical mismatch between the measurements is of the order of 2 s at the beginning and end for a typical total accumulation time of 500 s. The extracted spectrum above the heat map was generated by taking the mean of all spectra, in which the maximum observed difference spectrum reached 2/3 of the overall observed maximum change.

For de-convolution of the two components in Figure 4D, we followed the workflow illustrated in Supplementary Figure 29. First, the measurement for dbCOT3 was separated into two intervals. After each of the two measurements, a model was formed consisting of a modulating species and a background.

The modulating species was gained by using the integrated current of the CV curve (compensated for a linear ohmic fraction) and convoluting this current with an exponential decay. The concentration of the main component is thus the currently converted fraction of the molecules (the measured current). The experimentally found decay parameters are of the order of 1-2 frames (approximately 200 ms). The scale of the CV measurements and the measured spectral frames were correlated by estimating the different start and endpoints as well as the correlation between the measured CV data points, frames, and refining the parameter in a Global analysis scheme similar to transient optical absorption.[7, 8] In Global analysis, a parameter-based model, here created by shifting the time parameters of the measured current versus the frames of the spectra, is compared against the measured data and an optimum spectrum is extracted for each step of the optimization. The modeled matrix is then compared against the measured matrix and the parameters are chosen to minimize the difference. The extracted spectra represent the spectra associated with the process. As we measured the difference spectra while reducing and oxidizing dbCOT3 during the CV and used as the model a number representing the total amount of converted molecules (the measured current), the extracted spectrum represents the species formed during oxidation and reduction. Here the spectrum for the first and second reduction step, respectively, for the two voltage regions in which the scan was performed.

In a third and independent measurement, we again collected a CV with simultaneous UV-vis spectra over a wider range, encompassing both reduction steps. In the following analysis, we optimized each measured spectrum with a linear combination from the two spectra representing the first and second reduction steps. In this optimization, $\alpha*\beta$ are fit-factors while the spectra denoted x and y are fixed. The pre-factor α is allowed to vary between 0 and 10, the ratio β are fit-factors and are allowed to vary between 0 and 1. The fractions presented in Figure 4D are the factors $\alpha*\beta$ and $\alpha*(1-\beta)$ respectively, representing the fractions of the first and second reduced spectra in the measured spectrum. As the total factor (α) was not limited to 1 a total fraction > 1 would have been possible but was not observed. This suggests that the combination of mass-transport and conversion ratio is comparable between the three independent experiments.

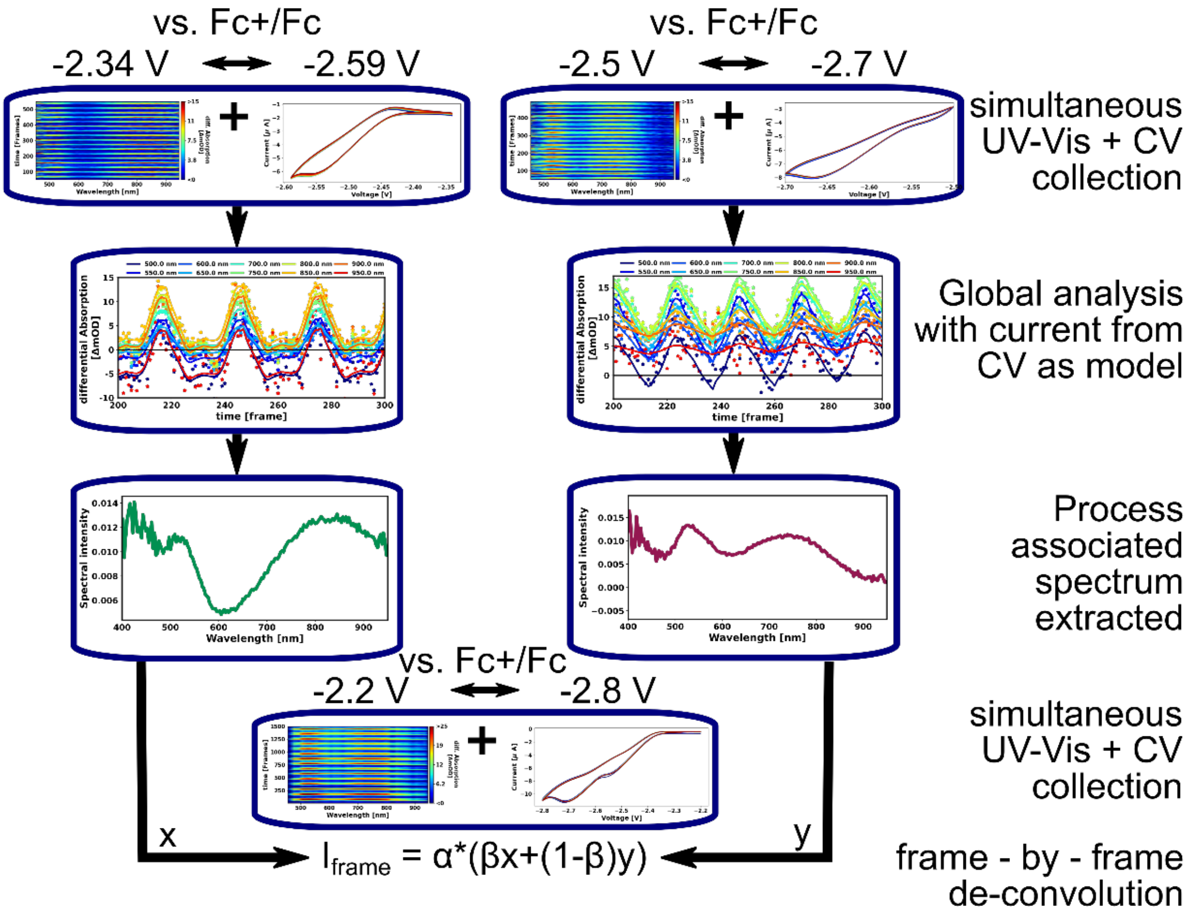

Supplementary Figure 29. Flow chart illustration the analysis procedure. Three independent data-sets are recorded. Two are modelled with Global analysis to extract Process associated spectra. The third data-set is then deconvoluted using these process associated spectra.

XII. Density functional theory calculations

Density functional theory (DFT) calculations were carried out using the standard B3LYP hybrid functional method[9] together with standard 6-311+G(d,p) all-electron split-valence plus polarization functions basis sets[10] and employing a polarizable continuum model (PCM) representation for a THF solvent environment throughout.[11] Subsequent frequency calculations were performed to validate the optimizations and provide free energies.

With formal full reduction/oxidation assignments, the investigated species can be described as $K^{+}_{2m}dbCOTn^{2m-}$ THF (PCM), where m corresponds to the specific number of $2e^{-}/2K^{+}$ reductions, and n the number of dbCOT units in the oligomer. The pairs of explicit K^{+} counter ions included per doubly reduced dbCOT unit were in all cases located with one ion on each side of the plane of the dbCOT central ring. This was consistent with suggestions from initial testing

to identify suitable starting geometries. The optimized molecular species (neutral and reduced) were subsequently used to assess calculated structural parameters and to provide information about the calculated electronic structure properties at the same level of theory. All neutral and step-by-step doubly reduced dbCOT dimers (dbCOT2) and trimers (dbCOT3) structures with associated total energies and HOMO and LUMO orbitals from the B3LYP/6-311+G(d,p) calculations are presented in Supplementary Table 11. Optimized xyz-coordinates are listed as further supplementary information in the separate coordinate file. The quantum chemical calculations were performed using the Gaussian16[12] program.

Supplementary Table 11. Selected geometry optimizations and electronic structure calculations of dbCOT dimers and trimers in different reduction states from B3LYP/6-311+G(d,p) calculations with a self-consistent reaction field polarizable continuum model of the THF sovent. Energies in Hartree (H) and molecular orbitals displayed using a standard 0.02 isovalue.

Description	Optimized geometry	HOMO	LUMO
**dbCOT2**			
Neutral dbCOT dimer.<br>E_{tot} = -1309.081330 H<br>G_{tot} = -1308.697892 H	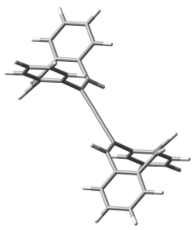	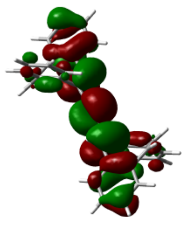	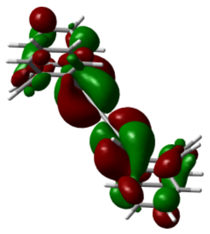
**K_2dbCOT2**			
Doubly reduced dbCOT dimer with potassium counter ions.<br>E_{tot} = -2509.052755 H<br>G_{tot} = -2508.678991 H	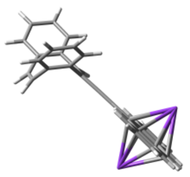	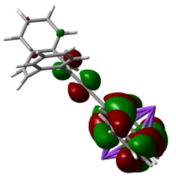	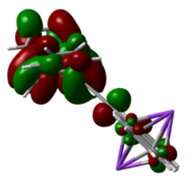

K_4dbCOT2

Fully (four-fold) reduced dbCOT dimer with potassium counter ions.

E_{tot} = -3709.010668 H

G_{tot} = -3708.643868 H

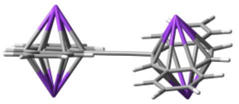
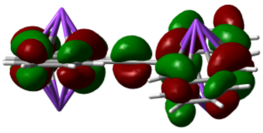
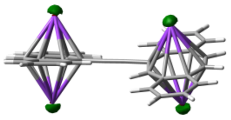

dbCOT3

Neutral dbCOT trimer.

E_{tot} = -2001.109397 H

G_{tot} = -2000.530297 H

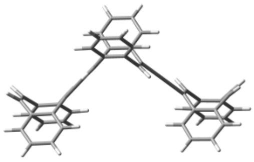
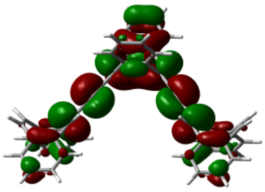
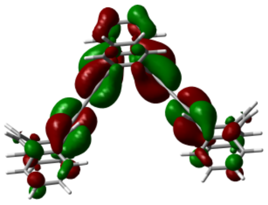

K_2dbCOT3(t)

Doubly reduced dbCOT trimer with potassium counter ions on terminal unit.

E_{tot} = -3201.081401 H

G_{tot} = -3200.510903 H

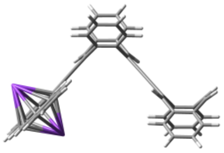
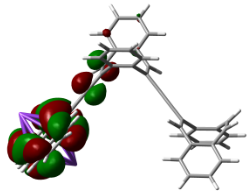
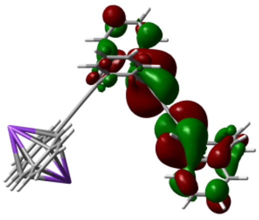

K_2dbCOT3(c)

Doubly reduced dbCOT trimer with potassium counter ions on central unit

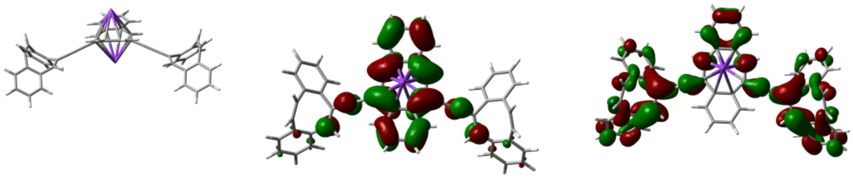

E_{tot} = -3201.078019 H

G_{tot} = -3200.510564 H

K_4dbCOT3

Fourfold reduced dbCOT trimer with potassium ions on the terminal units

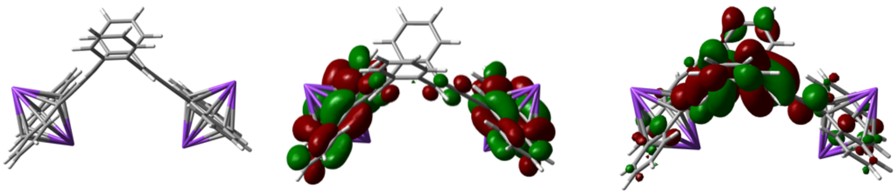

E_{tot} = -4401.05136998

G_{tot} = -4400.488721

K_6dbCOT3

Fully (six-fold) reduced dbCOT trimer with potassium counter ions

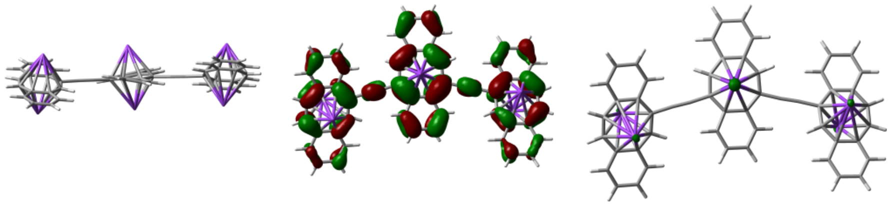

E_{tot} = -5600.994640 H

G_{tot} = -5600.441630 H

K_6dbCOT3(twist)

Fully (six-fold) reduced dbCOT trimer with potassium counter ions. Twisted dihedrals frozen in constrained optimization.

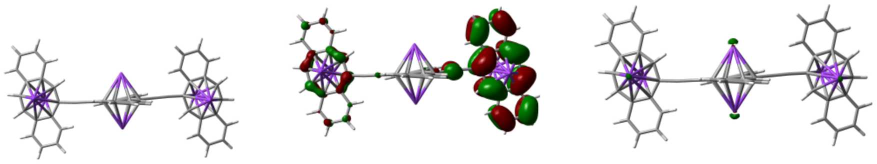

E_{tot} = -5600.992958 H

Coordinates for optimized geometries:

```
dbcot_gs_b3lyp_6311+Gdp_thf_opt (ID: tetralith_16694860)
   28
scf done:  -617.053647
 C     0.383916    -0.224942    -0.046566
 C     1.772367    -0.388138     0.128152
 C     0.923739     2.097669    -2.167818
 C     2.731671     0.736854     0.210063
 C     2.312213     1.934566    -1.993109
 C     2.958119     1.710987    -0.679526
 C    -0.440847    -1.363070    -0.018963
 H    -1.512711    -1.232996    -0.126110
 C     2.283033    -1.683182     0.323918
 H     3.349031    -1.804326     0.485990
 C     0.083311    -2.640091     0.139850
 H    -0.575886    -3.500696     0.144027
 C     1.458722    -2.801727     0.313008
 H     1.882447    -3.789579     0.453700
 H     3.393430     0.701129     1.073208
 H     3.784941     2.385101    -0.464826
 C     0.432627     2.394983    -3.451046
 H    -0.633992     2.547727    -3.578630
 C     3.156553     2.074859    -3.108316
 H     4.227794     1.976158    -2.966960
 C     1.276924     2.495274    -4.550074
 H     0.868346     2.712813    -5.530352
 C     2.652358     2.333512    -4.377037
 H     3.326628     2.423597    -5.221103
 C    -0.283356     1.091263    -0.169191
 C    -0.056996     2.065357    -1.058862
 H    -1.126212     1.232191     0.504554
 H    -0.734722     2.916368    -1.033441
---

dbcot_red2_k2_b3lyp_6311+Gdp_thf (ID: tetralith_16694739)
   30
scf done: -1817.024425
 C    -1.803465    -0.737191     0.000882
 C    -1.806819     0.728833    -0.010551
 C     1.806885    -0.728860     0.010465
 C    -0.710526     1.639585    -0.014752
 C     1.803531     0.737165    -0.000964
 C     0.703082     1.642846    -0.010992
 C    -3.099331    -1.379889     0.002488
 H    -3.100853    -2.467118     0.010964
 C    -3.105611     1.365543    -0.018953
 H    -3.112106     2.452754    -0.027442
 C    -4.299153    -0.723607    -0.005802
 H    -5.230399    -1.280663    -0.003898
 C    -4.302418     0.703730    -0.016949
 H    -5.236203     1.256482    -0.023732
```

```
 H    -1.075119     2.664922    -0.023832
 H     1.062983     2.669856    -0.018035
 C     3.105677    -1.365570     0.018878
 H     3.112172    -2.452780     0.027362
 C     3.099397     1.379863    -0.002552
 H     3.100919     2.467091    -0.011025
 C     4.302484    -0.703757     0.016889
 H     5.236269    -1.256508     0.023679
 C     4.299219     0.723581     0.005747
 H     5.230465     1.280636     0.003856
 C    -0.703016    -1.642872     0.010903
 C     0.710591    -1.639611     0.014658
 H     1.075184    -2.664948     0.023736
 H    -1.062917    -2.669882     0.017950
 K    -0.006546     0.019575     2.462246
 K     0.006661    -0.019556    -2.462340
---
dbcot2_gs_b3lyp_6311+Gdp_thf (ID: tetralith_16694810)
   56
scf done: -1309.081330
 C     4.188248     1.299239     0.199479
 C     5.107065     0.740422    -0.713423
 C     2.625139    -1.406656     0.286367
 C     4.711837    -0.111268    -1.858859
 C     3.524830    -1.972546    -0.633547
 C     4.030936    -1.262983    -1.830251
 C     4.663716     2.171591     1.194963
 H     3.951415     2.623555     1.877019
 C     6.467002     1.072744    -0.592525
 H     7.169495     0.664451    -1.311499
 C     6.016658     2.459079     1.323092
 H     6.358004     3.122234     2.109702
 C     6.926485     1.904896     0.421063
 H     7.983524     2.132525     0.498359
 H     5.103826     0.209508    -2.821698
 H     3.917027    -1.798873    -2.769993
 C     2.196043    -2.169350     1.383544
 H     1.490074    -1.733485     2.081531
 C     3.945634    -3.299636    -0.438339
 H     4.618665    -3.745732    -1.162911
 C     2.652317    -3.466381     1.584491
 H     2.315838    -4.031688     2.446035
 C     3.528668    -4.038947     0.661352
 H     3.879109    -5.056031     0.795353
 C     2.727643     1.114551     0.097191
 C     2.028688    -0.042188     0.107744
 H     2.148951     2.034619     0.071554
 C     0.605793     0.000007     0.034366
 C    -0.605673     0.000418    -0.033959
 C    -2.727420    -1.114328    -0.096341
 C    -4.663226    -2.172210    -1.193750
 C    -5.106975    -0.740182     0.713902
```

```
 C    -6.016101    -2.460021    -1.321842
 H    -3.950791    -2.624369    -1.875537
 C    -6.466840    -1.072847     0.593072
 C    -6.926095    -1.905580    -0.420136
 H    -6.357257    -3.123619    -2.108160
 H    -7.169464    -0.664352     1.311803
 H    -7.983085    -2.133458    -0.497372
 C    -2.028591     0.042500    -0.107376
 C    -4.712010     0.112144     1.858933
 H    -5.104069    -0.208203     2.821890
 C    -4.031233     1.263921     1.829846
 H    -3.917465     1.800272     2.769337
 C    -4.187987    -1.299280    -0.198660
 C    -2.625217     1.406775    -0.286683
 C    -2.196100     2.169004    -1.384184
 C    -3.525146     1.972967     0.632809
 C    -2.652611     3.465841    -1.585860
 H    -1.489941     1.732926    -2.081844
 C    -3.946213     3.299854     0.436851
 C    -3.529233     4.038689    -0.663161
 H    -2.316106     4.030790    -2.447629
 H    -4.619445     3.746185     1.161091
 H    -3.879876     5.055627    -0.797747
 H    -2.148600    -2.034312    -0.070286
---
dbcot2_red2_k2_b3lyp_6311+Gdp_thf (ID: tetralith_16694839)
   58
scf done: -2509.052755
 C    -3.526002     4.047761    -0.514199
 C    -2.766039     3.403776    -1.492060
 C    -2.469119     2.053387    -1.351395
 C    -2.944082     1.307058    -0.261079
 C    -3.733164     1.947921     0.709785
 C    -3.990794     3.323959     0.576634
 C    -2.506958    -0.126637    -0.151541
 C    -3.360730    -1.183412    -0.167871
 C    -4.833630    -1.177553    -0.219648
 C    -5.456165    -1.956733    -1.214751
 C    -6.837456    -2.077624    -1.295898
 C    -7.642581    -1.438646    -0.350735
 C    -7.046043    -0.696151     0.661298
 C    -5.651543    -0.539458     0.740673
 C    -5.119809     0.210195     1.901713
 C    -4.291410     1.261354     1.896496
 C    -1.102058    -0.317546    -0.127822
 C     0.110863    -0.455678    -0.089384
 C     1.533185    -0.531998    -0.039762
 C     2.063882     0.814424    -0.070949
 C     3.265688     1.560854    -0.042887
 C     4.657873     1.128736     0.053649
 C     5.670784     2.156970     0.061881
 C     5.417792     3.497919    -0.013458
```

```
 C      4.059376     3.928771    -0.106425
 C      3.060921     2.999106    -0.118537
 C      5.163881    -0.195447     0.145977
 C      4.698714    -1.516658     0.172882
 C      3.486633    -2.284823     0.123146
 C      2.086521    -1.878469     0.027238
 C      1.126510    -2.955446    -0.004403
 C      1.431417    -4.289987     0.049707
 C      2.789776    -4.683702     0.146968
 C      3.745013    -3.705921     0.180751
 H      2.032038     3.340865    -0.188595
 H      6.702175     1.821426     0.133782
 H      3.819248     4.985409    -0.166590
 H      6.229888     4.216643    -0.002177
 H      6.249940    -0.186056     0.213034
 H      5.548554    -2.189323     0.253605
 H      0.083540    -2.681416    -0.078361
 H      4.786545    -4.004405     0.254918
 H      0.637187    -5.028484     0.018189
 H      3.070422    -5.730510     0.193796
 H      1.225666     1.499656    -0.136423
 H     -4.830273    -2.470881    -1.936773
 H     -7.284038    -2.674498    -2.083397
 H     -7.666792    -0.224771     1.416506
 H     -8.721829    -1.532042    -0.393276
 H     -5.525968    -0.094224     2.864442
 H     -4.082439     1.737986     2.852011
 H     -1.850584     1.558148    -2.091270
 H     -2.395500     3.953864    -2.349800
 H     -4.576389     3.821794     1.342647
 H     -3.750603     5.104958    -0.600231
 H     -2.909055    -2.171328    -0.217786
 K      3.477424    -0.445453    -2.464221
 K      3.188934    -0.230054     2.559263
 ---
dbcot2_red4_k4_b3lyp_6311+Gdp_thf (ID: tetralith_16694939)
    60
scf done: -3709.010668
 C      2.743042    -4.619874    -0.783228
 C      1.441595    -4.051474    -0.732711
 C      1.309314    -2.709822    -0.494875
 C      2.399657    -1.782377    -0.280774
 C      3.745176    -2.369427    -0.348812
 C      3.815806    -3.793044    -0.598348
 C      2.012938    -0.408554    -0.028290
 C      2.705862     0.825227     0.238519
 C      3.996418     1.406826     0.367529
 C      3.966457     2.831244     0.645311
 C      5.069946     3.621128     0.805239
 C      6.366638     3.033583     0.699818
 C      6.457294     1.693549     0.443360
 C      5.327759     0.810722     0.266952
```

```
 C      5.671349    -0.545068     0.019456
 C      5.042930    -1.781127    -0.214502
 C      0.590933    -0.150134    -0.036014
 C     -0.590908     0.150287    -0.036053
 C     -2.012924     0.408621    -0.028359
 C     -2.705814    -0.825220     0.238251
 C     -3.996356    -1.406874     0.367146
 C     -5.327718    -0.810815     0.266550
 C     -6.457226    -1.693705     0.442823
 C     -6.366531    -3.033756     0.699180
 C     -5.069821    -3.621256     0.804624
 C     -3.966357    -2.831314     0.644815
 C     -5.671358     0.544984     0.019167
 C     -5.042972     1.781097    -0.214589
 C     -3.745228     2.369469    -0.348693
 C     -2.399690     1.782461    -0.280667
 C     -1.309366     2.709969    -0.494584
 C     -1.441679     4.051658    -0.732192
 C     -2.743141     4.620029    -0.782650
 C     -3.815889     3.793133    -0.597966
 H     -2.984347    -3.289760     0.725096
 H     -7.442739    -1.241388     0.363746
 H     -4.957431    -4.681411     1.007483
 H     -7.261360    -3.635045     0.820352
 H     -6.752179     0.672273     0.010674
 H     -5.804616     2.547285    -0.337123
 H     -0.310074     2.298188    -0.459889
 H     -4.812221     4.224412    -0.640428
 H     -0.558996     4.666239    -0.877610
 H     -2.888186     5.679765    -0.964890
 H     -1.960976    -1.599652     0.386957
 H      2.984461     3.289722     0.725575
 H      4.957590     4.681272     1.008174
 H      7.442793     1.241197     0.364303
 H      7.261484     3.634827     0.821088
 H      6.752165    -0.672409     0.011039
 H      5.804553    -2.547331    -0.337069
 H      0.310032    -2.298024    -0.460137
 H      0.558899    -4.666005    -0.878260
 H      4.812128    -4.224345    -0.640845
 H      2.888062    -5.679580    -0.965659
 H      1.961042     1.599664     0.387287
 K     -3.887378     0.003875    -2.430312
 K     -3.787523     0.947545     2.438501
 K      3.887542    -0.003613    -2.430088
 K      3.787311    -0.947848     2.438612
---
dbcot3_gs_b3lyp_6311+Gdp_thf (ID: tetralith_15602373)
   84
scf done: -2001.109397
 C      6.186830    -2.952322    -0.742421
 C      4.777068    -2.921452    -0.780778
```

C	6.175029	-0.617116	1.360201
C	3.992949	-1.672639	-0.837037
C	4.769837	-0.608142	1.323666
C	3.988089	-0.648052	0.044409
C	6.837459	-4.196166	-0.806425
H	7.922182	-4.218166	-0.804468
C	4.072591	-4.134156	-0.886215
H	2.989781	-4.105734	-0.945816
C	6.126148	-5.388174	-0.870356
H	6.654883	-6.333893	-0.904520
C	4.731165	-5.356908	-0.910491
H	4.162940	-6.277695	-0.976063
H	3.289618	-1.608845	-1.663648
C	6.819730	-0.466665	2.600382
H	7.904137	-0.446517	2.626012
C	4.053466	-0.477876	2.523447
H	2.970024	-0.456451	2.487991
C	6.100382	-0.356358	3.783674
H	6.622667	-0.254875	4.728208
C	4.705268	-0.369913	3.745875
H	4.130142	-0.282238	4.660513
C	7.034731	-1.737956	-0.732589
C	7.027316	-0.736982	0.155619
H	7.806918	-1.715398	-1.498651
H	7.793077	0.028485	0.050989
C	3.117551	0.458650	-0.177003
C	2.390543	1.416599	-0.338104
C	1.513065	2.516464	-0.557765
C	0.701214	2.468206	-1.816605
C	1.515782	3.564353	0.295822
C	-0.701306	2.468251	-1.816566
C	1.384953	2.384518	-3.039353
C	0.706303	4.795915	0.211499
H	2.234055	3.535898	1.111328
C	-1.385108	2.384612	-3.039286
C	-1.513111	2.516548	-0.557694
H	2.469009	2.368304	-3.034574
C	0.697750	2.324687	-4.245293
C	-0.706221	4.795961	0.211531
C	1.384436	6.026674	0.250028
H	-2.469165	2.368469	-3.034453
C	-0.697967	2.324738	-4.245259
C	-1.515764	3.564447	0.295883
H	1.246255	2.272244	-5.178790
C	-1.384272	6.026764	0.250097
H	2.468770	6.024748	0.280117
C	0.698123	7.234562	0.245932
H	-1.246520	2.272334	-5.178730
H	-2.234018	3.536032	1.111407
H	-2.468605	6.024908	0.280237
C	-0.697880	7.234607	0.245967
H	1.246641	8.169393	0.259015

```
 H    -1.246338     8.169473     0.259077
 C    -2.390613     1.416709    -0.337999
 C    -3.117607     0.458747    -0.176943
 C    -3.988137    -0.647966     0.044447
 C    -4.769888    -0.608116     1.323700
 C    -3.992971    -1.672536    -0.837023
 C    -6.175078    -0.617192     1.360220
 C    -4.053534    -0.477811     2.523486
 C    -4.777032    -2.921391    -0.780784
 H    -3.289649    -1.608701    -1.663638
 C    -6.819802    -0.466828     2.600402
 C    -7.027357    -0.737061     0.155629
 H    -2.970093    -0.456296     2.488035
 C    -4.705353    -0.369922     3.745911
 C    -6.186793    -2.952338    -0.742466
 C    -4.072486    -4.134057    -0.886214
 H    -7.904211    -0.446767     2.626025
 C    -6.100470    -0.356489     3.783700
 C    -7.034748    -1.738013    -0.732604
 H    -7.793137     0.028391     0.051021
 H    -4.130241    -0.282212     4.660554
 C    -6.837353    -4.196215    -0.806523
 H    -2.989675    -4.105575    -0.945779
 C    -4.730995    -5.356843    -0.910539
 H    -6.622768    -0.255073     4.728234
 H    -7.806942    -1.715463    -1.498659
 H    -7.922076    -4.218272    -0.804601
 C    -6.125978    -5.388183    -0.870458
 H    -4.162719    -6.277599    -0.976106
 H    -6.654661    -6.333930    -0.904662
---
dbcot3_red2_k2c_b3lyp_6311+Gdp_thf (ID: tetralith_15604809)
    86
scf done: -3201.078019
 C    -9.113069     4.374748    -0.831381
 C    -9.199035     3.265750     0.001692
 C    -8.087265     2.445462     0.259819
 C    -6.841846     2.774274    -0.323337
 C    -6.767498     3.925204    -1.132994
 C    -7.882406     4.708797    -1.400574
 C    -5.585987     2.060241    -0.035102
 C    -5.307200     0.737224    -0.180647
 C    -6.260859    -0.261473    -0.773707
 C    -7.484872    -0.606792    -0.175273
 C    -8.253164    -1.638068    -0.744121
 C    -7.853834    -2.284768    -1.906959
 C    -6.652040    -1.920909    -2.516641
 C    -5.863457    -0.931245    -1.941961
 C    -8.007447     0.037110     1.050808
 C    -8.274841     1.336980     1.223673
 C    -4.026361     0.222488     0.136364
 C    -2.932060    -0.234604     0.433069
```

C	-1.660038	-0.799478	0.716660
C	-1.638087	-2.190030	0.379078
C	-0.738592	-3.269025	0.272806
C	0.726186	-3.256317	0.226174
C	1.374535	-4.533848	-0.014516
C	0.717798	-5.718393	-0.164464
C	-0.712964	-5.732075	-0.109709
C	-1.379024	-4.559725	0.086520
C	1.615579	-2.165051	0.297648
C	1.647085	-0.784909	0.675325
C	0.722284	0.102307	1.394655
C	-0.724730	0.099173	1.407715
C	-1.360405	1.130535	2.175556
C	-0.697285	2.097576	2.892219
C	0.711004	2.097687	2.883971
C	1.366520	1.132620	2.157917
C	2.918440	-0.218439	0.389889
C	4.013122	0.239120	0.096161
C	5.298634	0.749133	-0.209862
C	6.246866	-0.249362	-0.812027
C	7.462958	-0.616042	-0.210174
C	8.224237	-1.647341	-0.788382
C	7.826286	-2.272945	-1.963173
C	6.632840	-1.887460	-2.575986
C	5.850608	-0.897805	-1.992598
C	7.983932	0.005254	1.028029
C	8.261037	1.300170	1.222106
C	8.089637	2.425047	0.274363
C	6.851644	2.774183	-0.312762
C	6.793597	3.938063	-1.105002
C	7.917437	4.715489	-1.352354
C	9.140747	4.361621	-0.779290
C	9.210585	3.239199	0.037094
C	5.587348	2.067451	-0.044126
H	10.151733	2.980054	0.511384
H	5.838866	4.225335	-1.533233
H	10.025243	4.963853	-0.953288
H	7.839448	5.597510	-1.978296
H	4.765319	2.708081	0.265944
H	9.148427	-1.947460	-0.305382
H	4.907576	-0.618456	-2.448742
H	8.438742	-3.055805	-2.396190
H	6.308340	-2.364621	-3.493960
H	8.748999	1.565655	2.158016
H	8.265600	-0.690018	1.816075
H	2.614538	-2.474547	0.003692
H	2.448297	1.136721	2.167090
H	-2.442037	1.131040	2.200159
H	1.276205	2.831652	3.448399
H	2.459573	-4.525289	-0.067026
H	-1.256129	2.830948	3.463664
H	-2.647530	-2.514906	0.142337

```
 H    -2.464977    -4.570917     0.112353
 H     1.271993    -6.635752    -0.331336
 H    -1.260517    -6.660103    -0.234260
 H    -4.760272     2.698223     0.270649
 H    -4.914567    -0.668246    -2.395632
 H    -9.183802    -1.921409    -0.263393
 H    -8.298746    -0.644050     1.847627
 H    -6.326221    -2.414702    -3.425309
 H    -5.807119     4.197351    -1.558478
 H    -8.471656    -3.067183    -2.333094
 H    -8.765631     1.621074     2.152598
 H   -10.145930     3.022355     0.472886
 H    -7.791824     5.580321    -2.039402
 H    -9.990786     4.982064    -1.021311
 K    -0.083961    -1.027085    -1.994492
 K     0.120769    -2.416051     3.142509
---
dbcot3_red2_k2t_b3lyp_6311+Gdp_thf (ID: tetralith_16694030)
    86
scf done: -3201.081401
 C    -5.958928    -5.709692    -0.507406
 C    -6.768704    -4.584733    -0.605734
 C    -6.229370    -3.287213    -0.618797
 C    -4.828063    -3.134062    -0.565867
 C    -4.020441    -4.284098    -0.507567
 C    -4.572139    -5.557972    -0.458267
 C    -4.147718    -1.830233    -0.688937
 C    -4.292682    -0.734465     0.089169
 C    -5.168757    -0.651154     1.303757
 C    -6.567130    -0.782792     1.247072
 C    -7.313334    -0.580602     2.421254
 C    -6.696633    -0.300666     3.634118
 C    -5.306353    -0.191394     3.691044
 C    -4.558096    -0.349267     2.530874
 C    -7.314156    -1.084394     0.004998
 C    -7.172725    -2.157154    -0.782341
 C    -3.504007     0.422363    -0.177653
 C    -2.850860     1.424883    -0.380183
 C    -2.060230     2.578476    -0.649667
 C    -2.217656     3.693922     0.097380
 C    -1.518453     4.984787    -0.046485
 C    -0.111525     5.118710     0.035428
 C     0.434315     6.417144     0.023762
 C    -0.361694     7.547873    -0.105423
 C    -1.748855     7.410638    -0.193328
 C    -2.312102     6.141811    -0.142614
 C     0.807256     3.989702     0.264274
 C     0.958768     2.864604    -0.483327
 C     0.224693     2.641717    -1.774114
 C    -1.168497     2.501407    -1.851280
 C    -1.767092     2.251716    -3.096547
 C    -1.007723     2.166654    -4.256638
```

C	0.378713	2.311361	-4.183118
C	0.982178	2.534968	-2.951785
C	1.912747	1.872131	-0.151521
C	2.720150	0.996052	0.118227
C	3.640134	-0.064123	0.359523
C	4.441558	0.064482	1.570255
C	5.469845	-0.837750	2.082049
C	6.114241	-0.449850	3.316494
C	5.832124	0.680595	4.032405
C	4.829526	1.552956	3.539483
C	4.186870	1.237218	2.371426
C	5.982118	-2.080657	1.577009
C	5.829971	-2.981027	0.514782
C	5.057895	-3.141030	-0.666911
C	4.027211	-2.254269	-1.200873
C	3.406704	-2.692221	-2.441738
C	3.724417	-3.839958	-3.107444
C	4.734547	-4.700499	-2.579527
C	5.351858	-4.340156	-1.414621
C	3.519424	-1.026209	-0.715562
H	6.121455	-4.990197	-1.006350
H	2.636112	-2.047125	-2.854430
H	5.005796	-5.618288	-3.089659
H	3.211574	-4.093165	-4.029738
H	2.755389	-0.667552	-1.396611
H	6.880683	-1.120987	3.692650
H	3.425623	1.918014	2.017438
H	6.363875	0.897261	4.952756
H	4.567223	2.460124	4.073676
H	6.518018	-3.815018	0.637828
H	6.751553	-2.452431	2.248493
H	1.491095	4.118809	1.099602
H	2.060114	2.630116	-2.887785
H	-2.843109	2.126745	-3.145882
H	0.985196	2.243876	-5.079345
H	1.509883	6.526817	0.114918
H	-1.491718	1.985006	-5.209554
H	-2.987681	3.667313	0.864751
H	-3.391128	6.032300	-0.176272
H	0.094027	8.531566	-0.125958
H	-2.384038	8.284488	-0.282921
H	-3.393054	-1.777946	-1.469843
H	-3.480879	-0.231159	2.565592
H	-8.394520	-0.655302	2.371795
H	-8.129919	-0.401877	-0.223027
H	-4.810903	0.030747	4.629262
H	-2.942211	-4.164702	-0.499070
H	-7.294939	-0.162007	4.527449
H	-7.884160	-2.271695	-1.597433
H	-7.845065	-4.702762	-0.673694
H	-3.926646	-6.426794	-0.398359
H	-6.403874	-6.697959	-0.485700

```
 K      2.958061    -2.767641     1.724540
 K      6.467528    -0.275906    -0.897258
---
dbcot3_red4_k4tt_b3lyp_6311+Gdp_thf (ID: tetralith_17094502)
   88
scf done: -4401.05136998
 6    -5.197259   -4.740951   -2.427235
 6    -5.818796   -4.348632   -1.275014
 6    -5.508109   -3.143101   -0.544077
 6    -4.456297   -2.285038   -1.084497
 6    -3.832109   -2.756722   -2.310798
 6    -4.165070   -3.909822   -2.959734
 6    -3.933703   -1.055004   -0.618769
 6    -4.051866   -0.069293    0.433997
 6    -4.839112    0.079372    1.650603
 6    -5.881487   -0.800024    2.175933
 6    -6.510392   -0.390778    3.411674
 6    -6.199783    0.737268    4.119439
 6    -5.180479    1.584674    3.616232
 6    -4.552800    1.249656    2.445530
 6    -6.422411   -2.034182    1.681028
 6    -6.286545   -2.949283    0.628283
 6    -3.147977    0.998065    0.154054
 6    -2.366876    1.884349   -0.154782
 6    -1.467547    2.906818   -0.551091
 6    -1.425966    4.104065    0.088934
 6    -0.596045    5.279854   -0.227674
 6     0.820036    5.255119   -0.277937
 6     1.507860    6.470237   -0.462589
 6     0.840174    7.677400   -0.626334
 6    -0.555587    7.702068   -0.575556
 6    -1.251961    6.518964   -0.363317
 6     1.626696    4.051426   -0.009188
 6     1.588753    2.850370   -0.642242
 6     0.720311    2.590991   -1.838699
 6    -0.681895    2.623572   -1.798783
 6    -1.402830    2.348187   -2.972757
 6    -0.758962    2.064976   -4.170636
 6     0.635990    2.029917   -4.209816
 6     1.359492    2.280458   -3.050741
 6     2.479303    1.804456   -0.288493
 6     3.251055    0.904544    0.003408
 6     4.119434   -0.190184    0.290391
 6     5.121667    0.058763    1.318155
 6     6.156889   -0.842497    1.821011
 6     7.028517   -0.317267    2.848126
 6     6.953875    0.940192    3.379710
 6     5.945639    1.812571    2.897228
 6     5.092406    1.370019    1.921214
 6     6.486341   -2.197280    1.479538
 6     6.089587   -3.245659    0.638339
 6     5.116106   -3.524292   -0.357895
```

```
 6     4.077007   -2.640493   -0.881279
 6     3.223806   -3.217286   -1.908553
 6     3.336333   -4.488944   -2.390128
 6     4.354936   -5.346489   -1.873535
 6     5.187652   -4.857883   -0.906204
 6     3.750328   -1.301607   -0.559175
 1     5.965691   -5.504656   -0.508809
 1     2.444752   -2.575570   -2.310492
 1     4.466407   -6.361226   -2.239774
 1     2.656440   -4.841372   -3.159047
 1     2.910613   -0.987426   -1.169387
 1     7.798827   -0.988313    3.216595
 1     4.333031    2.054520    1.570588
 1     7.649468    1.255315    4.150192
 1     5.843555    2.818811    3.289928
 1     6.726625   -4.112183    0.804032
 1     7.331364   -2.525305    2.079170
 1     2.395455    4.178439    0.749558
 1     2.442704    2.240281   -3.071659
 1    -2.486163    2.359214   -2.932314
 1     1.154358    1.804595   -5.135208
 1     2.592966    6.453552   -0.474402
 1    -1.339122    1.867438   -5.065184
 1    -2.145195    4.254467    0.890640
 1    -2.335069    6.540489   -0.297751
 1     1.401465    8.592556   -0.779042
 1    -1.093778    8.636714   -0.688142
 1    -3.165121   -0.716745   -1.305222
 1    -3.776625    1.910002    2.085096
 1    -7.289169   -1.043001    3.795884
 1    -7.202252   -2.381718    2.353580
 1    -4.893102    2.487073    4.145618
 1    -3.045507   -2.133577   -2.726976
 1    -6.721297    0.970531    5.041628
 1    -6.994965   -3.765317    0.756192
 1    -6.605024   -4.976486   -0.863622
 1    -3.648199   -4.189006   -3.872197
 1    -5.481694   -5.661972   -2.924274
19     3.506249   -2.533444    2.216809
19     6.673986   -0.897892   -1.318357
19    -6.870213   -0.254689   -0.814583
19    -3.423780   -2.777869    1.836289
---
dbcot3_red6_k6_b3lyp_6311+Gdp_thf_ctd2 (ID: te-
tralith_15662248)
   90
scf done: -5600.994640
 C    -8.167207    -4.950824    -1.244019
 C    -8.809692    -3.830835    -0.794240
 C    -8.159345    -2.581823    -0.472739
 C    -6.707385    -2.526834    -0.636736
 C    -6.084505    -3.745348    -1.121293
```

C	-6.750604	-4.901829	-1.414303
C	-5.785253	-1.470975	-0.403507
C	-5.675846	-0.108011	0.049832
C	-6.600810	0.916457	0.493828
C	-8.062901	0.851968	0.622629
C	-8.723968	2.056105	1.077331
C	-8.100480	3.228804	1.401333
C	-6.685132	3.288847	1.290233
C	-6.003042	2.182526	0.859983
C	-8.988912	-0.212727	0.381599
C	-9.037944	-1.553736	-0.038321
C	-4.282828	0.273057	0.041295
C	-3.087350	0.511981	0.013261
C	-1.695360	0.900938	-0.024061
C	-1.627454	2.325177	-0.162627
C	-0.722298	3.413038	-0.102561
C	0.722177	3.413010	0.102923
C	1.351320	4.711198	0.243953
C	0.700996	5.907911	0.134627
C	-0.701097	5.907934	-0.134053
C	-1.351429	4.711236	-0.243491
C	1.627350	2.325146	0.162885
C	1.695246	0.900875	0.024596
C	0.735592	-0.189473	-0.054408
C	-0.735755	-0.189409	0.055656
C	-1.339652	-1.491856	0.235675
C	-0.692016	-2.695015	0.149320
C	0.691785	-2.695135	-0.146422
C	1.339455	-1.492056	-0.233571
C	3.087221	0.511917	-0.013065
C	4.282700	0.273039	-0.041440
C	5.675708	-0.108058	-0.050265
C	6.600606	0.916426	-0.494372
C	8.062672	0.851930	-0.623467
C	8.723618	2.055954	-1.078655
C	8.100064	3.228628	-1.402617
C	6.684765	3.288768	-1.290946
C	6.002780	2.182521	-0.860347
C	8.988796	-0.212624	-0.382198
C	9.037968	-1.553457	0.038252
C	8.159419	-2.581556	0.472769
C	6.707386	-2.526823	0.636262
C	6.084516	-3.745550	1.120296
C	6.750736	-4.901851	1.413734
C	8.167473	-4.950433	1.244448
C	8.809922	-3.830315	0.794940
C	5.785182	-1.471042	0.402975
H	9.889073	-3.861828	0.666575
H	5.006010	-3.720686	1.251105
H	8.723835	-5.854645	1.467534
H	6.205795	-5.770374	1.769296
H	4.788221	-1.819629	0.650072

```
 H      9.805343     2.006059    -1.167101
 H      4.926326     2.249992    -0.781708
 H      8.675585     4.085445    -1.737155
 H      6.144004     4.196225    -1.540029
 H     10.067689    -1.905707     0.030412
 H      9.998370     0.126581    -0.600831
 H      2.633243     2.705485     0.307391
 H      2.403201    -1.506383    -0.424651
 H     -2.403397    -1.506040     0.426774
 H      1.240783    -3.621213    -0.284089
 H      2.421864     4.714215     0.430851
 H     -1.241040    -3.620981     0.287625
 H     -2.633320     2.705468    -0.307442
 H     -2.421970     4.714262    -0.430402
 H      1.245747     6.840332     0.239938
 H     -1.245835     6.840371    -0.239281
 H     -4.788294    -1.819434    -0.650781
 H     -4.926556     2.249935     0.781722
 H     -9.805725     2.006259     1.165412
 H     -9.998537     0.126473     0.600009
 H     -6.144417     4.196291     1.539463
 H     -5.006089    -3.720196    -1.252788
 H     -8.676089     4.085697     1.735526
 H    -10.067605    -1.906157    -0.030072
 H     -9.888756    -3.862623    -0.665209
 H     -6.205666    -5.770195    -1.770254
 H     -8.723446    -5.855232    -1.466619
 K      7.623968    -0.062085     2.347385
 K      7.079719    -1.600128    -2.339456
 K     -0.164897     1.624540     2.505408
 K      0.165516     1.624331    -2.504906
 K     -7.623818    -0.062533    -2.348052
 K     -7.079513    -1.599627     2.339264
---
dbcot3_red6_k6_twisted_b3lyp_6311+Gdp_thf_ctd (ID: tetralith
166939759)
  6    -9.200014    4.053040   -0.588087
  6    -9.530286    2.756969   -0.871392
  6    -8.616350    1.640477   -0.801934
  6    -7.240794    1.931282   -0.400562
  6    -6.952576    3.325076   -0.114266
  6    -7.858669    4.344437   -0.194345
  6    -6.117582    1.074822   -0.242940
  6    -5.681928   -0.295078   -0.338374
  6    -6.305474   -1.561198   -0.671935
  6    -7.692967   -1.834114   -1.071698
  6    -8.023702   -3.214236   -1.354992
  6    -7.158148   -4.269282   -1.276833
  6    -5.817515   -4.008312   -0.885005
  6    -5.442811   -2.721310   -0.606453
  6    -8.818190   -0.965698   -1.241406
  6    -9.190067    0.386404   -1.140182
```

6	-4.275468	-0.361441	-0.003704
6	-3.086930	-0.337713	0.264626
6	-1.695288	-0.332719	0.667360
6	-1.634738	-0.534451	2.083871
6	-0.726372	-0.734357	3.150883
6	0.732237	-0.756177	3.143395
6	1.377300	-1.074473	4.402897
6	0.720862	-1.308287	5.577067
6	-0.706330	-1.240843	5.594299
6	-1.366800	-0.965728	4.431358
6	1.636679	-0.489229	2.087731
6	1.691837	-0.100822	0.709997
6	0.728701	0.087985	-0.367279
6	-0.733093	-0.110254	-0.404724
6	-1.330341	-0.050025	-1.722020
6	-0.689668	0.293735	-2.881425
6	0.682007	0.633247	-2.816642
6	1.323986	0.522359	-1.613154
6	3.079957	0.107868	0.351111
6	4.265740	0.278042	0.127162
6	5.671143	0.415348	-0.189594
6	6.233101	1.734690	0.027458
6	7.604036	2.208215	-0.208857
6	7.869773	3.597052	0.099678
6	6.958019	4.488704	0.591528
6	5.633228	4.031644	0.825760
6	5.319021	2.728037	0.549285
6	8.766415	1.535605	-0.704050
6	9.200394	0.277323	-1.156983
6	8.687824	-1.030679	-1.363502
6	7.330382	-1.521486	-1.130339
6	7.109121	-2.923093	-1.437760
6	8.060226	-3.779325	-1.915917
6	9.383342	-3.292536	-2.142704
6	9.651525	-1.979868	-1.870264
6	6.170134	-0.854518	-0.651974
1	10.657515	-1.604611	-2.041156
1	6.105099	-3.303995	-1.270602
1	10.159835	-3.948095	-2.522399
1	7.809233	-4.815098	-2.120624
1	5.348362	-1.561616	-0.627091
1	8.883718	3.943599	-0.079940
1	4.305438	2.399445	0.732984
1	7.244528	5.515125	0.795680
1	4.874239	4.701433	1.217553
1	10.252996	0.325390	-1.429348
1	9.600684	2.231794	-0.742923
1	2.652070	-0.575121	2.458681
1	2.377183	0.760598	-1.580571
1	-2.383320	-0.284784	-1.778900
1	1.225781	0.961709	-3.696803
1	2.463326	-1.114322	4.395010

```
  1    -1.233843    0.313854   -3.820441
  1    -2.648940   -0.589579    2.463922
  1    -2.452762   -0.925809    4.444572
  1     1.276056   -1.533546    6.481631
  1    -1.258270   -1.411511    6.512693
  1    -5.265404    1.672524    0.060860
  1    -4.418513   -2.542905   -0.309494
  1    -9.049696   -3.410857   -1.653473
  1    -9.682439   -1.547059   -1.553216
  1    -5.093863   -4.813407   -0.805436
  1    -5.934237    3.558551    0.185058
  1    -7.492152   -5.275465   -1.507776
  1   -10.240770    0.501344   -1.399503
  1   -10.550345    2.530245   -1.171567
  1    -7.558705    5.361271    0.037590
  1    -9.941784    4.841230   -0.661716
 19     8.040202   -0.267001    1.660781
 19     6.809410    0.956597   -2.992228
 19     0.095406   -2.813615    0.865369
 19    -0.095429    2.080311    1.883487
 19    -8.039209   -0.311529    1.648385
 19    -6.810398    0.397817   -3.109913
{Constraints: {D 7 8 19 20 F; D 27 28 37 52 F}}
---
dbcot_gs_b3lyp_6311+Gdp_thf_opt (ID: tetralith_16694860)
   28
scf done:  -617.053647
 C     0.383916    -0.224942    -0.046566
 C     1.772367    -0.388138     0.128152
 C     0.923739     2.097669    -2.167818
 C     2.731671     0.736854     0.210063
 C     2.312213     1.934566    -1.993109
 C     2.958119     1.710987    -0.679526
 C    -0.440847    -1.363070    -0.018963
 H    -1.512711    -1.232996    -0.126110
 C     2.283033    -1.683182     0.323918
 H     3.349031    -1.804326     0.485990
 C     0.083311    -2.640091     0.139850
 H    -0.575886    -3.500696     0.144027
 C     1.458722    -2.801727     0.313008
 H     1.882447    -3.789579     0.453700
 H     3.393430     0.701129     1.073208
 H     3.784941     2.385101    -0.464826
 C     0.432627     2.394983    -3.451046
 H    -0.633992     2.547727    -3.578630
 C     3.156553     2.074859    -3.108316
 H     4.227794     1.976158    -2.966960
 C     1.276924     2.495274    -4.550074
 H     0.868346     2.712813    -5.530352
 C     2.652358     2.333512    -4.377037
 H     3.326628     2.423597    -5.221103
 C    -0.283356     1.091263    -0.169191
```

```
 C    -0.056996     2.065357    -1.058862
 H    -1.126212     1.232191     0.504554
 H    -0.734722     2.916368    -1.033441
---
dbcot_red2_k2_b3lyp_6311+Gdp_thf (ID: tetralith_16694739)
   30
scf done: -1817.024425
 C    -1.803465    -0.737191     0.000882
 C    -1.806819     0.728833    -0.010551
 C     1.806885    -0.728860     0.010465
 C    -0.710526     1.639585    -0.014752
 C     1.803531     0.737165    -0.000964
 C     0.703082     1.642846    -0.010992
 C    -3.099331    -1.379889     0.002488
 H    -3.100853    -2.467118     0.010964
 C    -3.105611     1.365543    -0.018953
 H    -3.112106     2.452754    -0.027442
 C    -4.299153    -0.723607    -0.005802
 H    -5.230399    -1.280663    -0.003898
 C    -4.302418     0.703730    -0.016949
 H    -5.236203     1.256482    -0.023732
 H    -1.075119     2.664922    -0.023832
 H     1.062983     2.669856    -0.018035
 C     3.105677    -1.365570     0.018878
 H     3.112172    -2.452780     0.027362
 C     3.099397     1.379863    -0.002552
 H     3.100919     2.467091    -0.011025
 C     4.302484    -0.703757     0.016889
 H     5.236269    -1.256508     0.023679
 C     4.299219     0.723581     0.005747
 H     5.230465     1.280636     0.003856
 C    -0.703016    -1.642872     0.010903
 C     0.710591    -1.639611     0.014658
 H     1.075184    -2.664948     0.023736
 H    -1.062917    -2.669882     0.017950
 K    -0.006546     0.019575     2.462246
 K     0.006661    -0.019556    -2.462340
---
dbcot2_gs_b3lyp_6311+Gdp_thf (ID: tetralith_16694810)
   56
scf done: -1309.081330
 C     4.188248     1.299239     0.199479
 C     5.107065     0.740422    -0.713423
 C     2.625139    -1.406656     0.286367
 C     4.711837    -0.111268    -1.858859
 C     3.524830    -1.972546    -0.633547
 C     4.030936    -1.262983    -1.830251
 C     4.663716     2.171591     1.194963
 H     3.951415     2.623555     1.877019
 C     6.467002     1.072744    -0.592525
 H     7.169495     0.664451    -1.311499
 C     6.016658     2.459079     1.323092
```

```
 H     6.358004     3.122234     2.109702
 C     6.926485     1.904896     0.421063
 H     7.983524     2.132525     0.498359
 H     5.103826     0.209508    -2.821698
 H     3.917027    -1.798873    -2.769993
 C     2.196043    -2.169350     1.383544
 H     1.490074    -1.733485     2.081531
 C     3.945634    -3.299636    -0.438339
 H     4.618665    -3.745732    -1.162911
 C     2.652317    -3.466381     1.584491
 H     2.315838    -4.031688     2.446035
 C     3.528668    -4.038947     0.661352
 H     3.879109    -5.056031     0.795353
 C     2.727643     1.114551     0.097191
 C     2.028688    -0.042188     0.107744
 H     2.148951     2.034619     0.071554
 C     0.605793     0.000007     0.034366
 C    -0.605673     0.000418    -0.033959
 C    -2.727420    -1.114328    -0.096341
 C    -4.663226    -2.172210    -1.193750
 C    -5.106975    -0.740182     0.713902
 C    -6.016101    -2.460021    -1.321842
 H    -3.950791    -2.624369    -1.875537
 C    -6.466840    -1.072847     0.593072
 C    -6.926095    -1.905580    -0.420136
 H    -6.357257    -3.123619    -2.108160
 H    -7.169464    -0.664352     1.311803
 H    -7.983085    -2.133458    -0.497372
 C    -2.028591     0.042500    -0.107376
 C    -4.712010     0.112144     1.858933
 H    -5.104069    -0.208203     2.821890
 C    -4.031233     1.263921     1.829846
 H    -3.917465     1.800272     2.769337
 C    -4.187987    -1.299280    -0.198660
 C    -2.625217     1.406775    -0.286683
 C    -2.196100     2.169004    -1.384184
 C    -3.525146     1.972967     0.632809
 C    -2.652611     3.465841    -1.585860
 H    -1.489941     1.732926    -2.081844
 C    -3.946213     3.299854     0.436851
 C    -3.529233     4.038689    -0.663161
 H    -2.316106     4.030790    -2.447629
 H    -4.619445     3.746185     1.161091
 H    -3.879876     5.055627    -0.797747
 H    -2.148600    -2.034312    -0.070286
---
dbcot2_red2_k2_b3lyp_6311+Gdp_thf (ID: tetralith_16694839)
   58
scf done: -2509.052755
 C    -3.526002     4.047761    -0.514199
 C    -2.766039     3.403776    -1.492060
 C    -2.469119     2.053387    -1.351395
```

C	-2.944082	1.307058	-0.261079
C	-3.733164	1.947921	0.709785
C	-3.990794	3.323959	0.576634
C	-2.506958	-0.126637	-0.151541
C	-3.360730	-1.183412	-0.167871
C	-4.833630	-1.177553	-0.219648
C	-5.456165	-1.956733	-1.214751
C	-6.837456	-2.077624	-1.295898
C	-7.642581	-1.438646	-0.350735
C	-7.046043	-0.696151	0.661298
C	-5.651543	-0.539458	0.740673
C	-5.119809	0.210195	1.901713
C	-4.291410	1.261354	1.896496
C	-1.102058	-0.317546	-0.127822
C	0.110863	-0.455678	-0.089384
C	1.533185	-0.531998	-0.039762
C	2.063882	0.814424	-0.070949
C	3.265688	1.560854	-0.042887
C	4.657873	1.128736	0.053649
C	5.670784	2.156970	0.061881
C	5.417792	3.497919	-0.013458
C	4.059376	3.928771	-0.106425
C	3.060921	2.999106	-0.118537
C	5.163881	-0.195447	0.145977
C	4.698714	-1.516658	0.172882
C	3.486633	-2.284823	0.123146
C	2.086521	-1.878469	0.027238
C	1.126510	-2.955446	-0.004403
C	1.431417	-4.289987	0.049707
C	2.789776	-4.683702	0.146968
C	3.745013	-3.705921	0.180751
H	2.032038	3.340865	-0.188595
H	6.702175	1.821426	0.133782
H	3.819248	4.985409	-0.166590
H	6.229888	4.216643	-0.002177
H	6.249940	-0.186056	0.213034
H	5.548554	-2.189323	0.253605
H	0.083540	-2.681416	-0.078361
H	4.786545	-4.004405	0.254918
H	0.637187	-5.028484	0.018189
H	3.070422	-5.730510	0.193796
H	1.225666	1.499656	-0.136423
H	-4.830273	-2.470881	-1.936773
H	-7.284038	-2.674498	-2.083397
H	-7.666792	-0.224771	1.416506
H	-8.721829	-1.532042	-0.393276
H	-5.525968	-0.094224	2.864442
H	-4.082439	1.737986	2.852011
H	-1.850584	1.558148	-2.091270
H	-2.395500	3.953864	-2.349800
H	-4.576389	3.821794	1.342647
H	-3.750603	5.104958	-0.600231

```
 H    -2.909055    -2.171328    -0.217786
 K     3.477424    -0.445453    -2.464221
 K     3.188934    -0.230054     2.559263
 ---
dbcot2_red4_k4_b3lyp_6311+Gdp_thf (ID: tetralith_16694939)
    60
scf done: -3709.010668
 C     2.743042    -4.619874    -0.783228
 C     1.441595    -4.051474    -0.732711
 C     1.309314    -2.709822    -0.494875
 C     2.399657    -1.782377    -0.280774
 C     3.745176    -2.369427    -0.348812
 C     3.815806    -3.793044    -0.598348
 C     2.012938    -0.408554    -0.028290
 C     2.705862     0.825227     0.238519
 C     3.996418     1.406826     0.367529
 C     3.966457     2.831244     0.645311
 C     5.069946     3.621128     0.805239
 C     6.366638     3.033583     0.699818
 C     6.457294     1.693549     0.443360
 C     5.327759     0.810722     0.266952
 C     5.671349    -0.545068     0.019456
 C     5.042930    -1.781127    -0.214502
 C     0.590933    -0.150134    -0.036014
 C    -0.590908     0.150287    -0.036053
 C    -2.012924     0.408621    -0.028359
 C    -2.705814    -0.825220     0.238251
 C    -3.996356    -1.406874     0.367146
 C    -5.327718    -0.810815     0.266550
 C    -6.457226    -1.693705     0.442823
 C    -6.366531    -3.033756     0.699180
 C    -5.069821    -3.621256     0.804624
 C    -3.966357    -2.831314     0.644815
 C    -5.671358     0.544984     0.019167
 C    -5.042972     1.781097    -0.214589
 C    -3.745228     2.369469    -0.348693
 C    -2.399690     1.782461    -0.280667
 C    -1.309366     2.709969    -0.494584
 C    -1.441679     4.051658    -0.732192
 C    -2.743141     4.620029    -0.782650
 C    -3.815889     3.793133    -0.597966
 H    -2.984347    -3.289760     0.725096
 H    -7.442739    -1.241388     0.363746
 H    -4.957431    -4.681411     1.007483
 H    -7.261360    -3.635045     0.820352
 H    -6.752179     0.672273     0.010674
 H    -5.804616     2.547285    -0.337123
 H    -0.310074     2.298188    -0.459889
 H    -4.812221     4.224412    -0.640428
 H    -0.558996     4.666239    -0.877610
 H    -2.888186     5.679765    -0.964890
 H    -1.960976    -1.599652     0.386957
```

```
 H      2.984461      3.289722      0.725575
 H      4.957590      4.681272      1.008174
 H      7.442793      1.241197      0.364303
 H      7.261484      3.634827      0.821088
 H      6.752165     -0.672409      0.011039
 H      5.804553     -2.547331     -0.337069
 H      0.310032     -2.298024     -0.460137
 H      0.558899     -4.666005     -0.878260
 H      4.812128     -4.224345     -0.640845
 H      2.888062     -5.679580     -0.965659
 H      1.961042      1.599664      0.387287
 K     -3.887378      0.003875     -2.430312
 K     -3.787523      0.947545      2.438501
 K      3.887542     -0.003613     -2.430088
 K      3.787311     -0.947848      2.438612
---
dbcot3_gs_b3lyp_6311+Gdp_thf (ID: tetralith_15602373)
   84
scf done: -2001.109397
 C      6.186830     -2.952322     -0.742421
 C      4.777068     -2.921452     -0.780778
 C      6.175029     -0.617116      1.360201
 C      3.992949     -1.672639     -0.837037
 C      4.769837     -0.608142      1.323666
 C      3.988089     -0.648052      0.044409
 C      6.837459     -4.196166     -0.806425
 H      7.922182     -4.218166     -0.804468
 C      4.072591     -4.134156     -0.886215
 H      2.989781     -4.105734     -0.945816
 C      6.126148     -5.388174     -0.870356
 H      6.654883     -6.333893     -0.904520
 C      4.731165     -5.356908     -0.910491
 H      4.162940     -6.277695     -0.976063
 H      3.289618     -1.608845     -1.663648
 C      6.819730     -0.466665      2.600382
 H      7.904137     -0.446517      2.626012
 C      4.053466     -0.477876      2.523447
 H      2.970024     -0.456451      2.487991
 C      6.100382     -0.356358      3.783674
 H      6.622667     -0.254875      4.728208
 C      4.705268     -0.369913      3.745875
 H      4.130142     -0.282238      4.660513
 C      7.034731     -1.737956     -0.732589
 C      7.027316     -0.736982      0.155619
 H      7.806918     -1.715398     -1.498651
 H      7.793077      0.028485      0.050989
 C      3.117551      0.458650     -0.177003
 C      2.390543      1.416599     -0.338104
 C      1.513065      2.516464     -0.557765
 C      0.701214      2.468206     -1.816605
 C      1.515782      3.564353      0.295822
 C     -0.701306      2.468251     -1.816566
```

C	1.384953	2.384518	-3.039353
C	0.706303	4.795915	0.211499
H	2.234055	3.535898	1.111328
C	-1.385108	2.384612	-3.039286
C	-1.513111	2.516548	-0.557694
H	2.469009	2.368304	-3.034574
C	0.697750	2.324687	-4.245293
C	-0.706221	4.795961	0.211531
C	1.384436	6.026674	0.250028
H	-2.469165	2.368469	-3.034453
C	-0.697967	2.324738	-4.245259
C	-1.515764	3.564447	0.295883
H	1.246255	2.272244	-5.178790
C	-1.384272	6.026764	0.250097
H	2.468770	6.024748	0.280117
C	0.698123	7.234562	0.245932
H	-1.246520	2.272334	-5.178730
H	-2.234018	3.536032	1.111407
H	-2.468605	6.024908	0.280237
C	-0.697880	7.234607	0.245967
H	1.246641	8.169393	0.259015
H	-1.246338	8.169473	0.259077
C	-2.390613	1.416709	-0.337999
C	-3.117607	0.458747	-0.176943
C	-3.988137	-0.647966	0.044447
C	-4.769888	-0.608116	1.323700
C	-3.992971	-1.672536	-0.837023
C	-6.175078	-0.617192	1.360220
C	-4.053534	-0.477811	2.523486
C	-4.777032	-2.921391	-0.780784
H	-3.289649	-1.608701	-1.663638
C	-6.819802	-0.466828	2.600402
C	-7.027357	-0.737061	0.155629
H	-2.970093	-0.456296	2.488035
C	-4.705353	-0.369922	3.745911
C	-6.186793	-2.952338	-0.742466
C	-4.072486	-4.134057	-0.886214
H	-7.904211	-0.446767	2.626025
C	-6.100470	-0.356489	3.783700
C	-7.034748	-1.738013	-0.732604
H	-7.793137	0.028391	0.051021
H	-4.130241	-0.282212	4.660554
C	-6.837353	-4.196215	-0.806523
H	-2.989675	-4.105575	-0.945779
C	-4.730995	-5.356843	-0.910539
H	-6.622768	-0.255073	4.728234
H	-7.806942	-1.715463	-1.498659
H	-7.922076	-4.218272	-0.804601
C	-6.125978	-5.388183	-0.870458
H	-4.162719	-6.277599	-0.976106
H	-6.654661	-6.333930	-0.904662

```
dbcot3_red2_k2c_b3lyp_6311+Gdp_thf (ID: tetralith_15604809)
    86
scf done: -3201.078019
 C    -9.113069     4.374748    -0.831381
 C    -9.199035     3.265750     0.001692
 C    -8.087265     2.445462     0.259819
 C    -6.841846     2.774274    -0.323337
 C    -6.767498     3.925204    -1.132994
 C    -7.882406     4.708797    -1.400574
 C    -5.585987     2.060241    -0.035102
 C    -5.307200     0.737224    -0.180647
 C    -6.260859    -0.261473    -0.773707
 C    -7.484872    -0.606792    -0.175273
 C    -8.253164    -1.638068    -0.744121
 C    -7.853834    -2.284768    -1.906959
 C    -6.652040    -1.920909    -2.516641
 C    -5.863457    -0.931245    -1.941961
 C    -8.007447     0.037110     1.050808
 C    -8.274841     1.336980     1.223673
 C    -4.026361     0.222488     0.136364
 C    -2.932060    -0.234604     0.433069
 C    -1.660038    -0.799478     0.716660
 C    -1.638087    -2.190030     0.379078
 C    -0.738592    -3.269025     0.272806
 C     0.726186    -3.256317     0.226174
 C     1.374535    -4.533848    -0.014516
 C     0.717798    -5.718393    -0.164464
 C    -0.712964    -5.732075    -0.109709
 C    -1.379024    -4.559725     0.086520
 C     1.615579    -2.165051     0.297648
 C     1.647085    -0.784909     0.675325
 C     0.722284     0.102307     1.394655
 C    -0.724730     0.099173     1.407715
 C    -1.360405     1.130535     2.175556
 C    -0.697285     2.097576     2.892219
 C     0.711004     2.097687     2.883971
 C     1.366520     1.132620     2.157917
 C     2.918440    -0.218439     0.389889
 C     4.013122     0.239120     0.096161
 C     5.298634     0.749133    -0.209862
 C     6.246866    -0.249362    -0.812027
 C     7.462958    -0.616042    -0.210174
 C     8.224237    -1.647341    -0.788382
 C     7.826286    -2.272945    -1.963173
 C     6.632840    -1.887460    -2.575986
 C     5.850608    -0.897805    -1.992598
 C     7.983932     0.005254     1.028029
 C     8.261037     1.300170     1.222106
 C     8.089637     2.425047     0.274363
 C     6.851644     2.774183    -0.312762
 C     6.793597     3.938063    -1.105002
 C     7.917437     4.715489    -1.352354
```

```
 C     9.140747     4.361621    -0.779290
 C     9.210585     3.239199     0.037094
 C     5.587348     2.067451    -0.044126
 H    10.151733     2.980054     0.511384
 H     5.838866     4.225335    -1.533233
 H    10.025243     4.963853    -0.953288
 H     7.839448     5.597510    -1.978296
 H     4.765319     2.708081     0.265944
 H     9.148427    -1.947460    -0.305382
 H     4.907576    -0.618456    -2.448742
 H     8.438742    -3.055805    -2.396190
 H     6.308340    -2.364621    -3.493960
 H     8.748999     1.565655     2.158016
 H     8.265600    -0.690018     1.816075
 H     2.614538    -2.474547     0.003692
 H     2.448297     1.136721     2.167090
 H    -2.442037     1.131040     2.200159
 H     1.276205     2.831652     3.448399
 H     2.459573    -4.525289    -0.067026
 H    -1.256129     2.830948     3.463664
 H    -2.647530    -2.514906     0.142337
 H    -2.464977    -4.570917     0.112353
 H     1.271993    -6.635752    -0.331336
 H    -1.260517    -6.660103    -0.234260
 H    -4.760272     2.698223     0.270649
 H    -4.914567    -0.668246    -2.395632
 H    -9.183802    -1.921409    -0.263393
 H    -8.298746    -0.644050     1.847627
 H    -6.326221    -2.414702    -3.425309
 H    -5.807119     4.197351    -1.558478
 H    -8.471656    -3.067183    -2.333094
 H    -8.765631     1.621074     2.152598
 H   -10.145930     3.022355     0.472886
 H    -7.791824     5.580321    -2.039402
 H    -9.990786     4.982064    -1.021311
 K    -0.083961    -1.027085    -1.994492
 K     0.120769    -2.416051     3.142509
---
dbcot3_red2_k2t_b3lyp_6311+Gdp_thf (ID: tetralith_16694030)
    86
scf done: -3201.081401
 C    -5.958928    -5.709692    -0.507406
 C    -6.768704    -4.584733    -0.605734
 C    -6.229370    -3.287213    -0.618797
 C    -4.828063    -3.134062    -0.565867
 C    -4.020441    -4.284098    -0.507567
 C    -4.572139    -5.557972    -0.458267
 C    -4.147718    -1.830233    -0.688937
 C    -4.292682    -0.734465     0.089169
 C    -5.168757    -0.651154     1.303757
 C    -6.567130    -0.782792     1.247072
 C    -7.313334    -0.580602     2.421254
```

C	-6.696633	-0.300666	3.634118
C	-5.306353	-0.191394	3.691044
C	-4.558096	-0.349267	2.530874
C	-7.314156	-1.084394	0.004998
C	-7.172725	-2.157154	-0.782341
C	-3.504007	0.422363	-0.177653
C	-2.850860	1.424883	-0.380183
C	-2.060230	2.578476	-0.649667
C	-2.217656	3.693922	0.097380
C	-1.518453	4.984787	-0.046485
C	-0.111525	5.118710	0.035428
C	0.434315	6.417144	0.023762
C	-0.361694	7.547873	-0.105423
C	-1.748855	7.410638	-0.193328
C	-2.312102	6.141811	-0.142614
C	0.807256	3.989702	0.264274
C	0.958768	2.864604	-0.483327
C	0.224693	2.641717	-1.774114
C	-1.168497	2.501407	-1.851280
C	-1.767092	2.251716	-3.096547
C	-1.007723	2.166654	-4.256638
C	0.378713	2.311361	-4.183118
C	0.982178	2.534968	-2.951785
C	1.912747	1.872131	-0.151521
C	2.720150	0.996052	0.118227
C	3.640134	-0.064123	0.359523
C	4.441558	0.064482	1.570255
C	5.469845	-0.837750	2.082049
C	6.114241	-0.449850	3.316494
C	5.832124	0.680595	4.032405
C	4.829526	1.552956	3.539483
C	4.186870	1.237218	2.371426
C	5.982118	-2.080657	1.577009
C	5.829971	-2.981027	0.514782
C	5.057895	-3.141030	-0.666911
C	4.027211	-2.254269	-1.200873
C	3.406704	-2.692221	-2.441738
C	3.724417	-3.839958	-3.107444
C	4.734547	-4.700499	-2.579527
C	5.351858	-4.340156	-1.414621
C	3.519424	-1.026209	-0.715562
H	6.121455	-4.990197	-1.006350
H	2.636112	-2.047125	-2.854430
H	5.005796	-5.618288	-3.089659
H	3.211574	-4.093165	-4.029738
H	2.755389	-0.667552	-1.396611
H	6.880683	-1.120987	3.692650
H	3.425623	1.918014	2.017438
H	6.363875	0.897261	4.952756
H	4.567223	2.460124	4.073676
H	6.518018	-3.815018	0.637828
H	6.751553	-2.452431	2.248493

```
 H     1.491095     4.118809     1.099602
 H     2.060114     2.630116    -2.887785
 H    -2.843109     2.126745    -3.145882
 H     0.985196     2.243876    -5.079345
 H     1.509883     6.526817     0.114918
 H    -1.491718     1.985006    -5.209554
 H    -2.987681     3.667313     0.864751
 H    -3.391128     6.032300    -0.176272
 H     0.094027     8.531566    -0.125958
 H    -2.384038     8.284488    -0.282921
 H    -3.393054    -1.777946    -1.469843
 H    -3.480879    -0.231159     2.565592
 H    -8.394520    -0.655302     2.371795
 H    -8.129919    -0.401877    -0.223027
 H    -4.810903     0.030747     4.629262
 H    -2.942211    -4.164702    -0.499070
 H    -7.294939    -0.162007     4.527449
 H    -7.884160    -2.271695    -1.597433
 H    -7.845065    -4.702762    -0.673694
 H    -3.926646    -6.426794    -0.398359
 H    -6.403874    -6.697959    -0.485700
 K     2.958061    -2.767641     1.724540
 K     6.467528    -0.275906    -0.897258
---
dbcot3_red4_k4tt_b3lyp_6311+Gdp_thf (ID: tetralith_17094502)
   88
scf done: -4401.05136998
 6    -5.197259   -4.740951   -2.427235
 6    -5.818796   -4.348632   -1.275014
 6    -5.508109   -3.143101   -0.544077
 6    -4.456297   -2.285038   -1.084497
 6    -3.832109   -2.756722   -2.310798
 6    -4.165070   -3.909822   -2.959734
 6    -3.933703   -1.055004   -0.618769
 6    -4.051866   -0.069293    0.433997
 6    -4.839112    0.079372    1.650603
 6    -5.881487   -0.800024    2.175933
 6    -6.510392   -0.390778    3.411674
 6    -6.199783    0.737268    4.119439
 6    -5.180479    1.584674    3.616232
 6    -4.552800    1.249656    2.445530
 6    -6.422411   -2.034182    1.681028
 6    -6.286545   -2.949283    0.628283
 6    -3.147977    0.998065    0.154054
 6    -2.366876    1.884349   -0.154782
 6    -1.467547    2.906818   -0.551091
 6    -1.425966    4.104065    0.088934
 6    -0.596045    5.279854   -0.227674
 6     0.820036    5.255119   -0.277937
 6     1.507860    6.470237   -0.462589
 6     0.840174    7.677400   -0.626334
 6    -0.555587    7.702068   -0.575556
```

6	-1.251961	6.518964	-0.363317
6	1.626696	4.051426	-0.009188
6	1.588753	2.850370	-0.642242
6	0.720311	2.590991	-1.838699
6	-0.681895	2.623572	-1.798783
6	-1.402830	2.348187	-2.972757
6	-0.758962	2.064976	-4.170636
6	0.635990	2.029917	-4.209816
6	1.359492	2.280458	-3.050741
6	2.479303	1.804456	-0.288493
6	3.251055	0.904544	0.003408
6	4.119434	-0.190184	0.290391
6	5.121667	0.058763	1.318155
6	6.156889	-0.842497	1.821011
6	7.028517	-0.317267	2.848126
6	6.953875	0.940192	3.379710
6	5.945639	1.812571	2.897228
6	5.092406	1.370019	1.921214
6	6.486341	-2.197280	1.479538
6	6.089587	-3.245659	0.638339
6	5.116106	-3.524292	-0.357895
6	4.077007	-2.640493	-0.881279
6	3.223806	-3.217286	-1.908553
6	3.336333	-4.488944	-2.390128
6	4.354936	-5.346489	-1.873535
6	5.187652	-4.857883	-0.906204
6	3.750328	-1.301607	-0.559175
1	5.965691	-5.504656	-0.508809
1	2.444752	-2.575570	-2.310492
1	4.466407	-6.361226	-2.239774
1	2.656440	-4.841372	-3.159047
1	2.910613	-0.987426	-1.169387
1	7.798827	-0.988313	3.216595
1	4.333031	2.054520	1.570588
1	7.649468	1.255315	4.150192
1	5.843555	2.818811	3.289928
1	6.726625	-4.112183	0.804032
1	7.331364	-2.525305	2.079170
1	2.395455	4.178439	0.749558
1	2.442704	2.240281	-3.071659
1	-2.486163	2.359214	-2.932314
1	1.154358	1.804595	-5.135208
1	2.592966	6.453552	-0.474402
1	-1.339122	1.867438	-5.065184
1	-2.145195	4.254467	0.890640
1	-2.335069	6.540489	-0.297751
1	1.401465	8.592556	-0.779042
1	-1.093778	8.636714	-0.688142
1	-3.165121	-0.716745	-1.305222
1	-3.776625	1.910002	2.085096
1	-7.289169	-1.043001	3.795884
1	-7.202252	-2.381718	2.353580

```
 1    -4.893102    2.487073    4.145618
 1    -3.045507   -2.133577   -2.726976
 1    -6.721297    0.970531    5.041628
 1    -6.994965   -3.765317    0.756192
 1    -6.605024   -4.976486   -0.863622
 1    -3.648199   -4.189006   -3.872197
 1    -5.481694   -5.661972   -2.924274
19     3.506249   -2.533444    2.216809
19     6.673986   -0.897892   -1.318357
19    -6.870213   -0.254689   -0.814583
19    -3.423780   -2.777869    1.836289
---
dbcot3_red6_k6_b3lyp_6311+Gdp_thf_ctd2 (ID: te-
tralith_15662248)
   90
scf done: -5600.994640
 C    -8.167207    -4.950824    -1.244019
 C    -8.809692    -3.830835    -0.794240
 C    -8.159345    -2.581823    -0.472739
 C    -6.707385    -2.526834    -0.636736
 C    -6.084505    -3.745348    -1.121293
 C    -6.750604    -4.901829    -1.414303
 C    -5.785253    -1.470975    -0.403507
 C    -5.675846    -0.108011     0.049832
 C    -6.600810     0.916457     0.493828
 C    -8.062901     0.851968     0.622629
 C    -8.723968     2.056105     1.077331
 C    -8.100480     3.228804     1.401333
 C    -6.685132     3.288847     1.290233
 C    -6.003042     2.182526     0.859983
 C    -8.988912    -0.212727     0.381599
 C    -9.037944    -1.553736    -0.038321
 C    -4.282828     0.273057     0.041295
 C    -3.087350     0.511981     0.013261
 C    -1.695360     0.900938    -0.024061
 C    -1.627454     2.325177    -0.162627
 C    -0.722298     3.413038    -0.102561
 C     0.722177     3.413010     0.102923
 C     1.351320     4.711198     0.243953
 C     0.700996     5.907911     0.134627
 C    -0.701097     5.907934    -0.134053
 C    -1.351429     4.711236    -0.243491
 C     1.627350     2.325146     0.162885
 C     1.695246     0.900875     0.024596
 C     0.735592    -0.189473    -0.054408
 C    -0.735755    -0.189409     0.055656
 C    -1.339652    -1.491856     0.235675
 C    -0.692016    -2.695015     0.149320
 C     0.691785    -2.695135    -0.146422
 C     1.339455    -1.492056    -0.233571
 C     3.087221     0.511917    -0.013065
 C     4.282700     0.273039    -0.041440
```

C	5.675708	-0.108058	-0.050265
C	6.600606	0.916426	-0.494372
C	8.062672	0.851930	-0.623467
C	8.723618	2.055954	-1.078655
C	8.100064	3.228628	-1.402617
C	6.684765	3.288768	-1.290946
C	6.002780	2.182521	-0.860347
C	8.988796	-0.212624	-0.382198
C	9.037968	-1.553457	0.038252
C	8.159419	-2.581556	0.472769
C	6.707386	-2.526823	0.636262
C	6.084516	-3.745550	1.120296
C	6.750736	-4.901851	1.413734
C	8.167473	-4.950433	1.244448
C	8.809922	-3.830315	0.794940
C	5.785182	-1.471042	0.402975
H	9.889073	-3.861828	0.666575
H	5.006010	-3.720686	1.251105
H	8.723835	-5.854645	1.467534
H	6.205795	-5.770374	1.769296
H	4.788221	-1.819629	0.650072
H	9.805343	2.006059	-1.167101
H	4.926326	2.249992	-0.781708
H	8.675585	4.085445	-1.737155
H	6.144004	4.196225	-1.540029
H	10.067689	-1.905707	0.030412
H	9.998370	0.126581	-0.600831
H	2.633243	2.705485	0.307391
H	2.403201	-1.506383	-0.424651
H	-2.403397	-1.506040	0.426774
H	1.240783	-3.621213	-0.284089
H	2.421864	4.714215	0.430851
H	-1.241040	-3.620981	0.287625
H	-2.633320	2.705468	-0.307442
H	-2.421970	4.714262	-0.430402
H	1.245747	6.840332	0.239938
H	-1.245835	6.840371	-0.239281
H	-4.788294	-1.819434	-0.650781
H	-4.926556	2.249935	0.781722
H	-9.805725	2.006259	1.165412
H	-9.998537	0.126473	0.600009
H	-6.144417	4.196291	1.539463
H	-5.006089	-3.720196	-1.252788
H	-8.676089	4.085697	1.735526
H	-10.067605	-1.906157	-0.030072
H	-9.888756	-3.862623	-0.665209
H	-6.205666	-5.770195	-1.770254
H	-8.723446	-5.855232	-1.466619
K	7.623968	-0.062085	2.347385
K	7.079719	-1.600128	-2.339456
K	-0.164897	1.624540	2.505408
K	0.165516	1.624331	-2.504906

```
 K    -7.623818    -0.062533    -2.348052
 K    -7.079513    -1.599627     2.339264
---
dbcot3_red6_k6_twisted_b3lyp_6311+Gdp_thf_ctd (ID: tetralith
166939759)
  6    -9.200014    4.053040   -0.588087
  6    -9.530286    2.756969   -0.871392
  6    -8.616350    1.640477   -0.801934
  6    -7.240794    1.931282   -0.400562
  6    -6.952576    3.325076   -0.114266
  6    -7.858669    4.344437   -0.194345
  6    -6.117582    1.074822   -0.242940
  6    -5.681928   -0.295078   -0.338374
  6    -6.305474   -1.561198   -0.671935
  6    -7.692967   -1.834114   -1.071698
  6    -8.023702   -3.214236   -1.354992
  6    -7.158148   -4.269282   -1.276833
  6    -5.817515   -4.008312   -0.885005
  6    -5.442811   -2.721310   -0.606453
  6    -8.818190   -0.965698   -1.241406
  6    -9.190067    0.386404   -1.140182
  6    -4.275468   -0.361441   -0.003704
  6    -3.086930   -0.337713    0.264626
  6    -1.695288   -0.332719    0.667360
  6    -1.634738   -0.534451    2.083871
  6    -0.726372   -0.734357    3.150883
  6     0.732237   -0.756177    3.143395
  6     1.377300   -1.074473    4.402897
  6     0.720862   -1.308287    5.577067
  6    -0.706330   -1.240843    5.594299
  6    -1.366800   -0.965728    4.431358
  6     1.636679   -0.489229    2.087731
  6     1.691837   -0.100822    0.709997
  6     0.728701    0.087985   -0.367279
  6    -0.733093   -0.110254   -0.404724
  6    -1.330341   -0.050025   -1.722020
  6    -0.689668    0.293735   -2.881425
  6     0.682007    0.633247   -2.816642
  6     1.323986    0.522359   -1.613154
  6     3.079957    0.107868    0.351111
  6     4.265740    0.278042    0.127162
  6     5.671143    0.415348   -0.189594
  6     6.233101    1.734690    0.027458
  6     7.604036    2.208215   -0.208857
  6     7.869773    3.597052    0.099678
  6     6.958019    4.488704    0.591528
  6     5.633228    4.031644    0.825760
  6     5.319021    2.728037    0.549285
  6     8.766415    1.535605   -0.704050
  6     9.200394    0.277323   -1.156983
  6     8.687824   -1.030679   -1.363502
  6     7.330382   -1.521486   -1.130339
```

```
  6      7.109121   -2.923093   -1.437760
  6      8.060226   -3.779325   -1.915917
  6      9.383342   -3.292536   -2.142704
  6      9.651525   -1.979868   -1.870264
  6      6.170134   -0.854518   -0.651974
  1     10.657515   -1.604611   -2.041156
  1      6.105099   -3.303995   -1.270602
  1     10.159835   -3.948095   -2.522399
  1      7.809233   -4.815098   -2.120624
  1      5.348362   -1.561616   -0.627091
  1      8.883718    3.943599   -0.079940
  1      4.305438    2.399445    0.732984
  1      7.244528    5.515125    0.795680
  1      4.874239    4.701433    1.217553
  1     10.252996    0.325390   -1.429348
  1      9.600684    2.231794   -0.742923
  1      2.652070   -0.575121    2.458681
  1      2.377183    0.760598   -1.580571
  1     -2.383320   -0.284784   -1.778900
  1      1.225781    0.961709   -3.696803
  1      2.463326   -1.114322    4.395010
  1     -1.233843    0.313854   -3.820441
  1     -2.648940   -0.589579    2.463922
  1     -2.452762   -0.925809    4.444572
  1      1.276056   -1.533546    6.481631
  1     -1.258270   -1.411511    6.512693
  1     -5.265404    1.672524    0.060860
  1     -4.418513   -2.542905   -0.309494
  1     -9.049696   -3.410857   -1.653473
  1     -9.682439   -1.547059   -1.553216
  1     -5.093863   -4.813407   -0.805436
  1     -5.934237    3.558551    0.185058
  1     -7.492152   -5.275465   -1.507776
  1    -10.240770    0.501344   -1.399503
  1    -10.550345    2.530245   -1.171567
  1     -7.558705    5.361271    0.037590
  1     -9.941784    4.841230   -0.661716
 19      8.040202   -0.267001    1.660781
 19      6.809410    0.956597   -2.992228
 19      0.095406   -2.813615    0.865369
 19     -0.095429    2.080311    1.883487
 19     -8.039209   -0.311529    1.648385
 19     -6.810398    0.397817   -3.109913
{Constraints: {D 7 8 19 20 F; D 27 28 37 52 F}}
---
```

XIII. *Copies of COSY, ^{13}C-HSQC, and ^{13}C-HMBC for dbCOT2, K_2dbCOT2, K_4dbCOT2, K_2dbCOT3, K_4dbCOT3, K_6dbCOT3, and K_6dbCOT5. Excitation transfer spectra for K_2dbCOT3.*

dbCOT2
COSY spectrum
$CDCl_3$, 500 MHz, 373.2 K

Strand 2022

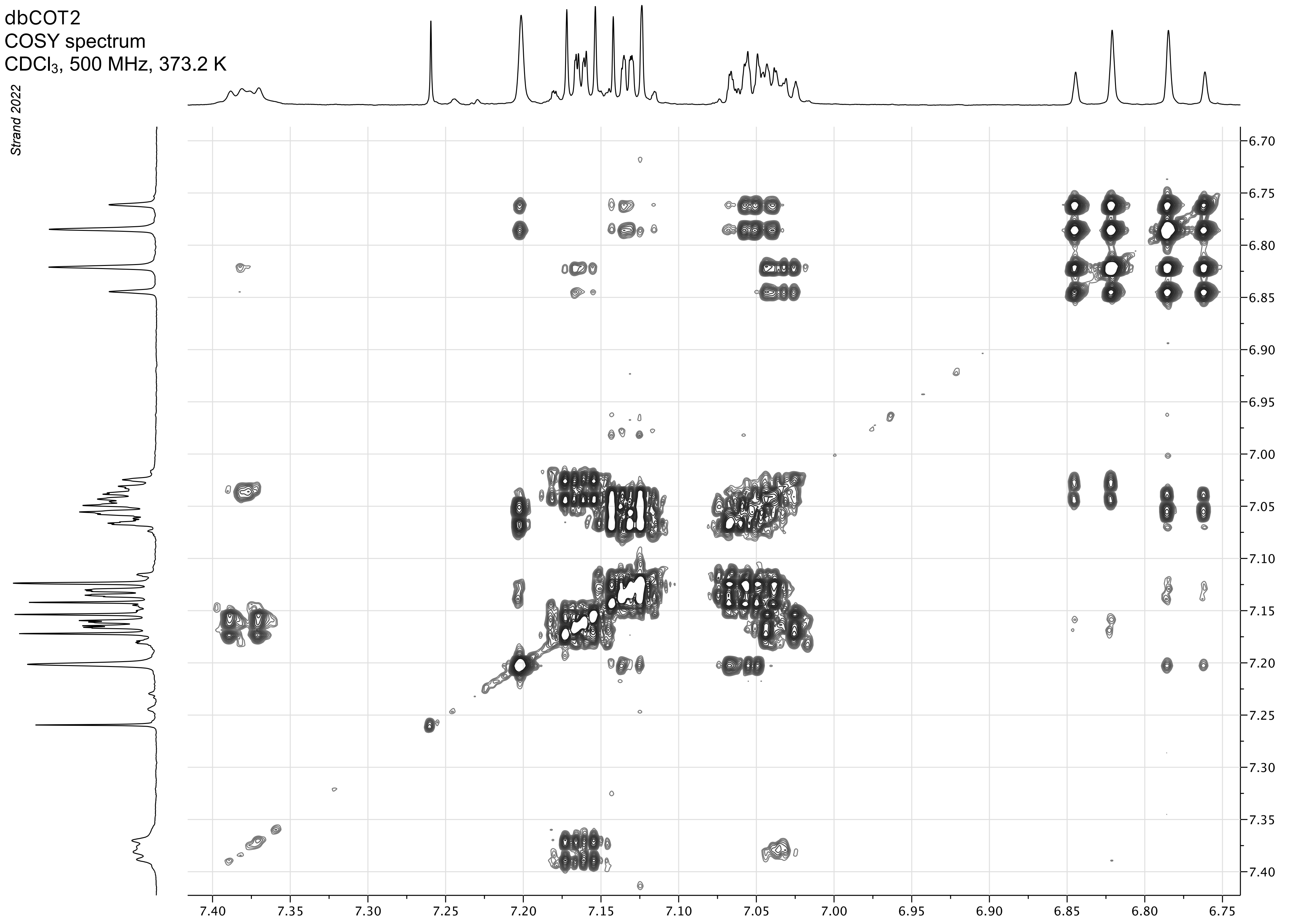

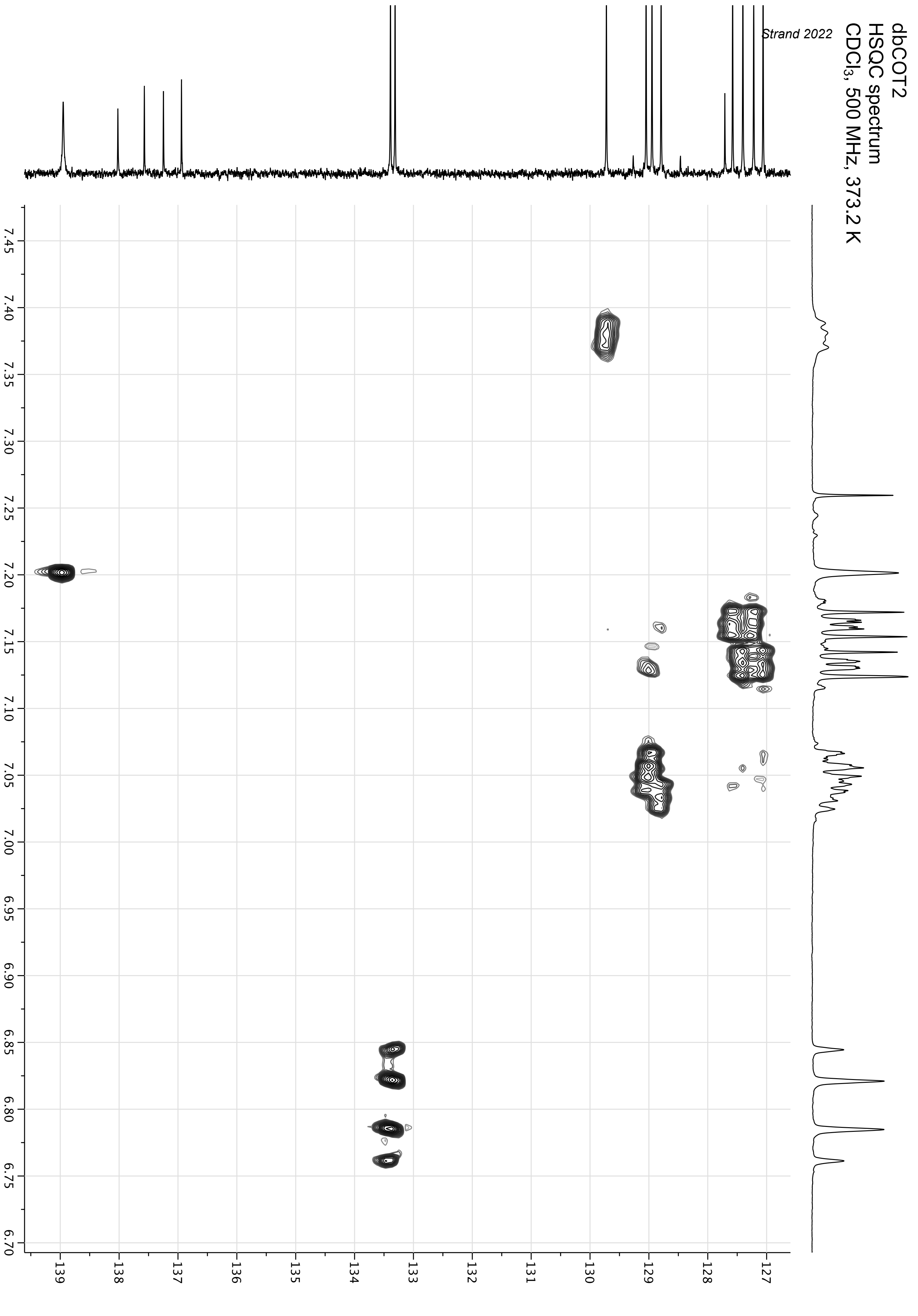
dbCOT2
HSQC spectrum
CDCl3, 500 MHz, 373.2 K
Strand 2022
f1 (ppm)

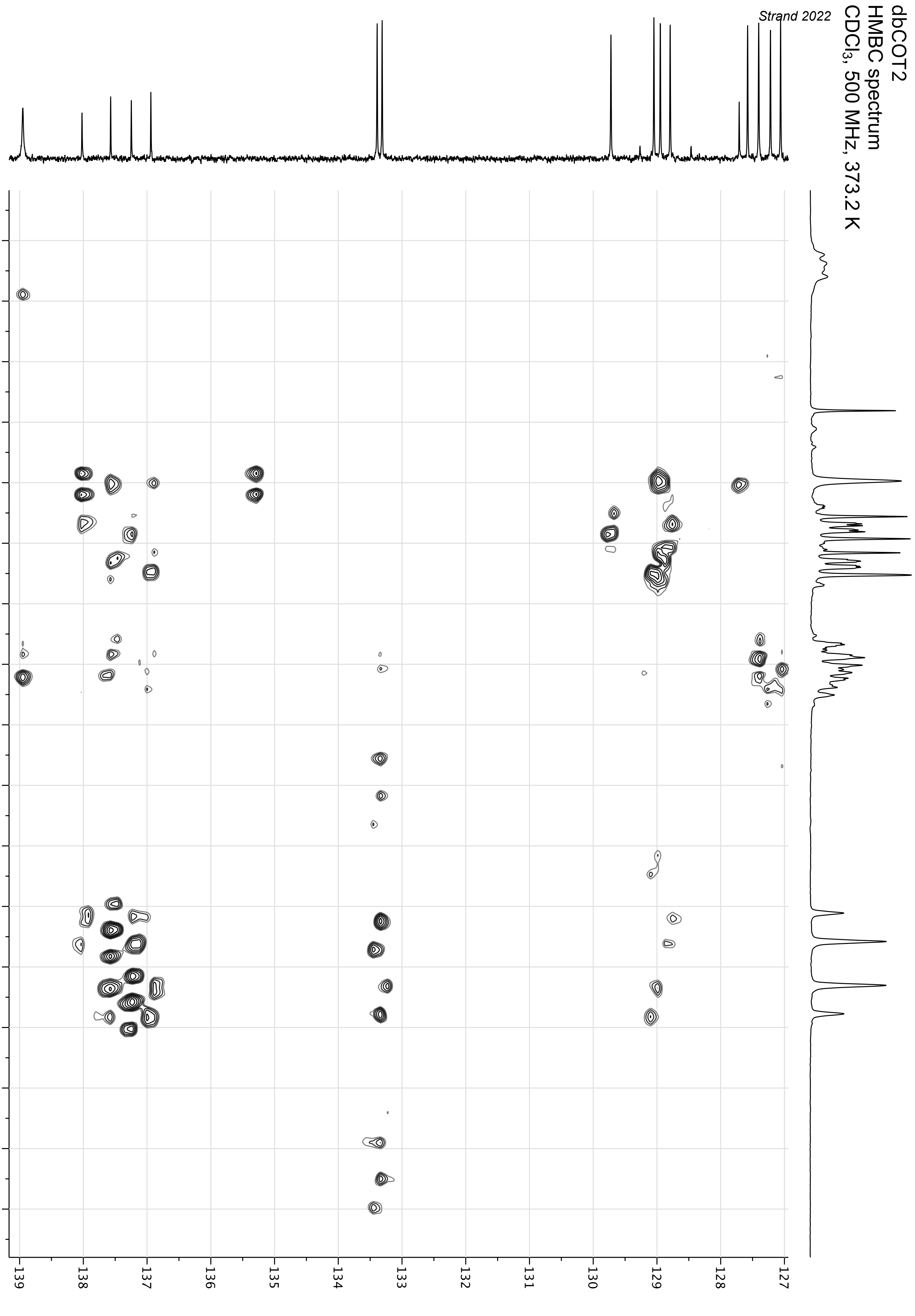
dbCOT2
HMBC spectrum
$CDCl_3$, 500 MHz, 373.2 K
Strand 2022
f1 (ppm)

K_2dbCOT2
COSY spectrum
THF-d8, 500 MHz, 237.0 K

Strand 2022

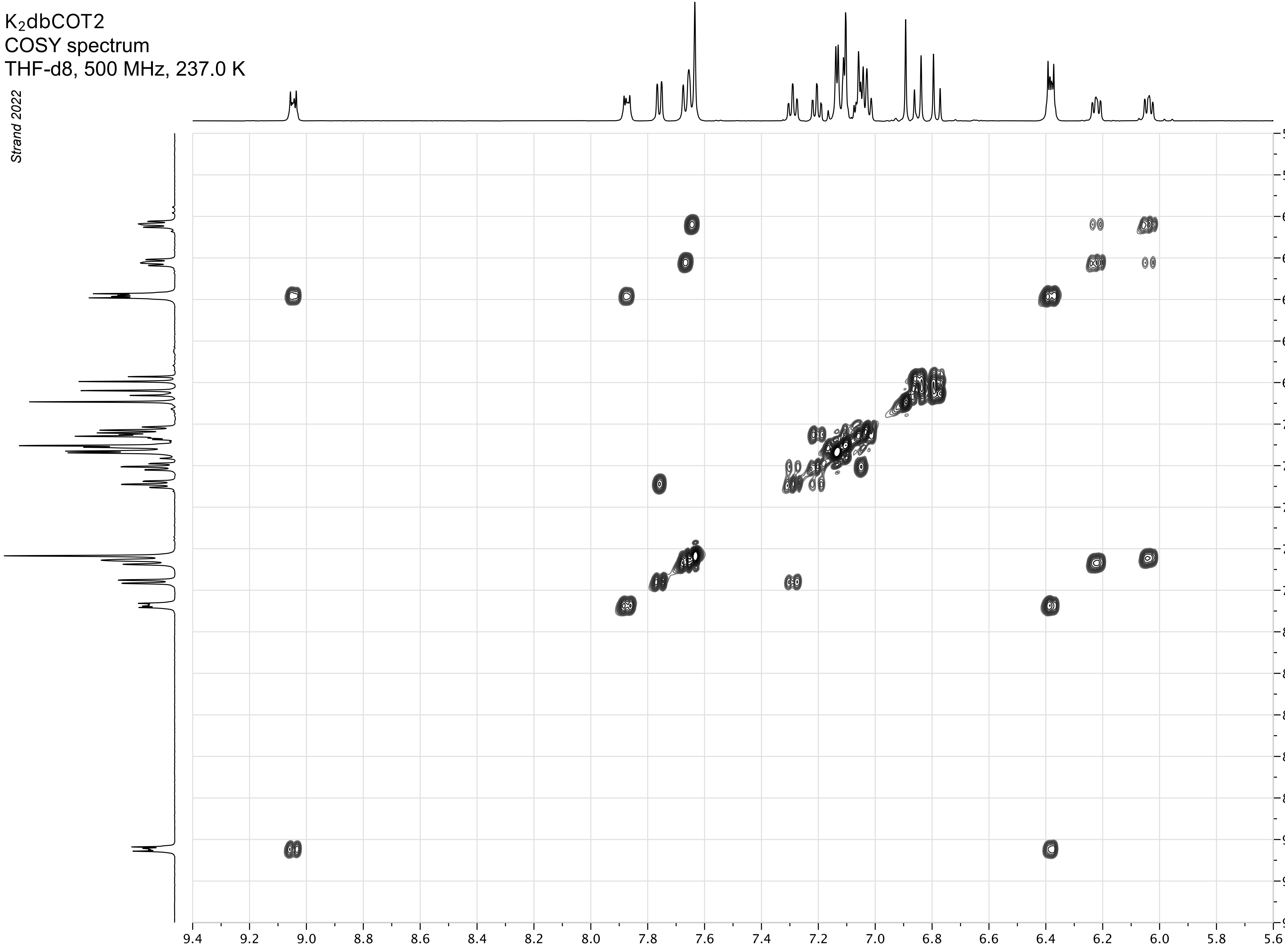

K$_2$dbCOT2
^{13}C-HSQC spectrum
THF-d8, 500 MHz, 237.0 K

Strand 2022

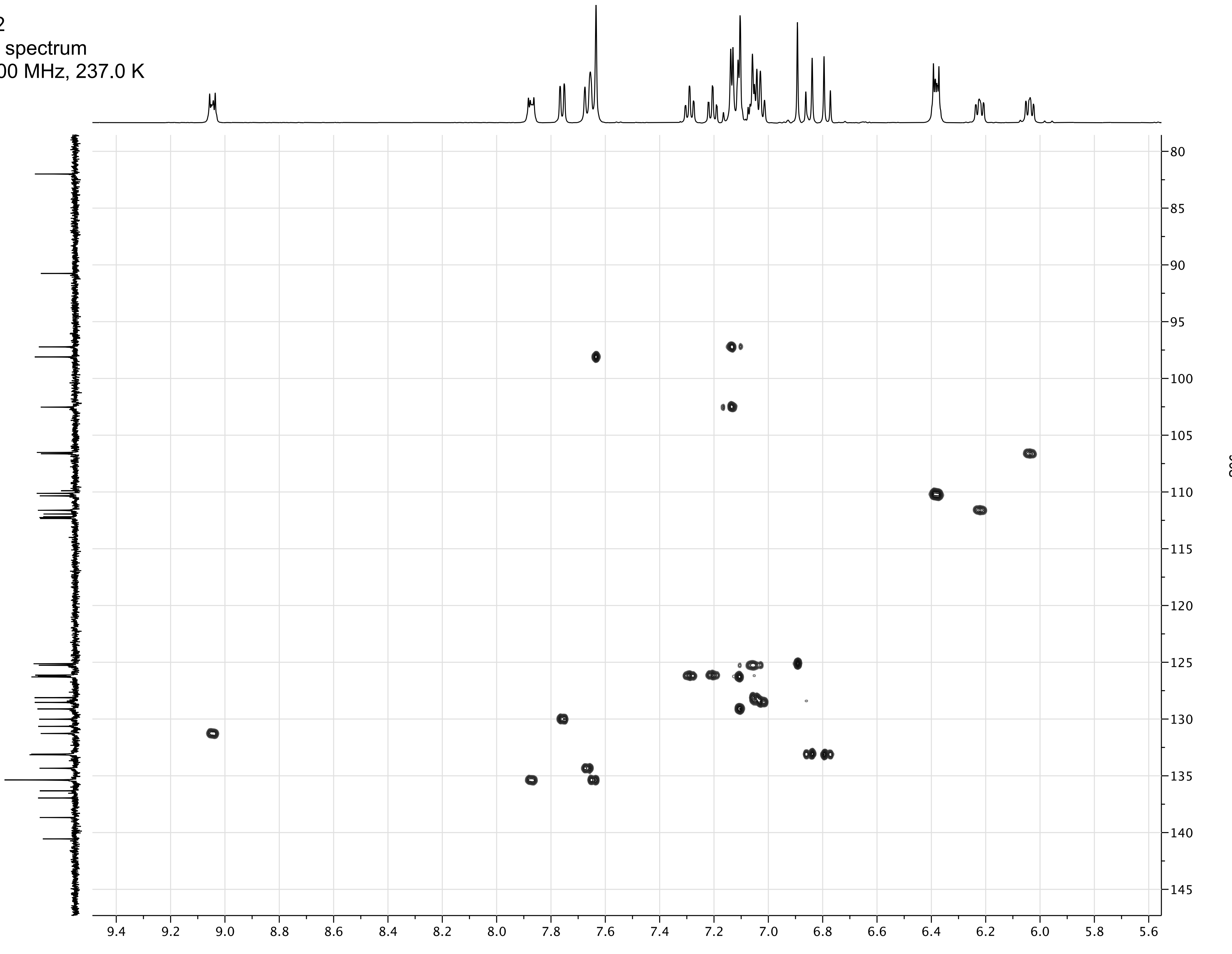

K$_2$dbCOT2
^{13}C-HMBC spectrum
THF-d8, 500 MHz, 237.0 K

Strand 2022

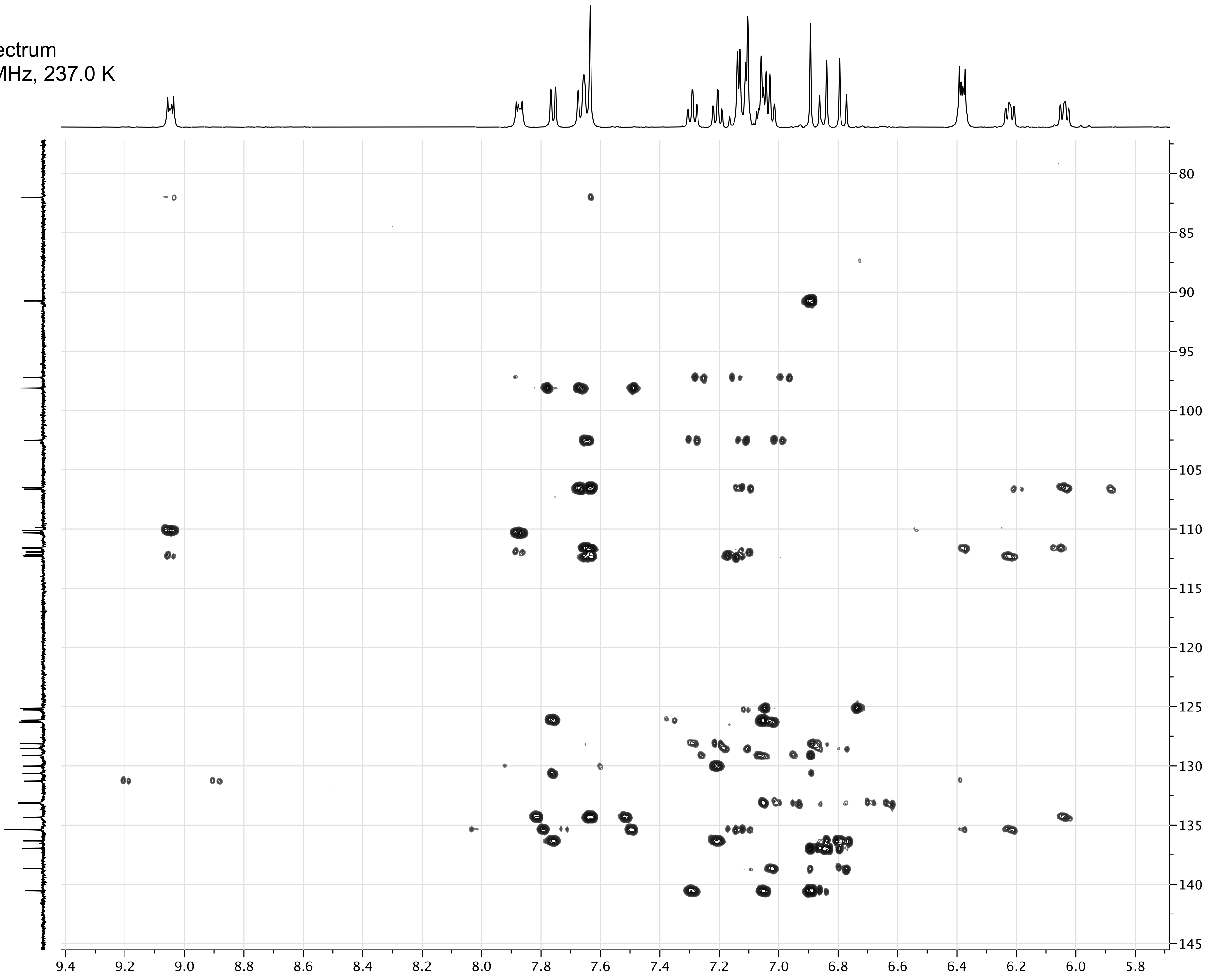

K$_4$dbCOT2
COSY spectrum
THF-d8, 500 MHz

Strand 2022

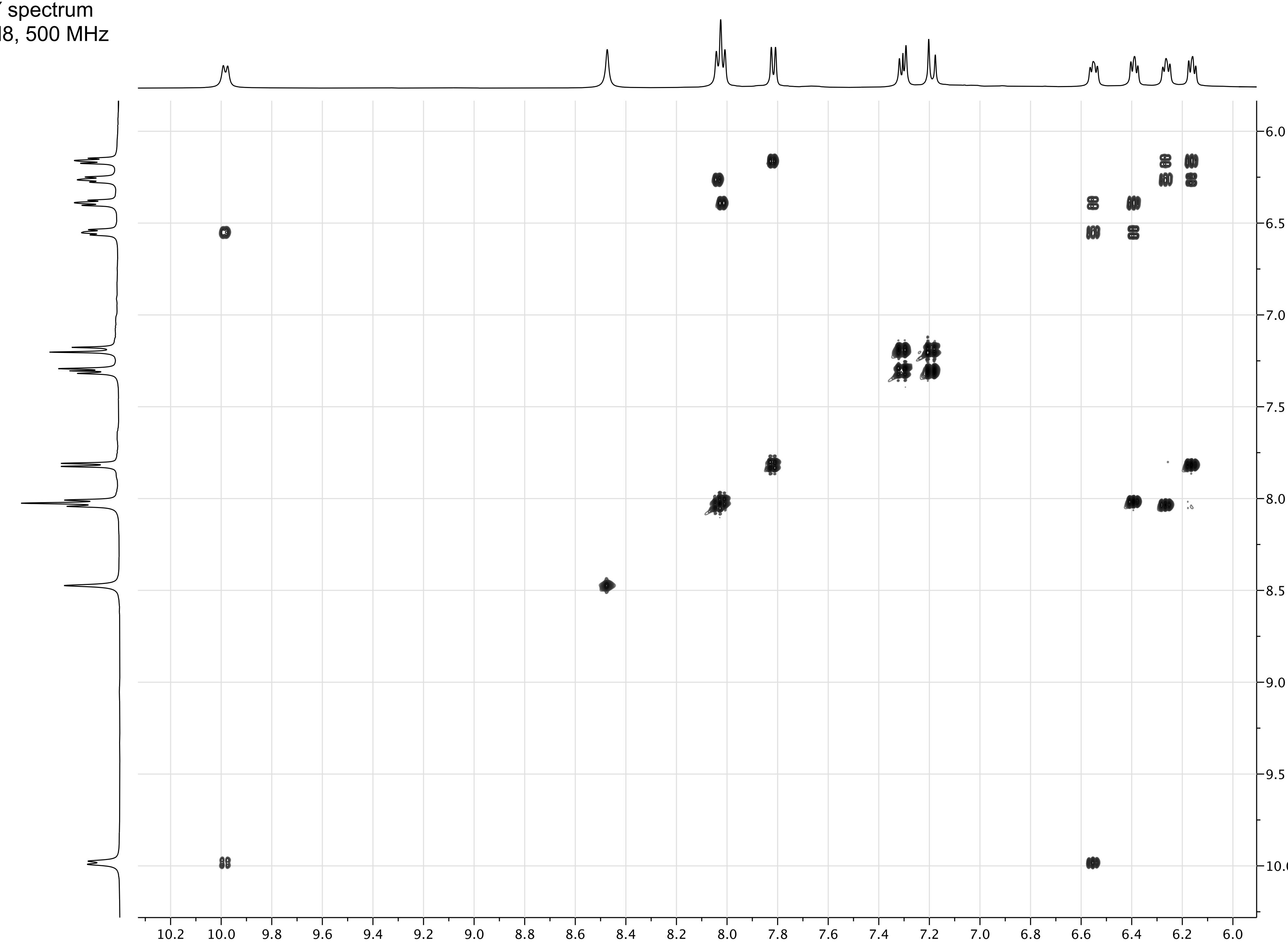

K_4dbCOT2
^{13}C-HSQC spectrum
THF-d8, 500 MHz

Strand 2022

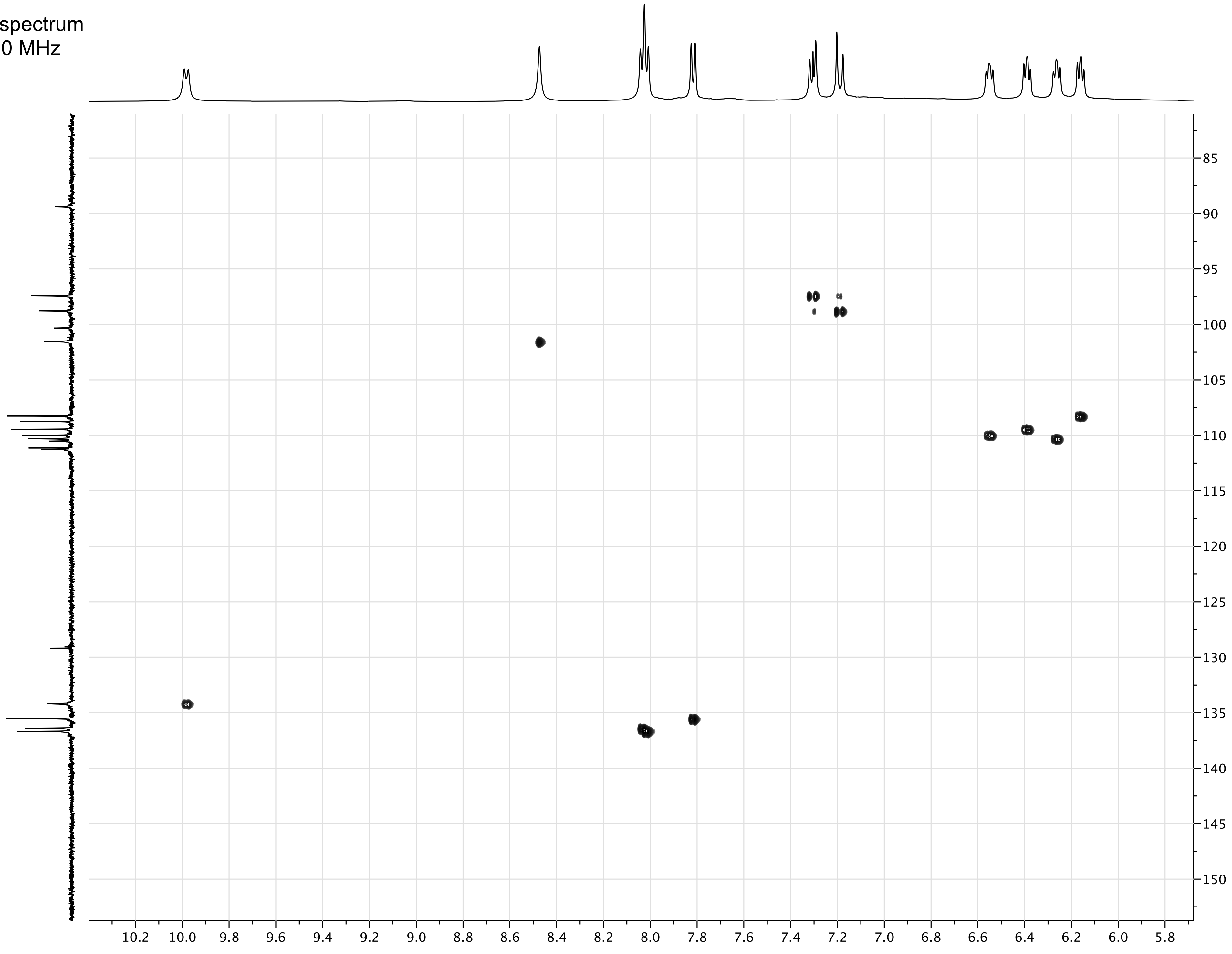

K_4dbCOT2
^{13}C-HMBC spectrum
THF-d8, 500 MHz

Strand 2022

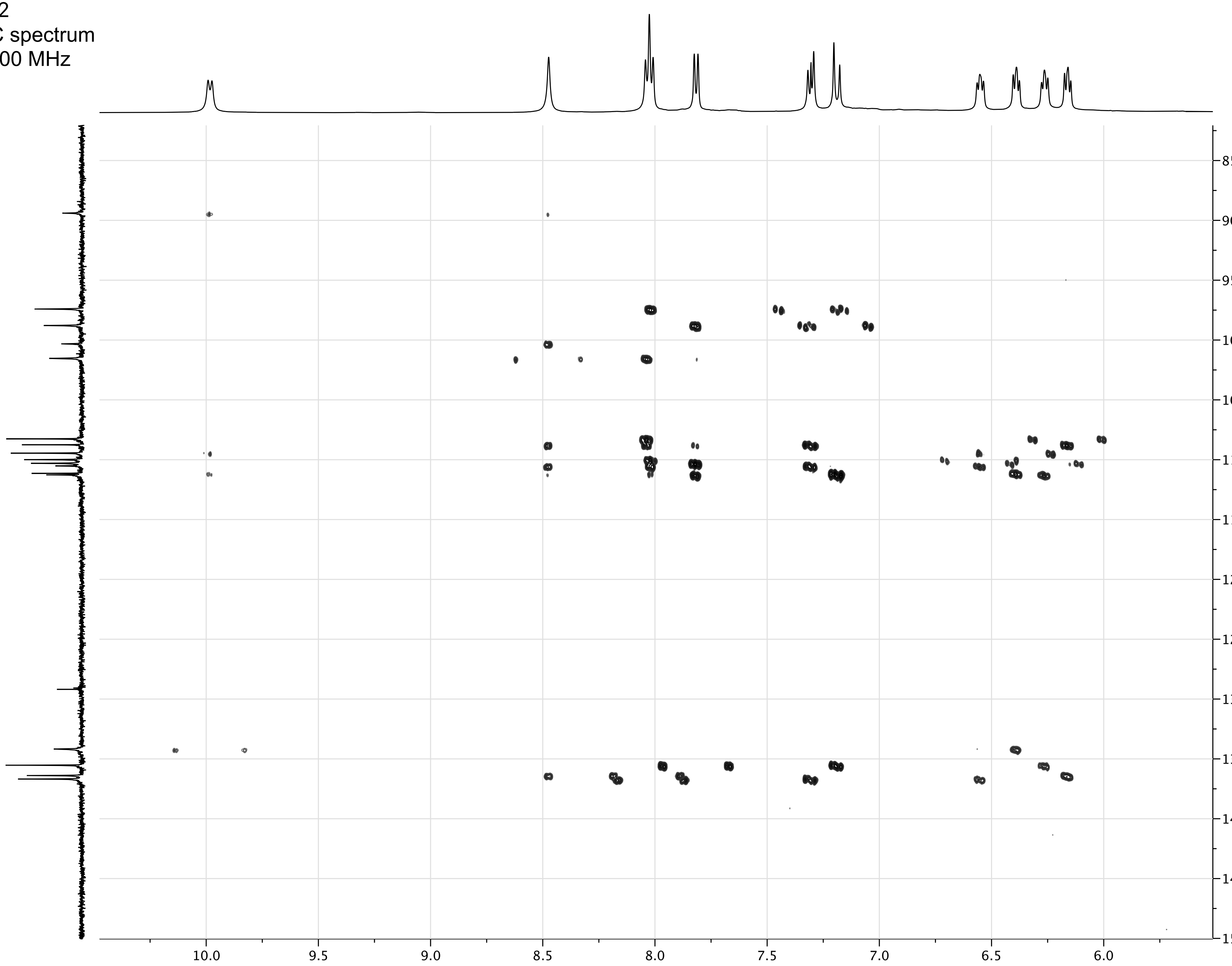

K2dbCOT3
COSY spectrum
THF-d8, 500 MHz, 223.9 K

Strand 2022

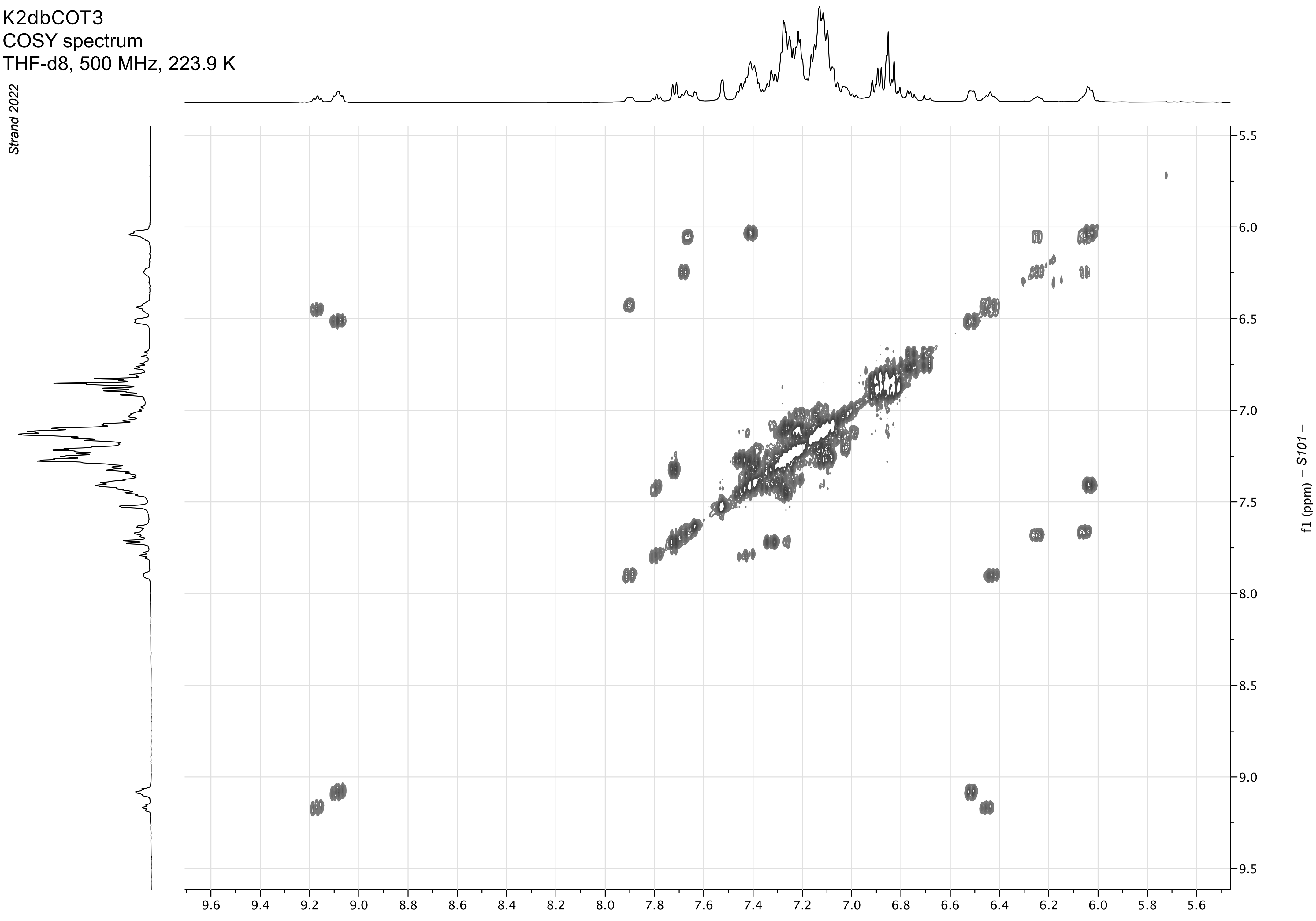

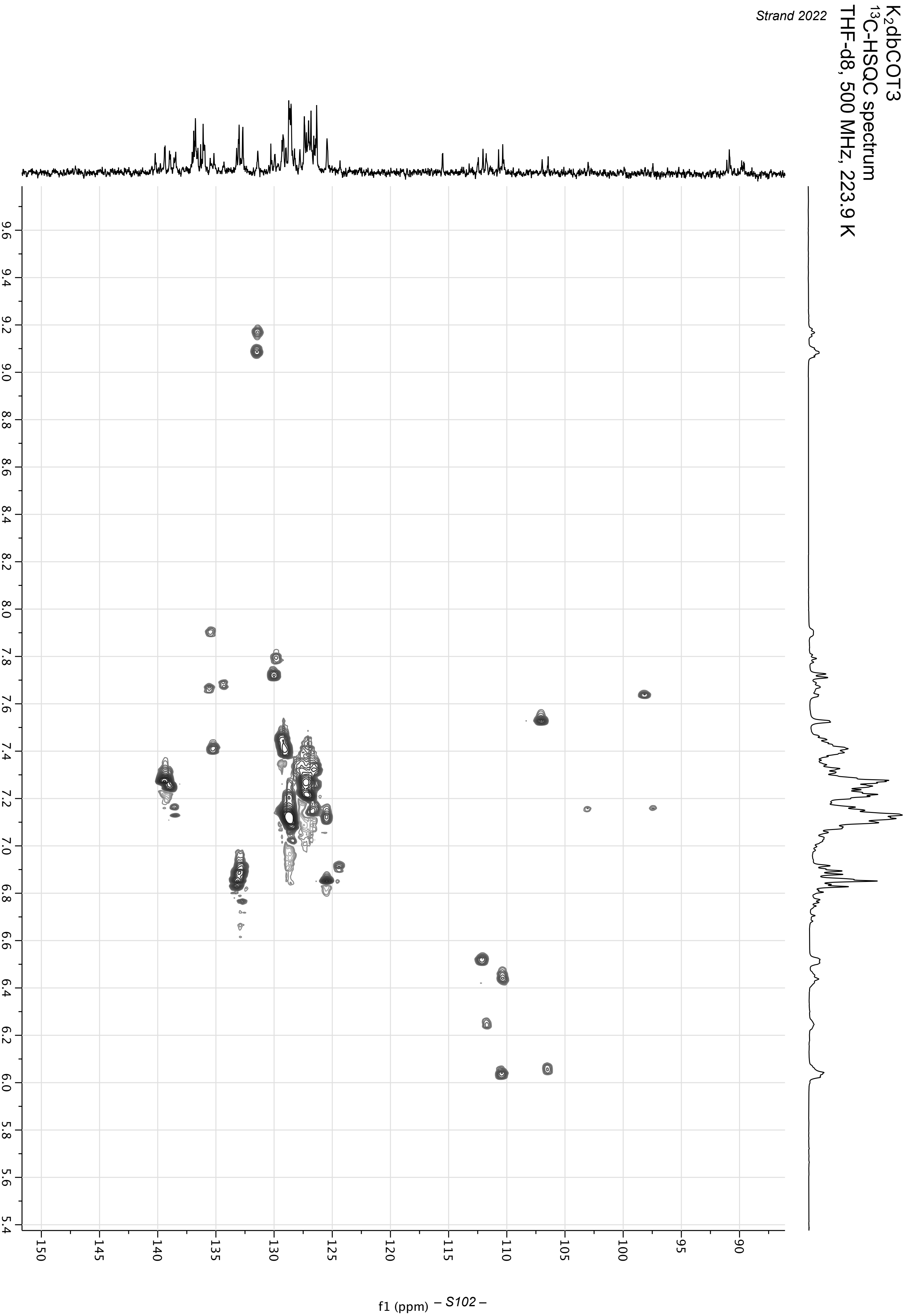
K2dbCOT3
13C-HSQC spectrum
THF-d8, 500 MHz, 223.9 K
Strand 2022
f1 (ppm)

K$_2$dbCOT3
^{13}C-HMBC spectrum
THF-d8, 500 MHz, 223.9 K

Strand 2022

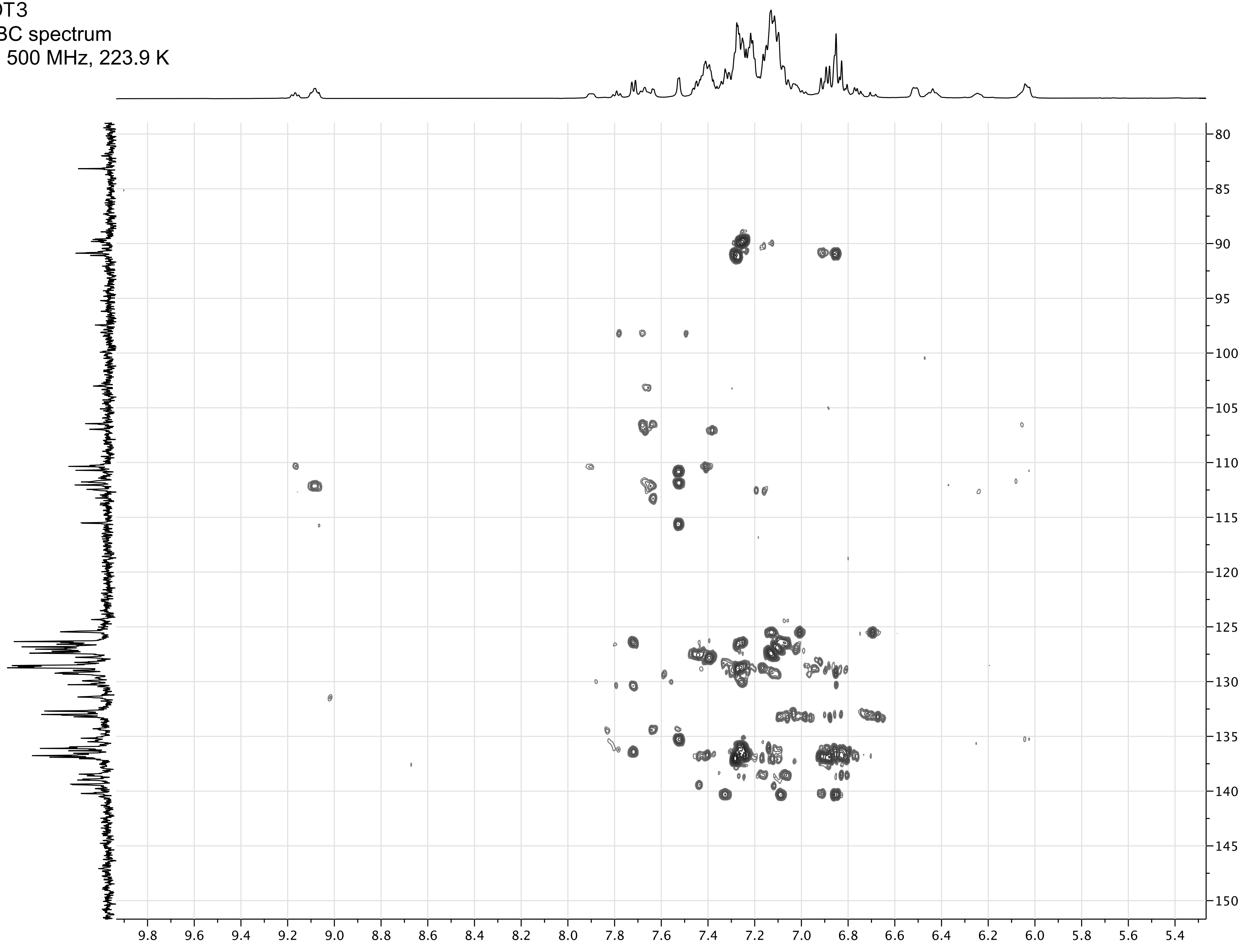

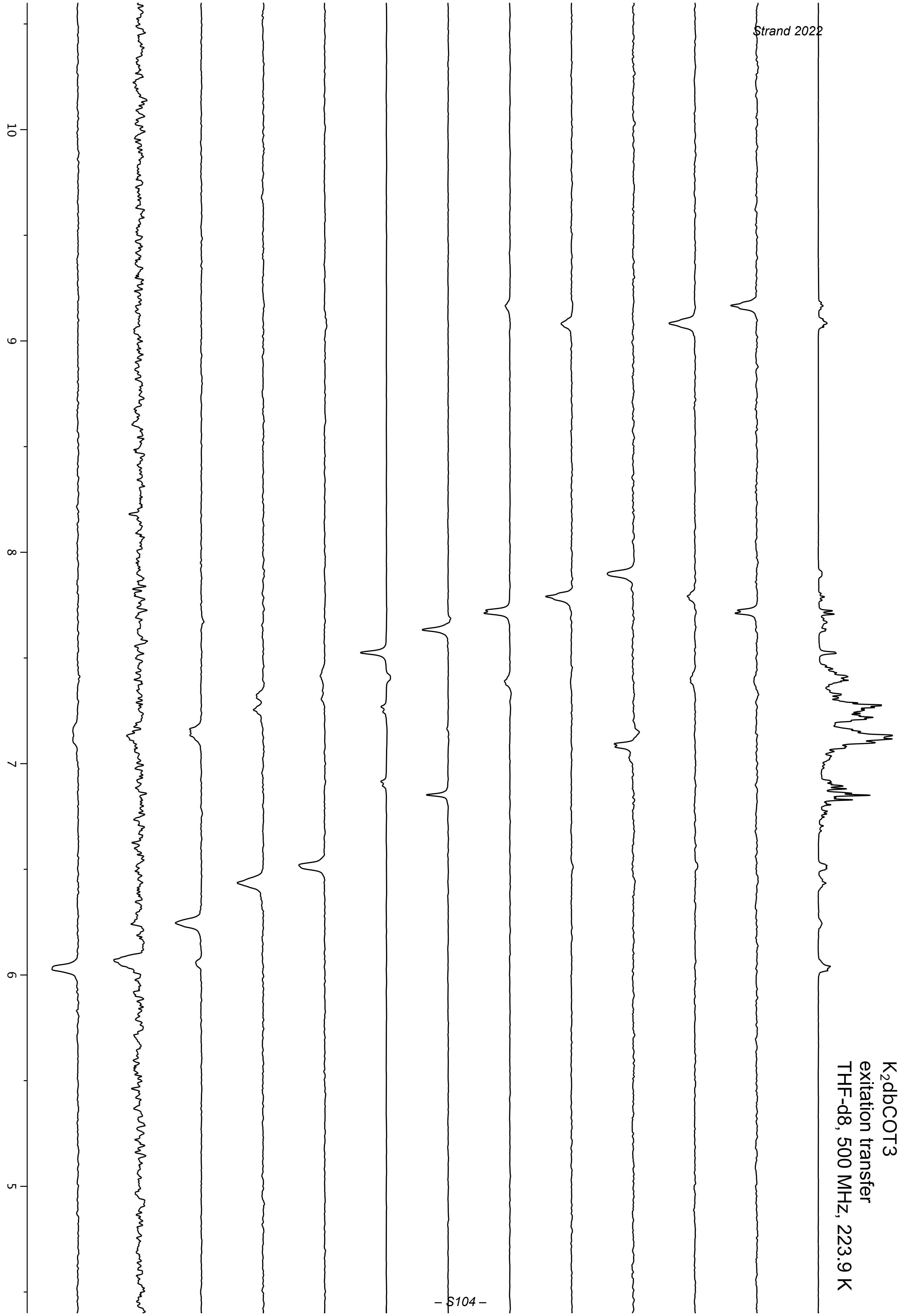
K2dbCOT3
exitation transfer
THF-d8, 500 MHz, 223.9 K
10
9
8
7
6
5

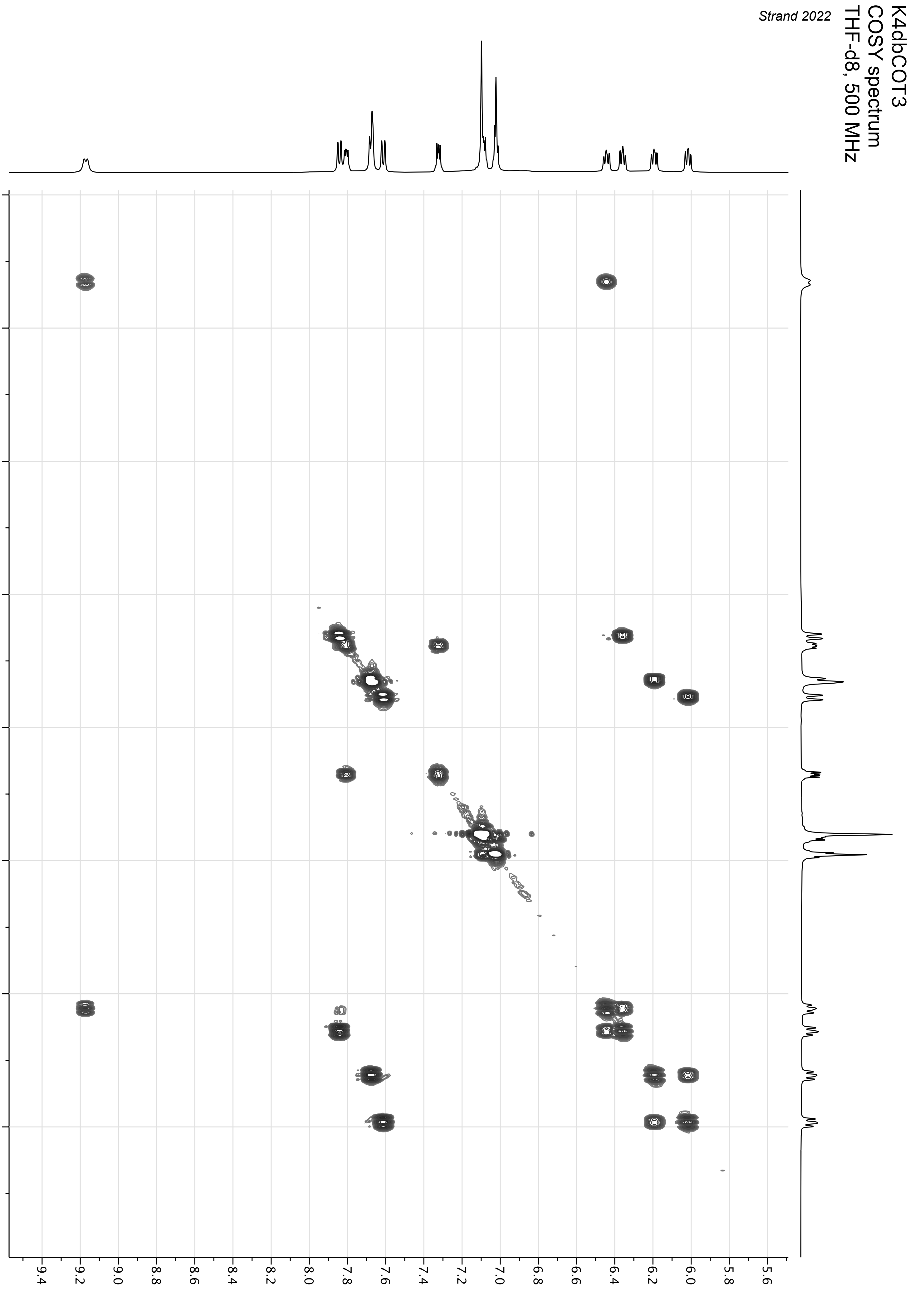
K4dbCOT3
COSY spectrum
THF-d8, 500 MHz
Strand 2022
f1 (ppm)

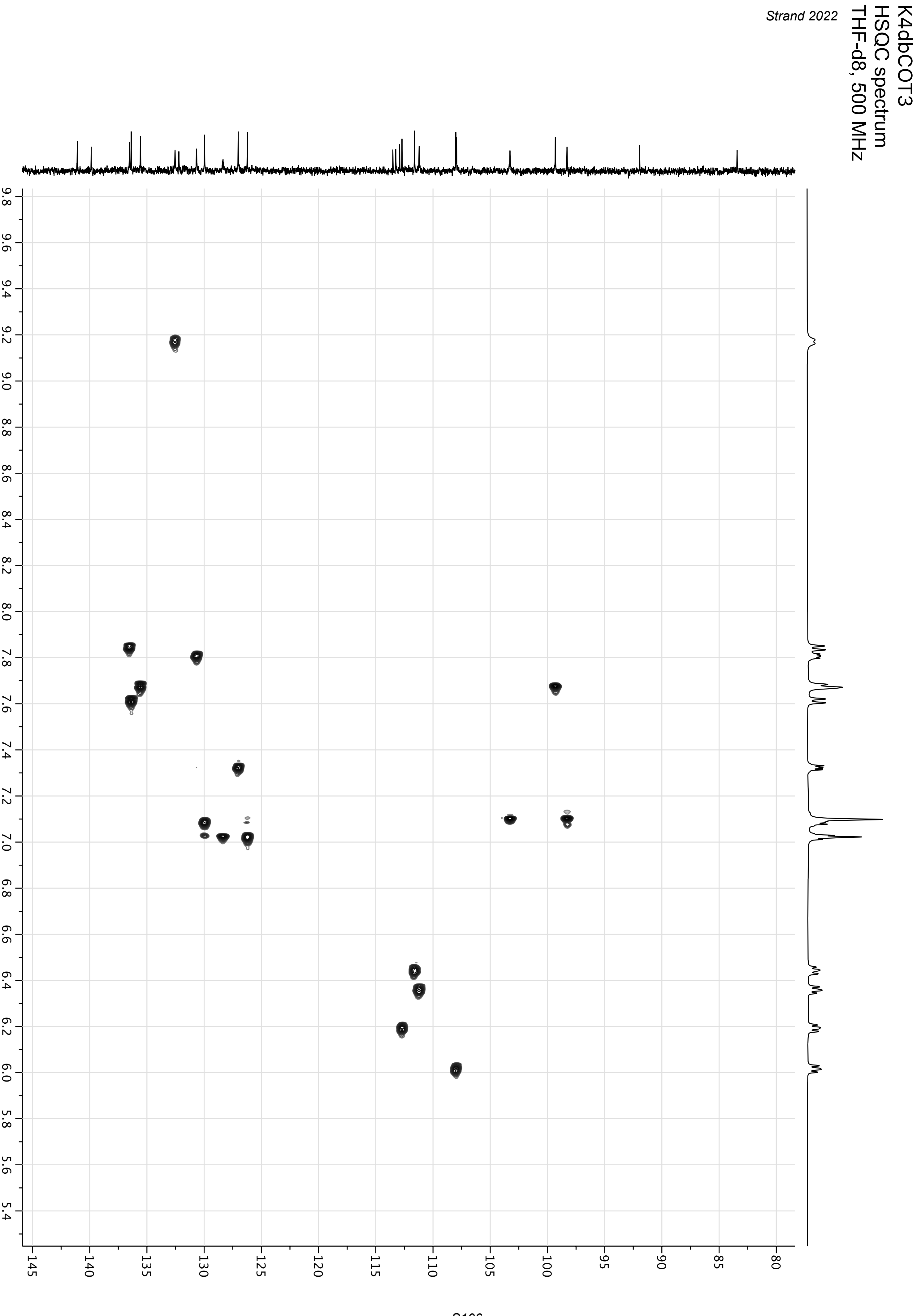
K4dbCOT3
HSQC spectrum
THF-d8, 500 MHz
Strand 2022
f1 (ppm)

K4dbCOT3
HMBC spectrum
THF-d8, 500 MHz

Strand 2022

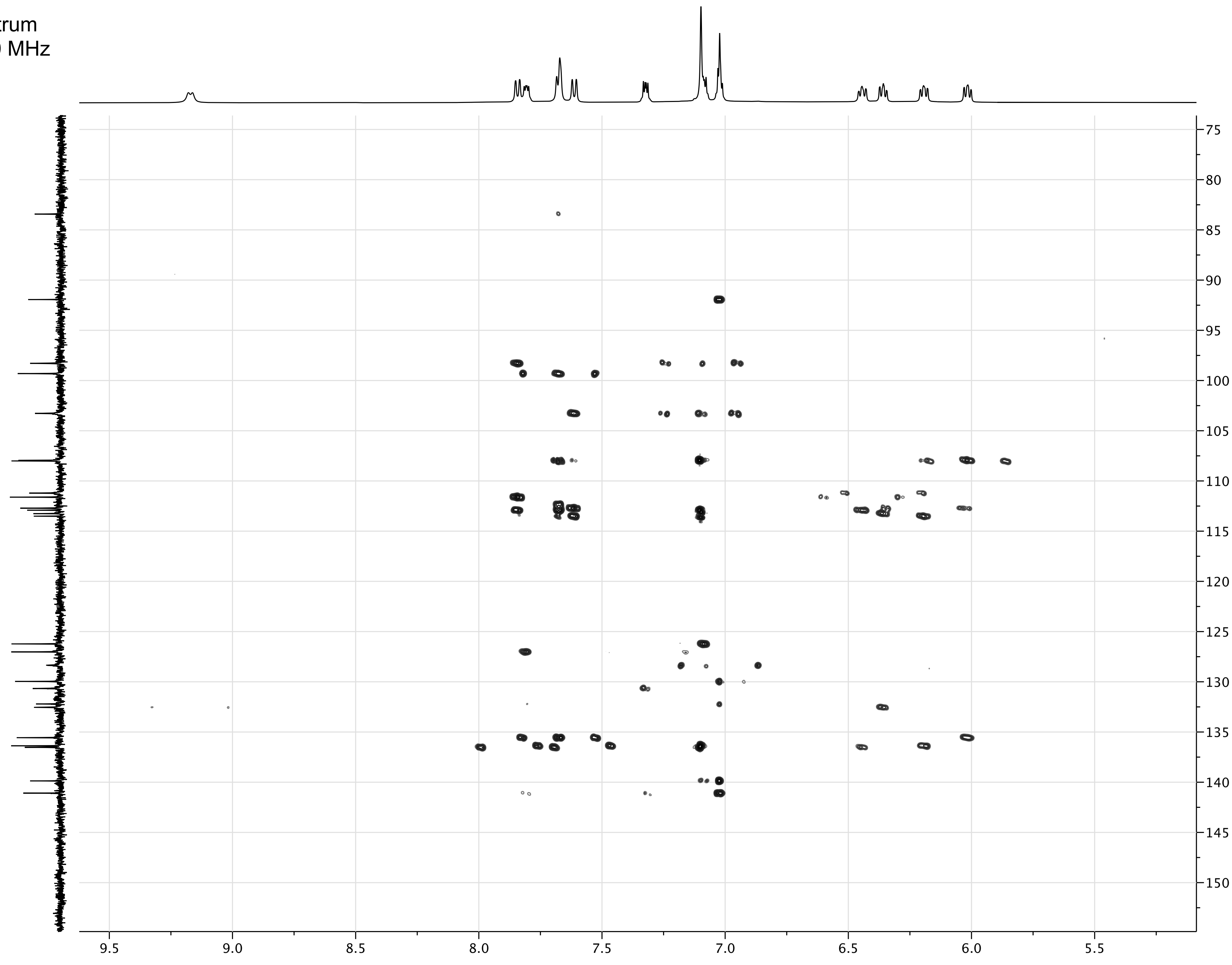

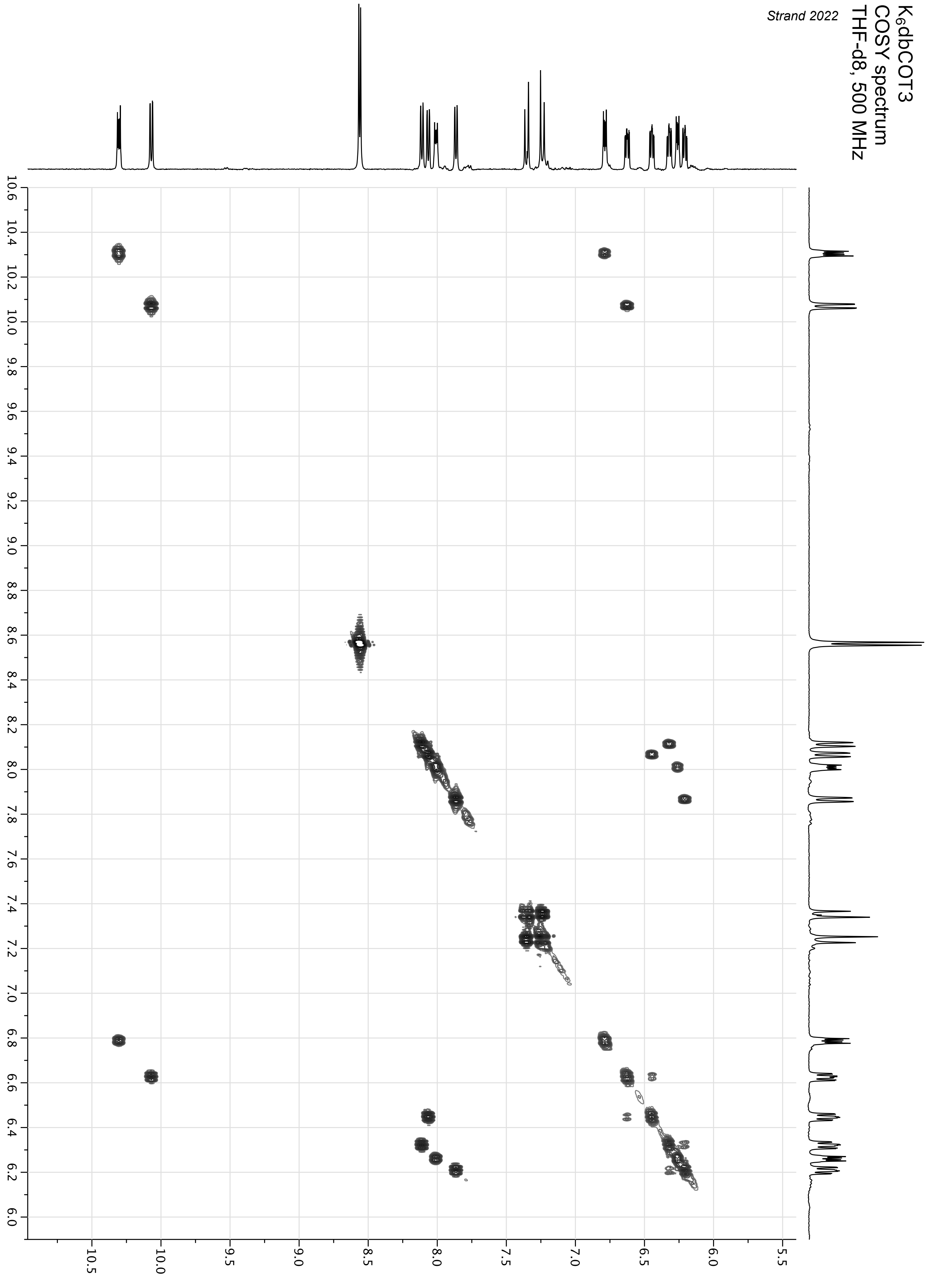
K6dbCOT3
COSY spectrum
THF-d8, 500 MHz
Strand 2022
f1 (ppm)

K$_6$dbCOT3
^{13}C-HSQC spectrum
THF-d8, 500 MHz, 268.2 K

Strand 2022

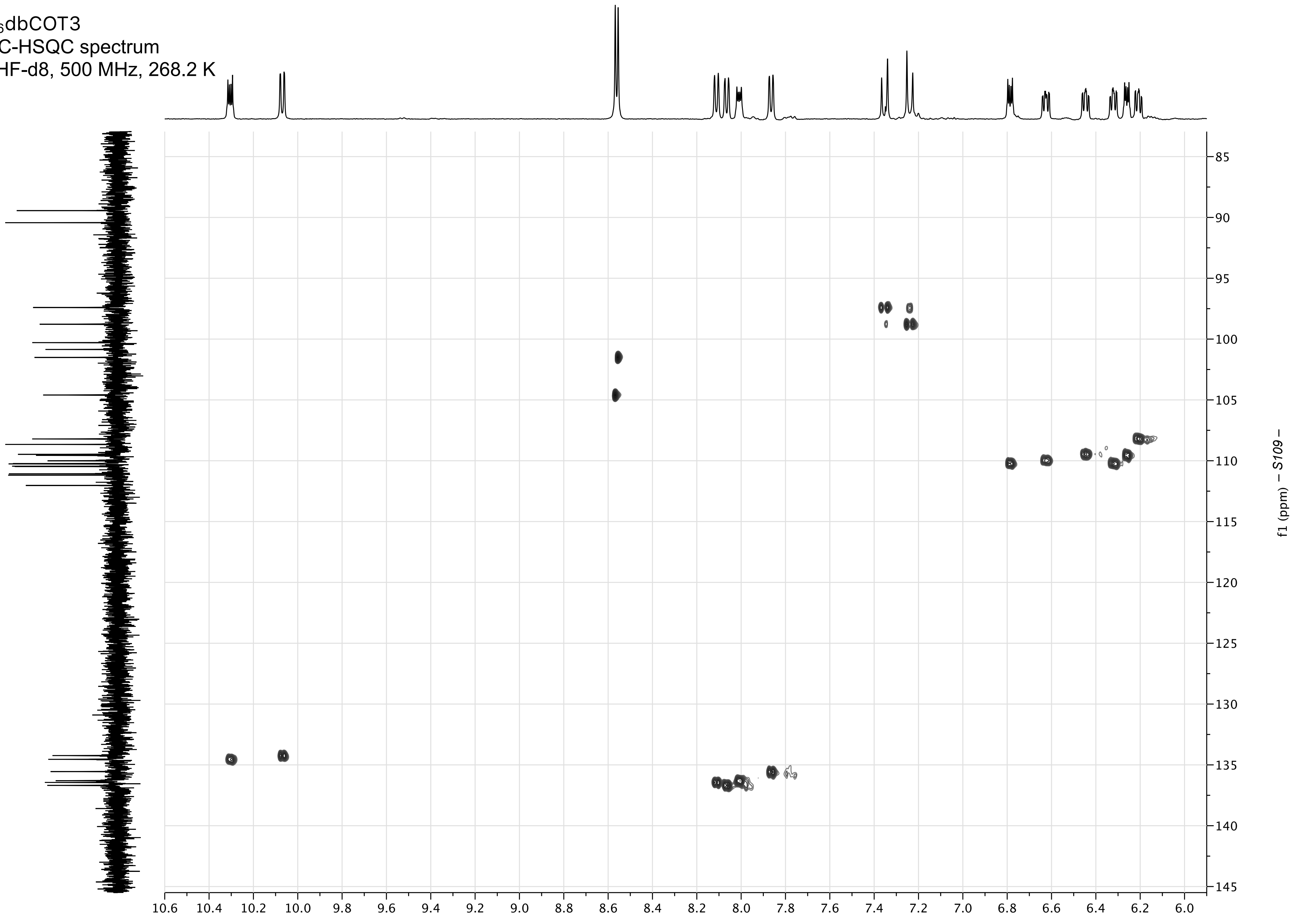

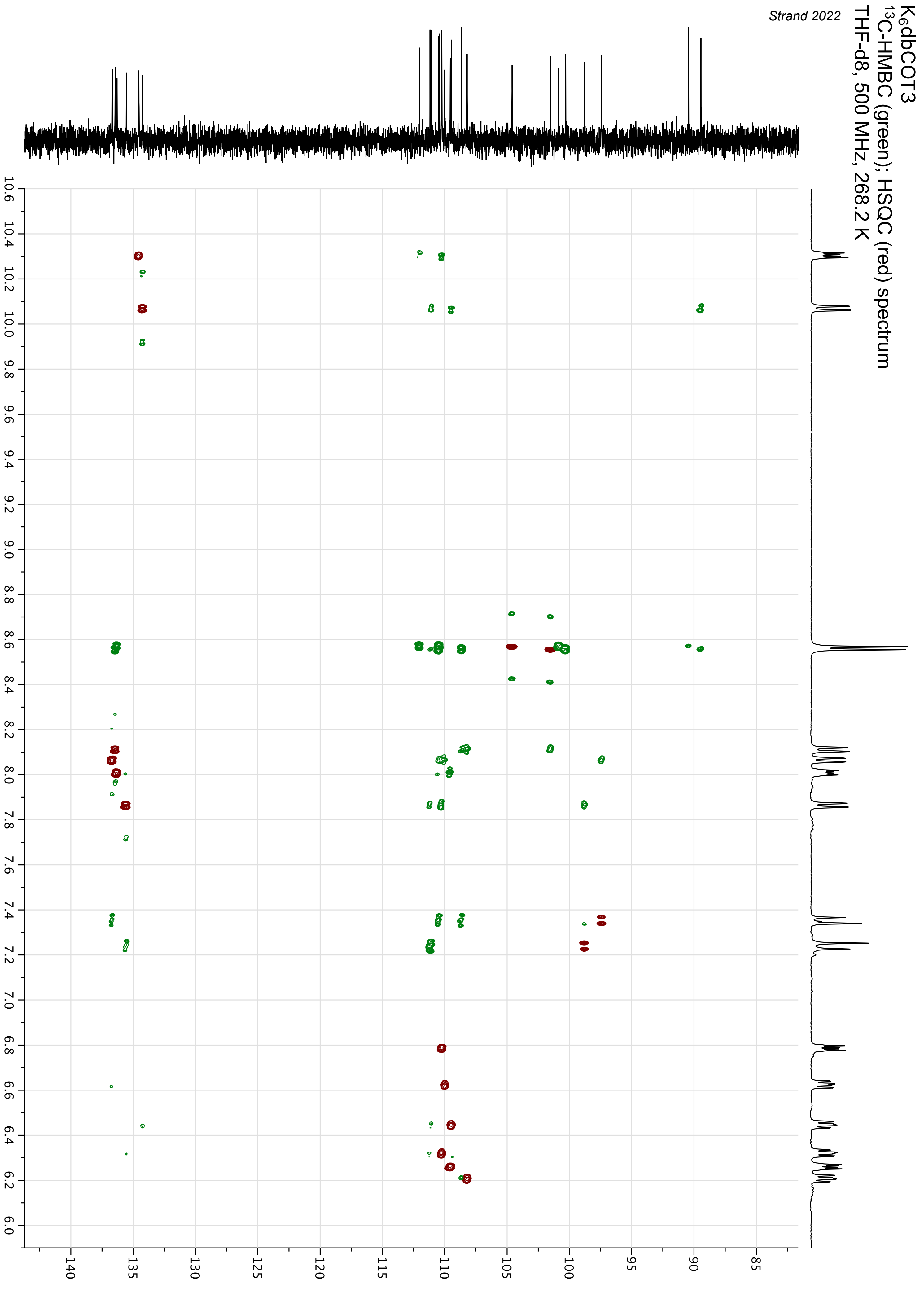
K6dbCOT3
13C-HMBC (green); HSQC (red) spectrum
THF-d8, 500 MHz, 268.2 K
Strand 2022
f1 (ppm)

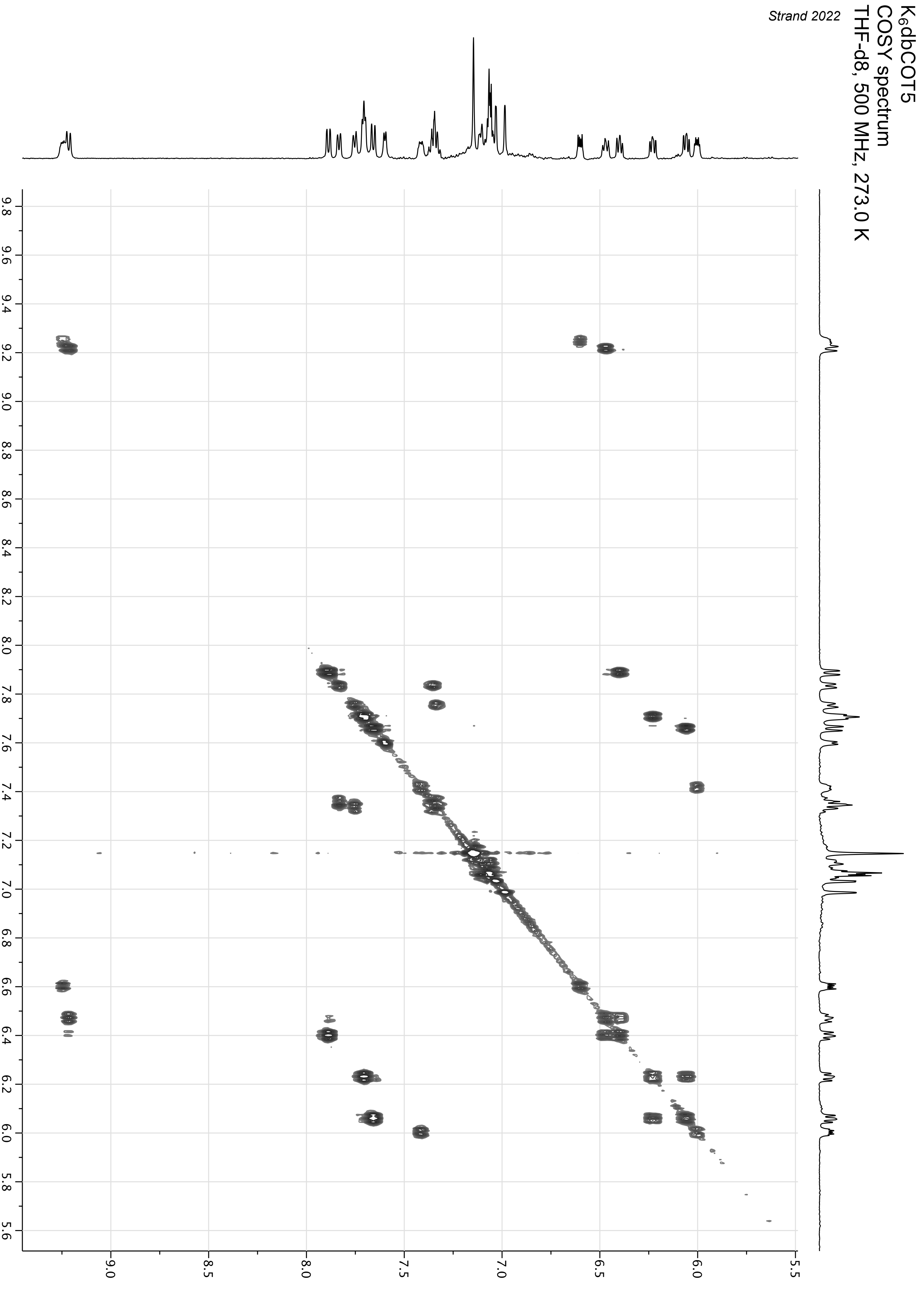

Strand 2022
K_6dbCOT5
COSY spectrum
THF-d8, 500 MHz, 273.0 K
f1 (ppm)

K$_6$dbCOT5
^{13}C-HSQC spectrum
THF-d8, 500 MHz, 273.0 K

Strand 2022

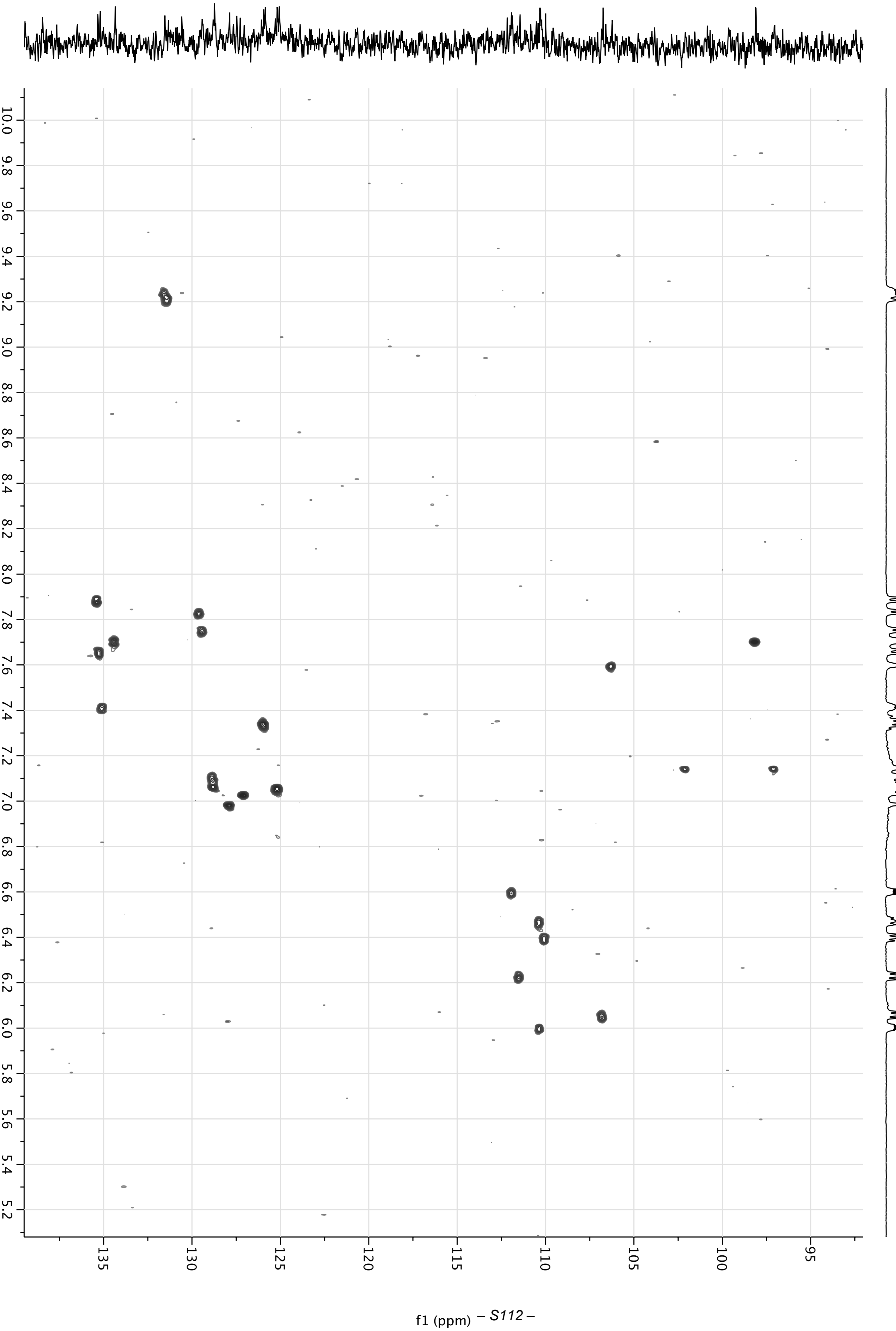

K_6dbCOT5
^{13}C-HMBC spectrum
THF-d8, 500 MHz, 273.0 K

Strand 2022

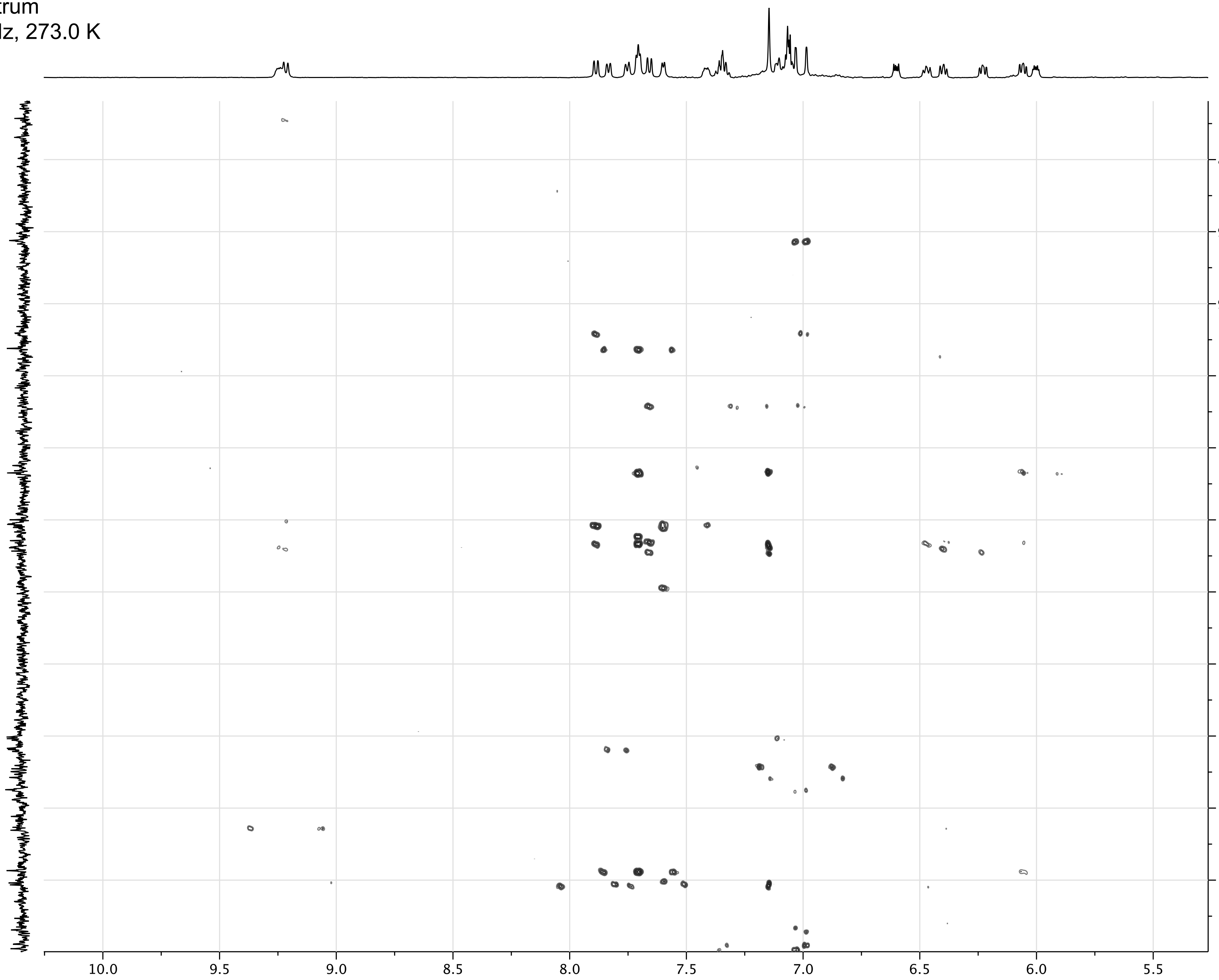

XIV. Copies of ^{1}H and ^{13}C NMR spectra

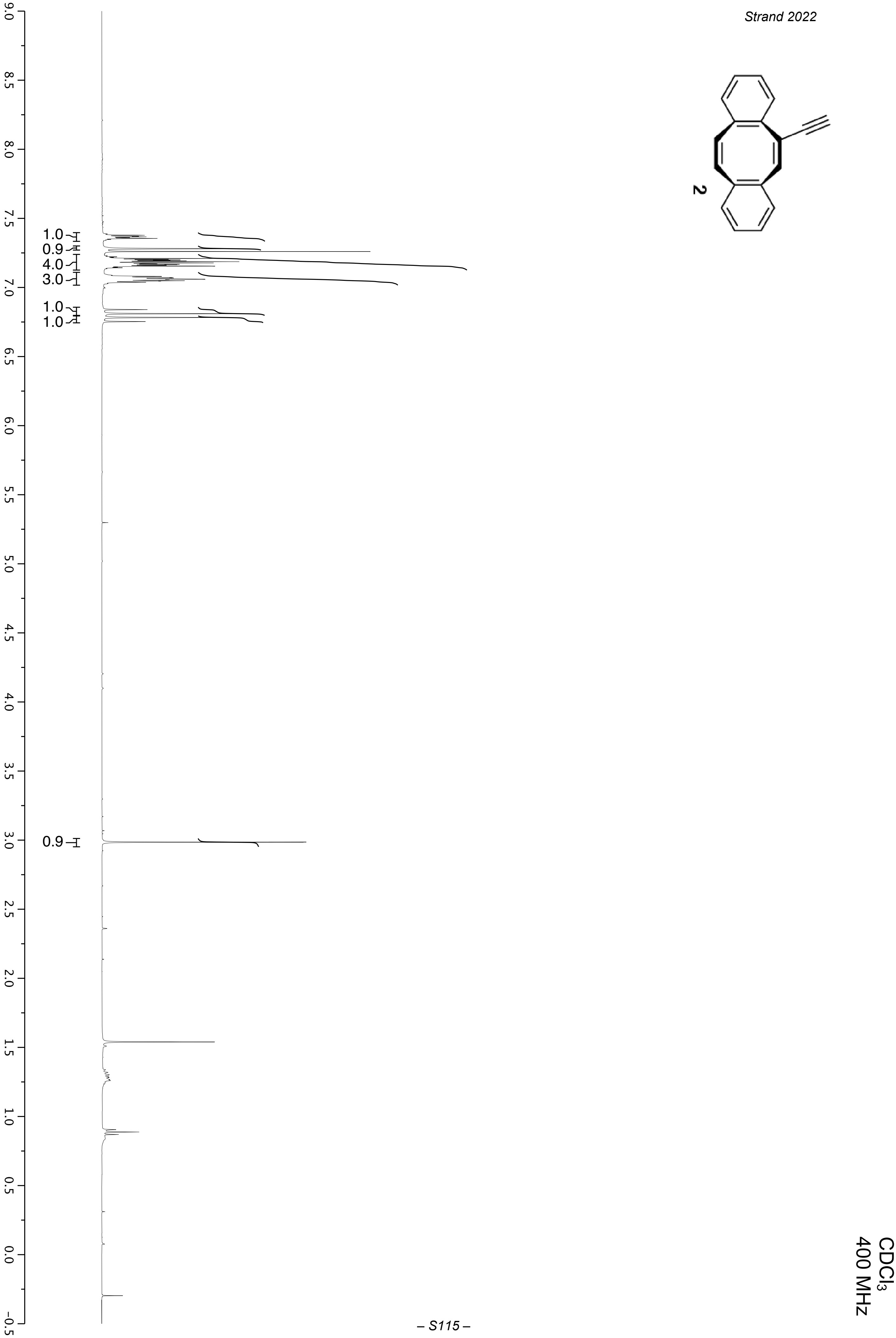
Strand 2022
2
CDCl3
400 MHz
1.0
0.9
4.0
3.0
1.0
1.0
0.9
9.0
8.5
8.0
7.5
7.0
6.5
6.0
5.5
5.0
4.5
4.0
3.5
3.0
2.5
2.0
1.5
1.0
0.5
0.0
–0.5

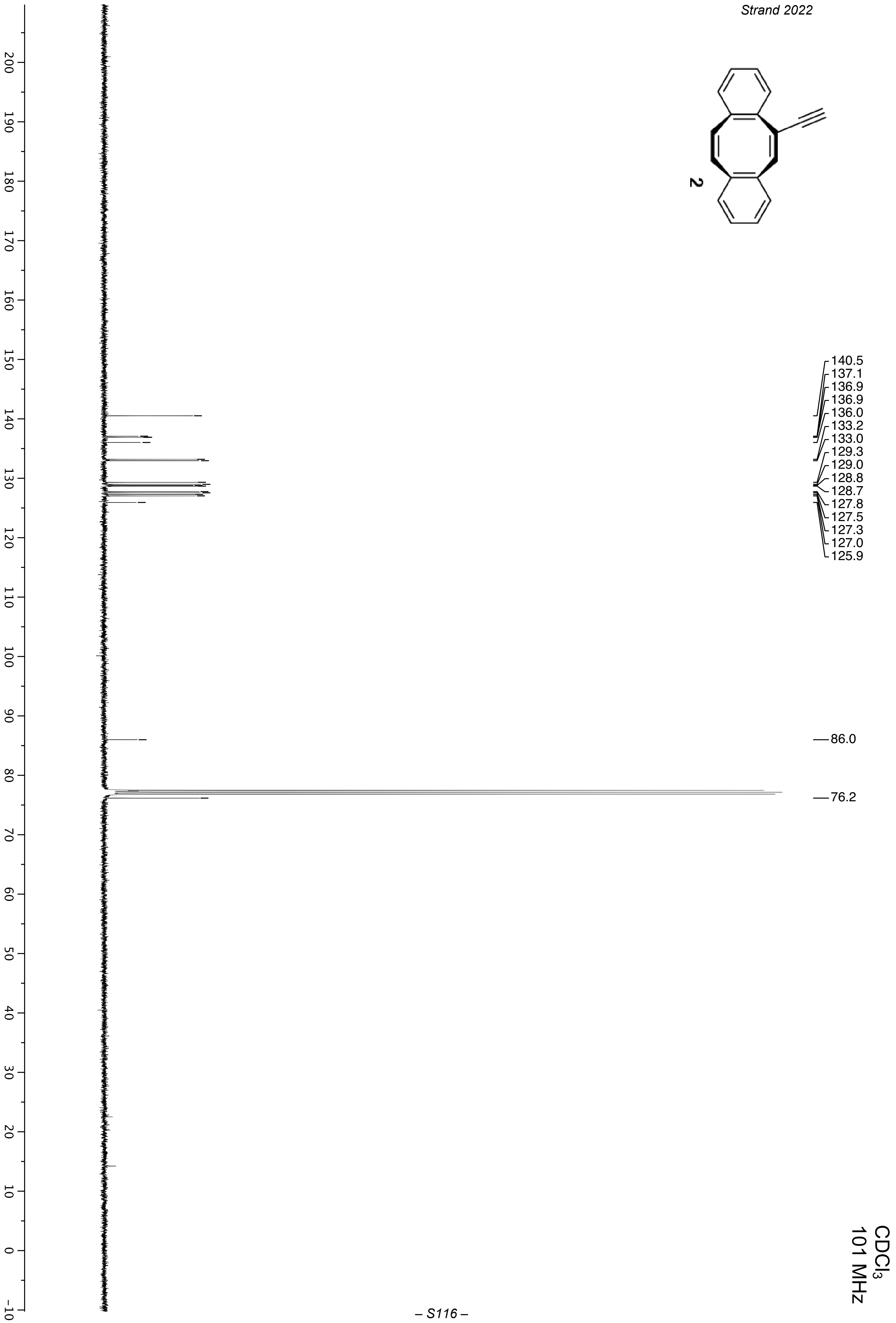
Strand 2022
2
140.5
137.1
136.9
136.9
136.0
133.2
133.0
129.3
129.0
128.8
128.7
127.8
127.5
127.3
127.0
125.9
86.0
76.2
$CDCl_3$
101 MHz

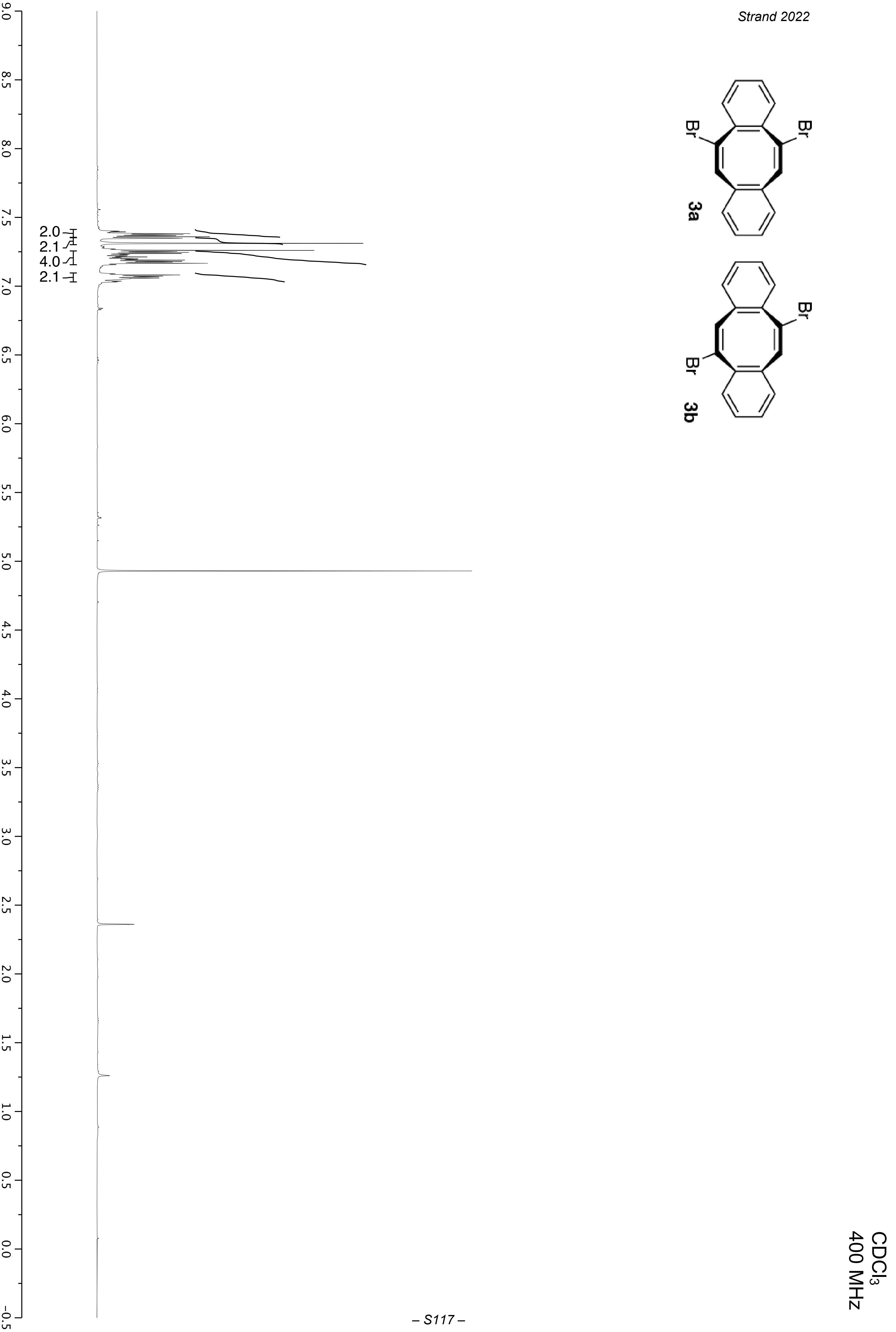
Br
Br
3a
Br
Br
3b
2.0
2.1
4.0
2.1
9.0
8.5
8.0
7.5
7.0
6.5
6.0
5.5
5.0
4.5
4.0
3.5
3.0
2.5
2.0
1.5
1.0
0.5
0.0
−0.5
CDCl3
400 MHz

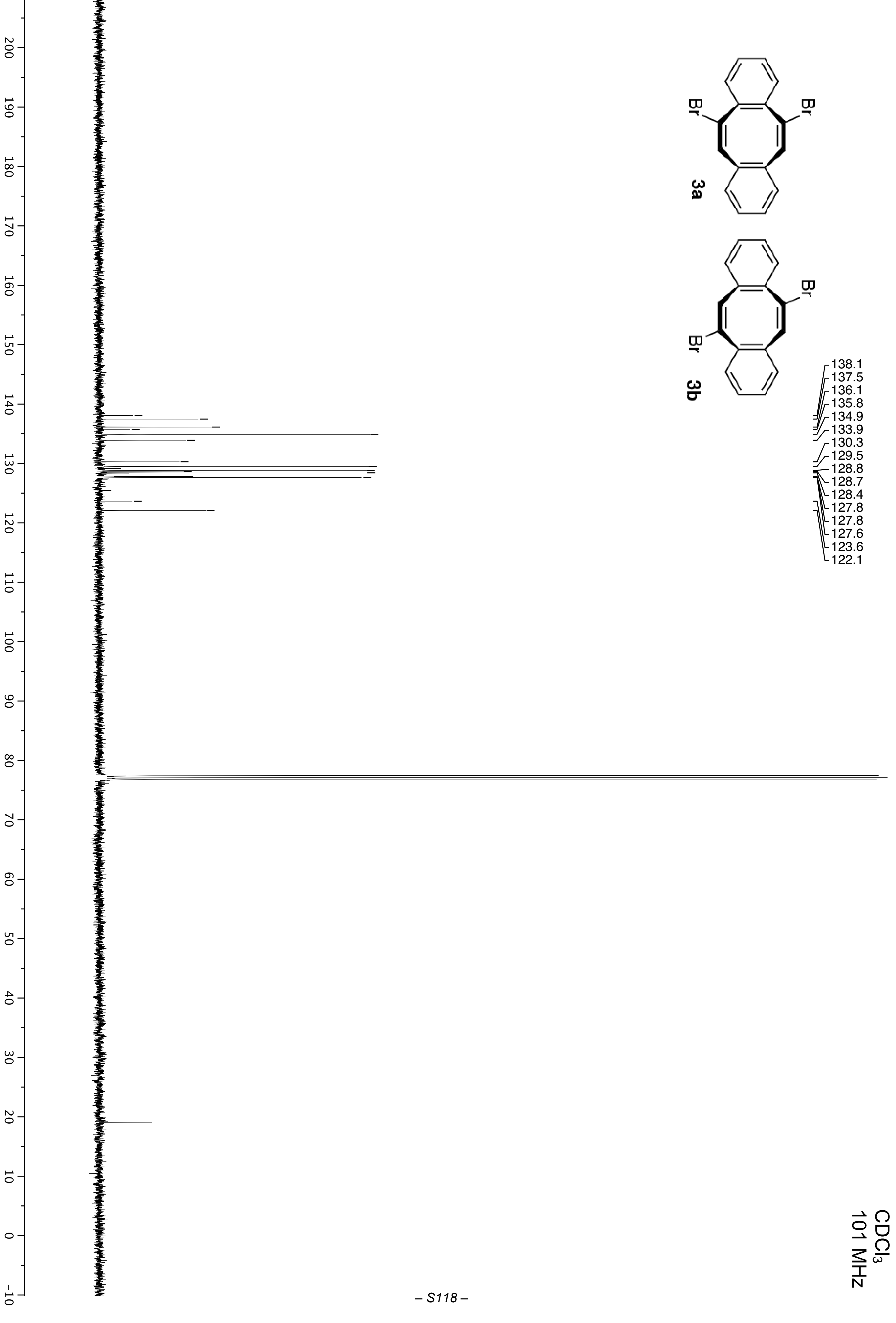

3a
3b
Br
138.1
137.5
136.1
135.8
134.9
133.9
130.3
129.5
128.8
128.7
128.4
127.8
127.8
127.6
123.6
122.1
CDCl3
101 MHz
200
190
180
170
160
150
140
130
120
110
100
90
80
70
60
50
40
30
20
10
0
−10

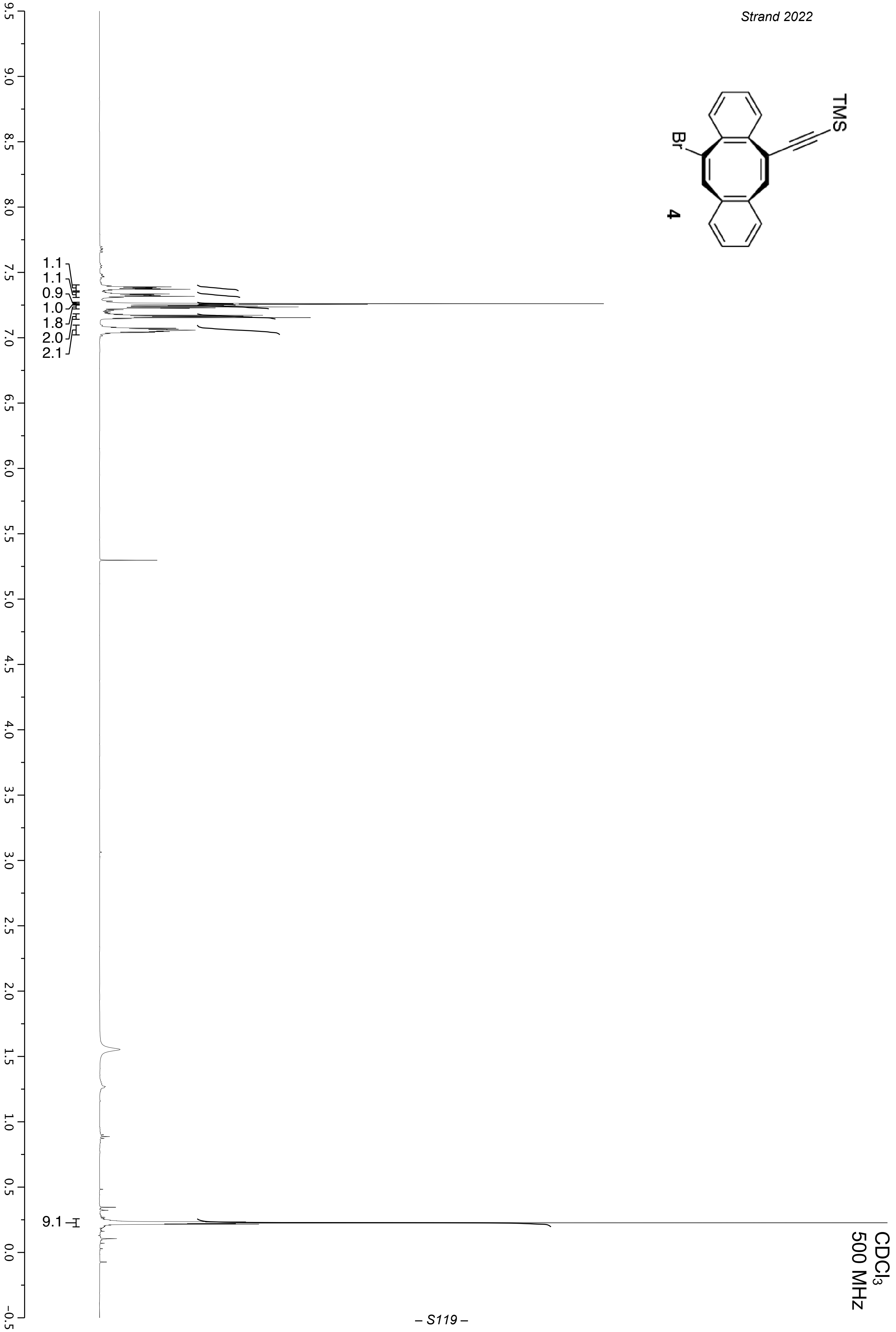
Strand 2022
TMS
Br
4
CDCl$_3$
500 MHz
1.1
1.1
0.9
1.0
1.8
2.0
2.1
9.1
9.5
9.0
8.5
8.0
7.5
7.0
6.5
6.0
5.5
5.0
4.5
4.0
3.5
3.0
2.5
2.0
1.5
1.0
0.5
0.0
−0.5

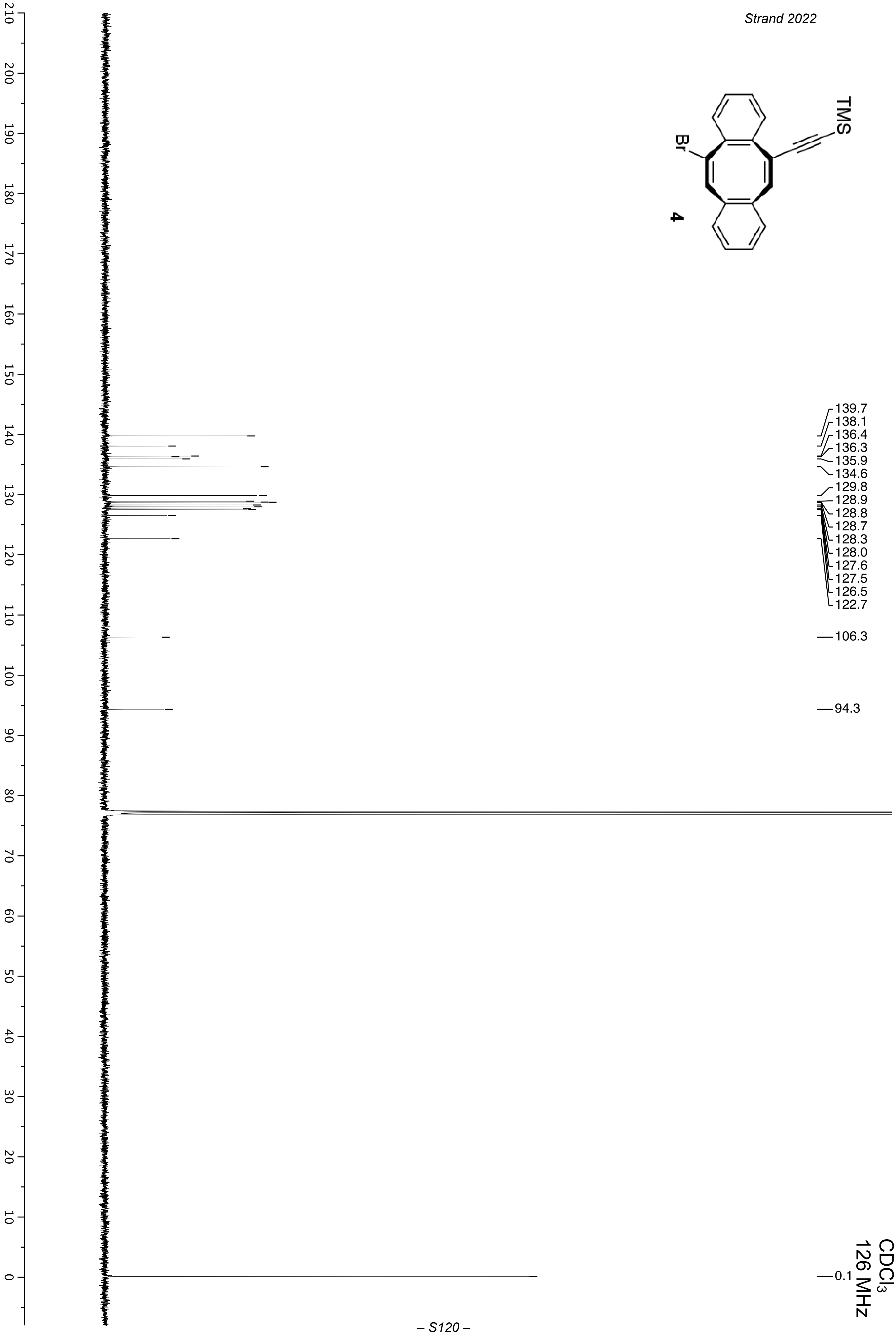
TMS
Br
4
139.7
138.1
136.4
136.3
135.9
134.6
129.8
128.9
128.8
128.7
128.3
128.0
127.6
127.5
126.5
122.7
106.3
94.3
0.1
$CDCl_3$
126 MHz
210
200
190
180
170
160
150
140
130
120
110
100
90
80
70
60
50
40
30
20
10
0

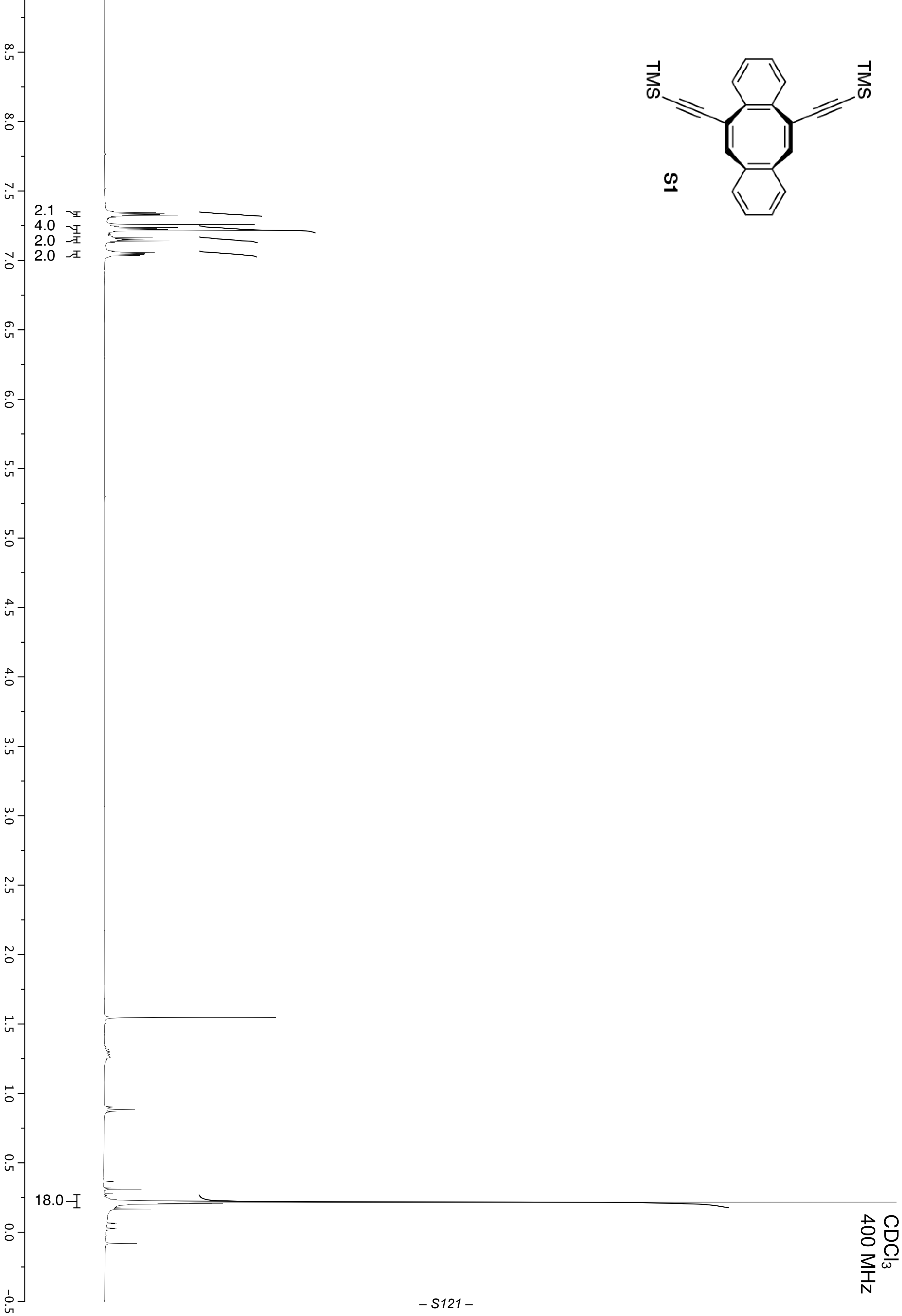
TMS
TMS
S1
2.1
4.0
2.0
2.0
18.0
CDCl3
400 MHz
9.0
8.5
8.0
7.5
7.0
6.5
6.0
5.5
5.0
4.5
4.0
3.5
3.0
2.5
2.0
1.5
1.0
0.5
0.0
−0.5

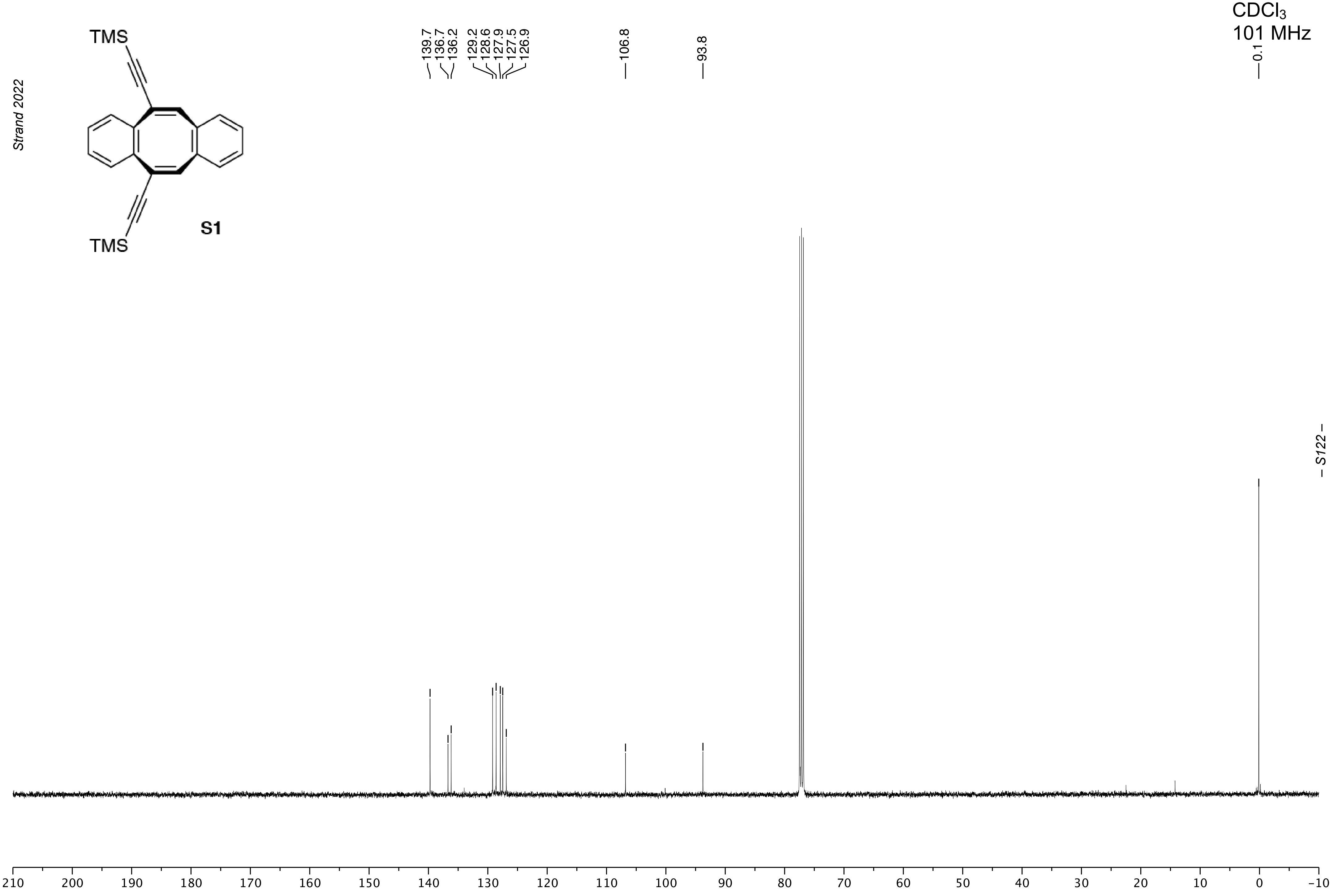
TMS
TMS
S1
CDCl$_3$
101 MHz
139.7
136.7
136.2
129.2
128.6
127.9
127.5
126.9
106.8
93.8
0.1
210
200
190
180
170
160
150
140
130
120
110
100
90
80
70
60
50
40
30
20
10
0
−10

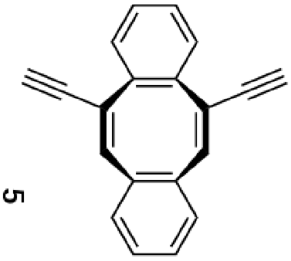

5

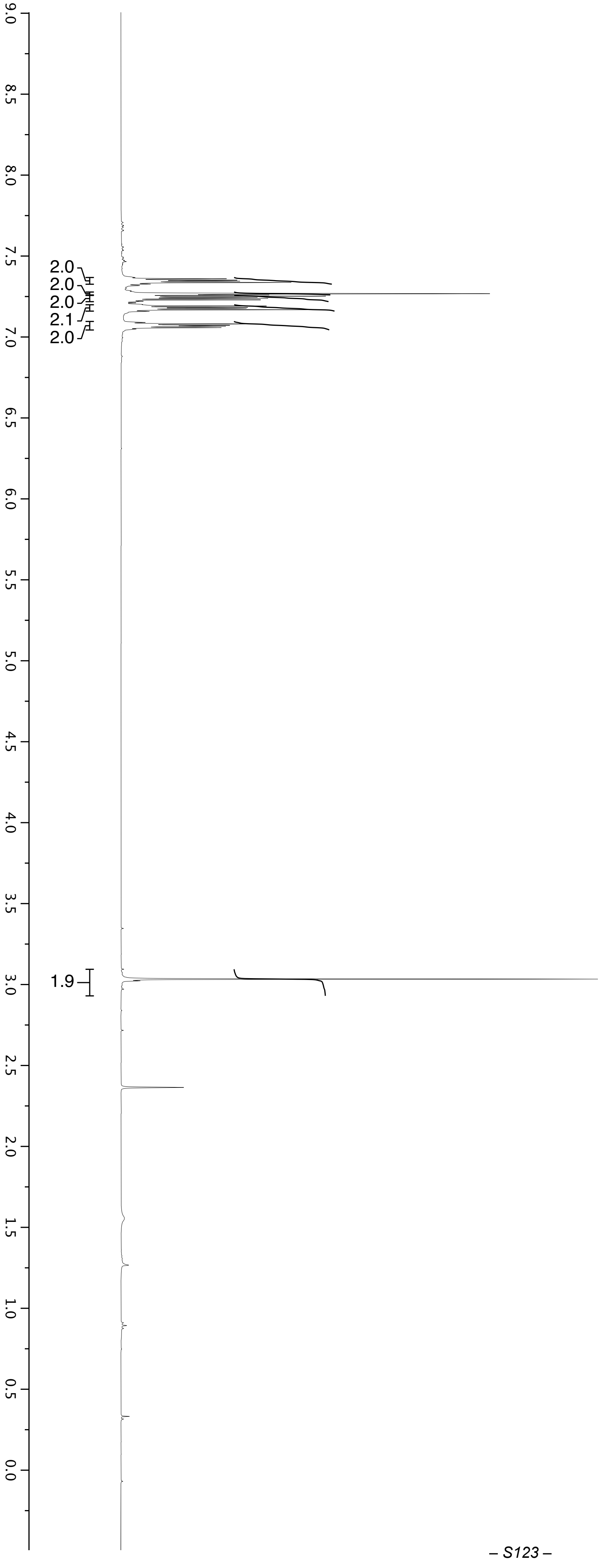

$CDCl_3$
400 MHz

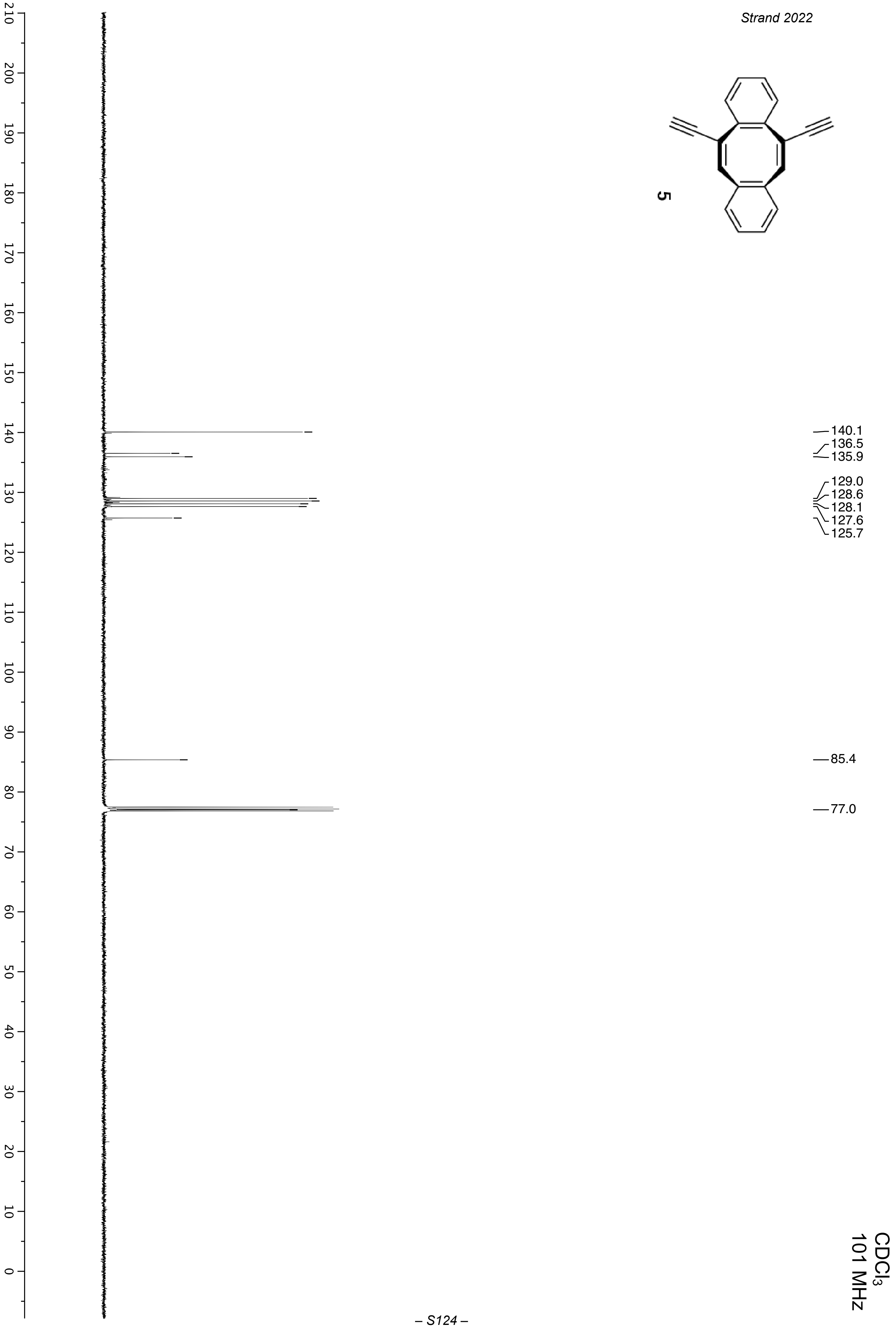
Strand 2022
5
140.1
136.5
135.9
129.0
128.6
128.1
127.6
125.7
85.4
77.0
CDCl$_3$
101 MHz
210
200
190
180
170
160
150
140
130
120
110
100
90
80
70
60
50
40
30
20
10
0

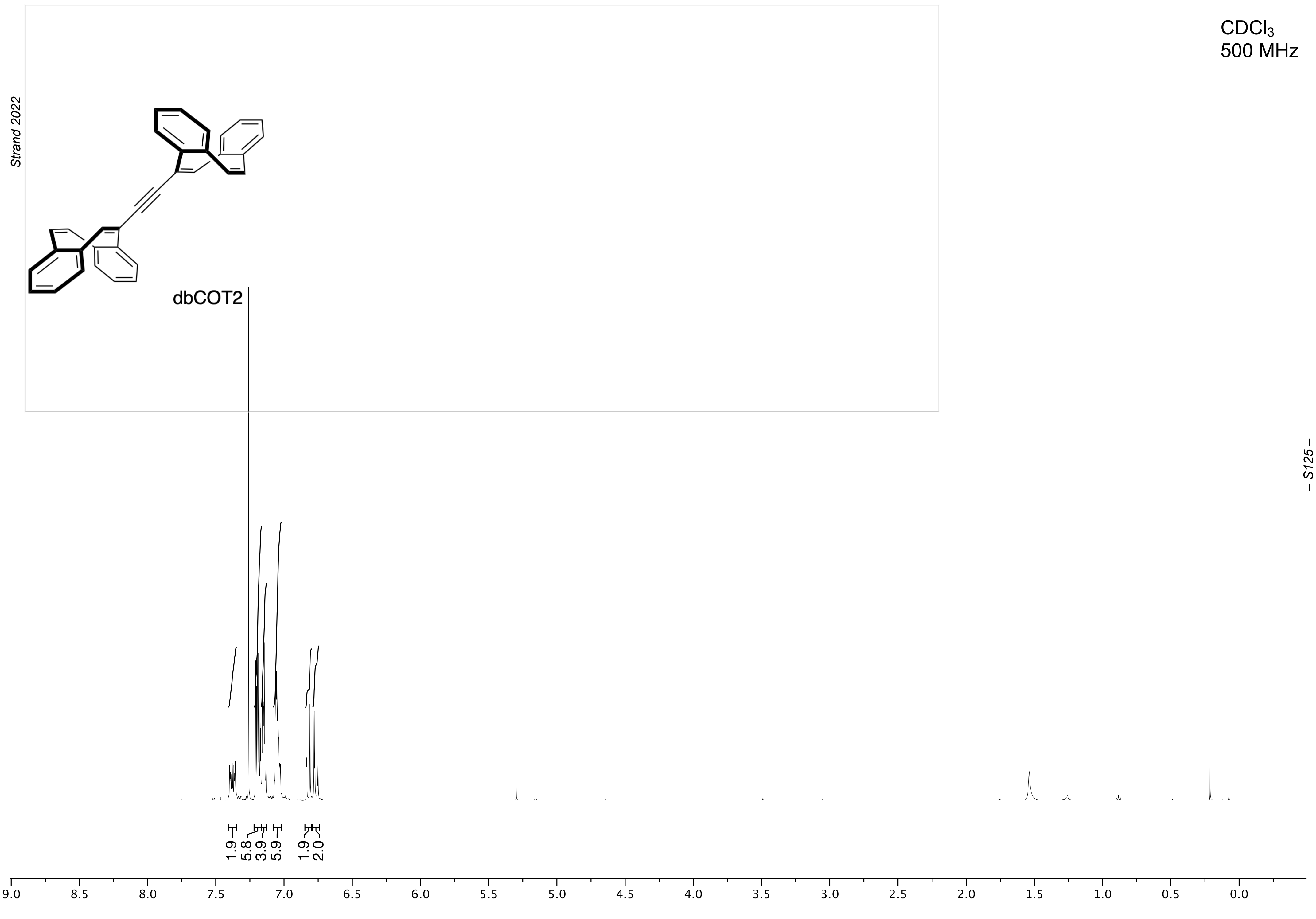
Strand 2022
dbCOT2
CDCl$_3$
500 MHz
1.9
5.8
3.9
5.9
1.9
2.0
9.0
8.5
8.0
7.5
7.0
6.5
6.0
5.5
5.0
4.5
4.0
3.5
3.0
2.5
2.0
1.5
1.0
0.5
0.0

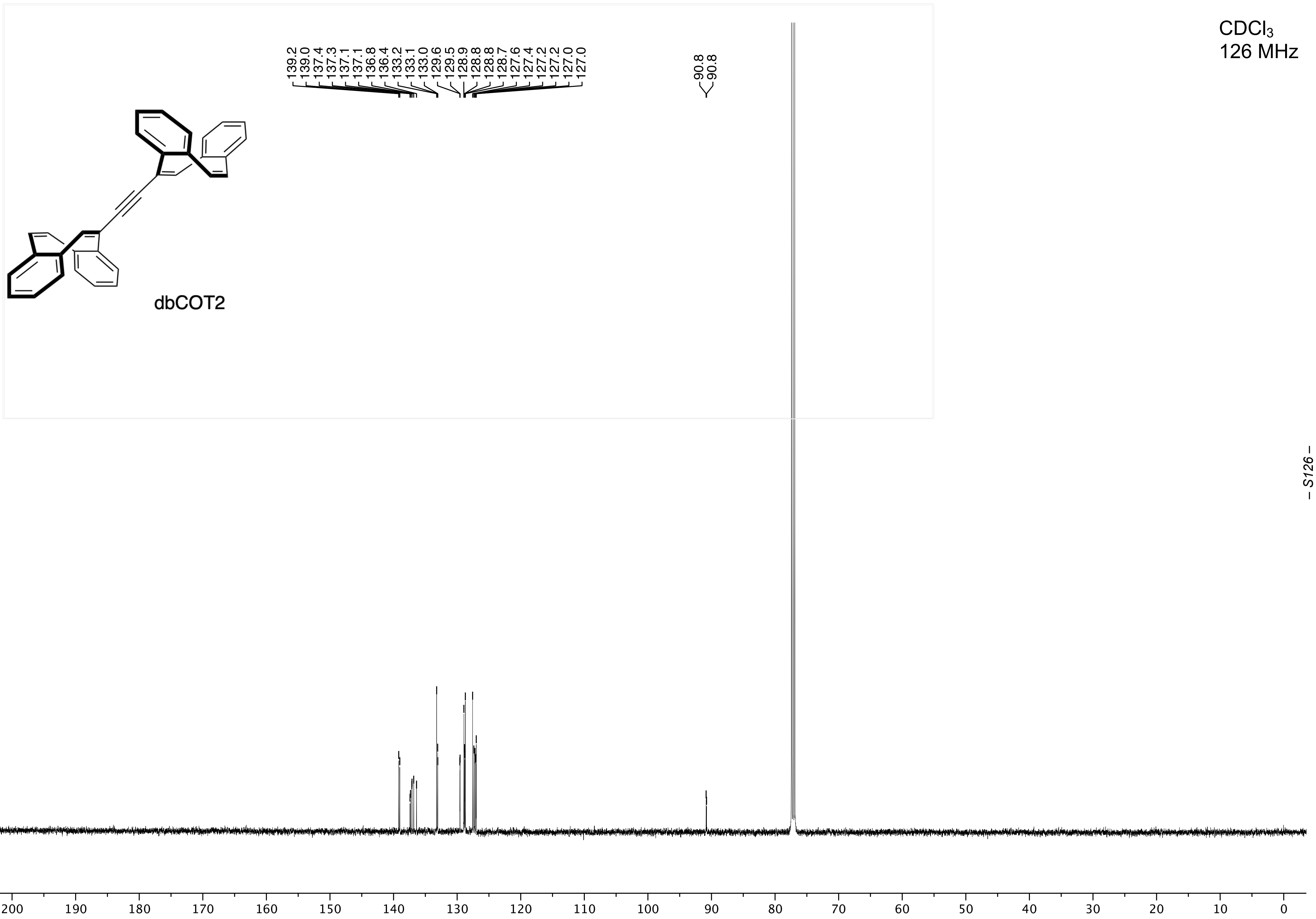
dbCOT2
139.2
139.0
137.4
137.3
137.1
137.1
136.8
136.4
133.2
133.1
133.0
129.6
129.5
128.9
128.8
128.8
128.7
127.6
127.4
127.2
127.2
127.0
127.0
90.8
90.8
CDCl3
126 MHz
200
190
180
170
160
150
140
130
120
110
100
90
80
70
60
50
40
30
20
10
0

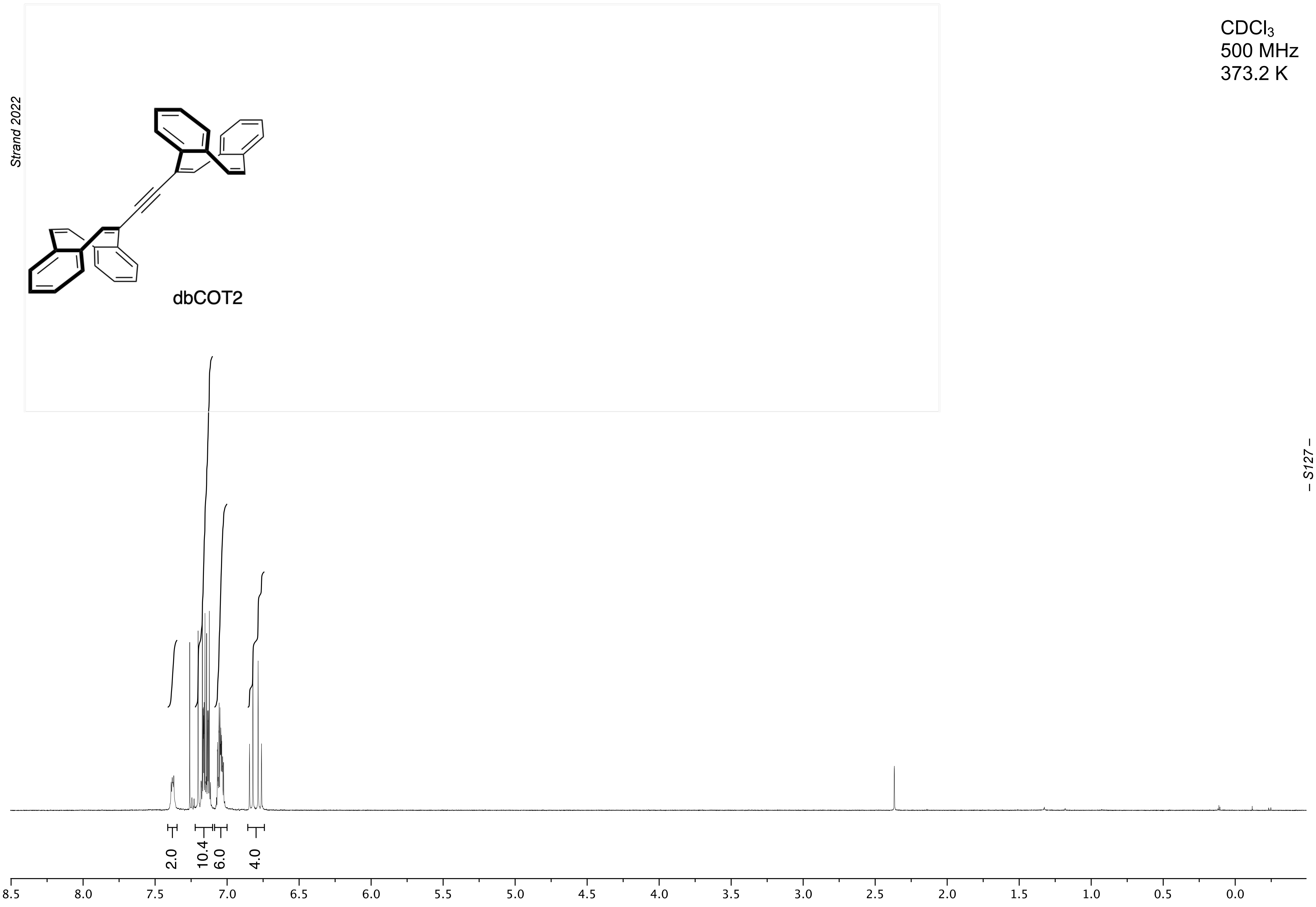
Strand 2022
dbCOT2
CDCl3
500 MHz
373.2 K
2.0
10.4
6.0
4.0
8.5
8.0
7.5
7.0
6.5
6.0
5.5
5.0
4.5
4.0
3.5
3.0
2.5
2.0
1.5
1.0
0.5
0.0

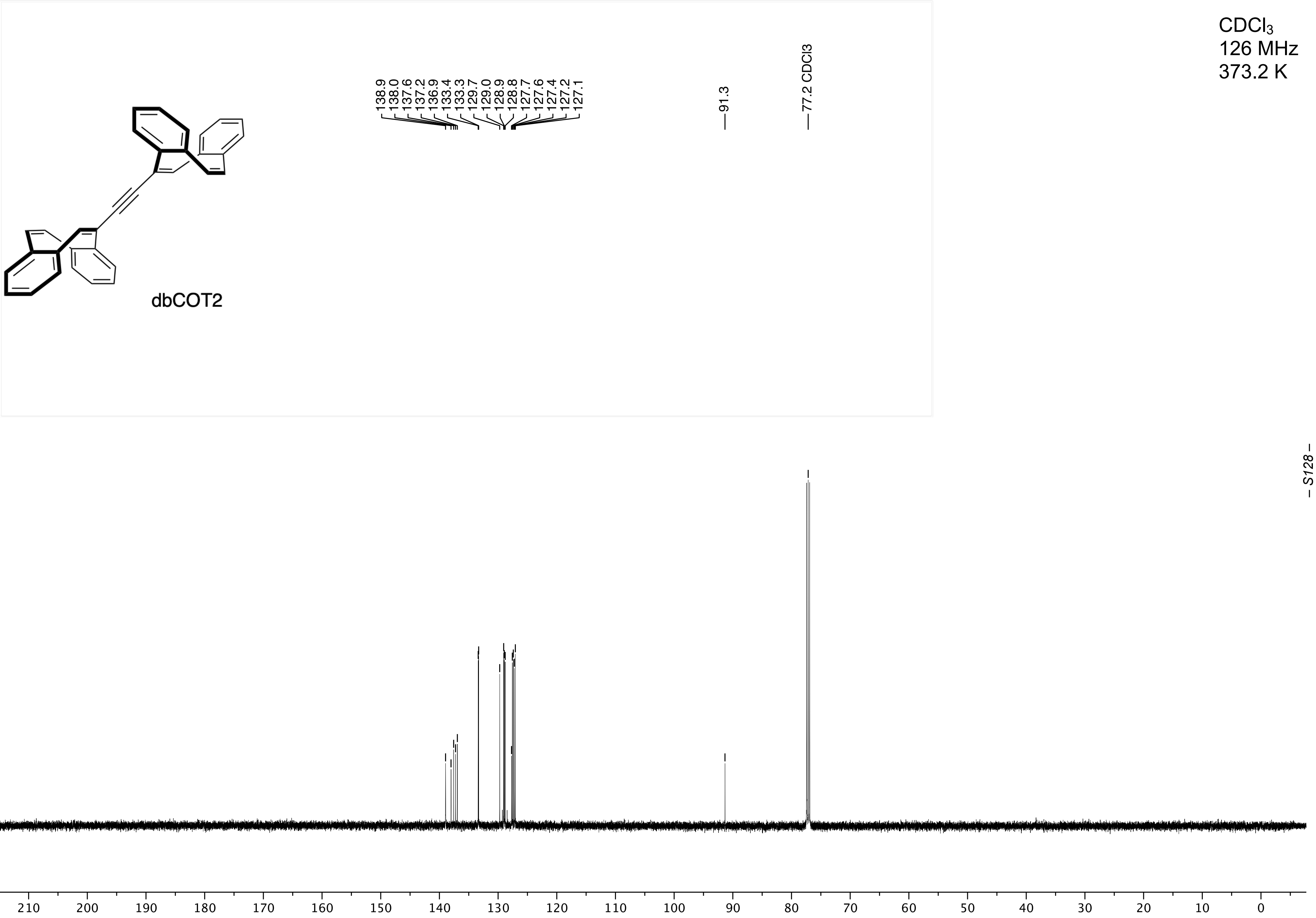
dbCOT2
138.9
138.0
137.6
137.2
136.9
133.4
133.3
129.7
129.0
128.9
128.8
127.7
127.6
127.4
127.2
127.1
91.3
77.2 CDCl3
CDCl3
126 MHz
373.2 K
210
200
190
180
170
160
150
140
130
120
110
100
90
80
70
60
50
40
30
20
10
0

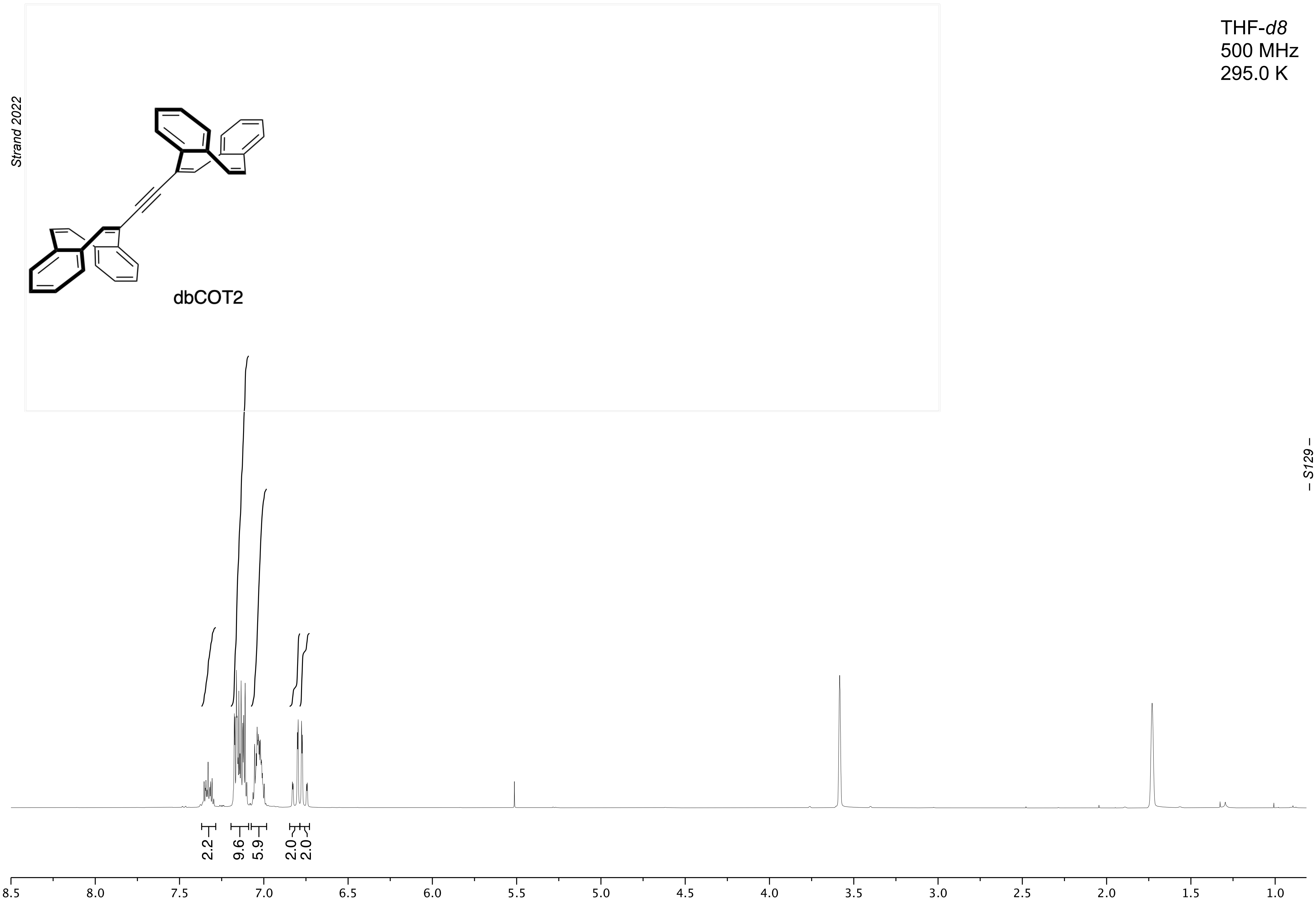
Strand 2022
dbCOT2
THF-d8
500 MHz
295.0 K
2.2
9.6
5.9
2.0
2.0
8.5
8.0
7.5
7.0
6.5
6.0
5.5
5.0
4.5
4.0
3.5
3.0
2.5
2.0
1.5
1.0

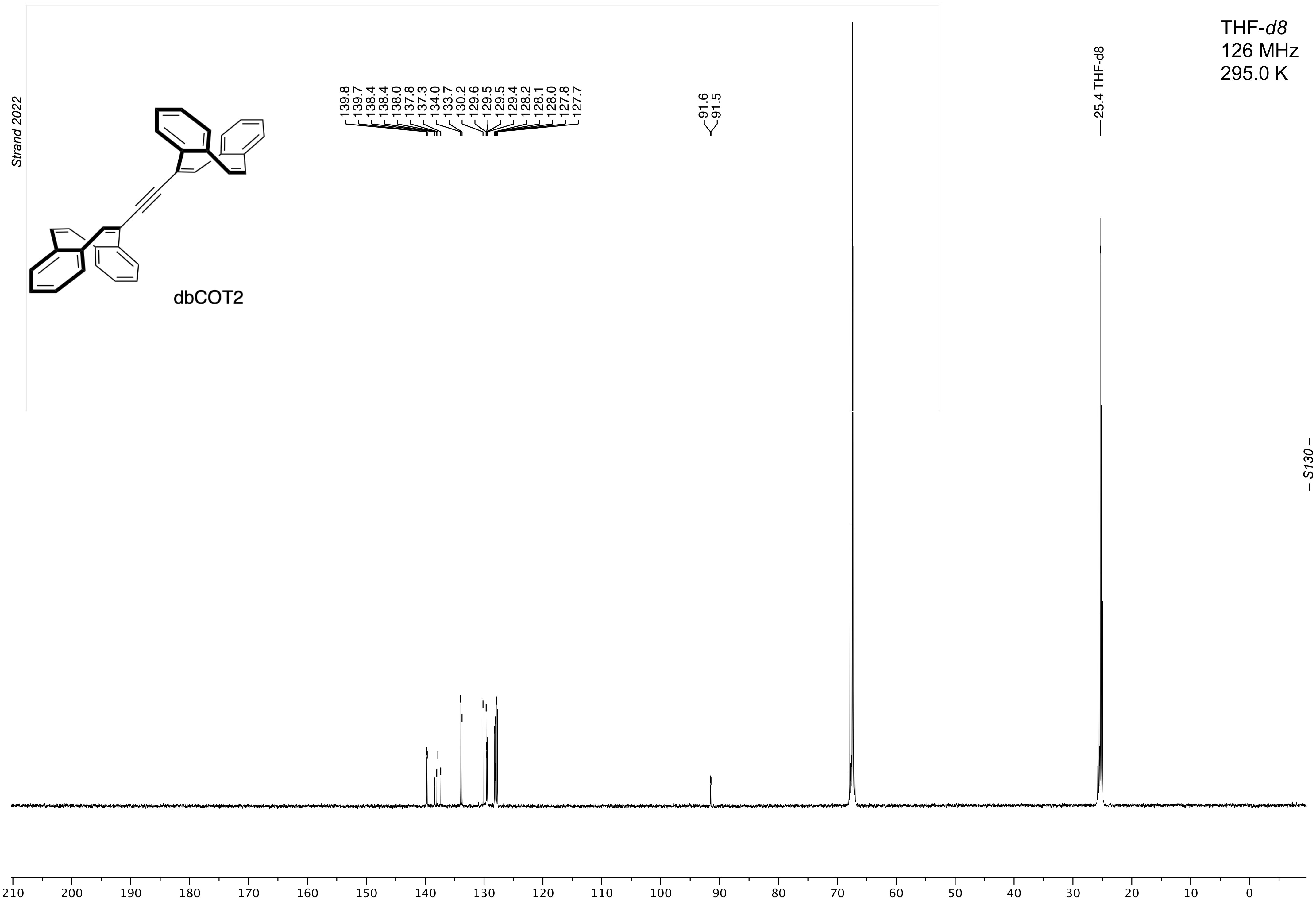
Strand 2022
dbCOT2
139.8
139.7
138.4
138.4
138.0
137.8
137.3
134.0
133.7
130.2
129.6
129.5
129.5
129.4
128.2
128.1
128.0
127.8
127.7
91.6
91.5
25.4 THF-d8
THF-d8
126 MHz
295.0 K
210 200 190 180 170 160 150 140 130 120 110 100 90 80 70 60 50 40 30 20 10 0

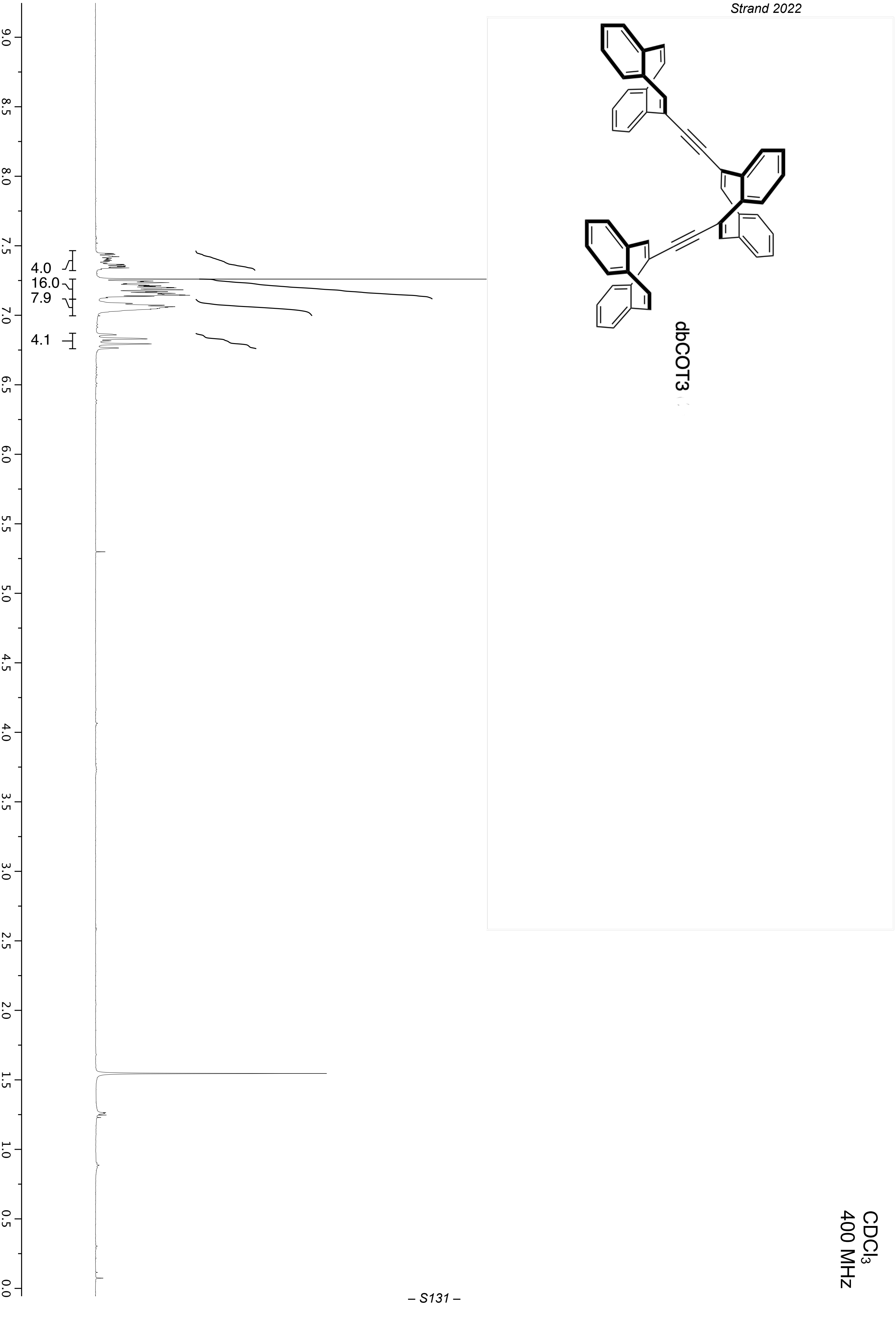
dbCOT3
CDCl3
400 MHz
4.0
16.0
7.9
4.1
9.0
8.5
8.0
7.5
7.0
6.5
6.0
5.5
5.0
4.5
4.0
3.5
3.0
2.5
2.0
1.5
1.0
0.5
0.0

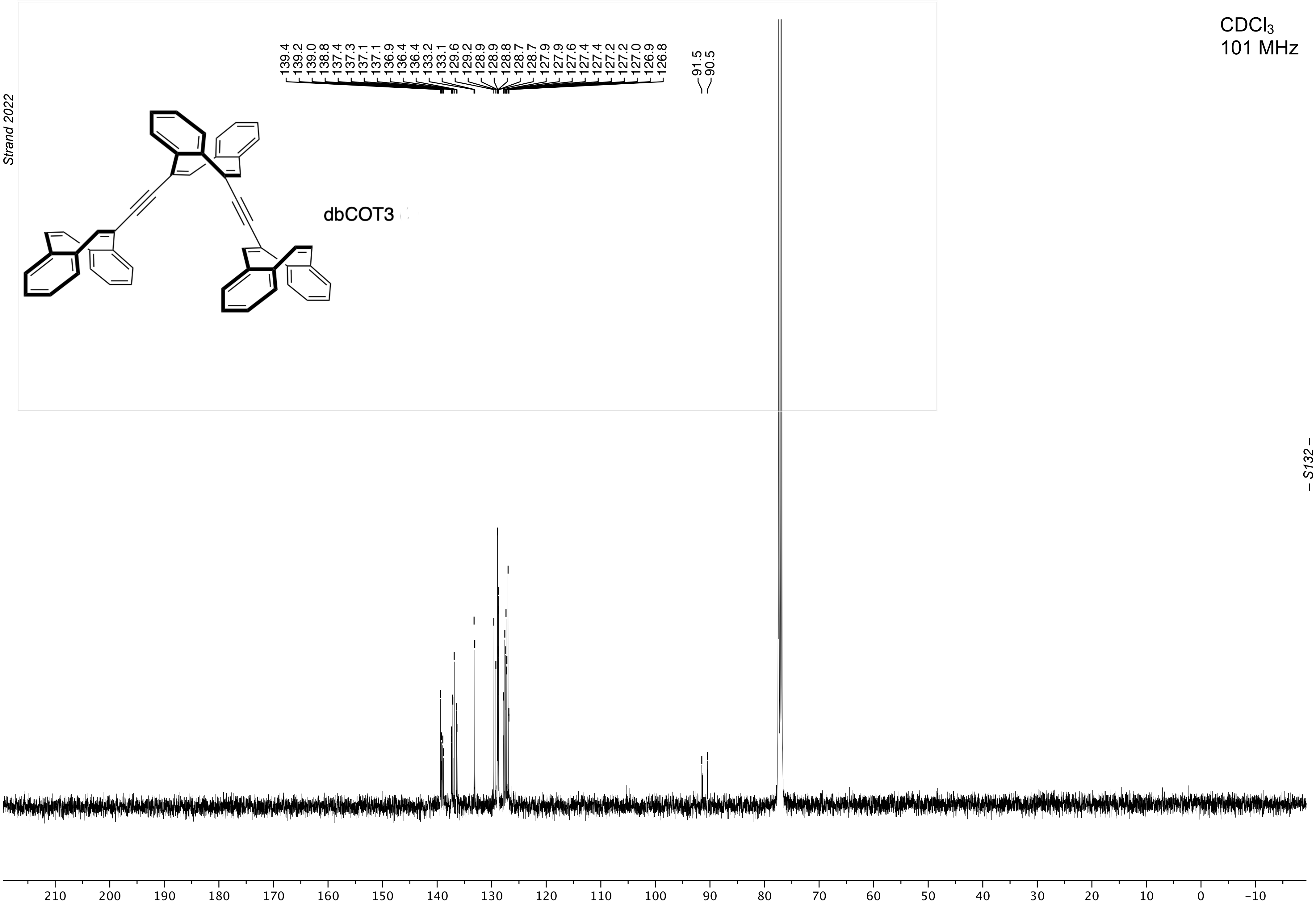
dbCOT3
139.4
139.2
139.0
138.8
137.4
137.3
137.1
137.1
136.9
136.4
136.4
133.2
133.1
129.6
129.2
128.9
128.9
128.8
128.7
128.7
127.9
127.9
127.6
127.4
127.4
127.2
127.2
127.0
126.9
126.8
91.5
90.5
$CDCl_3$
101 MHz
210 200 190 180 170 160 150 140 130 120 110 100 90 80 70 60 50 40 30 20 10 0 −10

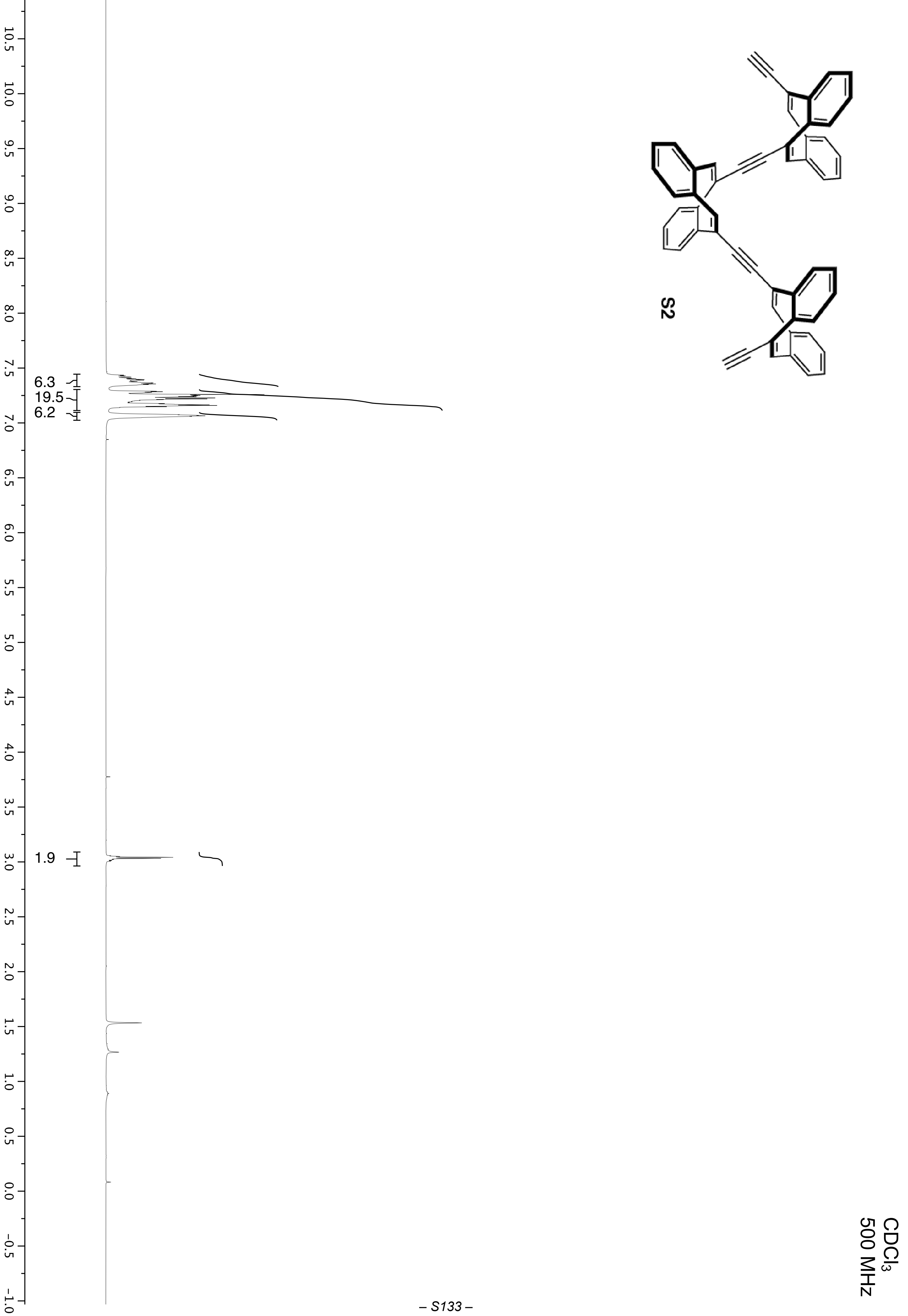
S2
CDCl3
500 MHz
6.3
19.5
6.2
1.9

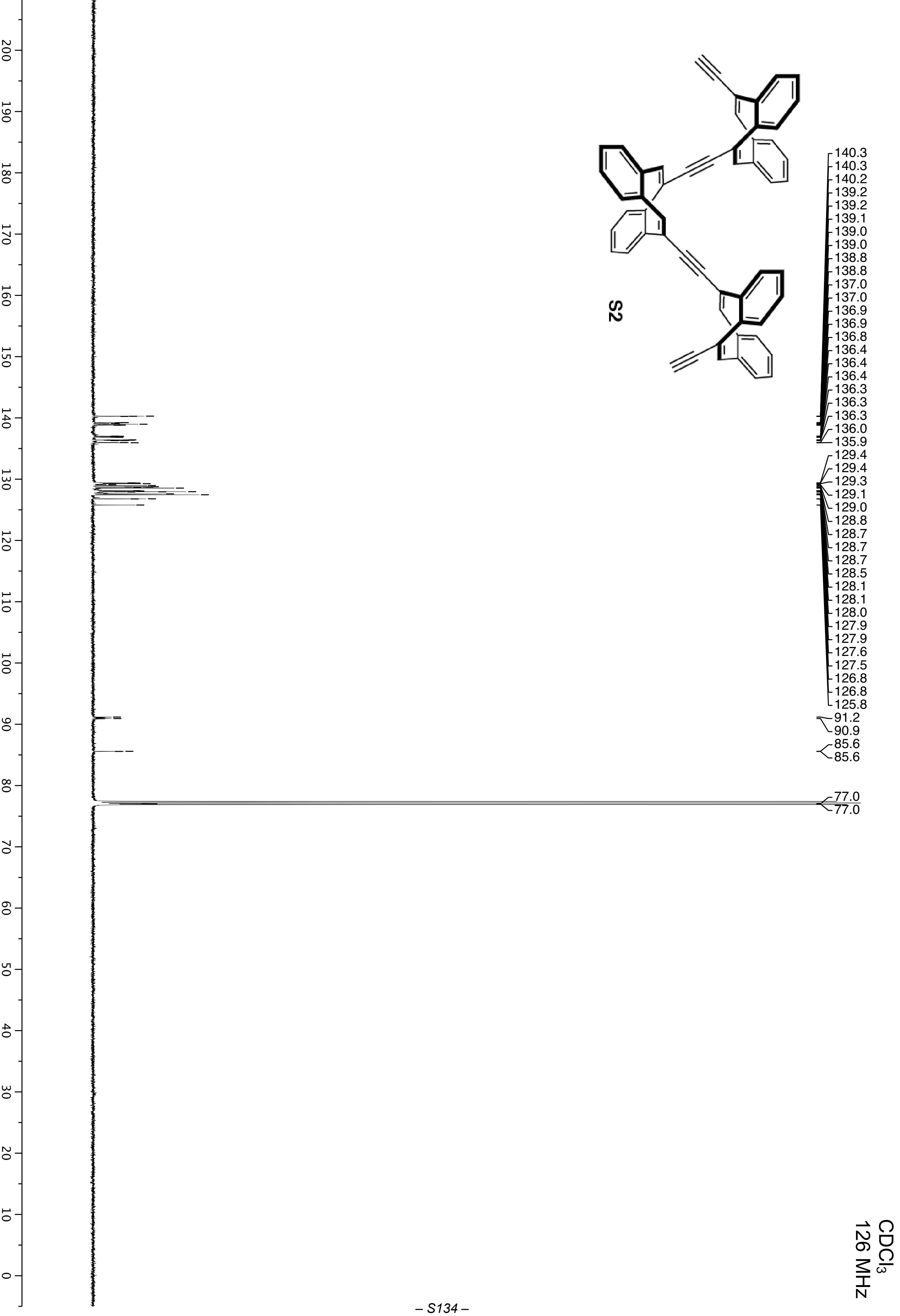
S2
140.3
140.3
140.2
139.2
139.2
139.1
139.0
139.0
138.8
138.8
137.0
137.0
136.9
136.9
136.8
136.4
136.4
136.4
136.3
136.3
136.3
136.0
135.9
129.4
129.4
129.3
129.1
129.0
128.8
128.7
128.7
128.7
128.5
128.1
128.1
128.0
127.9
127.9
127.6
127.5
126.8
126.8
125.8
91.2
90.9
85.6
85.6
77.0
77.0
CDCl$_3$
126 MHz
210
200
190
180
170
160
150
140
130
120
110
100
90
80
70
60
50
40
30
20
10
0

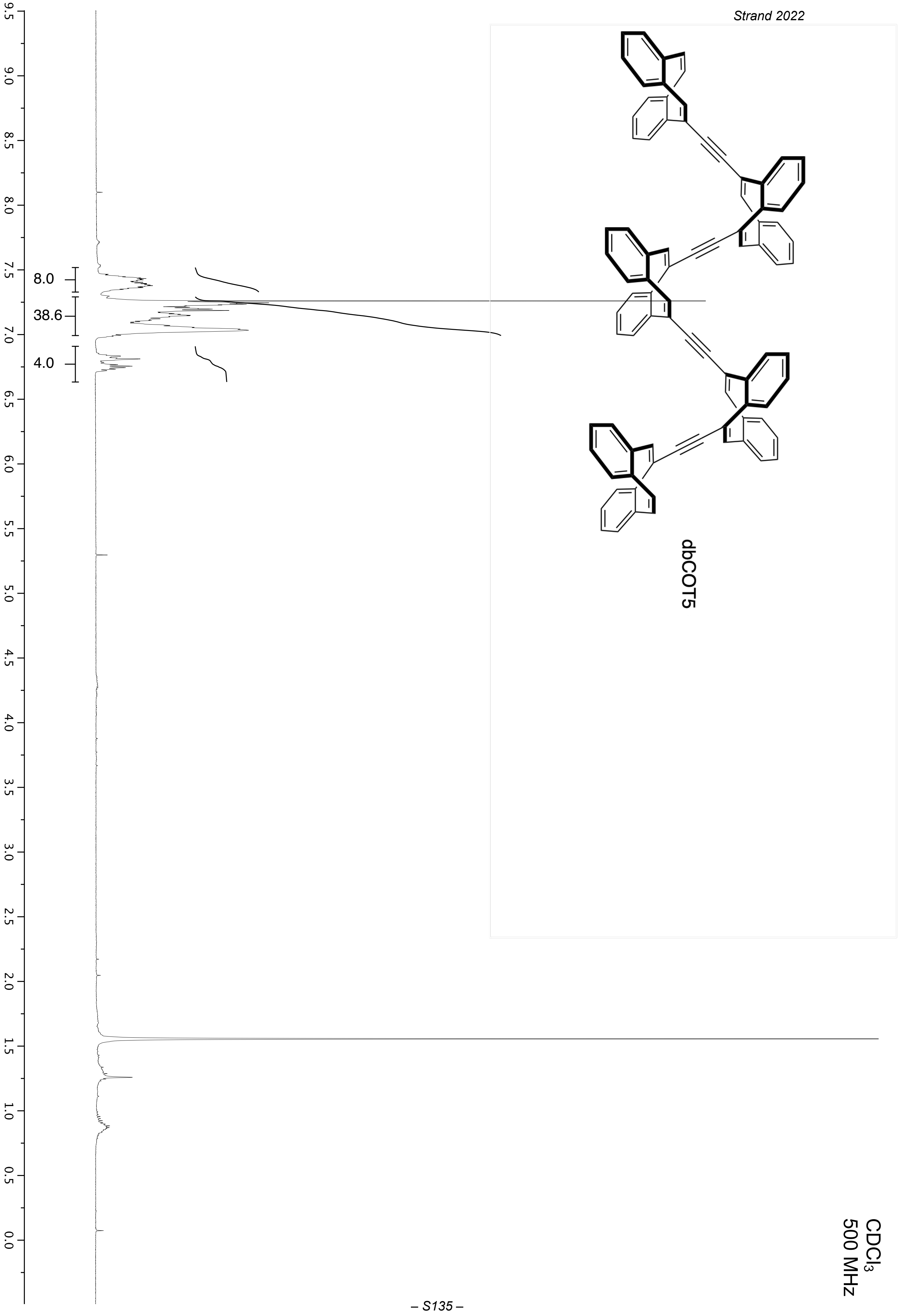

9.5
9.0
8.5
8.0
7.5
7.0
6.5
6.0
5.5
5.0
4.5
4.0
3.5
3.0
2.5
2.0
1.5
1.0
0.5
0.0
8.0
38.6
4.0
dbCOT5
CDCl3
500 MHz

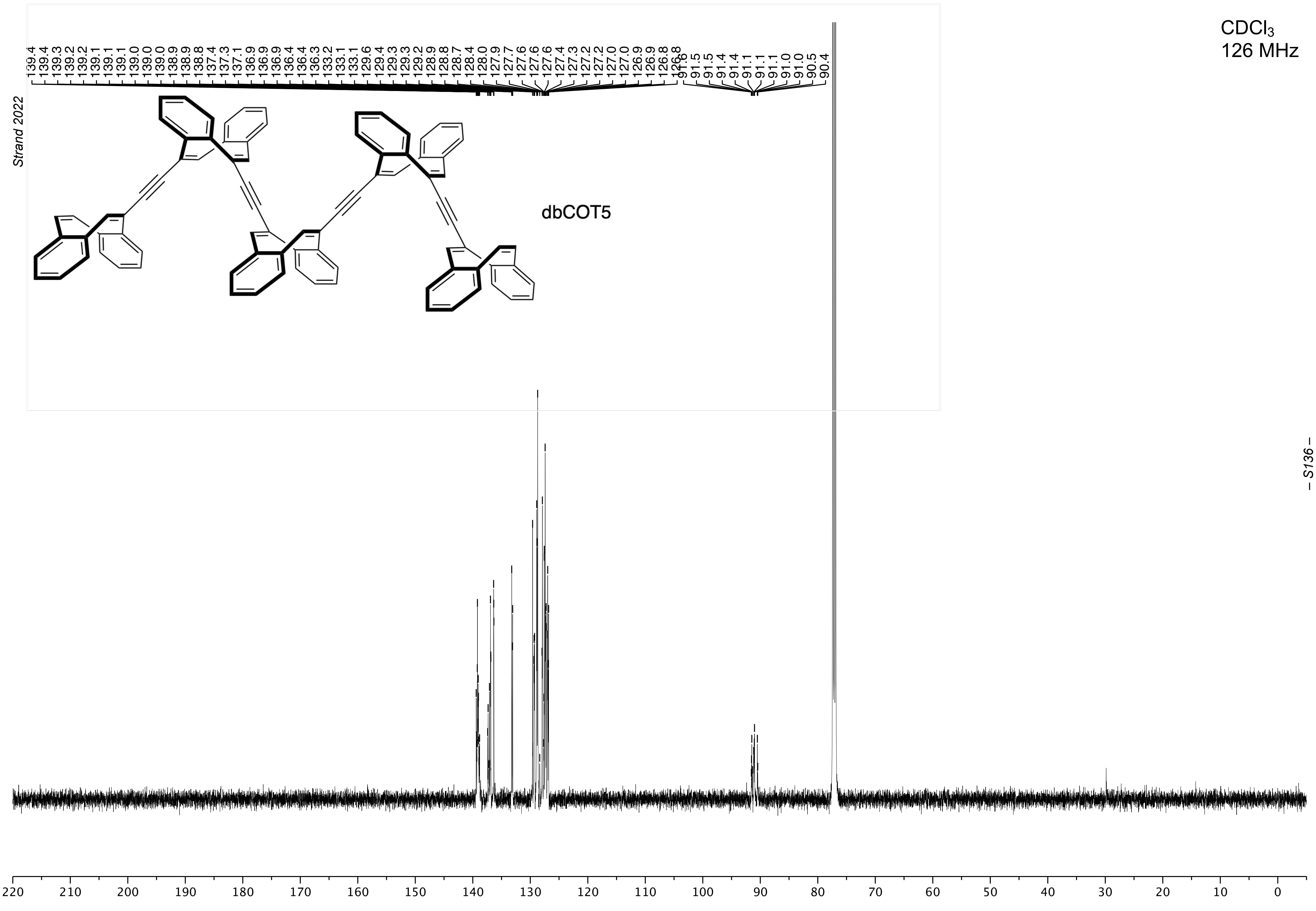
Strand 2022
139.4
139.4
139.3
139.2
139.2
139.1
139.1
139.1
139.0
139.0
139.0
138.9
138.9
138.8
137.4
137.3
137.1
136.9
136.9
136.9
136.4
136.4
136.3
133.2
133.1
133.1
129.6
129.4
129.3
129.3
129.2
128.9
128.8
128.7
128.4
128.0
127.9
127.7
127.6
127.6
127.6
127.4
127.3
127.2
127.2
127.0
127.0
126.9
126.9
126.8
126.8
91.6
91.5
91.5
91.4
91.4
91.1
91.1
91.1
91.0
91.0
90.5
90.4
dbCOT5
CDCl3
126 MHz
220
210
200
190
180
170
160
150
140
130
120
110
100
90
80
70
60
50
40
30
20
10
0

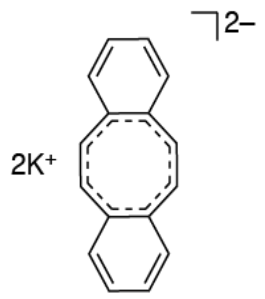

K_2dbCOT

THF-d_8
500 MHz

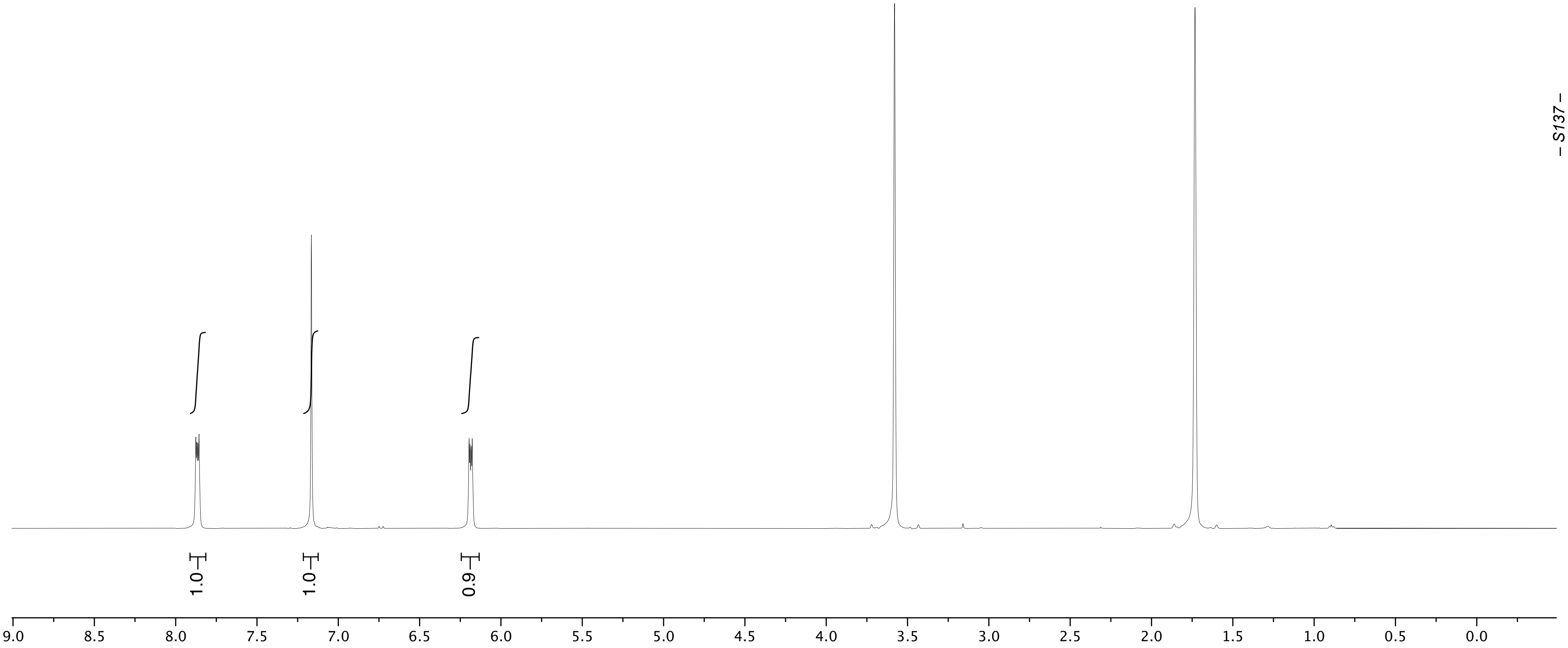

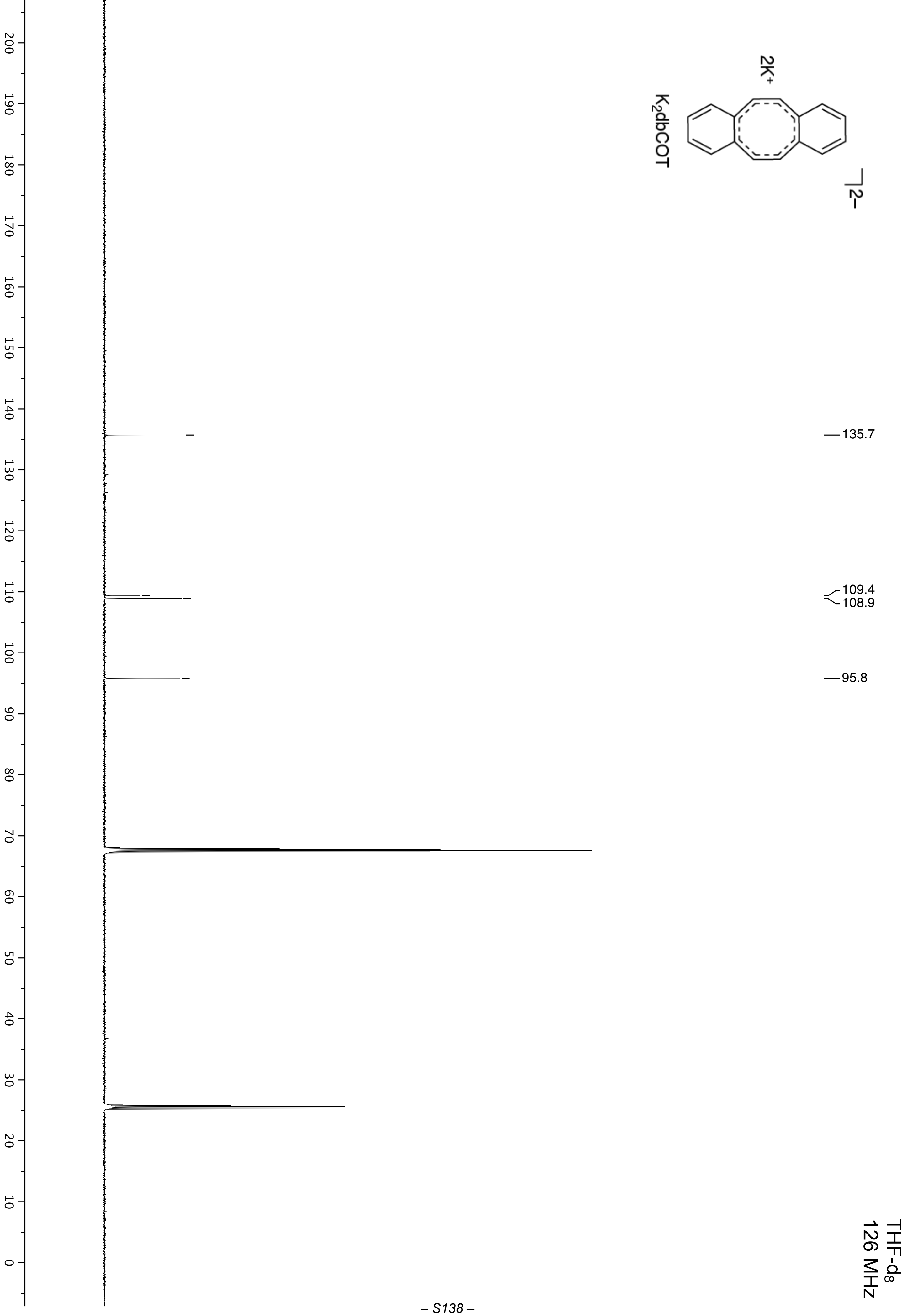

2K+
K2dbCOT
2–
135.7
109.4
108.9
95.8
THF-d8
126 MHz

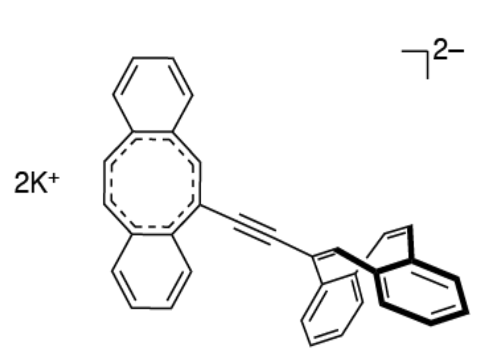
2K+
2–

K_2dbCOT2

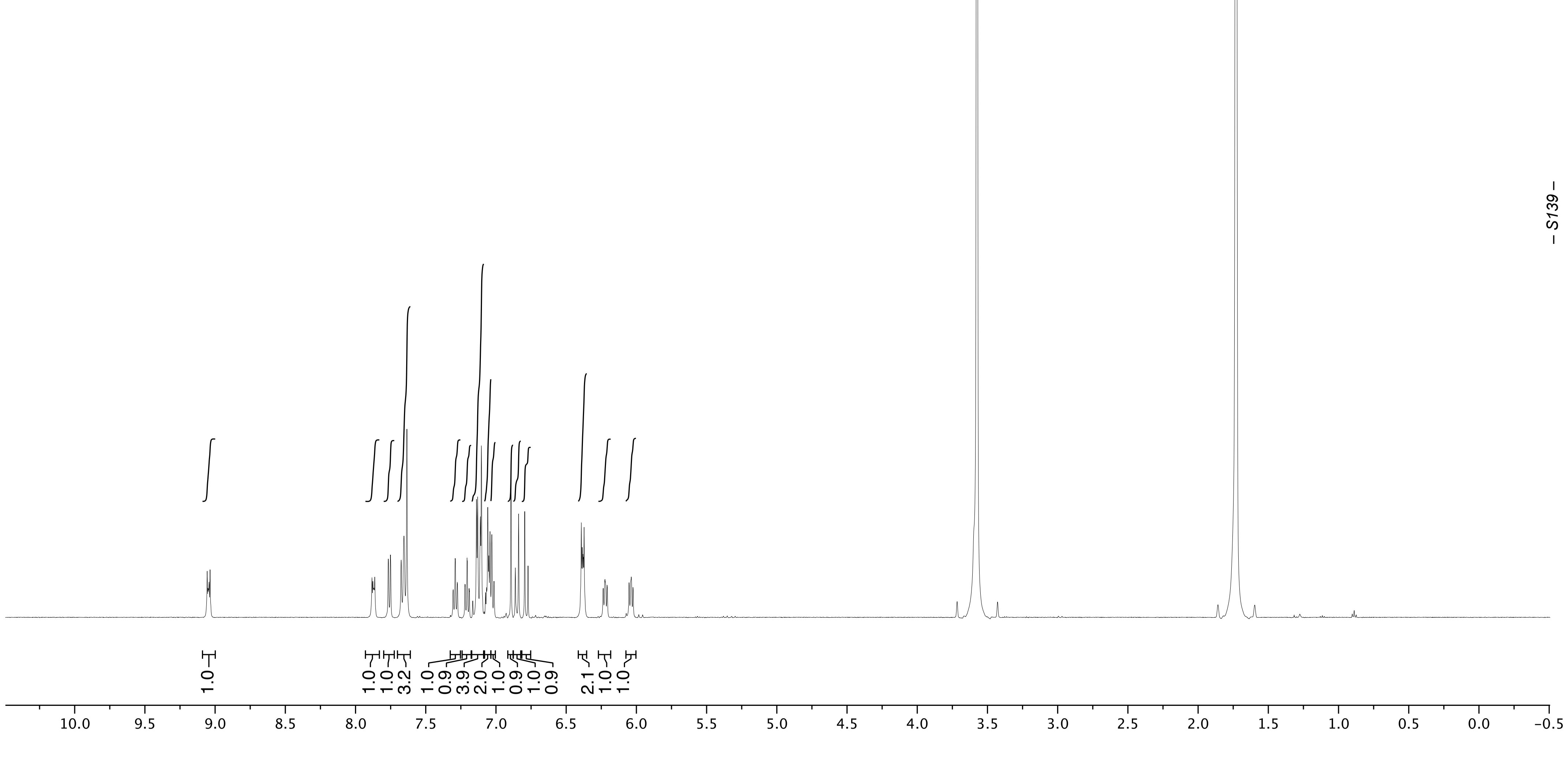
THF-d8
500 MHz
237.2 K
1.0
1.0
1.0
3.2
1.0
0.9
3.9
2.0
1.0
0.9
1.0
0.9
2.1
1.0
1.0
10.0
9.5
9.0
8.5
8.0
7.5
7.0
6.5
6.0
5.5
5.0
4.5
4.0
3.5
3.0
2.5
2.0
1.5
1.0
0.5
0.0
−0.5

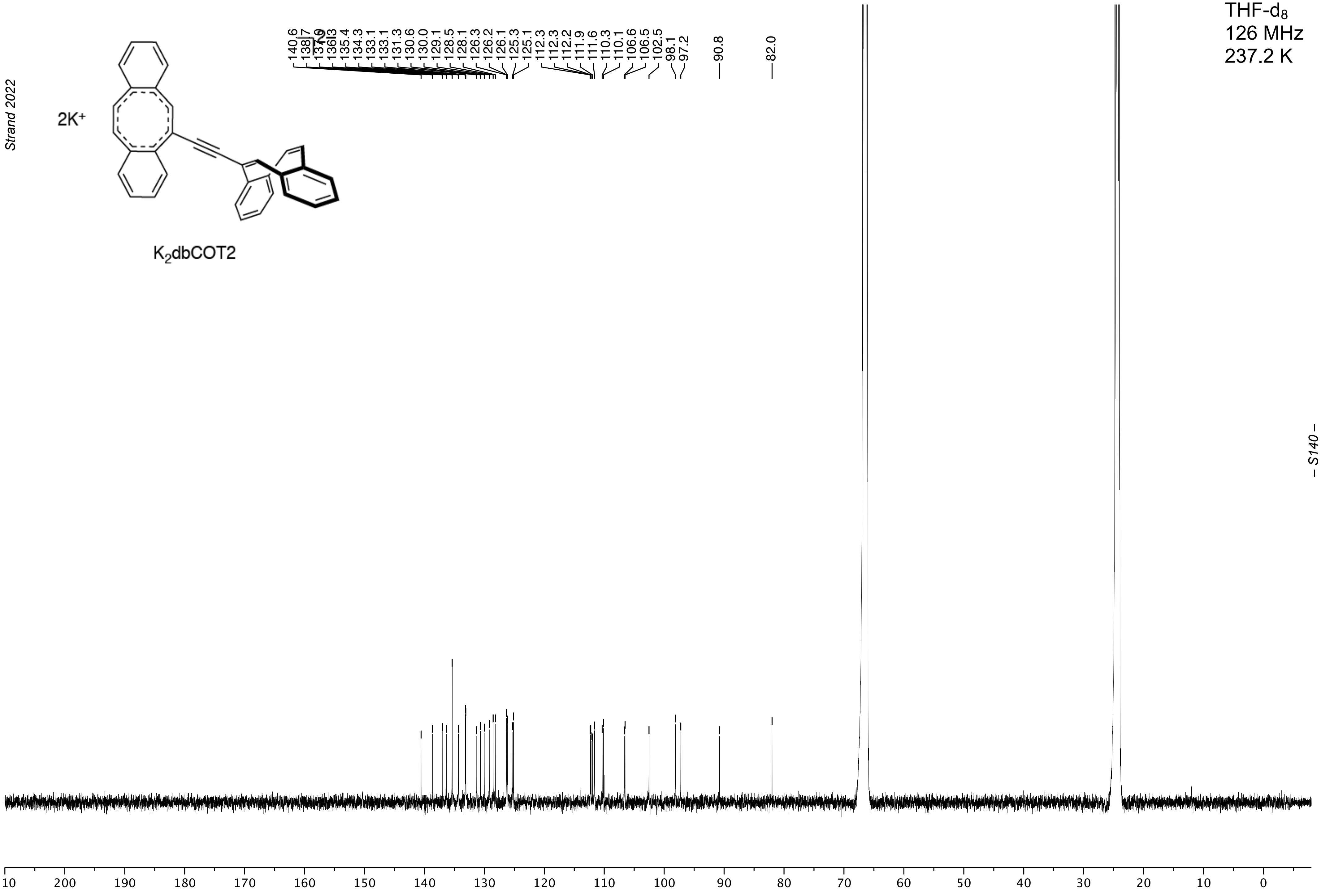
2K+
K2dbCOT2
140.6
135.4
134.3
133.1
133.1
131.3
130.6
130.0
129.1
128.5
128.1
126.3
126.2
126.1
125.3
125.1
112.3
112.3
112.2
111.9
111.6
110.3
110.1
106.6
106.5
102.5
98.1
97.2
90.8
82.0
THF-d8
126 MHz
237.2 K
210 200 190 180 170 160 150 140 130 120 110 100 90 80 70 60 50 40 30 20 10 0

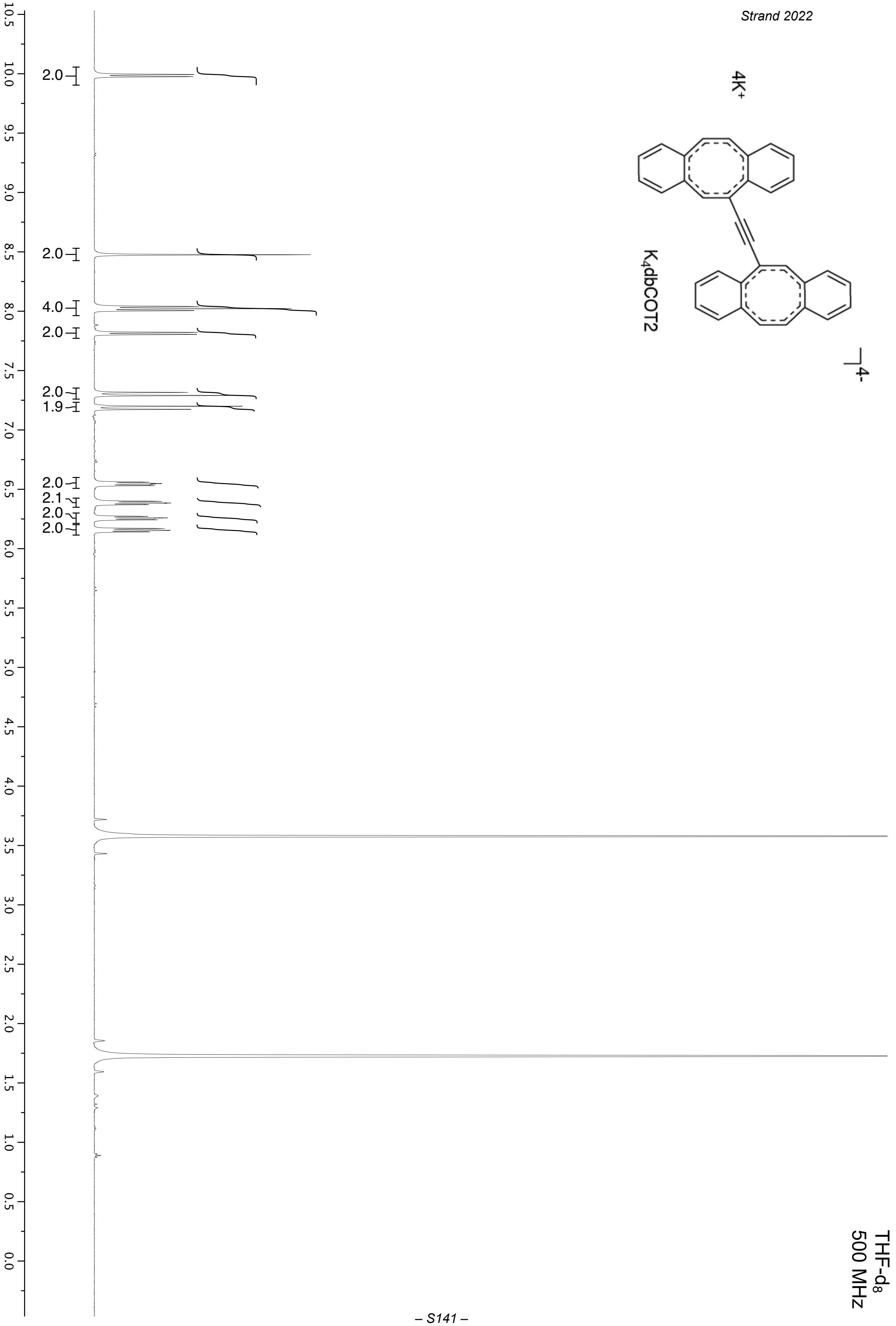
4K+
K4dbCOT2
4-
THF-d8
500 MHz
2.0
2.0
4.0
2.0
2.0
1.9
2.0
2.1
2.0
2.0
10.5
10.0
9.5
9.0
8.5
8.0
7.5
7.0
6.5
6.0
5.5
5.0
4.5
4.0
3.5
3.0
2.5
2.0
1.5
1.0
0.5
0.0

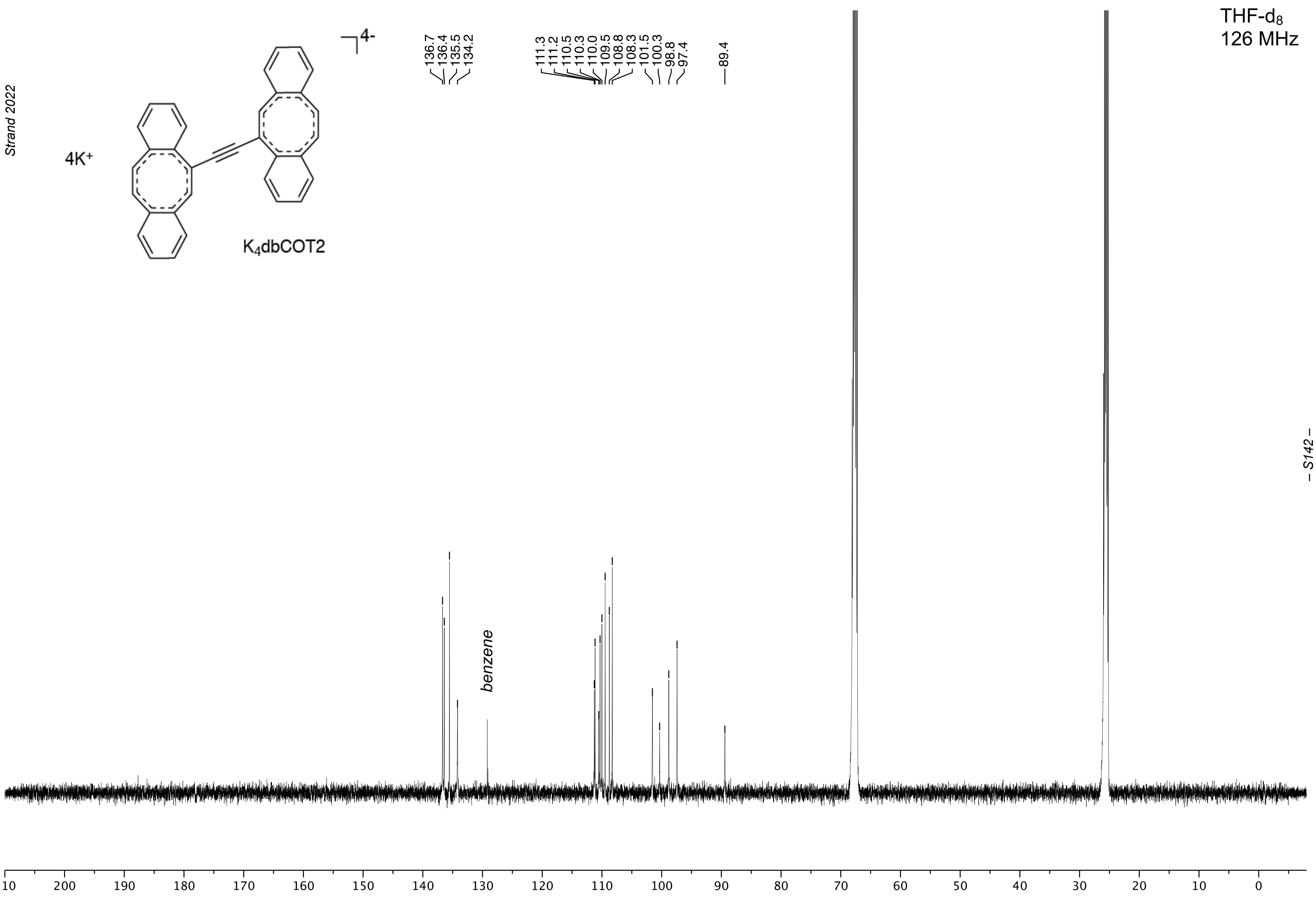
4K+
K4dbCOT2
4-
136.7
136.4
135.5
134.2
111.3
111.2
110.5
110.3
110.0
109.5
108.8
108.3
101.5
100.3
98.8
97.4
89.4
THF-d8
126 MHz
benzene
210
200
190
180
170
160
150
140
130
120
110
100
90
80
70
60
50
40
30
20
10
0

Strand 2022

THF-d_8
500 MHz
223.2 K

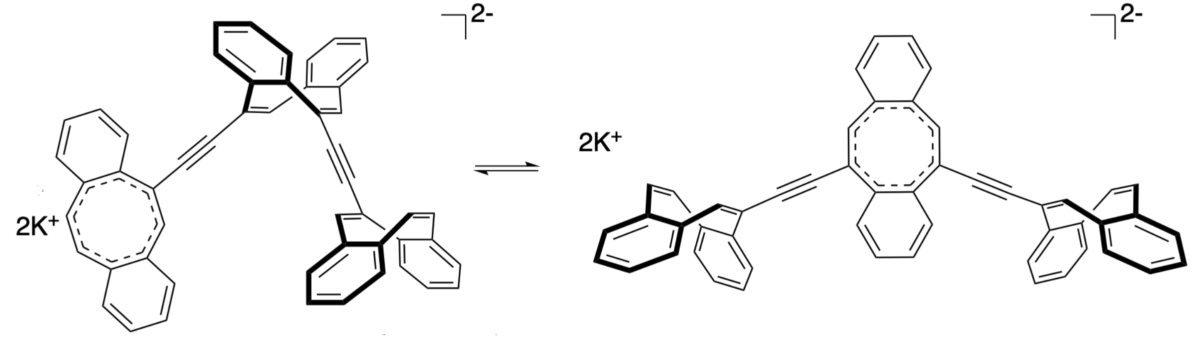

K_2dbCOT3

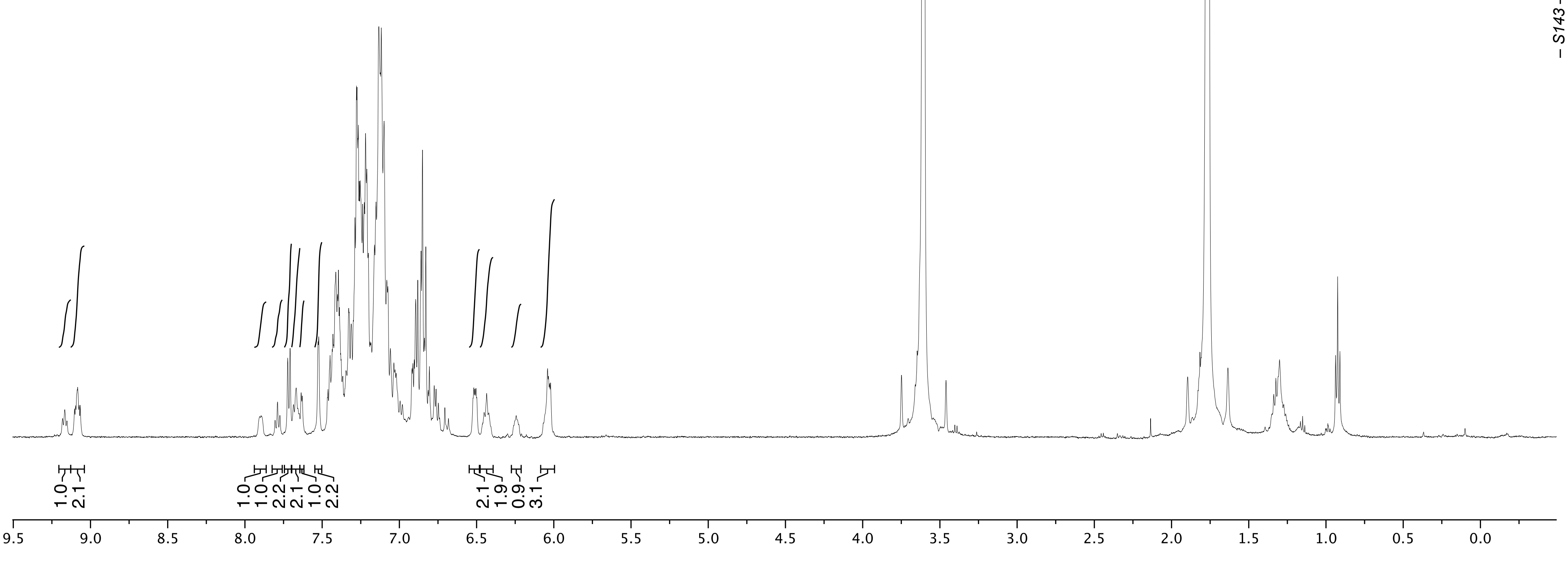

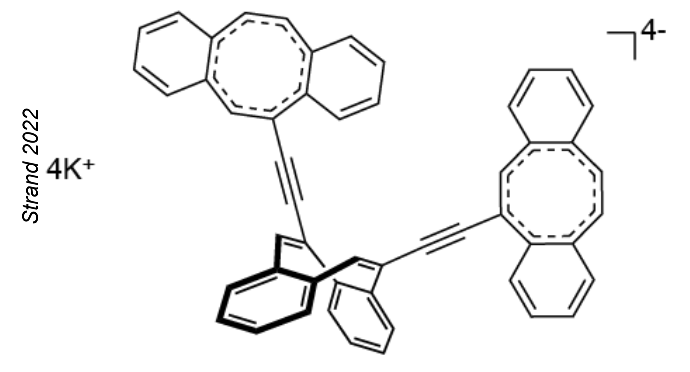

K$_4$dbCOT3

THF-d$_8$
500 MHz

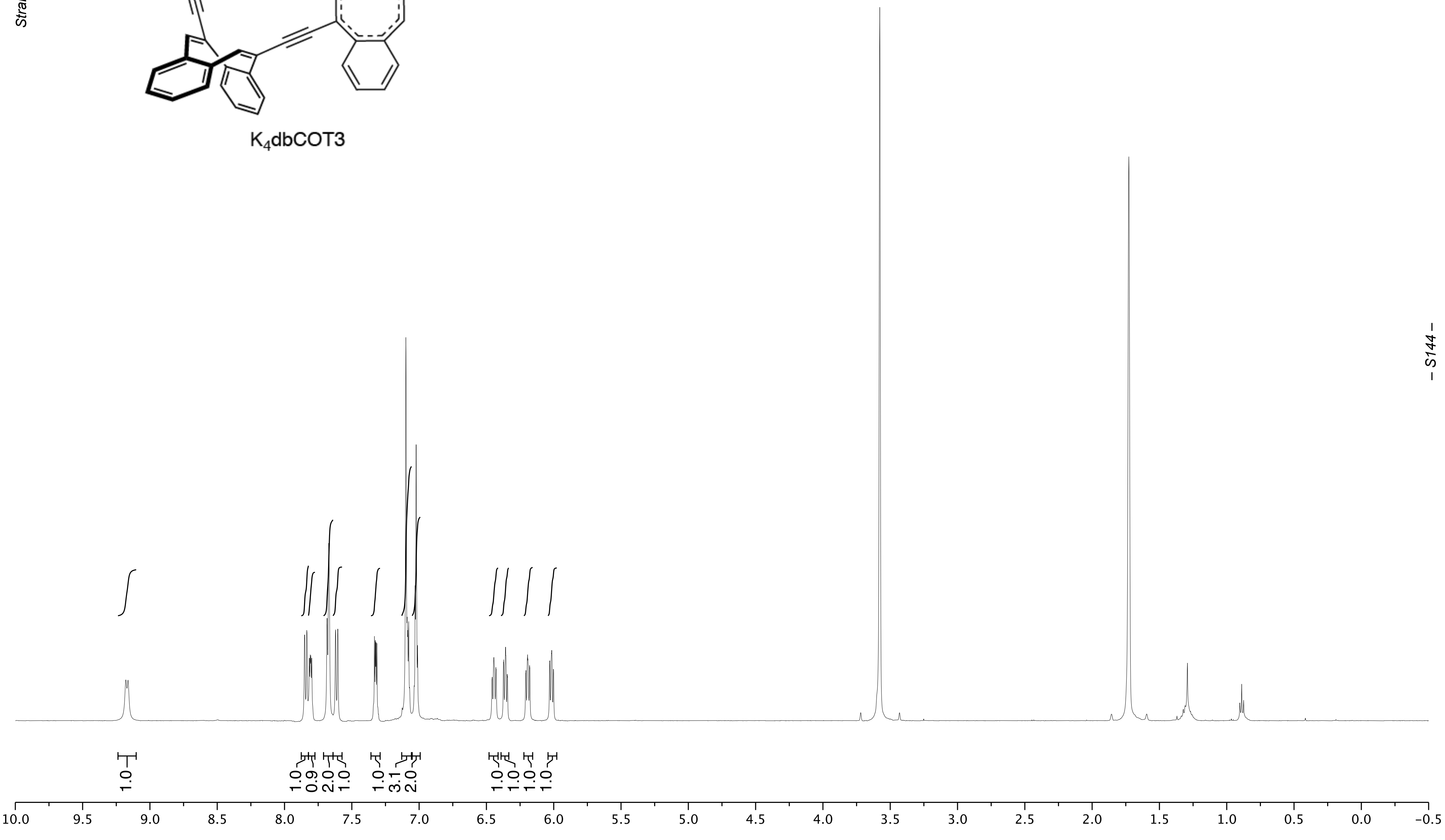

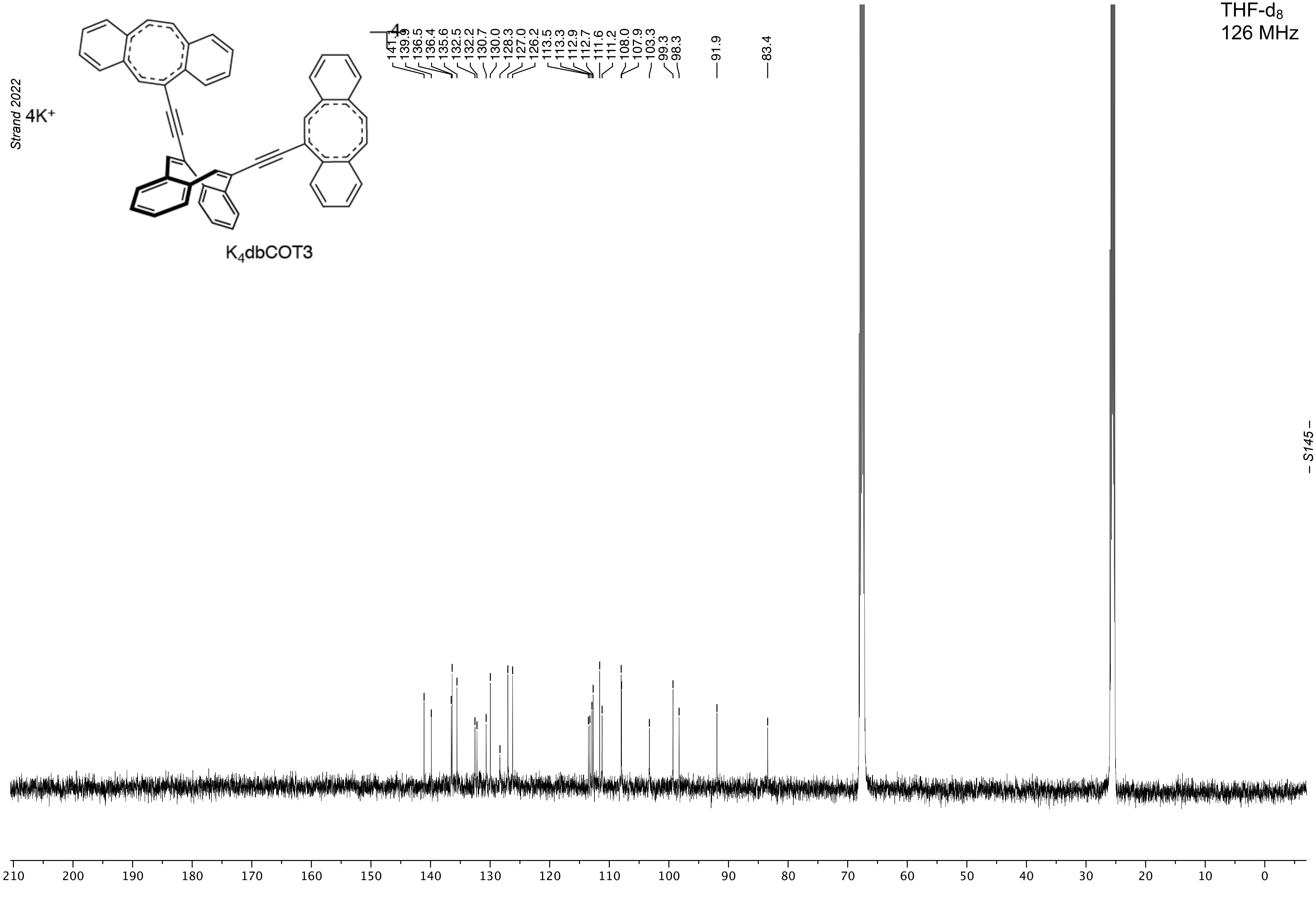
Strand 2022
4K+
K4dbCOT3
141.1
139.9
136.5
136.4
135.6
132.5
132.2
130.7
130.0
128.3
127.0
126.2
113.5
113.3
112.9
112.7
111.6
111.2
108.0
107.9
103.3
99.3
98.3
91.9
83.4
THF-d8
126 MHz
210 200 190 180 170 160 150 140 130 120 110 100 90 80 70 60 50 40 30 20 10 0

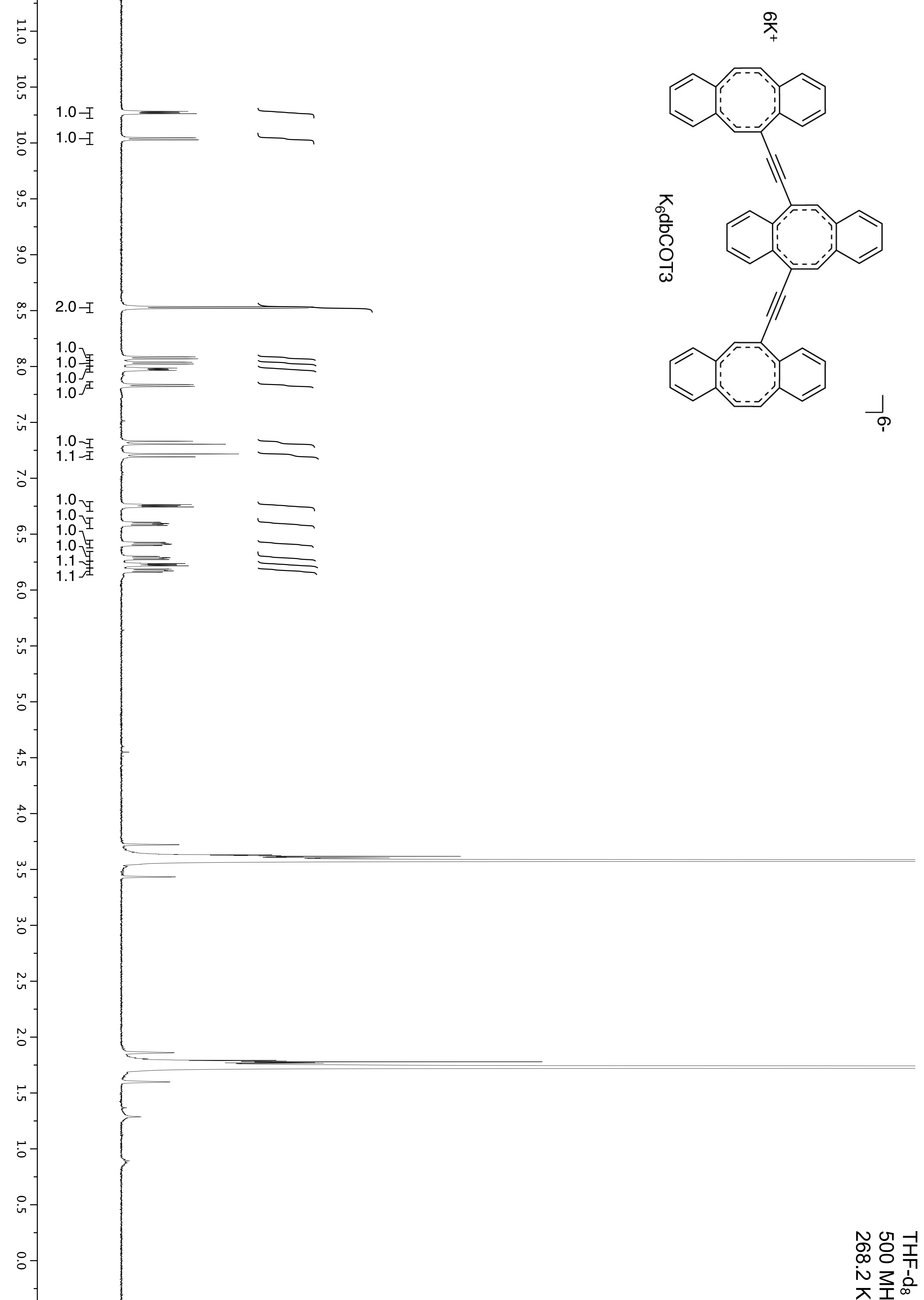
6K+
K6dbCOT3
6-
THF-d8
500 MHz
268.2 K

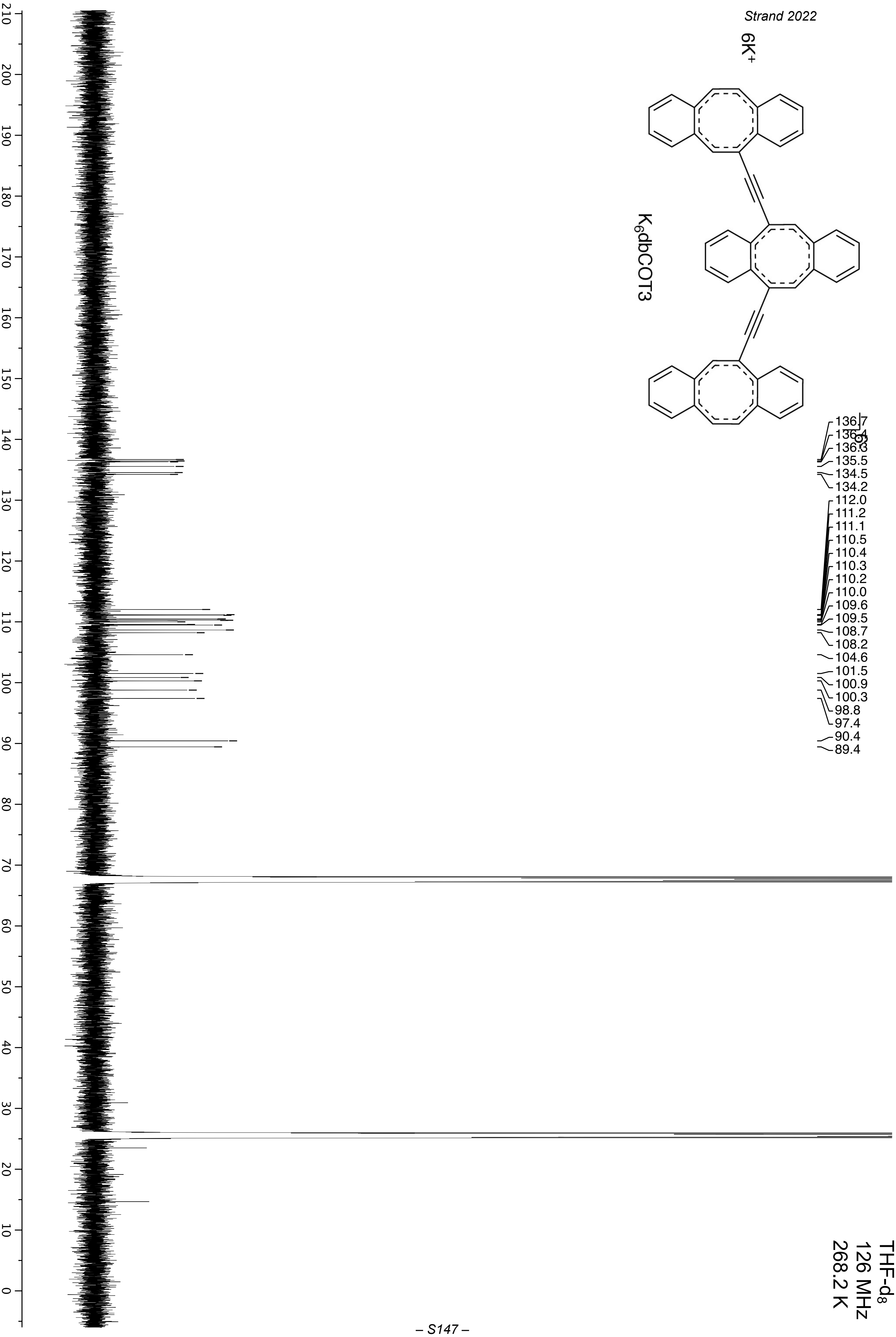
Strand 2022
6K+
K6dbCOT3
136.7
136.4
136.3
135.5
134.5
134.2
112.0
111.2
111.1
110.5
110.4
110.3
110.2
110.0
109.6
109.5
108.7
108.2
104.6
101.5
100.9
100.3
98.8
97.4
90.4
89.4
THF-d8
126 MHz
268.2 K

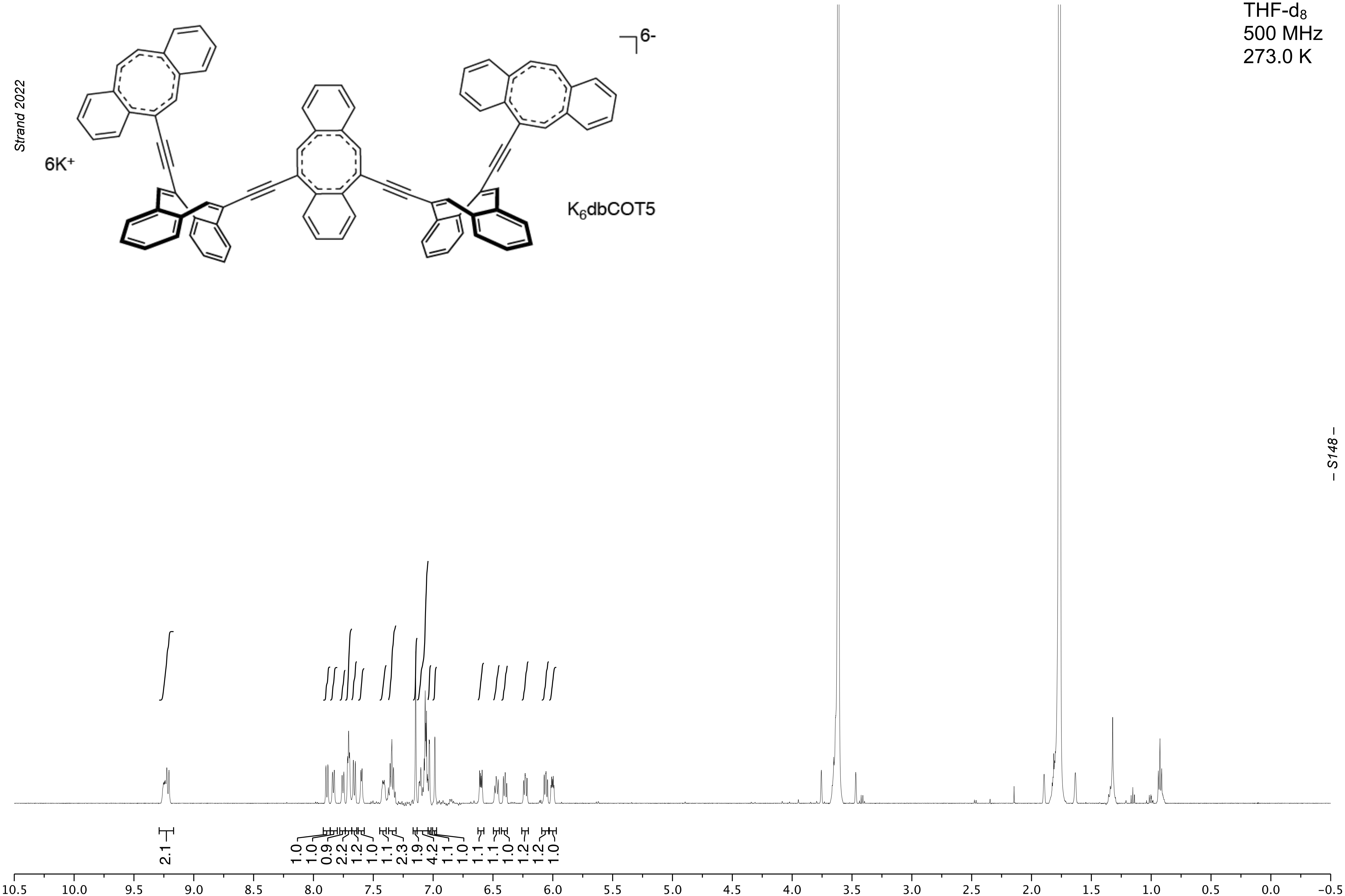
Strand 2022
6K+
6-
K6dbCOT5
THF-d8
500 MHz
273.0 K
2.1
1.0
1.0
0.9
2.2
1.2
1.0
1.1
2.3
1.9
4.2
1.1
1.0
1.1
1.1
1.0
1.2
1.2
1.0
10.5
10.0
9.5
9.0
8.5
8.0
7.5
7.0
6.5
6.0
5.5
5.0
4.5
4.0
3.5
3.0
2.5
2.0
1.5
1.0
0.5
0.0
−0.5

XV. Supplementary references

1. Still WC, Kahn M, Mitra A. Rapid Chromatographic Technique for Preparative Separations with Moderate Resolution. *J. Org. Chem.* 1978, **43**(14)**:** 2923-2925.

2. Chaffins S, Brettreich M, Wudl F. An efficient synthesis of dibenzocycloocta-4a,6a,-diene-5,11-diyne and its precursors. *Synthesis-Stuttgart* 2002(9)**:** 1191-1194.

3. Kojima H, Bard AJ, Wong HNC, Sondheimer F. Electrochemical Reduction of Sym-Dibenzocyclooctatetraene, Sym-Dibenzo-1,5-Cyclooctadiene-3,7-Diyne, and Sym-Dibenzo-1,3,5-Cyclooctatrien-7-Yne. *J. Am. Chem. Soc.* 1976, **98**(18)**:** 5560-5565.

4. Stott K, Keeler J, Van QN, Shaka AJ. One-dimensional NOE experiments using pulsed field gradients. *J. Magn. Reson.* 1997, **125**(2)**:** 302-324.

5. Ammann C, Meier P, Merbach A. A simple multinuclear NMR thermometer. *J. Magn. Reson. (1969)* 1982, **46**(2)**:** 319-321.

6. Hu H, Krishnamurthy K. Revisiting the initial rate approximation in kinetic NOE measurements. *J. Magn. Reson.* 2006, **182**(1)**:** 173-177.

7. Ruckebusch C, Sliwa M, Pernot P, de Juan A, Tauler R. Comprehensive data analysis of femtosecond transient absorption spectra: A review. *J. Photochem. Photobiol. C-Photochem. Rev.* 2012, **13**(1)**:** 1-27.

8. van Stokkum IH, Larsen DS, van Grondelle R. Global and target analysis of time-resolved spectra. *Biochim. Biophys. Acta* 2004, **1657**(2-3)**:** 82-104.

9. Becke AD. Density-functional thermochemistry. III. The role of exact exchange. *The J. C. Phys.* 1993, **98**(7)**:** 5648-5652.

10. Ditchfield R, Hehre WJ, Pople JA. Self-Consistent Molecular-Orbital Methods. IX. An Extended Gaussian-Type Basis for Molecular-Orbital Studies of Organic Molecules. *J. Chem. Phys.* 1971, **54**(2)**:** 724-728.

11. Tomasi J, Mennucci B, Cammi R. Quantum mechanical continuum solvation models. *Chem. Rev.* 2005, **105**(8)**:** 2999-3093.

12. Frisch MJ, Trucks GW, Schlegel HB, Scuseria GE, Robb MA, Cheeseman JR*, et al.* Gaussian 16 Rev. C.01. Wallingford, CT; 2016.
